# Kinetic Resolution of Heterocyclic Lactams by a Photocatalytic Cobalt-Catalyzed Dehydrogenation

**DOI:** 10.1021/jacs.5c07524

**Published:** 2025-07-10

**Authors:** Chao Zhou, Thorsten Bach

**Affiliations:** † School of Natural Sciences, Department Chemie, and Catalysis Research Center (CRC), Technische Universität München, Lichtenbergstrasse 4, 85747 Garching, Germany

## Abstract

Chiral heterocyclic lactams have been kinetically resolved in a photochemical process that involves selective hydrogen abstraction by a chiral benzophenone catalyst. Recognition of one enantiomer is achieved by a two-point hydrogen bonding interaction that directs the reactive carbonyl group of the photocatalyst to the C–H bond at the stereogenic center within the lactam. The generated radical is converted to an oxidized product in a cobaloxime-catalyzed dehydrogenation reaction. The unreactive enantiomer is retained and isolated in enantiomerically enriched form (21 examples, 31–56% yield, 90–99% ee).

## Graphical abstract

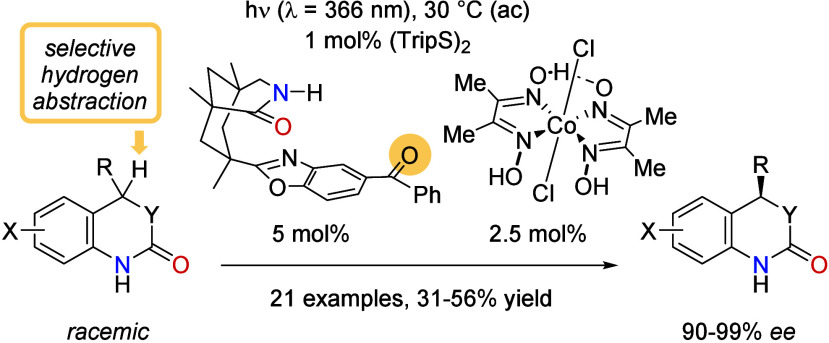

Diarylketones, such as benzophenone,^1^ reach upon direct excitation rapidly and with high quantum yield their lowest lying triplet state (T_1_). Typically, the T_1_ state is an nπ* state, rendering the oxygen atom of the carbonyl group electron deficient and highly reactive. If a suitable reaction partner is present, abstraction of a hydrogen atom is facile and converts the ketone to a protonated ketyl radical. It has been estimated that any C–H bond with a bond dissociation energy below 450 kJ mol^–^^1^ (108 kcal mol^–^^1^) can be cleaved by hydrogen atom transfer (HAT)^2^ to benzophenone.^3^

Recently, there has been increasing interest in chiral small molecules that enable an enantioselective HAT in the excited state. Based on fundamental studies by Roberts and co-workers on an amine-based kinetic resolution by HAT,^4^ the Phipps group has devised a chiral quinuclidine as catalyst that can act as an enantioselective HAT reagent upon oxidative single electron transfer. The catalyst has been successfully employed in the epimerization and oxidative desymmetrization of *meso*-diols.^5^ Our group has designed diarylketones, the benzoyl group of which is linked via a benzoxazole to a chiral azabicyclo[3.3.1]­nonan-2-one backbone.^6^ The compounds are competent to recognize a given substrate enantiomer which can be involved in a cascade of HAT and back HAT leading to a photochemical deracemization.^6b,7^ While this process requires a suitable structural element for back HAT, we hypothesized that the recognition properties could also be applied to compounds which allow exclusively a forward HAT (Scheme 1). If the substrate displays a binding site for recognition by a chiral benzophenone derivative, the forward HAT might be combined with a consecutive ablative reaction leading to an oxidized product. As a consequence, the enantiomer that is not susceptible to hydrogen abstraction is enriched (Scheme 1, top). If the concept was successful, it should lead to a photochemically driven^8,9^ kinetic resolution,^10^ in which one substrate enantiomer was not processed while the other substrate enantiomer was oxidized. We considered cobaloximes^11,12^ as possible cocatalysts which would enable the oxidation of a carbon-centered radical with concomitant hydrogen evolution. 4-Substituted 3,4-dihydroquinolin-2­(1*H*)-ones (dihydroquinolones), *rac*-**1**, were identified as substrates which could be kinetically resolved by chiral ketone **2**.

**Scheme 1:**
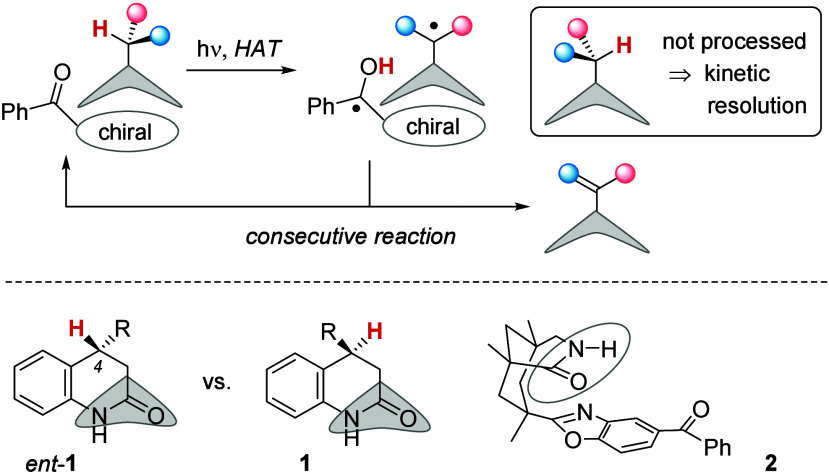
Concept for a Kinetic Resolution Based on the Selective Recognition of a Substrate Enantiomer by a Chiral Photocatalyst

Hydrogen bonding to the lactam site of the catalyst would expose the hydrogen atom at carbon atom C4 of enantiomer *ent*-**1** to a HAT by the photocatalyst (Scheme 1, bottom). The hydrogen atom at the same position in enantiomer **1** is not available for a HAT in a substrate–catalyst complex. Once the hydrogen atom was removed from position C4 in complex *ent*-**1**·**2**, the ensuing complex **3**·**4** was envisioned to enter a cobaloxime-catalyzed dehydrogenation reaction (Scheme 2). The required active cobalt catalyst, CoCl­(dmgH)_2_ (**7**), is typically generated *in situ* from appropriate precursors such as CoCl_2_(dmgH)­(dmgH_2_) (**7**·HCl) or CoCl­(dmgH)_2_(py) (**7**·py) which are both commercially available (dmgH_2_ = dimethylglyoxime, py = pyridine).

**Scheme 2:**
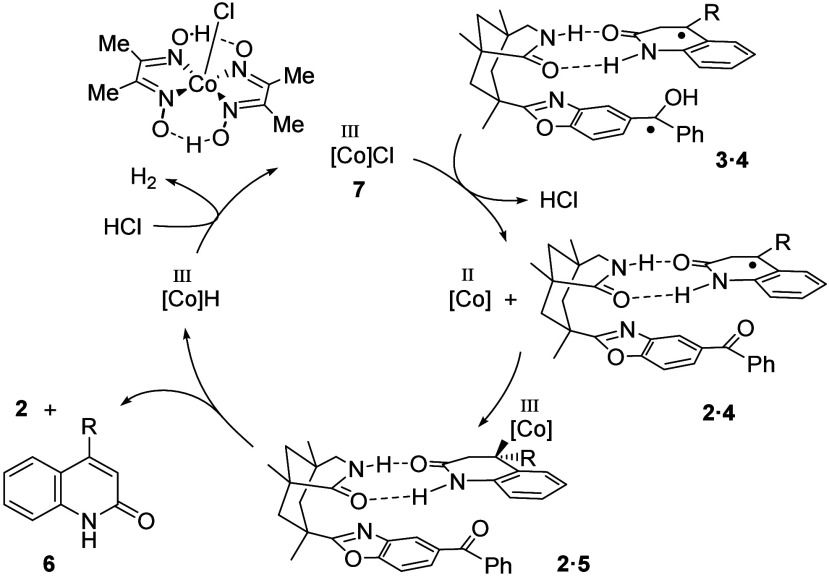
Mechanistic Hypothesis for the Cobalt-Catalyzed Dehydrogenation of Dihydroquinolones **1**

Oxidation of the protonated ketyl radical leads to the formation of a cobalt­(II) complex and restores photocatalyst **2**. Combination of the cobalt­(II) fragment with radical **4** generates intermediate **5** which is competent to undergo β-hydride elimination. The oxidation product of the dihydroquinolone is in the described scenario quinolin-2­(1*H*)-one (quinolone) **6** and a cobalt­(III) hydrido complex. The latter is known to undergo protonation^11^ by an acid, here hydrochloric acid, to close the catalytic cycle and to generate hydrogen as the byproduct of the oxidation reaction. Since 4-substituted quinolones are known to undergo a facile hydrogenation to racemic dihydroquinolones *rac*-**1**, the starting material can be regenerated, and the resolution process can be iterated.

Based on the described hypothesis, initial screening started with 4-phenyl-substituted substrate *rac*-**1a** and cobalt catalyst **7**·py in acetonitrile solution (see Supporting Information for details). Yield analysis was performed by NMR and hinted at a successful kinetic resolution (42%, 95% ee) to be achieved with 1 mol % of the cobalt catalyst and 10 mol % of photocatalyst **2**. Further screening on preparative runs showed an improvement of the yield, if a thiol or disulfide was added (Trip = 2,4,6-triisopropylphenyl) and if cocatalyst **7**·HCl was employed in acetone (ac) solution. Two sets of conditions evolved from the optimization studies, with conditions A being favorable due to the lower catalyst loading (Scheme 3). In some cases, a higher catalyst loading was required (conditions B) to reach a high level of enantioselectivity.^13^ Typically, the reaction time varied between 3 and 5 h but was extended to up to 24 h, if the enantioselectivity or the conversion remained low. A light emitting diode (LED) with an emission maximum at λ = 366 nm was employed as an irradiation source. Under optimized conditions (40 μmol scale), test substrate *rac*-**1a** delivered the desired enantiomer **1a** in a yield of 46% with 96% ee under conditions A. The result was fully reproducible on larger scale (0.5 mmol) with 48% yield and 96% ee. Conditions B delivered the product in 53% yield with 92% ee. The values are close to the maximum to be achieved in a kinetic resolution (50%, >99% ee) and the calculated selectivity factor^10^ is high (*s* = 80). Other arylated dihydroquinolones behaved similarly and gave the respective products **1b**–**1k** in yields of 31–42% and with 92–98% ee. Functional group tolerance with the halogen substituents fluorine (**1d**, **1h**), chlorine (**1e**, **1g**, **1k**), and bromine (**1f**) was shown as was the compatibility with alkyl (**1c**, **1i**) and ether groups (**1b**, **1j**). In the alkyl series, the size of the substituent was irrelevant to the success of the reaction, and the respective products **1l**–**1r** were obtained in high yields (39–50%) and with excellent enantioselectivity (92–99% ee). Apart from the functional group (lactam) intrinsically present in the substrate, fluorine (**1q**) and silyl (**1r**) groups were shown to be stable under the reaction conditions. A dihydroquinolone with a 4-butyloxycarbonyl substitution successfully underwent the kinetic resolution to yield product **1s** in 41% yield with 90% ee. The absolute configuration of the products was established by comparing the HPLC traces of product **1m** with an authentic sample the configuration of which was known.^14^ The configuration assignment of the other products was based on analogy and matches the suggested differentiation in the HAT transfer step (Scheme 1).

**Scheme 3:**
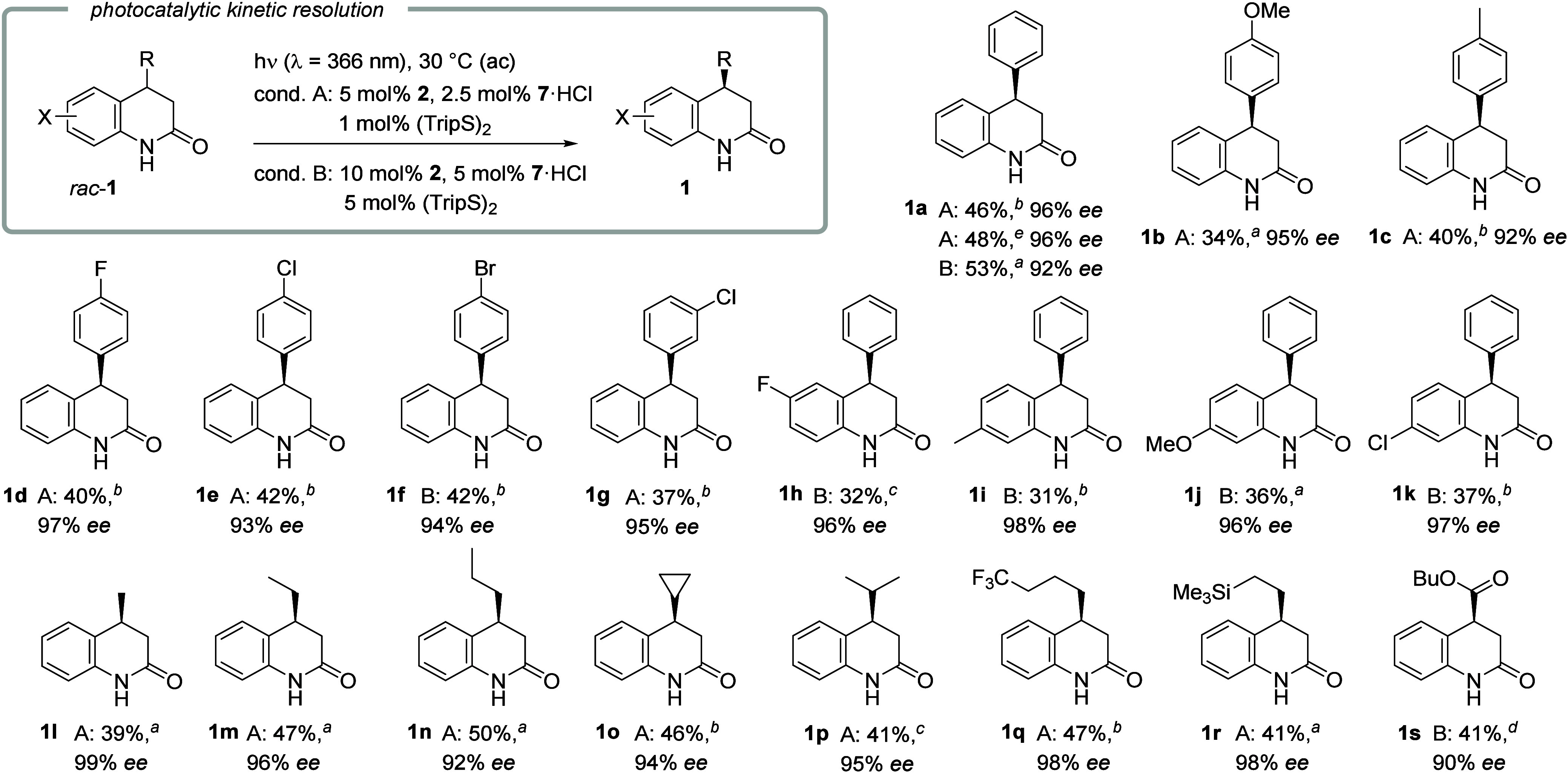
Scope of the Photocatalytic Kinetic Resolution of 4-Substituted Dihydroquinolones (40 μmol Scale)^f^

a Reaction time *t* = 3 h.
b Reaction time *t* = 5 h.
c Reaction time *t* = 15 h.
d Reaction time *t* = 24 h.
e The reaction was performed on 0.5 mmol scale.
f For additional examples, see the Supporting Information. The reactions were performed with Co­(dmgH)­(dmgH_2_)­Cl_2_ (**7**·HCl) and disulfide (TripS)_2_ as co-catalysts in acetone (ac) solution. Two slightly different conditions (A or B) were used, and the conditions that worked best for a given substrate are shown. All yields refer to isolated compounds. The enantiomeric excess (ee) was determined by chiral HPLC analyses.

Mechanistic evidence for the suggested cobalt-catalyzed oxidation was obtained by isolating the respective oxidized quinolones as byproducts of the reaction. In the mentioned 0.5 mmol scale reaction of substrate *rac*-**1a**, quinolone **6a** (R = Ph) was isolated in 39% yield. Also in some of the small scale reactions, the isolation of the byproduct was feasible, e.g., for quinolone **6l** (R = Me, 53% yield) or **6n** (R = Pr, 39% yield). Hydrogenation of the reduced compounds is facile, and it is possible to prepare dihydroquinolones *rac*-**1** from quinolones **6** (see the Supporting Information). By doing so and by repeating the catalytic enantioselective step, the overall yield of the kinetic resolution can be further increased.

For the kinetic resolution *rac*-**1a** → **1a**, dissolving the reaction gas in the headspace of the reaction mixture in acetonitrile-*d*_3_ resulted in a new signal in the ^1^H NMR spectrum which was assigned to molecular hydrogen (δ = 4.57 ppm).^15^ Additional mechanistic conclusions were drawn from the product analysis and control experiments. The yield of isolated quinolone **6o** from the reaction of cyclopropyl substrate *rac*-**1o** was only 28% which could possibly indicate a ring opening of the radical **4o**. Unfortunately, the byproducts were not sufficiently clean to allow for a proper analysis. The kinetic resolution protocol was also applicable to other lactams, such as substrates *rac*-**8** and *rac*-**9** (Scheme 4). In the former case, the product yield slightly exceeds 50% which possibly indicates that the oxidation product is reduced by the cobalt catalyst regenerating some racemic *rac*-**8**.^16^ In fact, if quinolone **6a** was subjected to the reaction conditions employing parent benzophenone as the catalyst (20 mol %) and triethylamine (3 equiv) as reductant, racemic product *rac*-**1a** was obtained albeit in low yield (28%). In the absence of a reductant, a minimal (4%) hydrogen transfer from substrate *rac*-**1a** to quinolone **6s** was recorded (see the Supporting Information for details).

**Scheme 4:**
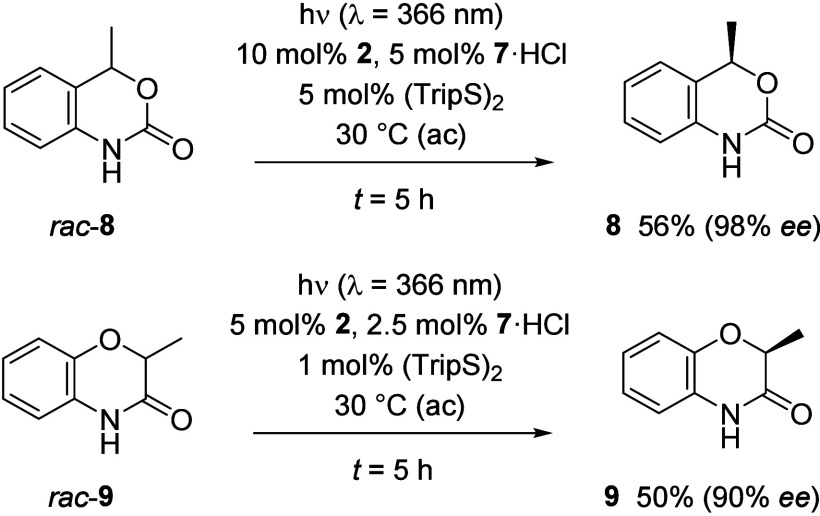
Substrate Variation beyond 4-Substituted Dihydroquinolones: Successful Kinetic Resolution of Substrates *rac*-**8** and *rac*-**9**

By employing enantiomerically pure substrates *ent*-**1a** and *ent*-**1a-** *d*_1_, which are susceptible to HAT by the catalyst, it was possible to determine the kinetic isotope effect (KIE) at position C4 (Scheme 5, top). The initial reaction rates^17^ were measured in separate experiments and provided *k*_H_/*k*_D_ = 2.1 (±0.2). By employing the doubly deuterated substrate *ent*-**1a-** *d*_2_ and comparing it to *ent*-**1a**, a kinetic isotope effect of *k*_H_/*k*_D_ = 1.4 (±0.2) was recorded. The results support a turnover-limiting HAT, in which a hybridization change from sp^3^ to sp^2^ occurs^18^ at carbon atom C4. If the β-hydride elimination **5** → **6** was turnover-limiting, the KIE for *ent*-**1a-** *d*_2_ would be expected to be larger.^12f^ However, it cannot be ruled out that the primary kinetic isotope effect results from a slow proton transfer either in the formation of H­(D)Cl or in the hydrogen evolution step. Circumstantial evidence for the HAT as turnover-limiting step is the low value for the primary kinetic isotope effect at C4 which matches the value obtained for a related HAT process induced by photoexcited catalyst **2**.^6a^ The catalytic cycle proposed in Scheme 2 seems reasonable with the addition of disulfide avoiding catalyst decomposition by thiyl radicals acting as a hydrogen atom shuttle^19^ that avoids any side reaction stemming from protonated ketyl radical **3**.

**Scheme 5:**
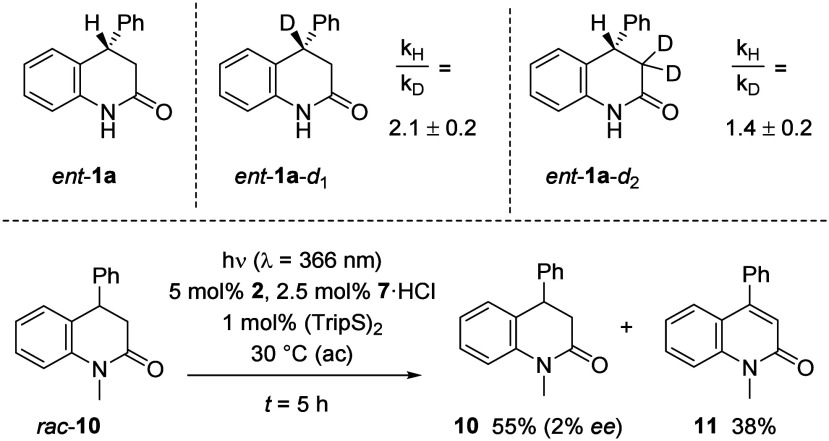
Mechanistic Experiments: Kinetic Isotope Effects and the Importance of a Two-Point Binding Motif

The relevance of the molecular recognition event was corroborated by experiments performed with *N*-methylated analogue *rac*-**10** of substrate *rac*-**1a** (Scheme 5, bottom). The compound underwent dehydrogenation under standard reaction conditions A but delivered the recovered product in racemic form (2% ee) together with the expected quinolone **11** as oxidation product.

The products of the kinetic resolution invite several consecutive reactions, a few of which were pursued with product **1a** as a test case (Scheme 6). Although the phenyl group at C4 renders the position somewhat more reactive, the stereogenic center turned out to be configurationally stable, even at an elevated temperature. Specifically, an alkylation to 3,4-disubstituted product **12** was conducted,^20^ proceeded with perfect diastereoselectivity (dr = diastereomeric ratio), and delivered the desired compound as a single diastereoisomer. Reduction to tetrahydroquinoline **13** was performed in a yield of 71% with the borane–THF complex under refluxing conditions. An *N*-arylation was achieved with phenyl bromide as the electrophile in a Cu-catalyzed reaction (DACH = diaminocyclohexane)^21^ and gave product **14** in 80% yield. As an example for another conversion at C2, a thiocarbonylation was conducted with the Lawesson reagent [2,4-bis­(4-methoxyphenyl)-1,3,2,4-dithiadiphosphetane-2,4-disulfide][Bibr ref22] in refluxing toluene providing product **15** in 65% yield.

**Scheme 6:**
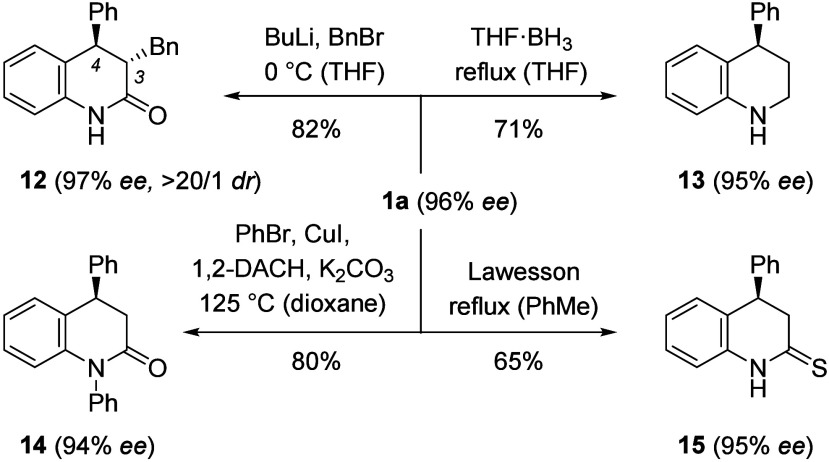
Downstream Reactions Performed with Enantiomerically Enriched Product **1a** Occurring with Retention of Configuration

In summary, it has been successfully demonstrated that chiral benzophenone **2** acts as a suitable reagent for a selective hydrogen abstraction at nonactivated positions. The resulting radical pair appears to be sufficiently stable to undergo consecutive reactions, in the given example, a cobalt-catalyzed dehydrogenation reaction. Although preliminary data have been obtained that support the intermediacy of molecular assemblies **3**·**4**, **2**·**4**, and **2**·**5**, additional information on their structure and their lifetimes are desirable. Synthetically, the high enantioselectivity exerted by chiral benzophenones, such as **2**, invites future applications of HAT reactions in the construction of defined stereogenic centers.

## Supplementary Material



## Data Availability

The data that supports the findings of this study are available in the Supporting Information of this article. Primary research data are openly available in the repository RADAR4Chem at DOI: 10.22000/tk02zqa61419y4gj.

## Notes

Experimental procedures, analytical data and NMR spectra for all new compounds, HPLC traces of all chiral compounds, details of the optimization and mechanistic experiments (PDF)
