# Supplementary material for: Kinetic Resolution of Heterocyclic Lactams by a Photocatalytic Cobalt-Catalyzed Dehydrogenation
Source: J Am Chem Soc. 2025 Jul 10;147(29):25148–52. doi: 10.1021/jacs.5c07524 (PMC12291455; doi:10.1021/jacs.5c07524)
Supplement: Supplementary file 1 [file ja5c07524_si_001.pdf]

Supporting Information for

Kinetic Resolution of Heterocyclic Lactams by a Photocatalytic Cobalt-Catalyzed Dehydrogenation

Chao Zhou,[a] Thorsten Bach[a]*

[a] School of Natural Sciences, Department Chemie, and Catalysis Research Center (CRC), Technische Universität München, Lichtenbergstrasse 4, 85747 Garching, Germany;

Correspondence to: thorsten.bach@ch.tum.de

Table of Contents

1. General Information 3
2. Analytical Methods 6
3. General Procedures 8
4. Condition Optimization 13
5. Mechanistic Studies 14
6. Substrate Synthesis 19
7. Photochemical Deracemization Reactions 38
8. NMR Spectra 53
9. Chiral HPLC Traces 97
10. References 125

1. General Information

All reactions sensitive to air or moisture, were carried out in flame-dried glassware under positive pressure of argon using standard Schlenk techniques.

Commercially available chemicals were used without further purification, if not further mentioned. For moisture sensitive reactions, dichloromethane (DCM) and tetrahydrofuran (THF) were purified using a MBSPS 800 *MBraun* solvent purification system. The following columns were used:

CH_2Cl_2: 2 × MB-KOL-A type (aluminium oxide)

THF: 2 × MB-KOL-M type 2 (3 Å molecular sieve)

Anhydrous *α,α,α*-trifluorotoluene ($PhCF_3$) was purchased from *Sigma Aldrich (Merck).* $PhCF_3$ was additionally stored over 3 Å molecular sieves. Anhydrous acetonitrile (MeCN), dimethylformamide (DMF), methanol (MeOH) and toluene ($PhCH_3$), were purchased from *Thermo Fisher Scientific* and stored over 3 Å molecular sieves.

Technical solvents for column chromatography [acetone (acetone), chloroform (CH_3Cl), ethyl acetate (EtOAc), methanol (MeOH), *n*-pentane (Pn)] were used after simple distillation.

Normal-phase flash column chromatography (FCC) was performed on silica 60 (*Merck*, 230-400 mesh) with the indicated eluent mixture.

Commercially available chemicals were purchased either from *Sigma Aldrich (Merck)*, *TCI Chemicals*, *ABCR* or *BLDpharm* and were used without further purification, if not further mentioned.

Unless otherwise stated, initial screenings and optimized photochemical reactions were carried out in crimp-cap vials using a 3 W 366 nm LED (High-Power-LED - Single Color LuxiGen LZ1 MCPCB) (Figure S1). The LED setup is depicted in Figure S2. Prior to the start of a photoreaction, each reaction mixture was degassed by being sparged with argon under ultrasonication for 10 min.

Lehrstuhl OC 1 - TUM

200 nm 250 nm 300 nm 350 nm 400 nm 450 nm 500 nm 550 nm 600 nm 650 nm

Datasheet pLED003 367

Basic Information

Type	High-Power-LED - Single Color LuxiGen LZ1 MCPCB
Description	LZ1-10UV0R-0000
Manufacturer / Supplier	ams OSRAM / Mouser
Order number / Date of purch.	n/A / 2021
Internal lot / serial number	2021 / pLED003

Specification Manufacturer

Type / size	SMD, 4.4 mm width
Mechanical specification	3.2 mm lens
Electrical specification	700 mA, 3.8 V
Wavelength (range, typ.)	n/a, typ. 365 nm
Spectral width (FWHM)	n/a
Datasheet	mouser.de

Characterization

Description of measurement	Measured with Ocean-optics USB4000 spectrometer using a calibrated setup (cosine corrector/fibre). The distance between the emitting surface and the surface of the cosine corrector was 20 mm. The LED was operated at 700 mA on a passive heat-sink at approx. 20 °C	
Measured dominant wavelength / Int.	367 nm	12032 µW/mm²nm
Measured spectral width (FWHM)	11 nm	
Integral Reference intensity / range	163787 µW/cm²	350-420 nm

Spectrum

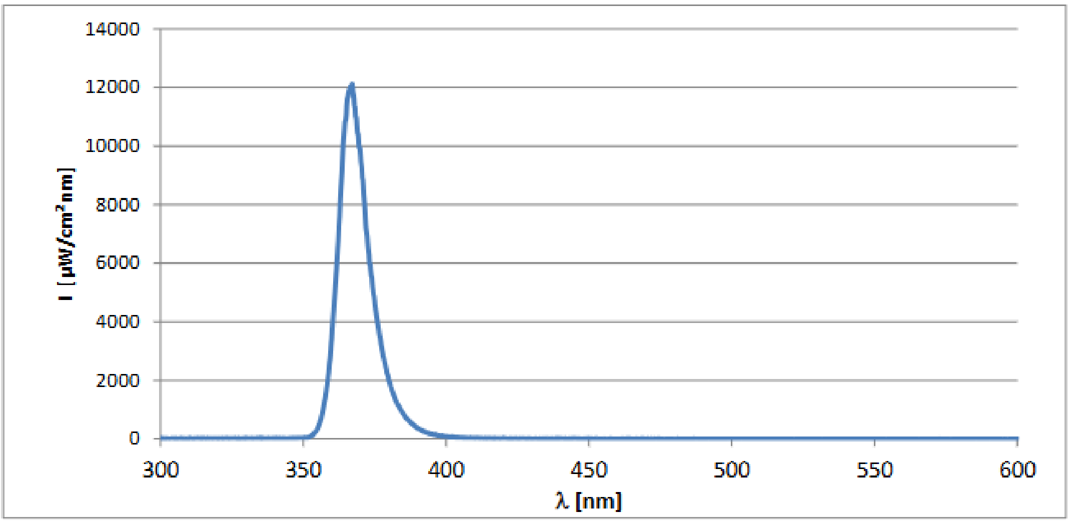

Figure S1: Emission spectrum of the 366 nm LED Parallel Reactor.

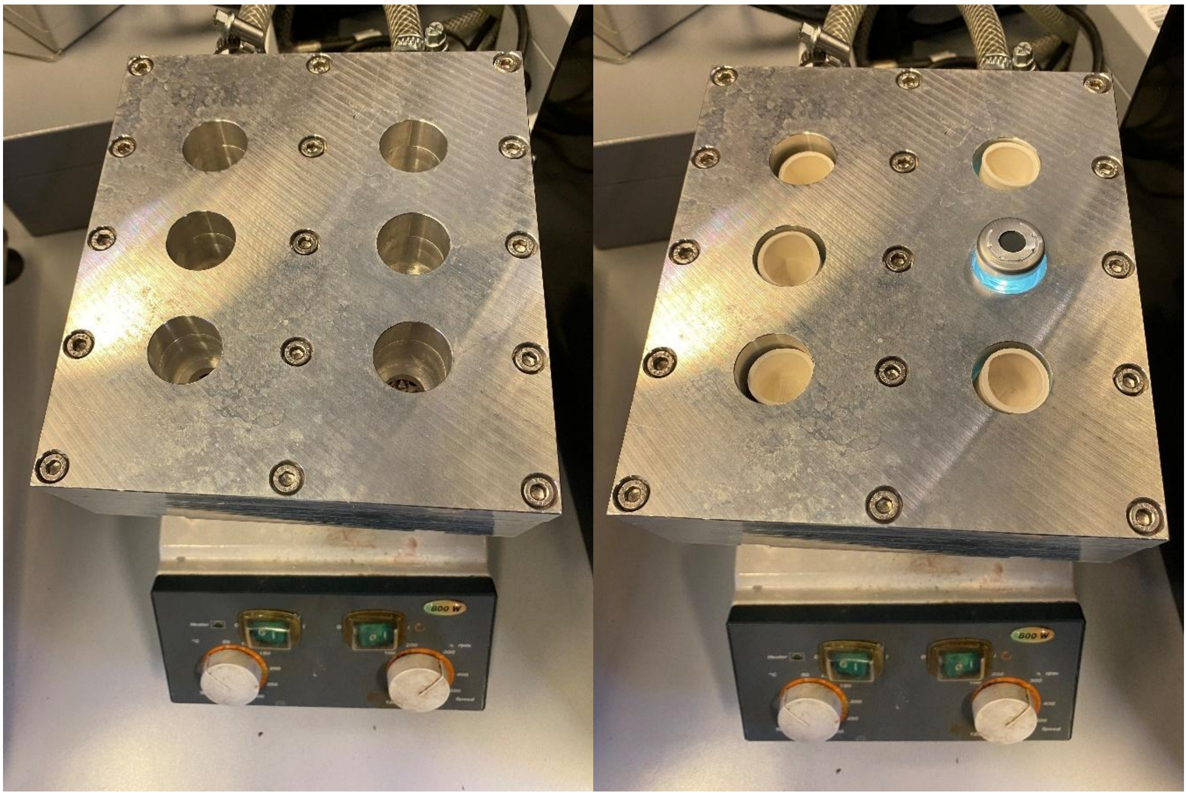

Figure S2: Irradiation setup with six slots from above (left) and enabled (right).

2. Analytical Methods

Thin layer chromatography (TLC) was performed on silica coated glass plates (silica gel 60 F_{254}) with detection by UV-light (λ = 254 nm) and potassium permanganate stain [$KMnO_4$].

Infrared spectra (IR) were recorded on a JASCO IR-4100 or a *Perkin Elmer* Frontier IR-FTR spectrometer by ATR technique. The signal intensity is assigned using the following abbreviations: s (strong), m (medium), w (weak). The following abbreviations were used: aliph = aliphatic, arom = aromatic.

Melting points (M.p.) were determined using a Kofler ("Thermopan", Fs *Reichert*, Wien) apparatus. In case of degradation/polymerization of NCAs upon heating, a melting point of > 260 °C is reported.

Nuclear magnetic resonance (NMR) (^{1}H, ^{13}C and ^{19}F-NMR) spectra were recorded at room temperature (r.t.) on either a *Bruker* AVHD-400, AVHD-500, or a *Bruker* AV-II-500 equipped with a cryo probe head. Chemical shifts of the NMR spectra are reported relative to $CHCl_3$ (^{1}H-NMR: δ = 7.26 ppm, ^{13}C-NMR: δ = 77.16 ppm) or DMSO (^{1}H-NMR: δ = 2.50 ppm, ^{13}C-NMR: δ = 128.06 ppm). The data are reported as follows: chemical shift (δ) [multiplicity, coupling constant *J* (Hz), relative integral, number of protons] where multiplicity is defined as: m = multiplet, s = singlet, d = doublet, t = triplet, non = nonet, br = broad. Apparent multiplets which occur because of coupling constant equality between magnetically non-equivalent protons are marked as virtual (*virt.*).

Mass spectrometry (MS) and **high-resolution mass spectrometry (HRMS)** were measured on a *Thermo Scientific* LTQ-FT Ultra (ESI).

GLC-FID GLC-Analysis performed on an Agilent 7890B Series gas chromatograph using a HP5 column (poly-dimethyl/diphenyl-siloxane, 95/5) with a flame ionization detector. Standard method used for analysis of the compounds *ent*-**1a**, *ent*-**1a**-d_1, *ent*-**1a**- d_2: 60 °C → 260 °C, 15 °C/min; 260 °C → 300 °C, 4 °C/min.

Specific Rotation was determined using an ADP440+ polarimeter (Fa *Bellingham+Stanley*) and is reported as follows: $[\alpha]_D^T$ (c in g per 100 mL solvent).

High Performance Liquid Chromatography (HPLC) was performed using a chiral stationary phase [Chiralpak IC (250 × 4.6 mm), Chiralpak IA (250 × 4.6 mm), Chiralpak OD-RH (150 × 4.6 mm), Chiralpak AS-RH (150 × 4.6 mm), Chiralpak OJ-RH (150 × 4.6 mm), *Daicel Chemical Industries*] with UVD 340 Photodiode Array Detector, P580 Pump and an ASI-100 Automated Sample Injector at 20 °C. For normal-phase HPLC a *Daicel* ChiralPak IA and IC was used as stationary phase, and a mixture of *n*-heptane/*i*-propanol was used as mobile

phase. For reverse-phase HPLC a Daicel ChiralPak AS-RH, OJ-RH, OD-RH was used as stationary phase and a mixture of acetonitrile/water as mobile phase.

3. General Procedures

General Procedure A (GP A):

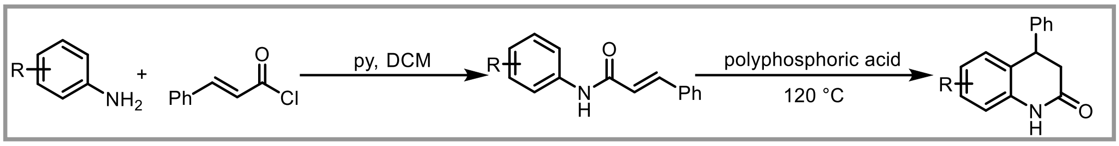

Following a modified procedure[1], to a solution of the aromatic amine (10 mmol) in dichloromethane (DCM, 40 mL), cinnamoyl chloride (10.5 mmol) was added. The resulting suspension was stirred for 30 minutes, after which pyridine (10.5 mmol) was added, and the reaction mixture was stirred at room temperature for 12 hours. Upon completion, the mixture was diluted with DCM (50 mL), washed successively with saturated aqueous ammonium chloride (50 mL) and water (2 × 50 mL), dried over anhydrous Na_2SO_4, and concentrated under reduced pressure to afford a crude light brown solid. The crude product was recrystallized from a DCM/*n*-pentane mixture to afford the corresponding acrylamide derivative.

Polyphosphoric acid (20 g) was heated to 120 °C, and the acrylamide derivative was added. The mixture was stirred for 10 minutes, then cooled to ambient temperature. Crushed ice was added, and the resulting slurry was stirred rapidly for 20 minutes. The mixture was extracted with dichloromethane (DCM, 3 × 50 mL), and the combined organic layers were washed with water (2 × 100 mL), dried over anhydrous Na_2SO_4, and concentrated under reduced pressure. The crude residue was purified by flash chromatography on silica gel to afford the desired product.

General Procedure B (GP B):

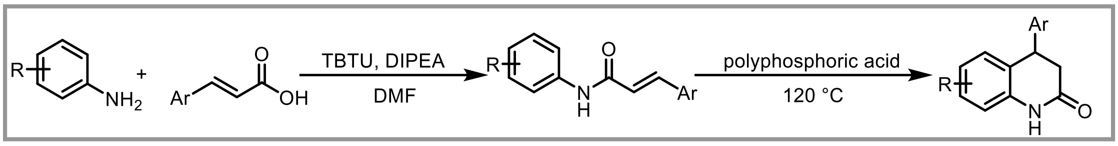

Following a modified procedure[1,2], aromatic amine (5 mmol) was added into a round-bottom flask (50 mL), and 15 mL dimethylformamide (DMF) were added as the solvent. Then, this initial solution was kept under stirring at room temperature, before the cinnamic acid (5.5 mmol) was added. Subsequently, 5.5 mmol 2-(1H-benzotriazol-1-yl)-1,1,3,3-tetramethyluronium hexafluorophosphate (TBTU) was added to the solution, which remained under stirring for 15 min. Thereafter, 17.5 mmol *N*,*N'*-diisopropylethylamine (DIPEA) was added, and the mixture was kept under stirring at room temperature for 48 h. Both the reaction progress and completion were monitored by TLC. Upon completion, 20 mL of saturated $NaHCO_3$ solution was added to the crude mixture, resulting in a precipitated powder after 15 min stirring at room temperature. The precipitate was filtered off and washed successively with saturated $NaHCO_3$ solution (3 × 10 mL) and cold water (3 × 25 mL). The residue was recrystallized from DCM/*n*-pentane to give the corresponding acrylamide derivative. The product was subjected to cyclization as described in GP A.

General Procedure C (GP C):

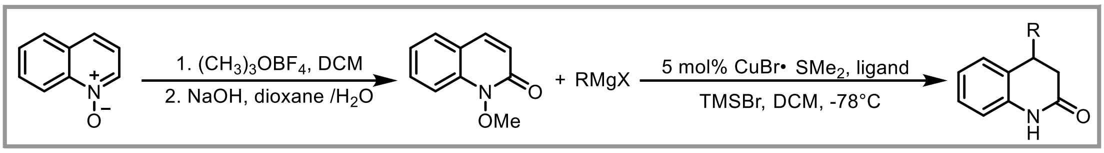

Following a modified procedure[3], a mixture of trimethyloxonium tetrafluoroborate (5.5 mmol, 1.1 equiv.) and quinoline *N*-oxide (5.0 mmol, 1 equiv.) was stirred in dichloromethane (20 mL) for 12 hours. The reaction mixture was then concentrated under reduced pressure, and the crude product was recrystallized from ethyl acetate (EtOAc) to yield the *N*-methoxyquinoline-1-ium tetrafluoroborate salt. A glass reaction flask equipped with a magnetic stirring bar was charged with *N*-methoxyquinoline-1-ium tetrafluoroborate salt (4 mmol), sodium hydroxide (6 mmol, 1.5 equiv.), and a 1:1 mixture of dioxane and water (32 mL). The reaction mixture was stirred at room temperature under an air atmosphere for 48 hours. Reaction progress was monitored by thin-layer chromatography (TLC). Upon completion, the mixture was concentrated under reduced pressure and extracted with dichloromethane (3 × 30 mL). The combined organic layers were washed with brine (50 mL), dried over anhydrous Na_2SO_4, and concentrated under reduced pressure. The crude residue was purified by silica gel flash chromatography to afford the desired product *N*-OMe-2-quinolone.

In a flame-dried Schlenk tube equipped with a septum and magnetic stirring bar, CuBr·SMe_2 (5 mol%), the ligand racemic 2,2′-bis(diphenylphosphino)-1,1′-binaphthyl (BINAP) or (-)-1,2-bis((*2R*,*5R*)-2,5-diphenylphospholano)ethane (*R*,*R*)-Ph-BPE) (6 mol%), and *N*-OMe-2-quinolone (0.2 mmol, 1.0 equiv.) were dissolved in DCM (2 mL) and stirred under nitrogen atmosphere for 20 minutes. The solution was then cooled to −78 °C, and TMSBr (2.0 equiv.) was added dropwise. The reaction mixture was stirred at this temperature for 20 minutes, after which RMgBr (2.0 equiv.) was added dropwise within 5 minutes. The mixture was stirred for 12 hours at −78 °C, then quenched with saturated aqueous ammonium chloride solution (5 mL). The reaction mixture was extracted with DCM (3 × 10 mL), and the combined organic phases were dried over Na_2SO_4 and filtered. The solvent was evaporated under reduced pressure, and the residue was purified by silica gel flash chromatography to yield the desired product.

General Procedure D (GP D):

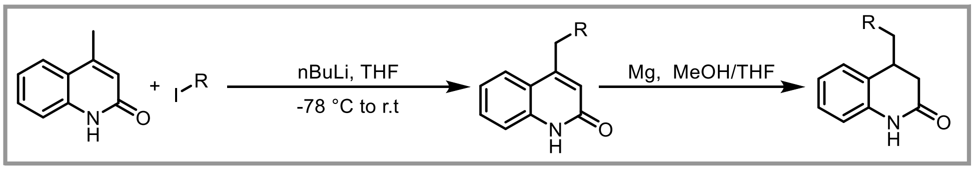

Following a modified procedure[4,5], to a stirred suspension of 4-methylquinolin-2(1H)-one (4 mmol) in dry tetrahydrofuran (20 mL) under a nitrogen atmosphere at −78 °C, a solution of *n*-BuLi (8.8 mmol, 2.2 equiv., 2.5 M in hexanes) was added slowly. Upon completion of the addition, the reaction mixture was allowed to warm to room temperature and stirred for 2 hours. The mixture was then treated with the respective alkyl halide (10 mmol, 2.5 equiv.) at 0 °C and stirred at room temperature for an additional 30 minutes. The reaction mixture was cooled again to 0 °C, treated with 8 mL of 2 N hydrochloric acid (HCl), and extracted with dichloromethane (3 × 30 mL). The organic layer was separated and washed sequentially with saturated Na_2CO_3 solution (10 mL) and saturated sodium chloride solution (50 mL). The organic phase was dried over anhydrous Na_2SO_4 and concentrated under reduced pressure. The residue was further purified by silica gel flash chromatography to yield the desired product.

For the next step, quinolinone derivatives (1.0 mmol) and magnesium (20.0 mmol) were dissolved in a mixture of methanol (15 mL) and THF (5 mL) and stirred at room temperature for 12 hours under an argon atmosphere. Upon completion, the reaction mixture was concentrated under reduced pressure and diluted with saturated NH_4Cl solution (50 mL). The aqueous phase was extracted with dichloromethane (2 × 50 mL). The organic phase was dried over anhydrous Na_2SO_4, concentrated under reduced pressure, and the crude product was purified by silica gel column chromatography to afford the desired product.

General Procedure E (GP E): Kinetic resolution of heterocyclic lactams by a photocatalytic cobalt-catalyzed dehydrogenation.

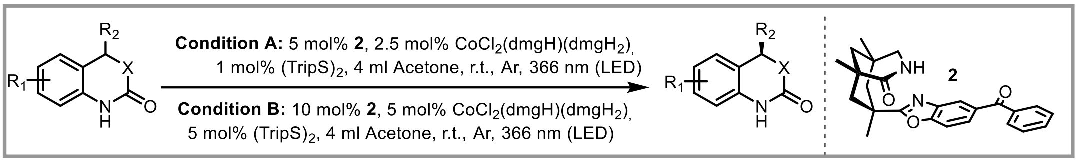

The racemic 4-substituted 3,4-dihydroquinolin-2(1*H*)-one (0.04 mmol, c = 10 mM, 1.00 equiv.) was combined with enantiomerically pure (−)-benzophenone **2** (5.0 mol% for condition A or 10.0 mol% for condition B), $CoCl_2(dmgH)(dmgH_2)$ (2.5 mol% for condition A or 5.0 mol% for condition B), and $(TripS)_2$ (1 mol% for condition A or 5.0 mol% for condition B). The components were dissolved in dry acetone (4 mL) in a dried photo-vial, and the solution was degassed by bubbling argon through the mixture for 10 minutes after ultrasonication. The solution was then irradiated at λ = 366 nm.

After irradiation, the solvent was removed under reduced pressure, and the crude product was subjected to flash column chromatography to yield the enantiomerically enriched products. The enantiomeric excess (*ee*) and specific rotation of the purified enantioenriched/enantiopure products were determined.

4. Condition Optimization

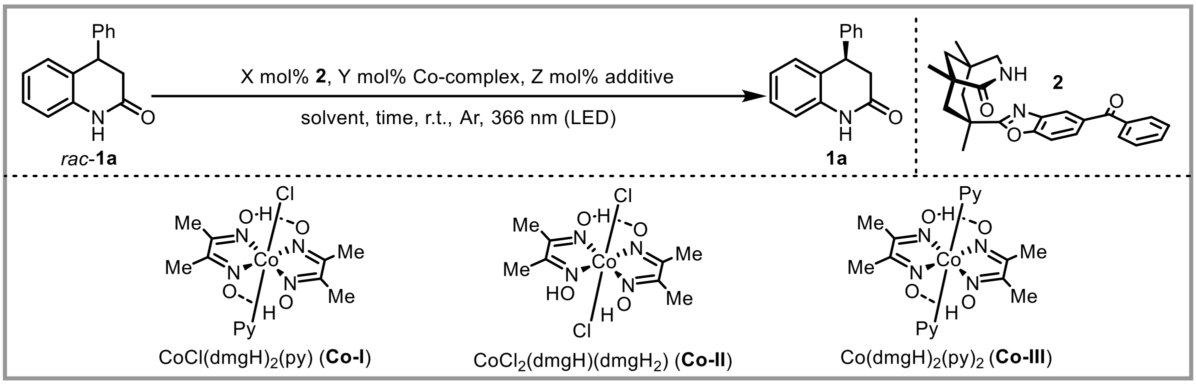

Table S1: Optimization of reaction conditions for the kinetic resolution of *rac*-**1a**.

Entry	**2a**	Co-complex	additive	solvent	t [h]	Yield [%]	*ee* [%]
1	10 mol%	10 mol% **Co-I**	-	8 ml MeCN	4	53	66
2	10 mol%	5 mol% **Co-I**	-	8 ml MeCN	4	48	83
3	10 mol%	1 mol% **Co-I**	-	8 ml MeCN	4	42	95
4	10 mol%	5 mol% **Co-I**	10 mol% TripSH	4 ml MeCN	6	47	88
5	10 mol%	5 mol% **Co-II**	10 mol% TripSH	4 ml MeCN	6	47	94
6	10 mol%	5 mol% **Co-III**	10 mol% TripSH	8 ml $PhCF_3$	5	45	95
7	10 mol%	5 mol% **Co-II**	10 mol% TripSH	4 ml Acetone	5	46	98
8	10 mol%	5 mol% **Co-II**	10 mol% $(TripS)_2$	4 ml Acetone	3	55	88
9	10 mol%	5 mol% **Co-II**	5 mol% $(TripS)_2$	4 ml Acetone	3	58	92
10	5 mol%	2.5 mol% **Co-II**	2.5 mol% $(TripS)_2$	4 ml Acetone	3	50	94
11	5 mol%	2.5 mol% **Co-II**	1 mol% $(TripS)_2$	4 ml Acetone	5	50	97
12	10 mol%	-	10 mol% $(TripS)_2$	4 ml MeCN	16	71	40
13	10 mol%	-	10 mol% $(TripS)_2$	8 ml $PhCF_3$	16	24	80
14	10 mol%	-	-	8 ml $PhCF_3$	16	54	32

All reactions were performed on a 0.04 mmol scale. Yields refer to NMR yields using 1,4-dicyanobenzene as an internal standard. The enantiomeric excess (*ee*) was calculated from the ratio of enantiomers (**1a**/*ent*-**1a**) as determined by chiral HPLC analysis

5. Mechanistic Studies

Conformation of the formation of H_2

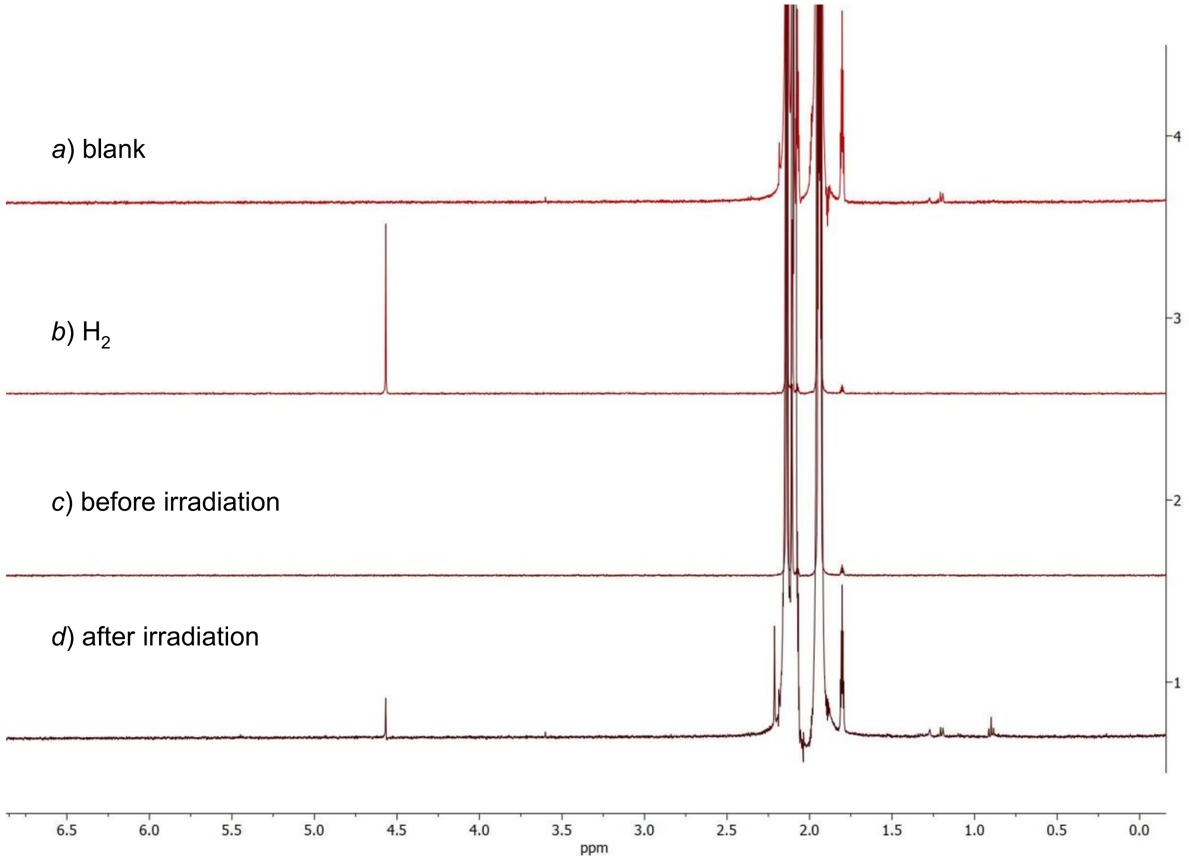

Figure S3. ^{1}H NMR spectrum of blank (*a*), H_2 (*b*, δ [ppm] = 4.57)[6], before irradiation (*c*) and after irradiation (*d*) in acetonitrile-d_3. Before or after irradiation of the standard reaction, the reaction gas was injected into J. Young NMR tube and the NMR was recorded on AVHD500 Bruker NMR.

KIE experiments

Experimental procedure for the determination of the KIE: A dried photo-vial was charged with 4-phenyl-3,4-dihydroquinolin-2(1*H*)-one *ent*-**1a**, *ent*-**1a-*d*₁** or *ent*-**1a-*d*₂** (0.04 mmol, c = 10 mM, 1.00 equiv.), enantiomerically pure (−)-benzophenone **2** (5.0 mol%), $CoCl_2$(dmgH)($dmgH_2$) (2.5 mol%), $(TripS)_2$ (1 mol%), and trimethyl 1,3,5-benzenetricarboxylate (internal standard, 0.25 equiv.). All components were dissolved in 4 mL of dry acetone. The mixture was sonicated at room temperature for 30 minutes to ensure complete dissolution of the substrate, then degassed by bubbling argon through the solution for 10 minutes.

The first aliquot was taken before the reaction mixture was irradiated with parallel LEDs (366 nm) at room temperature. At various time intervals, the light source was briefly turned off, and aliquots (50 µL) were withdrawn from the reaction suspension. Each aliquot was diluted to 0.6 mL with methanol (MeOH) and filtered into a GLC interlocked vial. All samples were subsequently analyzed by gas-liquid chromatography (GLC).

Table S2. The concentration of *ent*-**1a** at different irradiation times.

Entry	Time [s]	[*ent*-**1a**, 1st run] [mM]	[*ent*-**1a**, 2nd run] [mM]	[*ent*-**1a**, 3rd run] [mM]
1	0	10.079	10.179	10.006
2	70	9.226	9.212	9.164
3	100	8.993	9.060	8.964
4	130	8.854	8.796	8.837
5	160	8.744	8.614	8.629
6	190	8.349	8.435	8.1721

Table S3. The concentration of *ent*-**1a-d_1** at different irradiation times.

Entry	Time [s]	[*ent*-**1a-d_1**, 1st run] [mM]	[*ent*-**1a-d_1**, 2nd run] [mM]	[*ent*-**1a-d_1**, 3rd run] [mM]
1	0	10.124	10.098	10.189
2	70	9.794	9.805	9.831
3	110	9.596	9.647	9.566
4	150	9.380	9.376	9.350
5	190	9.088	9.450	9.230
6	230	8.961	9.139	9.147
7	270	8.849	8.907	8.964
8	310	8.750	8.837	8.901
9	350	8.643	8.775	8.745

Table S4. The concentration of *ent*-**1a**-$\boldsymbol{d_2}$ at different irradiation times.

Entry	Time [s]	[*ent*-**1a**-$\boldsymbol{d_2}$, 1st run] [mM]	[*ent*-**1a**-$\boldsymbol{d_2}$, 2nd run] [mM]	[*ent*-**1a**-$\boldsymbol{d_2}$, 3rd run] [mM]
1	0	9.806	9.73	9.652
2	70	9.083	9.283	9.223
3	110	9.010	9.143	9.131
4	150	8.812	8.953	8.941
5	190	8.594	8.838	8.816
6	230	8.415	8.526	8.492

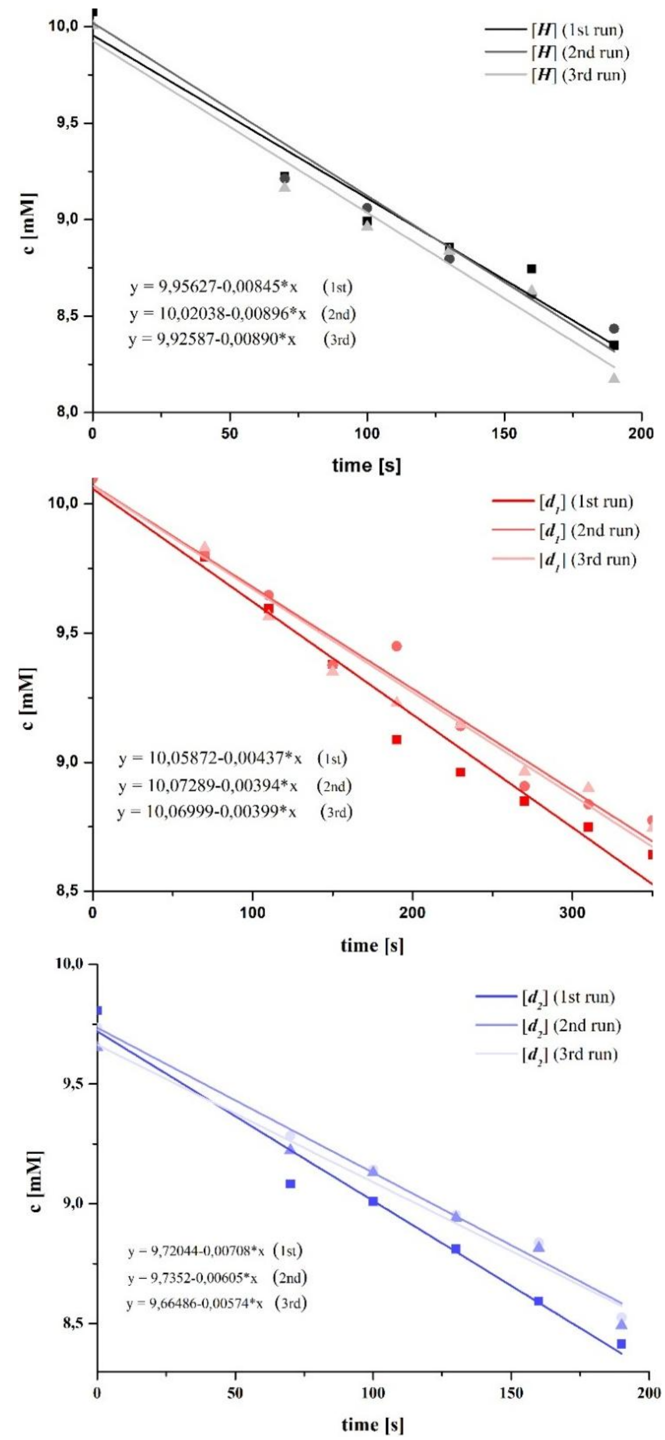

Figure S4. Data plots for the conversion of *ent*-**1a** (*top*), *ent*-**1a-*d₁*** (*middle*) and *ent*-**1a-*d₂*** (*bottom*) over time and the corresponding linear fit.

$$KIE = \frac{k_H}{k_D} = \frac{\frac{\mathrm{d[H]}}{\mathrm{dt}}}{\frac{\mathrm{d[D]}}{\mathrm{dt}}} \qquad \text{(at time } t = 0\text{)}$$

$$KIE(d_1) = \frac{k_H(average)}{k_{d_1}(average)} = \frac{\frac{k_H(1st) + k_H(2nd) + k_H(3rd)}{3}}{\frac{k_{d_1}(1st) + k_{d_1}(2nd) + k_{d_1}(3rd)}{3}}$$

$$= \frac{\frac{-0.00845 - 0.00896 - 0.00890}{3}}{\frac{-0.00437 - 0.00394 - 0.00399}{3}} = 2.1 \pm 0.2$$

(with the standard error of the mean $\sigma_{\overline{KIE(d_1)}}$ = 0.081 and a 95% confidence interval of ±0.2)

$$KIE(d_2) = \frac{k_H(average)}{k_{d_2}(average)} = \frac{\frac{k_H(1st) + k_H(2nd) + k_H(3rd)}{3}}{\frac{k_{d_2}(1st) + k_{d_2}(2nd) + k_{d_2}(3rd)}{3}}$$

$$= \frac{\frac{-0.00845 - 0.00896 - 0.00890}{3}}{\frac{-0.00708 - 0.00605 - 0.00574}{3}} = 1.4 \pm 0.2$$

(with the standard error of the mean $\sigma_{\overline{KIE(d_2)}}$ = 0.093 and a 95% confidence interval of ±0.2)

The standard error was calculated based on the law of error propagation using the following formula:

$$\sigma_{\overline{KIE}} = \sqrt{\frac{1}{k(average)_D^2} \times \sigma_{\overline{k_H}}^2 + \frac{k(average)_H^2}{k(average)_D^4} \times \sigma_{\overline{k_D}}^2}$$

with $\sigma_{\overline{k}} = \frac{1}{\sqrt{N}} \times \sqrt{\frac{1}{N-1} \times \sum_{i=1}^{N} (k(i) - k(average))^2}$ and N: number of experiments.

Additional control experiments

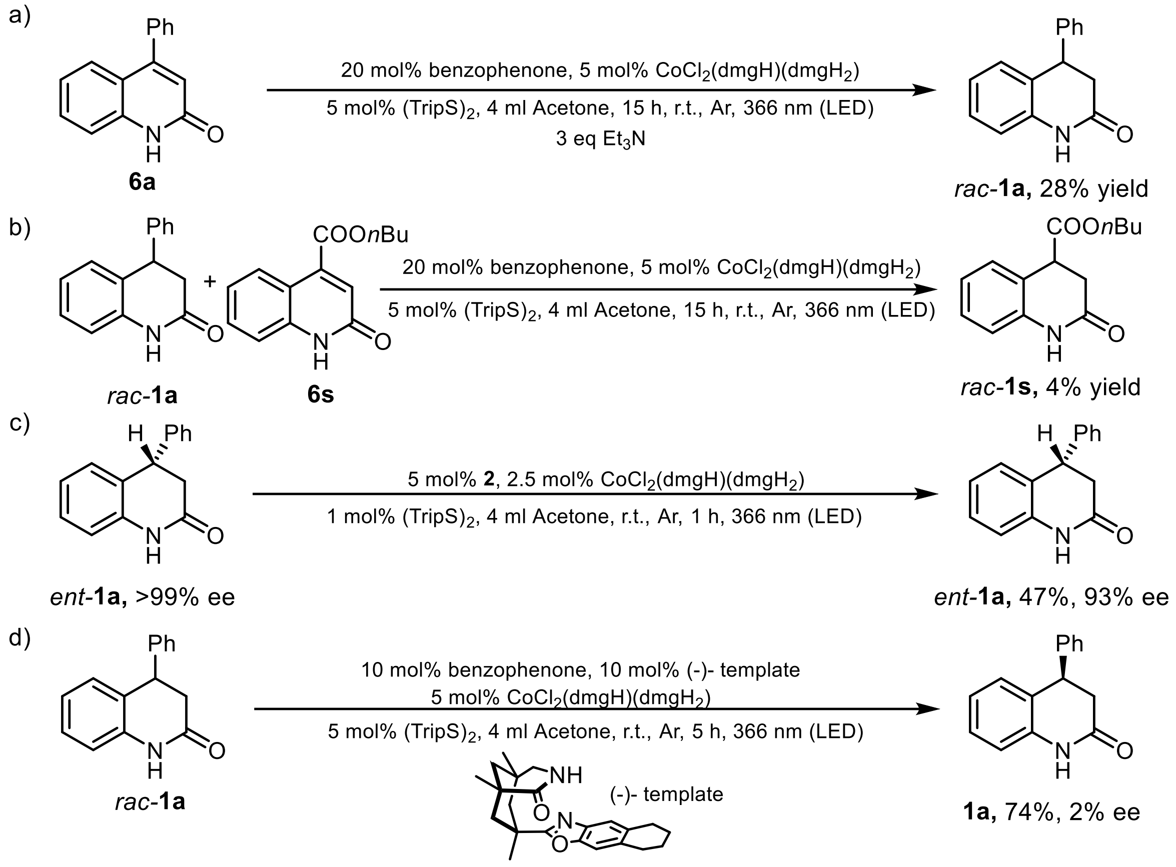

Figure S5. The control experiments.

Note: For control experiment a) and b), these two reactions indicate that the oxidation product is reduced by the cobalt catalyst regenerating some racemic starting substrates. For control experiment c), the decrease of *ee* of *ent*-**1a** also demonstrates the regeneration of racemic starting substrate. For control experiment d), the reaction shows that the kinetic resolution is only successful if the benzophenone is covalently linked to the azabicyclo[3.3.0]nonan-2-one backbone.

6. Substrate Synthesis

4-Phenyl-3,4-dihydroquinolin-2(1*H*)-one (*rac*-1a)

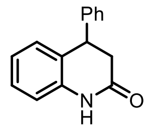

rac-**1a**
$C_{15}H_{13}NO$
M = 223.28 g mol^{-1}

According to GP A, compound *rac*-**1a** was synthesized from aniline (10 mmol) and cinnamoyl chloride (10.5 mmol) as white solid (1.05g, 4.7 mmol, 47%).

TLC (Hex:EtOAc = 2:1): R_f = 0.38 [UV] [KMnO4].

^{1}H-NMR (500 MHz, CDCl3, 300 K): δ [ppm]= 8.10 (s, 1H), 7.36 – 7.32 (m, 2H), 7.29 – 7.26 (m, 1H), 7.23 – 7.18 (m, 3H), 6.99 – 6.91 (m, 2H), 6.83 (dd, ^3J = 7.8 Hz, ^4J = 1.2 Hz, 1H), 4.30 (*virt.* t, $^3J \approx {}^3J$ = 7.5 Hz, 1H), 2.99 – 2.89 (m, 2H).

^{13}C-NMR (101 MHz, CDCl3, 300 K): δ [ppm] = 170.5, 141.5, 137.1, 129.1, 128.6, 128.2, 128.0, 127.4, 126.9, 123.5, 115.6, 42.2, 38.6.

Spectral data matched those reported in the literature.[1]

4-(4-Methoxyphenyl)-3,4-dihydroquinolin-2(1*H*)-one (*rac*-1b)

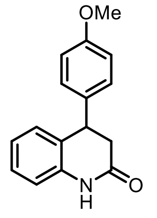

rac-**1b**
$C_{16}H_{15}NO_2$
M = 253.30 g mol^{-1}

According to GP B, compound *rac*-**1b** was synthesized from aniline (5 mmol) and (*E*)-3-(4-methoxyphenyl)acrylic acid (5.5 mmol) as white solid (0.57g, 2.26 mmol, 45%).

TLC (Hex:EtOAc = 2:1): R_f = 0.31 [UV] [KMnO4].

^{1}H-NMR (400 MHz, CDCl3, 300 K): δ [ppm] = 8.57 (s, 1H), 7.20 (*virt.* td, $^3J \approx {}^3J$ = 7.5 Hz, ^4J = 1.8 Hz, 1H), 7.13 – 7.08 (m, 2H), 6.99 – 6.90 (m, 2H), 6.89 – 6.83 (m, 3H), 4.25 (dd, ^3J = 8.4 Hz, ^3J = 6.6 Hz, 1H), 3.79 (s, 3H), 2.95 – 2.85 (m, 2H).

^{13}C-NMR (101 MHz, CDCl3, 300 K): δ [ppm] = 171.0, 158.9, 137.1, 133.5, 129.0, 128.5, 128.1, 127.3, 123.5, 115.8, 114.4, 55.4, 41.4, 38.7.

Spectral data matched those reported in the literature.[7]

4-(*p*-Tolyl)-3,4-dihydroquinolin-2(1*H*)-one (*rac*-1c)

According to GP B, compound *rac*-**1c** was synthesized from aniline (5 mmol) and (*E*)-3-(*p*-tolyl)acrylic acid (5.5 mmol) as white solid (0.51g, 2.15 mmol, 43%).

TLC (Hex:EtOAc = 2:1): R_f = 0.39 [UV] [KMNO4].

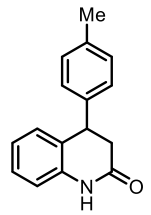

rac-**1c**
$C_{16}H_{15}NO$
M = 237.30 g mol^{-1}

^{1}H-NMR (500 MHz, $CDCl_3$, 300 K): δ [ppm] = 8.78 (s, 1H), 7.20 (*virt.* td, $^{3}J \approx {}^{3}J$ = 7.6 Hz, ^{4}J = 1.7 Hz, 1H), 7.16 – 7.13 (m, 2H), 7.10 – 7.07 (m, 2H), 6.98 – 6.90 (m, 2H), 6.86 (dd, ^{3}J = 7.9 Hz, ^{4}J = 1.2 Hz, 1H), 4.27 (*virt.* t, $^{3}J \approx {}^{3}J$ = 7.5 Hz, 1H), 2.97 – 2.86 (m, 2H), 2.34 (s, 3H).

^{13}C-NMR (101 MHz, $CDCl_3$, 300 K): δ [ppm] = 171.1, 138.5, 137.1, 137.0, 129.7, 128.5, 128.1, 127.8, 127.1, 123.5, 115.8, 41.8, 38.6, 21.2.

Spectral data matched those reported in the literature.[7]

4-(4-Fluorophenyl)-3,4-dihydroquinolin-2(1*H*)-one (*rac*-1d)

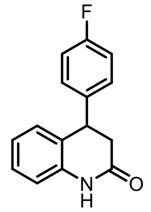

rac-**1d**
$C_{15}H_{12}FNO$
M = 241.27 g mol^{-1}

According to GP B, compound *rac*-**1d** was synthesized from aniline (5 mmol) and (*E*)-3-(4-fluorophenyl)acrylic acid (5.5 mmol) as white solid (0.49g, 2.04 mmol, 41%).

TLC (Hex:EtOAc = 2:1): R_f = 0.37 [UV] [$KMnO_4$].

^{1}H-NMR (400 MHz, $CDCl_3$, 300 K): δ [ppm] = 8.33 (s, 1H), 7.22 (*virt.* td, $^{3}J \approx {}^{3}J$ = 7.6 Hz, ^{4}J = 1.6 Hz, 1H), 7.18 – 7.12 (m, 2H), 7.05 – 6.96 (m, 3H), 6.94 – 6.90 (m, 1H), 6.85 (dd, ^{3}J = 7.9 Hz, ^{4}J = 1.2 Hz, 1H), 4.29 (dd, ^{3}J = 8.3 Hz, ^{3}J =6.3 Hz, 1H), 2.97 – 2.84 (m, 2H).

^{13}C-NMR (101 MHz, $CDCl_3$, 300 K)**:** δ [ppm] = 170.8, 162.1 (d, J = 245.9 Hz), 137.4 (d, J = 3.2 Hz), 137.2, 129.5 129.4, 128.4 (d, J = 9.2 Hz), 126.6, 123.6, 116.0, 115.9 (d, J = 19.2 Hz), 41.5, 38.7.

^{19}F-NMR (376 MHz, $CDCl_3$, 300 K)**:** δ [ppm] = −115.4.

Spectral data matched those reported in the literature.[7]

4-(4-Chlorophenyl)-3,4-dihydroquinolin-2(1*H*)-one (*rac*-1e)

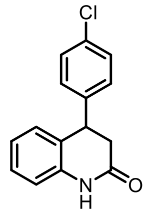

rac-**1e**
$C_{15}H_{12}ClNO$
M = 257.72 g mol^{-1}

According to GP B, compound *rac*-**1e** was synthesized from aniline (5 mmol) and (*E*)-3-(4-chlorophenyl)acrylic acid (5.5 mmol) as white solid (0.27g, 1.05 mmol, 21%).

TLC (Hex:EtOAc = 2:1): R_f = 0.37 [UV] [$KMnO_4$].

^{1}H-NMR (500 MHz, $CDCl_3$, 300 K): δ [ppm] = 8.41 (s, 1H), 7.32 – 7.28 (m, 2H), 7.22 (*virt.* td, $^{3}J \approx {}^{3}J$ = 7.7 Hz, ^{4}J = 1.5 Hz, 1H), 7.15 – 7.10 (m, 2H),

6.98 (*virt.* td, $^3J \approx {}^3J$ = 7.5 Hz, ^4J = 1.2 Hz, 1H), 6.93 – 6.90 (m, 1H), 6.85 (dd, ^3J = 8.0 Hz, ^4J = 1.1 Hz, 1H), 4.28 (dd, ^3J = 8.4 Hz, ^3J = 6.2 Hz, 1H), 2.97 – 2.84 (m, 2H).

^{13}C-NMR (101 MHz, $CDCl_3$, 300 K): δ [ppm] = 170.3, 140.1, 137.1, 133.2, 129.3, 129.2, 128.5, 128.4, 126.3, 123.7, 115.9, 41.6, 38.5.

Spectral data matched those reported in the literature.[7]

4-(4-Bromophenyl)-3,4-dihydroquinolin-2(1*H*)-one (*rac*-1f)

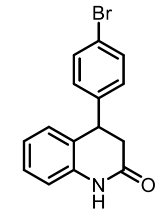

rac-**1f**
$C_{15}H_{12}BrNO$
M = 302.17 g mol^{-1}

According to GP B, compound *rac*-**1f** was synthesized from aniline (5 mmol) and (*E*)-3-(4-bromophenyl)acrylic acid (5.5 mmol) as white solid (0.45g, 1.49 mmol, 30%).

TLC (Hex:EtOAc = 2:1): R_f = 0.36 [UV] [$KMnO_4$].

^{1}H-NMR (400 MHz, $CDCl_3$, 300 K): δ [ppm] = 8.51 (s, 1H), 7.47 – 7.43 (m, 2H), 7.22 (*virt.* td, $^3J \approx {}^3J$ = 7.6 Hz, ^4J = 1.6 Hz, 1H), 7.09 – 7.05 (m, 2H), 6.98 (*virt.* td, $^3J \approx {}^3J$ = 7.5 Hz, ^4J = 1.2 Hz, 1H), 6.93 – 6.90 (m, 1H), 6.86 (dd, ^3J = 7.8 Hz, ^4J = 1.2 Hz, 1H), 4.27 (dd, ^3J = 8.3 Hz, ^3J = 6.3 Hz, 1H), 2.97 – 2.83 (m, 2H).

^{13}C-NMR (101 MHz, $CDCl_3$, 300 K): δ [ppm] = 170.3, 140.6, 137.1, 132.2, 129.7, 128.5, 128.4, 126.2, 123.7, 121.3, 115.9, 41.7, 38.4.

Spectral data matched those reported in the literature.[8]

4-(3-Chlorophenyl)-3,4-dihydroquinolin-2(1*H*)-one (*rac*-1g)

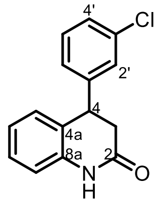

rac-**1g**
$C_{15}H_{12}ClNO$
M = 257.72 g mol^{-1}

According to GP B, compound *rac*-**1g** was synthesized from aniline (5 mmol) and (*E*)-3-(3-chlorophenyl)acrylic acid (5.5 mmol) as white solid (0.23g, 0.89 mmol, 18%).

TLC (Hex:EtOAc = 2:1): R_f = 0.36 [UV] [$KMnO_4$].

M.p.: 133 °C.

^{1}H-NMR (500 MHz, $CDCl_3$, 300 K): δ [ppm] = 7.87 (s, NH), 7.29 – 7.21 (m, 3H, H5, H7, H5'), 7.16 (*virt.* t, $^4J \approx {}^4J$ = 1.8 Hz, 1H, H2'), 7.08 (*virt.* dt, $^3J \approx {}^3J$ = 6.8 Hz, ^4J = 2.0 Hz, 1H, H4'), 7.00 (*virt.* td, $^3J \approx {}^3J$ = 7.4 Hz, ^4J = 1.2 Hz, H6), 6.94 (d, ^3J = 7.5 Hz, H6'), 6.82 (dd, ^3J = 7.9 Hz, ^4J = 1.2 Hz, H8), 4.28 (dd, ^3J = 8.2 Hz, ^3J = 6.3 Hz, H4), 2.99 – 2.84 (m, 2H, H3).

^{13}C-NMR (101 MHz, $CDCl_3$, 300 K)**:** *δ* [ppm] = 170.1 (C2), 143.7 (C1'), 137.1 (C8a), 134.9 (C3'), 130.4 (C5'), 128.6 (C6'), 128.5 (C5), 128.1(C2'), 127.7 (C7), 126.1(C4'), 125.9 (C4a), 123.7 (C6), 115.9 (C8), 41.9 (C4), 38.4 (C3).

HRMS (ESI) *m*/*z*: calculated for $[M(^{35}Cl)+H]^+$: 258.0680; found:258.0679.

IR (film): $\tilde{\nu}_{max}/cm^{-1}$ = 3195 (m, NH), 3057 (m, CH_{arom}), 2982 (m, CH_{aliph}), 2895(m, CH_{aliph}), 1671 (s, C=O), 1593 (m, $C{=}C_{arom}$), 1487(m, $C{=}C_{arom}$), 1383 (m, C-N), 759 (m, CH_{arom}).

6-Fluoro-4-phenyl-3,4-dihydroquinolin-2(1*H*)-one (*rac*-1h)

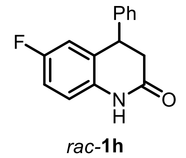

rac-**1h**
$C_{15}H_{12}FNO$
M = 241.27 g mol^{-1}

According to GP A, compound *rac*-**1h** was synthesized from 4-fluoroaniline (5 mmol) and cinnamoyl chloride (5.25 mmol) as white solid (0.54g, 2.25 mmol, 45%).

TLC (Hex:EtOAc = 2:1): R_f = 0.36 [UV] [$KMnO_4$].

^{1}H-NMR (500 MHz, $CDCl_3$, 300 K): *δ* [ppm] = 9.57 (s, 1H), 7.40 – 7.27 (m, 3H), 7.20 (dd, ^3J = 7.0 Hz, ^4J = 1.9 Hz, 2H), 6.93 – 6.85 (m, 2H), 6.61 (dd, ^3J = 9.0 Hz, ^4J = 2.5 Hz, 1H), 4.27 (*virt.* t, $^3J \approx {}^3J$ = 7.8 Hz, 1H), 2.96 – 2.87 (m, 2H).

^{13}C-NMR (101 MHz, $CDCl_3$, 300 K)**:** *δ* [ppm] = 171.1, 158.9 (d, *J* = 242.3 Hz), 140.7, 133.3 (d, *J* = 2.6 Hz), 129.1, 128.7 (d, *J* = 7.1 Hz), 127.8, 127.6, 116.9 (d, *J* = 8.1 Hz), 115.3 (d, *J* = 23.7 Hz), 114.6 (d, *J* = 23.0 Hz), 42.1, 38.0.

^{19}F-NMR (376 MHz, $CDCl_3$, 300 K)**:** δ [ppm] = –119.3.

Spectral data matched those reported in the literature.[7]

7-Methyl-4-phenyl-3,4-dihydroquinolin-2(1H)-one (*rac*-1i)

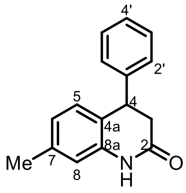

rac-**1i**
$C_{16}H_{15}NO$
M = 237.30 g mol^{-1}

According to GP B, compound *rac*-**1i** was synthesized from 3-methylaniline (5 mmol) and cinnamic acid (5.5 mmol) as white solid (0.29g, 1.23 mmol, 25%).

TLC (Hex:EtOAc = 2:1): R_f = 0.36 [UV] [$KMnO_4$].

M.p.: 182°C.

^{1}H-NMR (500 MHz, $CDCl_3$, 300 K): *δ* [ppm] = 8.50 (s, 1H, NH), 7.36 – 7.30 (m, 2H, H3',H5'), 7.29 – 7.23 (m, 1H, H4'), 7.22 – 7.17 (m, 2H, H2', H6'), 6.84 – 6.75 (m, 2H, H5, H6), 6.67 (d, *J* = 1.6 Hz, 1H, H8), 4.26 (dd, *J* = 8.4, 6.5 Hz, 1H, H4), 2.98 – 2.86 (m, 2H, H3), 2.31 (s, 3H, CH_3).

^{13}**C-NMR** (101 MHz, $CDCl_3$, 300 K): *δ* [ppm] = 170.9 (C2), 141.9 (C1’), 138.2 (C7), 137.0 (C8a), 129.0 (C3’, C5’), 128.4 (C5), 127.9 (C2’, C6’), 127.3 (C4’), 124.3 (C6), 123.9(C4a), 116.3 (C8), 41.9 (C4), 38.8 (C3), 21.2 (CH_3).

HRMS (ESI) *m/z*: calculated for $[M+H]^+$: 238.1218; found:238.1226.

IR (film): $\tilde{\nu}_{max}/cm^{-1}$ = 3194 (m, NH), 3027 (m, CH_{arom}), 2981 (m, CH_{aliph}), 2910 (m, CH_{aliph}), 1676 (s, C=O), 1583 (m, $C{=}C_{arom}$), 1483(m, $C{=}C_{arom}$), 1372 (m, C-N), 701 (m, CH_{arom}).

7-Methoxy-4-phenyl-3,4-dihydroquinolin-2(1H)-one (*rac*-1j)

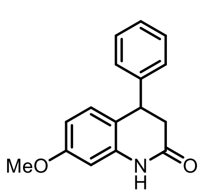

rac-**1j**
$C_{16}H_{15}NO_2$
M = 253.30 g mol^{-1}

According to GP B, compound *rac*-**1j** was synthesized from 3-methoxyaniline (5 mmol) and cinnamic acid (5.5 mmol) as white solid (0.25g, 0.99 mmol, 20%).

TLC (Hex:EtOAc = 2:1): R_f = 0.31 [UV] [$KMnO_4$].

^{1}**H-NMR** (500 MHz, $CDCl_3$, 300 K): *δ* [ppm] = 8.09 (s, 1H), 7.35 – 7.31 (m, 2H), 7.28 – 7.24 (m, 2H), 7.20 – 7.16 (m, 2H), 6.82 (d, *J* = 8.4 Hz, 1H), 6.51 (dd, *J* = 8.4, 2.5 Hz, 1H), 6.38 (d, *J* = 2.5 Hz, 1H), 4.24 (dd, *J* = 8.4, 6.4 Hz, 1H), 3.78 (s, 3H), 3.07 – 2.79 (m, 2H).

^{13}**C-NMR** (101 MHz, $CDCl_3$, 300 K): *δ* [ppm] = 170.6, 159.7, 142.0, 138.1, 129.5, 129.0, 127.9, 127.3, 119.1, 108.6, 101.8, 55.6, 41.6, 38.9.

Spectral data matched those reported in the literature.[9]

7-Chloro-4-phenyl-3,4-dihydroquinolin-2(1*H*)-one (*rac*-1k)

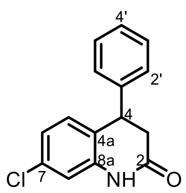

rac-**1k**
$C_{15}H_{12}ClNO$
M = 257.72 g mol^{-1}

According to GP B, compound *rac*-**1k** was synthesized from 3-chloroaniline (5 mmol) and the cinnamic acid (5.5 mmol) as white solid (0.44g, 1.71 mmol, 34%).

TLC (Hex:EtOAc = 2:1): R_f = 0.36 [UV] [$KMnO_4$].

M.p.: 167 °C.

^{1}**H-NMR** (500 MHz, $CDCl_3$, 300 K): *δ* [ppm] = 8.62 (s, 1H, NH), 7.37 – 7.33 (m, 2H, H3’, H5’), 7.31 – 7.27 (m, 1H, H4’), 7.20 – 7.15 (m, 2H, H2’, H6’), 6.93 (dd, ^3J = 8.2 Hz, ^4J = 2.1 Hz, 1H, H5), 6.87 (d, ^4J = 2.1 Hz, 1H, H8), 6.84 (d, ^3J = 8.2 Hz, 1H, H6), 4.27 (dd, ^3J = 8.5 Hz, ^3J = 6.6 Hz, 1H, H4), 2.99 – 2.86 (m, 2H, H3).

^{13}C-NMR (101 MHz, $CDCl_3$, 300 K)**:** δ [ppm] = 170.9 (C2), 141.1 (C1'), 138.3 (C8a), 133.7 (C7), 129.7 (C6), 129.2 (C3', C5'), 127.9 (C2', C6'), 127.6 (C4'), 125.3 (C4a), 123.4 (C5), 115.8 (C8), 41.7 (C4), 38.4 (C3).

HRMS (ESI) *m/z*: calculated for $[M(^{35}Cl)+H]^+$: 258.0680; found:258.0679.

IR (film): $\tilde{\nu}_{max}/cm^{-1}$ = 3124 (m, NH), 3027 (m, CH_{arom}), 2955 (m, CH_{aliph}), 1678 (s, C=O), 1606 (m, $C=C_{arom}$), 1481(m, $C=C_{arom}$), 1366 (m, C-N), 757 (m, CH_{arom}).

4-Methyl-3,4-dihydroquinolin-2(1*H*)-one (*rac*-1l)

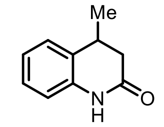

rac-**1l**
$C_{10}H_{11}NO$
M = 161.20 g mol^{-1}

According to GP D, compound *rac*-**1l** was prepared from 4-methylquinolin-2(1H)-one **6l** (2 mmol) as white solid (182mg, 1.13 mmol, 57%).

TLC (Hex:EtOAc = 2:1): R_f = 0.37 [UV] [$KMnO_4$].

^{1}H-NMR (400 MHz, $CDCl_3$, 300 K): δ [ppm] = 8.54 (s, 1H), 7.22 – 7.13 (m, 2H), 7.03 (*virt.* td, $^3J \approx {}^3J$ = 7.5 Hz, ^4J = 1.2 Hz, 1H), 6.81 (dd, ^3J = 7.8 Hz, ^4J = 1.2 Hz, 1H), 3.14 (*virt.* h, $^3J \approx {}^3J \approx {}^3J$ = 6.9 Hz, 1H), 2.74 (dd, ^2J = 16.1 Hz, ^3J = 5.8 Hz, 1H), 2.43 (dd, ^2J = 16.1 Hz, ^3J = 7.2 Hz, 1H), 1.32 (d, ^3J = 7.0 Hz, 3H).

^{13}C-NMR (101 MHz, $CDCl_3$, 300 K): δ [ppm] = 171.4, 136.6, 128.9, 127.7, 126.7, 123.5, 115.7, 38.5, 30.9, 19.9.

Spectral data matched those reported in the literature.[10]

4-Ethyl-3,4-dihydroquinolin-2(1*H*)-one (*rac*-1m)

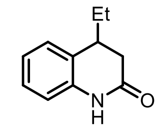

rac-**1m**
$C_{11}H_{13}NO$
M = 175.23 g mol^{-1}

According to GP D, 4-ethylquinolin-2(1H)-one (***6m***) was synthesized from 4-methylquinolin-2(1H)-one (4 mmol) and the iodomethane (10 mmol, 2.5 equiv.) as white solid (421mg, 2.43 mmol, 61%). Then, compound *rac*-**1m** was prepared from quinolone **6m** (1 mmol) as white solid (129mg, 0.74 mmol, 74%).

TLC (Hex:EtOAc = 2:1): R_f = 0.38 [UV] [$KMnO_4$].

^{1}H-NMR (500 MHz, $CDCl_3$, 300 K): δ [ppm] = 8.45 (s, 1H), 7.21 – 7.13 (m, 2H), 7.01 (*virt.* td, $^3J \approx {}^3J$ = 7.5 Hz, ^4J = 1.2 Hz, 1H), 6.80 (dd, ^3J = 7.8 Hz, ^4J = 1.2 Hz, 1H), 2.88 – 2.82 (m, 1H), 2.77 (dd, ^2J = 16.1 Hz, ^3J = 6.2 Hz, 1H), 2.56 (dd, ^2J = 16.2 Hz, ^3J = 4.0 Hz, 1H), 1.70 – 1.54 (m, 2H), 0.94 (t, ^3J = 7.4 Hz, 3H).

^{13}**C-NMR** (101 MHz, $CDCl_3$, 300 K): δ [ppm] = 171.3, 136.5, 128.2, 127.7, 127.6, 123.2, 115.8, 38.0, 36.0, 27.2, 11.5.

Spectral data matched those reported in the literature.[10]

4-Ethylquinolin-2(1*H*)-one (6m)

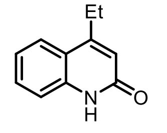

6m
$C_{11}H_{11}NO$
M = 173.22 g mol^{-1}

TLC (Hex:EtOAc = 2:1): R_f = 0.25 [UV] [$KMnO_4$].

^{1}**H-NMR** (500 MHz, $CDCl_3$, 300 K): δ [ppm] = 11.95 (s, 1H), 7.74 (dd, ^3J = 8.3 Hz, ^4J = 1.4 Hz, 1H), 7.51 (ddd, ^3J = 8.4 Hz, ^3J = 7.1 Hz, ^4J = 1.3 Hz, 1H), 7.43 (dd, ^3J = 8.2 Hz, ^4J = 1.3 Hz, 1H), 7.26 – 7.23 (m, 1H), 6.62 (s, 1H), 2.92 (qd, ^3J = 7.4, ^4J = 1.1 Hz, 2H), 1.37 (t, ^3J = 7.5 Hz, 3H).

^{13}**C-NMR** (101 MHz, $CDCl_3$, 300 K): δ [ppm] = 164.5, 154.8, 138.6, 130.5, 124.2, 122.7, 120.1, 118.6, 116.9, 25.3, 13.0.

Spectral data matched those reported in the literature.[11]

(*S*)-4-Ethyl-3,4-dihydroquinolin-2(1*H*)-one (1m)

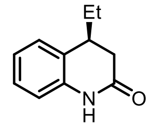

1m
$C_{11}H_{13}NO$
M = 175.23 g mol^{-1}

According to GP C, 1-methoxyquinolin-2(1H)-one was synthesized from quinoline *N*-oxide (13.8 mmol) and trimethyloxonium tetrafluoroborate (15.2 mmol, 1.1 equiv.) as white solid (368mg, 2.10 mmol, 15%). Then, compound **1m** was synthesized from 1-methoxyquinolin-2(1H)-one (0.2 mmol) with CuBr·SMe_2 (0.01 mmol, 5 mol%), and ligand (*R*, *R*)-Ph-BPE (0.012 mmol, 6 mol%) as white solid (27.3mg, 156 µmol, 78% yield, 99% *ee*).[3]

4-Propyl-3,4-dihydroquinolin-2(1*H*)-one (*rac*-1n)

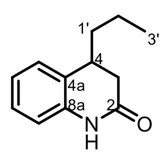

rac-**1n**
$C_{12}H_{15}NO$
M = 189.26 g mol^{-1}

According to GP D, 4-propylquinolin-2(1H)-one (***6n***) was synthesized from 4-methylquinolin-2(1H)-one (***6l***, 4 mmol) and the iodoethane (10 mmol, 2.5 equiv.) as white solid (437mg, 2.31 mmol, 58%). Then, compound *rac*-**1n** was prepared from quinolone **6n** (1 mmol) as white solid (140 mg, 0.74 mmol, 74%).

TLC (Hex:EtOAc = 2:1): R_f = 0.37 [UV] [$KMnO_4$].

M.p.: 83 °C.

^{1}H-NMR (500 MHz, $CDCl_3$, 300 K): *δ* [ppm] = 8.19 (s, 1H, NH), 7.20 – 7.13 (m, 2H, H5, H7), 7.00 (*virt.* td, $^3J \approx {}^3J$ = 7.5 Hz, ^4J = 1.2 Hz, 1H, H6), 6.78 (dd, ^3J = 7.8 Hz, ^4J = 1.2 Hz, 1H, H8), 2.95 (*virt.* qd, $^3J \approx {}^3J$ = 7.0, ^3J = 3.7 Hz, 1H, H4), 2.76 (dd, ^2J = 16.1, ^3J = 6.1 Hz, 1H, H3a), 2.54 (dd, ^2J = 16.1, ^3J = 3.9 Hz, 1H, H3b), 1.64 – 1.48 (m, 2H, H1'), 1.47 – 1.37 (m, 1H, H2a'), 1.34 – 1.25 (m, 1H, H2b'), 0.91 (t, ^3J = 7.3 Hz, 3H, H3').

^{13}C-NMR (101 MHz, $CDCl_3$, 300 K): *δ* [ppm] = 171.4 (C2), 136.6 (C8a), 128.0 (C5), 127.9 (4a), 127.6 (C7), 123.1 (C6), 115.8 (C8), 36.5 (C1'), 36.3 (C3 or C4, undistinguishable), 36.3 (C3 or C4, undistinguishable), 20.1 (C2'), 14.1 (C3').

HRMS (ESI) *m/z*: calculated for $[M+H]^+$: 190.1226; found:190.1225.

IR (film): $\tilde{\nu}_{max}/cm^{-1}$ = 3194 (m, NH), 3061 (m, CH_{arom}), 2964 (m, CH_{aliph}), 2918 (m, CH_{aliph}), 1671 (s, C=O), 1591 (m, $C{=}C_{arom}$), 1490(m, $C{=}C_{arom}$), 1382 (m, C-N), 752 (m, CH_{arom}).

4-Propylquinolin-2(1*H*)-one (6n)

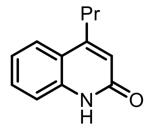

6n

$C_{12}H_{13}NO$

M = 187.24 g mol^{-1}

TLC (Hex:EtOAc = 2:1): R_f = 0.25 [UV] [$KMnO_4$].

^{1}H-NMR (500 MHz, $CDCl_3$, 300 K): *δ* [ppm] = 12.15 (s, 1H), 7.74 (dd, ^3J = 8.1 Hz, ^4J = 1.3 Hz, 1H), 7.51 (ddd, ^3J = 8.3 Hz, ^3J = 7.0 Hz, ^4J = 1.3 Hz, 1H), 7.45 (dd, ^3J = 8.2 Hz, ^4J = 1.3 Hz, 1H), 7.26 – 7.22 (m, 1H), 6.61 (s, 1H), 2.85 (t, ^3J = 7.5 Hz, 2H), 1.78 (*virt.* h, $^3J \approx {}^3J$ = 7.4 Hz, 2H), 1.06 (t, ^3J = 7.4 Hz, 3H).

^{13}C-NMR (101 MHz, $CDCl_3$, 300 K): *δ* [ppm] = 164.3, 153.4, 138.6, 130.5, 124.4, 122.7, 120.1, 119.6, 116.9, 34.4, 22.2, 14.2.

Spectral data matched those reported in the literature.[12]

4-Cyclopropyl-3,4-dihydroquinolin-2(1*H*)-one (*rac*-1o)

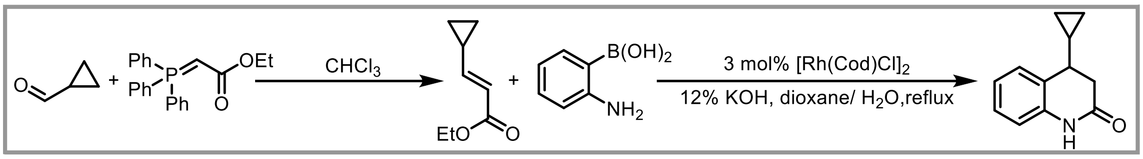

According to a modified procedure[8,13], ethyl (triphenylphosphoranylidene)acetate (6.0 mmol, 1.2 equiv) was added to a solution of cyclopropanecarboxaldehyde (5.0 mmol) in anhydrous dichloromethane (20 mL) under an inert atmosphere. The solution was stirred for 16 h at room

temperature. Volatiles were removed *in vacuo* and the resulting solid was purified by flash chromatography to give the desired ethyl (*E*)-3-cyclopropylacrylate (638mg, 4.55 mmol, 91%). To a suspension of chloro(1,5-cyclooctadiene)rhodium(I) dimer (0.06 mmol, 3 mol%), 2-aminophenylboronic acid pinacol boronate (4.00 mmol, 2.0 equiv) and ethyl (*E*)-3-cyclopropylacrylate (2 mmol) in dioxane (5.6 mL) was added 2.5 M KOH (0.48 mmol, 0.25 equiv). The mixture was heated under reflux conditions for 8 h. The solvent was removed *in vacuo*. The residue was dissolved in EtOAc (30 mL) and washed with 1 N HCl (2 x 10 mL), sated $NaHCO_3$ (20 mL), brine (20 mL) and then dried (Na_2SO_4). The organic layer was concentrated *in vacuo* to give the crude product which was purified by flash column chromatography to obtain compound *rac*-**1o** as white solid (179mg, 0.96 mmol, 48%).

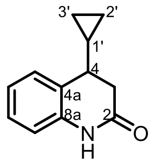

rac-**1o**

$C_{12}H_{13}NO$

M = 187.24 g mol^{-1}

TLC (Hex:EtOAc = 2:1): R_f = 0.37 [UV] [$KMnO_4$].

M.p.: 157°C.

^{1}H-NMR (400 MHz, $CDCl_3$, 300 K): δ [ppm] = 8.04 (s, 1H, NH), 7.39 (d, ^3J = 7.5 Hz, 1H, H5), 7.21 (*virt.* td, $^3J \approx {}^3J$ = 7.7 Hz, ^4J = 1.5 Hz, 1H, H7), 7.04 (*virt.* td, $^3J \approx {}^3J$ = 7.5 Hz, ^4J = 1.2 Hz, 1H, H6), 6.78 (dd, ^3J = 7.9 Hz, ^4J = 1.2 Hz, 1H, H8), 2.79 (dd, ^2J = 16.1 Hz, ^3J = 5.8 Hz, 1H, H3a), 2.62 (dd, ^2J = 16.1 Hz, ^3J = 7.7 Hz, 1H, H3b), 2.23 (*virt.* td, $^3J \approx {}^3J$ = 8.5, ^3J = 5.9 Hz, 1H, H4), 0.98 – 0.91 (m, 1H, H1'), 0.69 – 0.53 (m, 2H, H2a', H3a'), 0.38 – 0.32 (m, 1H, H2b'), 0.27 – 0.21 (m, 1H, H3b').

^{13}C-NMR (101 MHz, $CDCl_3$, 300 K): δ [ppm] = 171.2 (C2), 136.8 (8a), 127.9 (C7), 127.6 (C5), 127.4 (C4a), 123.4 (C6), 115.4 (C8), 41.2 (C4), 37.2 (C3), 15.2 (C1'), 4.9 (C2'), 3.2 (C3').

HRMS (ESI) *m/z*: calculated for $[M+H]^+$: 188.1071; found:188.1068.

IR (film): $\tilde{\nu}_{max}/cm^{-1}$ = 3181 (m, NH), 3076 (m, CH_{arom}), 2983(m, CH_{aliph}), 2920 (m, CH_{aliph}), 1676 (s, C=O), 1590 (m, $C{=}C_{arom}$), 1488(m, $C{=}C_{arom}$), 1372 (m, C-N), 753 (m, CH_{arom}).

4-Isopropyl-3,4-dihydroquinolin-2(1*H*)-one (*rac*-1p)

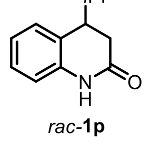

rac-**1p**

$C_{12}H_{15}NO$

M = 189.26 g mol^{-1}

According to GP C, compound **1p** was synthesized from 1-methoxyquinolin-2(1H)-one (1.0 mmol) with CuBr·SMe_2 (0.05 mmol, 5 mol%), and ligand *rac*-BINAP (0.06 mmol, 6 mol%) as white solid (57mg, 0.74 mmol, 30%).

TLC (Hex:EtOAc = 2:1): R_f = 0.38 [UV] [$KMnO_4$].

^{1}**H-NMR** (500 MHz, $CDCl_3$, 300 K): δ [ppm] = 8.13 (s, 1H), 7.21 – 7.12 (m, 2H), 7.00 (*virt.* td, $^{3}J \approx {}^{3}J$ = 7.5 Hz, ^{4}J = 1.2 Hz, 1H), 6.77 (dd, ^{3}J = 7.8 Hz, ^{4}J = 1.2 Hz, 1H), 2.74 – 2.67 (m, 3H), 1.93 – 1.87 (m, 1H), 0.95 (d, ^{3}J = 6.7 Hz, 3H), 0.89 (d, ^{3}J = 6.8 Hz, 3H).
^{13}**C-NMR** (101 MHz, $CDCl_3$, 300 K): δ [ppm] = 171.8, 137.0, 129.2, 127.7, 126.6, 122.9, 115.7, 43.1, 33.6, 31.8, 20.7, 19.3.
Spectral data matched those reported in the literature.[3]

4-(4,4,4-Trifluorobutyl)-3,4-dihydroquinolin-2(1*H*)-one (*rac*-1q)

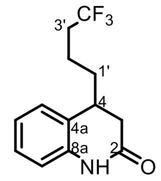

rac-**1q**
$C_{13}H_{14}F_3NO$
M = 257.26 g mol^{-1}

According to GP D, 4-(2-(trimethylsilyl)ethyl)quinolin-2(1H)-one (***6q***) was synthesized from 4-methylquinolin-2(1H)-one (4 mmol) and the 1,1,1-trifluoro-3-iodopropane (10 mmol, 2.5 equiv.) as white solid (540mg, 2.10 mmol, 53%). Then, compound *rac*-**1q** was prepared from quinolone **6q** (1 mmol) as white solid (206mg, 0.80 mmol, 80%).

TLC (Hex:EtOAc = 2:1): R_f = 0.35 [UV] [$KMnO_4$].

M.p.: 108 °C.

^{1}**H-NMR** (500 MHz, $CDCl_3$, 300 K): δ [ppm] = 8.05 (s, 1H, NH), 7.21 (*virt.* td, $^{3}J \approx {}^{3}J$ = 7.7 Hz, ^{4}J = 1.5 Hz, 1H, H5), 7.14 (dd, ^{3}J = 7.5 Hz, ^{4}J = 1.4 Hz, 1H, H7), 7.03 (*virt.* td, $^{3}J \approx {}^{3}J$ = 7.5 Hz, ^{4}J = 1.2 Hz, 1H, H6), 6.79 (dd, ^{3}J = 7.9 Hz, ^{4}J = 1.2 Hz, 1H, H8), 2.98 – 2.92 (m, 1H, H3), 2.80 (dd, ^{2}J = 16.2 Hz, ^{3}J = 6.1 Hz, 1H, H3a), 2.55 (dd, ^{2}J = 16.2 Hz, ^{3}J = 3.4 Hz, 1H, H3b), 2.15 – 1.93 (m, 2H, H3’), 1.70 – 1.60 (m, 3H, H1’, H2a’), 1.56 – 1.47 (m, 1H, H2b’).
^{13}**C-NMR** (101 MHz, $CDCl_3$, 300 K): δ [ppm] = 170.7 (C2), 136.3 (C8a), 128.0 (C7 or C5, undistinguishable), 127.9 (C7 or C5, undistinguishable), 126.9 (q, J = 277.8 Hz, CF_3), 126.6 (4a), 123.2 (C6), 115.9 (C8), 36.3 (C4), 36.2 (C3), 33.7 (q, J = 28.6 Hz, C3’), 33.1 (C1’), 19.5 (q, J = 3.0 Hz, C2’).
^{19}**F-NMR** (376 MHz, $CDCl_3$, 300 K)**:** δ [ppm] = –66.3.
HRMS (ESI) *m/z*: calculated for $[M+H]^+$: 258.1100; found: 258.1096.
IR (film): $\tilde{\nu}_{max}$/cm^{-1} = 3192 (m, NH), 3060 (m, CH_{arom}), 2969 (m, CH_{aliph}), 2919 (m, CH_{aliph}), 1679 (s, C=O), 1597 (m, $C{=}C_{arom}$), 1491(m, $C{=}C_{arom}$), 1388 (m, C-N), 1131 (m, CF_3), 756 (m, CH_{arom}).

4-(4,4,4-Trifluorobutyl)quinolin-2(1*H*)-one (6q)

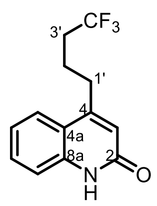

6q

$C_{13}H_{12}F_3NO$

M = 255.24 g mol^{-1}

TLC (Hex:EtOAc = 2:1): R_f = 0.24 [UV] [KMnO4].

M.p.: 161 °C.

^{1}H-NMR (400 MHz, $CDCl_3$, 300 K): δ [ppm] = 12.60 (s, 1H, NH), 7.69 (dd, ^3J = 8.2 Hz, ^4J = 1.3 Hz, 1H, H5), 7.54 (ddd, ^3J = 8.3 Hz, ^3J = 6.9 Hz, ^4J = 1.3 Hz, 1H, H7), 7.49 (dd, ^3J = 8.2 Hz, ^4J = 1.4 Hz, 1H, H8), 7.29 – 7.23 (m, 1H, H6), 6.61 (s, 1H, H3), 2.97 (t, ^3J = 7.7 Hz, 2H, H1'), 2.30 – 2.16 (m, 2H, H3'), 2.08 – 2.00 (m, 2H, H2').

^{13}C-NMR (101 MHz, $CDCl_3$, 300 K): δ [ppm] = 164.2 (C2), 151.6(C4), 138.8(C8a), 130.9 (C7), 127.1 (d, J = 276.5 Hz, CF_3), 124.0 (C5), 123.0 (C6), 120.0 (C3), 119.6 (4a), 117.2 (C8), 33.5 (q, J = 28.9 Hz, C3'), 31.1 (C1'), 21.3 (q, J = 3.0 Hz, C2').

^{19}F-NMR (376 MHz, $CDCl_3$, 300 K)**:** δ [ppm] = –66.0.

HRMS (ESI) *m/z*: calculated for $[M+H]^+$: 256.0944; found: 256.0940.

IR (film): $\tilde{v}_{max}/cm^{-1}$ = 3191 (m, NH), 3071 (m, CH_{arom}), 2951(m, CH_{aliph}), 2828 (m, CH_{aliph}), 1655 (s, C=O), 1514 (m, $C=C_{arom}$), 1441(m, $C=C_{arom}$), 1356 (m, C-N), 1113 (m, CF_3), 746 (m, CH_{arom}).

4-(2-(Trimethylsilyl)ethyl)-3,4-dihydroquinolin-2(1*H*)-one (*rac*-1r)

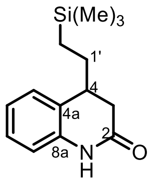

***rac*-1r**

$C_{14}H_{21}NOSi$

M = 247.41 g mol^{-1}

According to GP D, 4-(2-(trimethylsilyl)ethyl)quinolin-2(1H)-one (***6r***) was synthesized from 4-methylquinolin-2(1H)-one (4 mmol) and the (iodomethyl)trimethylsilane (10 mmol, 2.5 equiv.) as white solid (550mg, 2.24 mmol, 56%). Then, compound *rac*-**1r** was prepared from quinolone **6r** (1 mmol) as white solid (161mg, 0.65 mmol, 65%).

TLC (Hex:EtOAc = 2:1): R_f = 0.38 [UV] [KMnO4].

M.p.: 78 °C.

^{1}H-NMR (500 MHz, $CDCl_3$, 300 K): δ [ppm] = 8.26 (s, 1H), 7.21 – 7.15 (m, 2H, H5, H7), 7.01 (*virt.* td, $^3J \approx {}^3J$ = 7.5 Hz, ^4J = 1.2 Hz, 1H, H6), 6.79 (dd, ^3J = 7.8 Hz, ^4J = 1.2 Hz, 1H, H8), 2.84 (*virt.* qd, $^3J \approx {}^3J$ = 6.9, ^3J = 3.5 Hz, 1H, H4), 2.76 (dd, ^2J = 16.2 Hz, ^3J = 6.2 Hz, 1H, H3a), 2.62 (dd, ^2J = 16.1 Hz, ^3J = 3.6 Hz, 1H, H3b), 1.61 – 1.49 (m, 2H, H1'), 0.59 (ddd, ^2J = 14.2 Hz, ^3J = 12.0, ^3J = 5.3 Hz, 1H, H2a'), 0.42 (ddd, ^2J = 14.2 Hz, ^3J = 12.0, ^3J = 5.6 Hz, 1H, H2b'), -0.05 (s, 9H).

^{13}C-NMR (101 MHz, $CDCl_3$, 300 K): δ [ppm] = 171.6 (C2), 136.6 (8a), 128.2 (C7), 127.7 (C4a), 127.6 (C5), 123.1 (C6), 115.9 (C8), 39.5 (C4), 35.7 (C3), 28.8 (C2'), 13.8 (C1'), -1.7.

HRMS (ESI) *m/z*: calculated for $[M+H]^+$: 248.1465; found: 248.1463.

IR (film): $\tilde{\nu}_{max}/cm^{-1}$ = 3187 (m, NH), 3058 (m, CH_{arom}), 2953 (m, CH_{aliph}), 2914 (m, CH_{aliph}), 1682 (s, C=O), 1594 (m, $C{=}C_{arom}$), 1490(m, $C{=}C_{arom}$), 1383 (m, C-N), 747 (m, CH_{arom}).

4-(2-(Trimethylsilyl)ethyl)quinolin-2(1*H*)-one (6r)

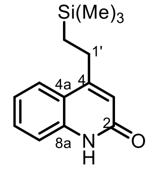

6r

$C_{14}H_{19}NOSi$

M = 245.40 g mol-1

TLC (Hex:EtOAc = 2:1): R_f = 0.25 [UV] [$KMnO_4$].

M.p.: 180 °C.

^{1}H-NMR (400 MHz, $CDCl_3$, 300 K): δ [ppm] = 11.09 (s, 1H, NH), 7.70 (dd, ^3J = 8.2 Hz, ^4J = 1.4 Hz, 1H, H5), 7.49 (ddd, ^3J = 8.3 Hz, ^3J = 7.1 Hz, ^4J = 1.3 Hz, 1H, H7), 7.34 (dd, ^3J = 8.3 Hz, ^4J = 1.3 Hz, 1H, H8), 7.24 (ddd, ^3J = 8.3 Hz, ^3J = 7.1 Hz, ^4J = 1.2 Hz, 1H, H6), 6.61 (d, ^4J = 1.1 Hz, 1H, H3), 2.88 – 2.79 (m, 2H, H1'), 0.98 – 0.89 (m, 2H, H2'), 0.11 (s, 9H).

^{13}C-NMR (101 MHz, $CDCl_3$, 300 K): δ [ppm] = 164.5 (C2), 155.8 (C4), 138.7(C8a), 130.4(C7), 124.2(C5), 122.5 (C6), 119.8 (C4a), 118.8 (C3), 116.8 (C8), 26.8 (C1'), 16.4 (C2'), -1.6.

HRMS (ESI) *m/z*: calculated for $[M+H]^+$: 246.1309; found: 246.1306.

IR (film): $\tilde{\nu}_{max}/cm^{-1}$ = 3145 (m, NH), 3060 (m, CH_{arom}), 2952 (m, CH_{aliph}), 2889 (m, CH_{aliph}), 1649 (s, C=O), 1559 (m, $C{=}C_{arom}$), 1430(m, $C{=}C_{arom}$), 1398 (m, C-N), 864 (m, C3-H), 740 (m, CH_{arom}).

Butyl 2-oxo-1,2,3,4-tetrahydroquinoline-4-carboxylate (*rac*-1s)

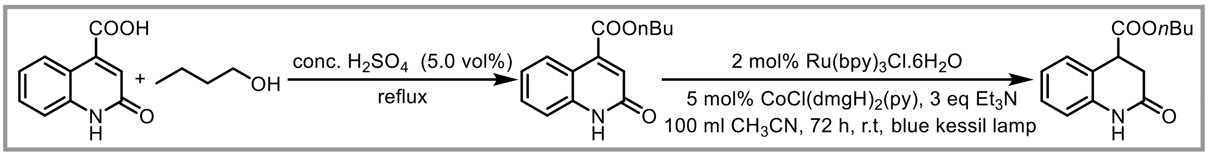

According to a modified procedure[14,15], 2-oxo-1,2-dihydroquinoline-4- carboxylic acid (10 mmol) was suspended in *n*-butanol (1 M, 10 mL), conc. H_2SO_4 was added (5 vol%) dropwise and the mixture was heated for 4 h. The suspension was diluted with EtOAc (50 mL) and filtered, before washing the organic layer with Na_2CO_3solution (30 mL) and brine (30 mL). The organic layer was dried over Na_2SO_4, filtered and the solvent was removed under reduced pressure. Normal-phase flash chromatography gave butyl 2-oxo-1,2-dihydroquinoline-4-carboxylate (***6s***) as light-yellow solid (1.47g, 5.99 mmol, 60%).

In an argon-filled glove box, a round-bottom flask (100 mL) was charged with compound **6s** (2 mmol), $CoCl(dmgH)_2(py)$ (0.1 mmol, 5 mol%), $Ru(bpy)_3Cl_2$•$6H_2O$ (0.04 mmol, 2 mol%) and CH_3CN (100 mL). After the degassing with Ar for 30 mins, the Et_3N (6 mmol, 3.0 equiv.) was added to the flask via syringe, and the syringe hole was carefully sealed with parafilm. The mixture was further irradiated by Kessil Tuna Blue LEDs for 72 hours at room temperature. After the reaction was finished, the mixture was concentrated under reduced pressure and purified by column chromatography to afford compound *rac*-**1s** as light-yellow solid (285mg, 1.15 mmol, 58%).

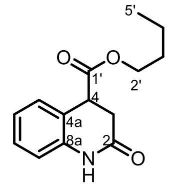
rac-**1s**
$C_{14}H_{17}NO_3$
M = 247.29 g mol^{-1}

TLC (EtOAc): R_f = 0.39 [UV] [$KMnO_4$].

M.p.: 104 °C.

^{1}H-NMR (400 MHz, $CDCl_3$, 300 K): δ [ppm] = 8.16 (s, 1H, NH), 7.29 (dd, ^3J = 7.7 Hz, ^4J = 1.3 Hz, 1H, H5), 7.23 (*virt.* td, $^3J \approx {}^3J$ = 7.7 Hz, ^4J = 1.4 Hz, 1H, H7), 7.03 (*virt.* td, $^3J \approx {}^3J$ = 7.6 Hz, ^4J = 1.2 Hz, 1H, H6), 6.79 (dd, ^3J = 7.9 Hz, ^4J = 1.2 Hz, 1H, H8), 4.10 (t, ^3J = 6.6 Hz, 2H, H2'), 3.93 (dd, ^3J = 6.6 Hz, ^3J = 4.0 Hz, 1H, H4), 2.98 (dd, ^2J = 16.4 Hz, ^3J = 4.0 Hz, 1H, H3a'), 2.79 (dd, ^2J = 16.4 Hz, ^3J = 6.7 Hz, 1H, H3b'), 1.59 – 1.53 (m, 2H, H3'), 1.35 – 1.25 (m, 2H, H4'), 0.87 (t, ^3J = 7.4 Hz, 3H, H5').

^{13}C-NMR (101 MHz, $CDCl_3$, 300 K): δ [ppm] = 171.8 (C1'), 169.4 (C2), 137.1 (C8a), 129.1 (C7 or C5, undistinguishable), 129.0 (C7 or C5, undistinguishable), 123.5 (C6), 120.4 (C4a), 116.0 (C8), 65.5 (C2'), 53.6, 42.7 (C4), 33.0 (C3), 30.6 (C3'), 19.1 (C4'), 13.7 (C5').

HRMS (ESI) *m/z*: calculated for $[M+H]^+$: 248.1281; found: 248.1279.

IR (film): $\tilde{v}_{max}/cm^{-1}$ = 3210 (m, NH), 3070 (m, CH_{arom}), 2975 (m, CH_{aliph}), 2932 (m, CH_{aliph}), 1717 (s, C=O), 1677 (s, C=O), 1597 (m, $C{=}C_{arom}$), 1493(m, $C{=}C_{arom}$), 1387 (m, C-N), 1330 (s, C-O-C), 1205 (s, C-O-C), 746 (m, CH_{arom}).

Butyl 2-oxo-1,2-dihydroquinoline-4-carboxylate (6s)

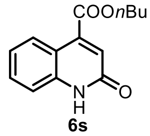

6s
$C_{14}H_{15}NO_3$
M = 245.28 g mol^{-1}

TLC (Hex:EtOAc = 2:1): R_f = 0.20 [UV] [$KMnO_4$].

^{1}H-NMR (300 MHz, $CDCl_3$, 300 K): δ [ppm] = 12.04 (s, 1H), 8.36 (dd, ^3J = 8.4 Hz, ^4J = 1.4 Hz, 1H), 7.57 (ddd, ^3J = 8.4 Hz, ^3J = 7.1 Hz, ^4J = 1.4 Hz, 1H), 7.43 (dd, ^3J = 8.3 Hz, ^4J = 1.3 Hz, 1H), 7.30 (ddd, ^3J = 8.4 Hz, ^3J = 7.1 Hz, ^4J = 1.3 Hz, 1H), 7.24 (s, 1H), 4.42 (t, ^3J = 6.6 Hz, 2H), 1.85 – 1.74 (m, 2H), 1.55 – 1.46 (m, 2H), 1.00 (t, ^3J = 7.4 Hz, 3H).

^{13}C-NMR (75 MHz, $CDCl_3$, 300 K): δ [ppm] = 165.4, 163.4, 141.2, 139.0, 131.4, 126.8, 124.1, 123.7, 117.2, 116.6, 66.1, 30.7, 19.4, 13.8.
Spectral data matched those reported in the literature.[14]

4-Methyl-1,4-dihydro-2*H*-benzo[*d*][1,3]oxazin-2-one (*rac*-8)

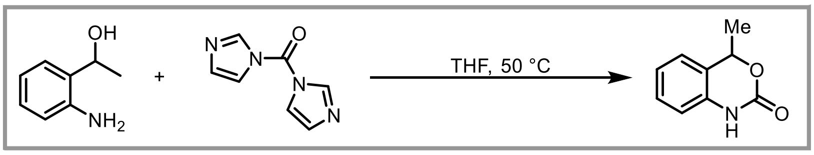

According to a modified procedure[16], to a solution of 1-(2-aminophenyl)ethan-1-ol (10 mmol) in dry THF (50 mL) was added 1,1'- carbonyldiimidazole (12 mmol, 1.2 equiv.) under Ar. The reaction solution was heated at 50 °C overnight. The solvent was removed *in vacuo* and the residue was dissolved in ethyl acetate (100 mL). The solution was washed with 1N aqueous hydrochloride solution (2 x 60 mL), brine (60 mL), and dried with Na_2SO_4. After removal of the solvent *in vacuo*, the residue purified by column chromatography to afford compound *rac*-**8** as light-yellow solid (207mg, 1.27 mmol, 13%).

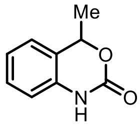

rac-**8**
$C_9H_9NO_2$
M = 163.18 g mol^{-1}

TLC (Hex:EtOAc = 2:1): R_f = 0.41 [UV] [$KMnO_4$].
^{1}H-NMR (400 MHz, $CDCl_3$, 300 K): δ [ppm] = 8.68 (s, 1H), 7.28 – 7.24 (m, 1H), 7.14 – 7.04 (m, 2H), 6.86 (dd, ^3J = 7.9 Hz, ^4J = 1.1 Hz, 1H), 5.52 (q, ^3J = 6.6 Hz, 1H), 1.72 (d, ^3J = 6.6 Hz, 3H).
^{13}C-NMR (101 MHz, $CDCl_3$, 300 K): δ [ppm] = 153.6, 135.1, 129.3, 123.9, 123.6, 122.8, 114.4, 76.1, 20.5.
Spectral data matched those reported in the literature.[16]

2-Methyl-2*H*-benzo[*b*][1,4]oxazin-3(4*H*)-one (*rac*-9)

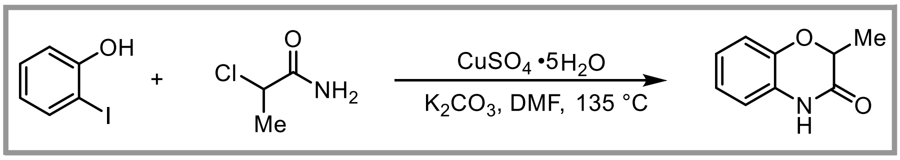

According to a modified procedure[17], 2-iodophenol (3 mmol), chloroacetamide (3.6 mmol, 1.2 equiv.), $CuSO_4{\cdot}H_2O$ (0.3 mmol, 10 mol%), and K_2CO_3 (6.6 mmol, 2.2 equiv.) were added into an oven-dried Schlenk tube. Seal the reaction tube, evacuate it, and refill it with argon gas three times. Subsequently, under an argon atmosphere, introduce 6 mL of DMF into the tube. The

Schlenk tube was heated to 135 °C and stirred for 12 h. After the reaction was completed, the heterogeneous mixture was cooled to room temperature, diluted with ethyl acetate (60 mL). The mixture was washed with water (50 mL), brine (50 mL), and dried with Na_2SO_4. After removal of the solvent *in vacuo*, the residue purified by column chromatography to obtain compound *rac*-**9** as light-yellow solid (367mg, 2.25 mmol, 75%).

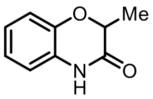

rac-**9**
$C_9H_9NO_2$
M = 163.18 g mol^{-1}

TLC (Hex:EtOAc = 2:1): R_f = 0.46 [UV] [$KMnO_4$].

^{1}H-NMR (500 MHz, $CDCl_3$, 300 K): δ [ppm] = 8.24 (s, 1H), 7.00 – 6.94 (m, 3H), 6.83 – 6.80 (m, 1H), 4.66 (q, ^3J = 6.8 Hz, 1H), 1.59 (d, ^3J = 6.8 Hz, 3H).

^{13}C-NMR (101 MHz, $CDCl_3$, 300 K): δ [ppm] = 168.3, 143.5, 126.7, 124.3, 122.8, 117.3, 115.7, 73.7, 16.4.

Spectral data matched those reported in the literature.[17]

1-Methyl-4-phenyl-3,4-dihydroquinolin-2(1*H*)-one (*rac*-10)

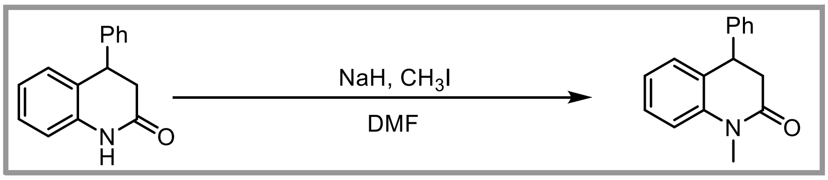

According to a modified procedure[18], to a stirred solution of 4-phenyl-3,4-dihydroquinolin-2(1H)-one (0.5 mmol) in anhydrous DMF (6 mL), sodium hydride (60%, 1.0 mmol, 2.0 equiv.) was added portionwise at 0 °C. The reaction mixture was then allowed to warm to room temperature, followed by the addition of iodomethane (0.55 mmol, 1.1 equiv.), and the resulting mixture was stirred for 3 hours. Upon completion of the reaction, the mixture was poured into cold water (60 mL) and extracted with ethyl acetate (2 × 60 mL). The combined organic layers were washed with brine (60 mL), dried over anhydrous sodium sulfate, filtered, and concentrated under reduced pressure. The crude product was purified by flash chromatography on silica gel to afford compound *rac*-**10** as a white solid (111 mg, 0.47 mmol, 94%).

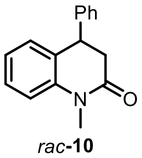

rac-**10**
$C_{16}H_{15}NO$
M = 237.30 g mol^{-1}

TLC (Hex:EtOAc = 4:1): R_f = 0.25 [UV] [$KMnO_4$].

^{1}H-NMR (500 MHz, $CDCl_3$, 300 K): δ [ppm] = 7.35 – 7.25 (m, 4H), 7.19 – 7.15 (m, 2H), 7.06 (dd, ^3J = 8.1 Hz, ^4J = 1.1 Hz, 1H), 7.00 (*virt.* td, $^3J \approx {}^3J$ = 7.5 Hz, ^4J = 1.1 Hz, 1H), 6.93 (d, ^3J = 7.5 Hz, 1H), 4.24 (dd, ^3J = 8.3 Hz, ^3J = 6.4 Hz, 1H), 3.40 (s, 3H), 3.03 – 2.91 (m, 2H).

^{13}C-NMR (101 MHz, $CDCl_3$, 300 K): δ [ppm] = 169.4, 141.2, 140.5, 129.3, 129.0, 128.2, 128.0, 127.9, 127.3, 123.1, 115.0, 41.7, 39.0, 29.7.

Spectral data matched those reported in the literature.[19]

4-Phenyl-3,4-dihydroquinolin-2(1*H*)-one-4-*d* (*rac*-1a-*d₁*)

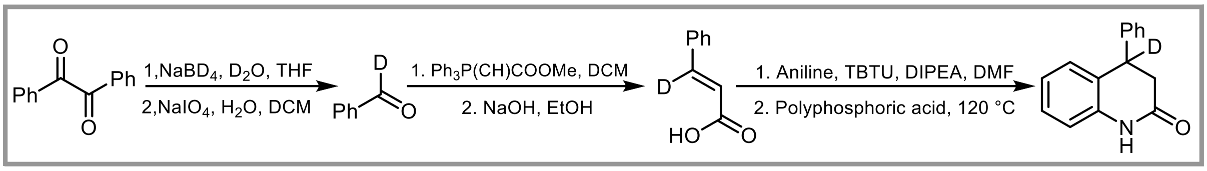

According to a modified procedure[20,21], benzil (5 mmol) was added to a mixture of THF (20 mL) and D_2O (2 mL), and the solution was cooled to 0 °C. Sodium borodeuteride (12 mmol, 2.4 equiv.) was added in small portions under stirring. The reaction mixture was then allowed to warm to room temperature and stirred overnight. The reaction was quenched with water (10 mL), and a saturated aqueous solution of ammonium chloride was added to adjust the pH to neutral. The aqueous phase was extracted with ethyl acetate (3 × 60 mL). The combined organic extracts were dried over anhydrous Na_2SO_4, filtered, and concentrated under reduced pressure to afford the corresponding diol, which was used directly in the next step without further purification.

The crude diol was oxidized at room temperature overnight by treatment with sodium periodate (10 mmol) in a biphasic mixture of dichloromethane (DCM, 10 mL) and water (10 mL). The aqueous layer was extracted with DCM (3 × 20 mL), and the combined organic extracts were washed with brine (30 mL), dried over Na_2SO_4, and concentrated under reduced pressure to afford deuteriobenzaldehyde (***S1***) as a colorless oil (977 mg, 9.1 mmol, 91% yield, >99% D/H).

To a stirred solution of deuteriobenzaldehyde (8 mmol) in $CHCl_3$ (25 mL) at room temperature was added methyl (triphenylphosphoranylidene)acetate (11.2 mmol, 1.4 equiv.). The reaction mixture was stirred for 12 h at room temperature. The volatiles were removed under reduced pressure to yield a residue, which was subjected to hydrolysis with 3M NaOH in EtOH (10 m L) for 5 h. Whereafter, the reaction mixture was concentrated under reduced pressure and extracted with ethyl acetate (3 × 30 mL). The combined organic layers were dried over anhydrous Na_2SO_4, filtered, and concentrated under reduced pressure. The crude product was purified by flash chromatography on silica gel to afford deuterated cinnamic acid (***S2***) as a white solid (623 mg, 4.2 mmol, 52% yield, >99% D/H).

Following GP B, compound *rac*-**1a**-d_1 was synthesized from aniline (4 mmol) and deuterated cinnamic acid (4.4 mmol, 1.1 equiv.) as a white solid (431 mg, 1.9 mmol, 48% yield, >99% D/H).

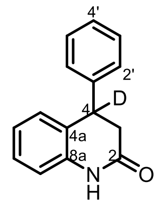

rac-**1a**-*d1*
$C_{15}H_{12}DNO$
M = 224.28 g mol^{-1}

TLC (Hex:EtOAc = 2:1): R_f = 0.38 [UV] [KMnO4].

M.p.: 185°C.

^{1}H-NMR (400 MHz, $CDCl_3$, 300 K): δ [ppm] = 8.47 (s, 1H, NH), 7.36 – 7.32 (m, 2H, H3’,H5’), 7.29 – 7.26 (m, 1H, H4’), 7.23 – 7.19 (m, 3H, H2’, H4’, H7), 6.97 (*virt.* td, $^{3}J \approx {}^{3}J$ = 7.4 Hz, ^{4}J = 1.2 Hz, 1H, H6), 6.92 (dd, ^{3}J = 7.6 Hz, ^{4}J = 1.6 Hz, 1H, H5), 6.85 (dd, ^{3}J = 7.9 Hz, ^{4}J = 1.2 Hz, 1H, H8), 2.98 – 2.89 (m, 2H, H3).

^{13}C-NMR (101 MHz, $CDCl_3$, 300 K): δ [ppm] = 170.7 (C2), 141.6 (C1’), 137.2 (C8a), 129.1(C3’, C5’), 128.6 (C5), 128.2 (C7), 127.9 (C2’, C4’), 127.4 (C4’), 126.8 (C4a), 123.5 (C6), 115.7 (C8), 41.8 (t, J = 20.2 Hz, C4),38.5 (C3).

HRMS (ESI) *m/z*: calculated for $[M+H]^+$: 225.1133; found:225.1131.

IR (film): $\tilde{\nu}_{max}/cm^{-1}$ = 3190 (m, NH), 3055 (m, CH_{arom}), 2979 (m, CH_{aliph}), 2896 (m, CH_{aliph}), 1670 (s, C=O), 1593 (m, $C=C_{arom}$), 1486(m, $C=C_{arom}$), 1371 (m, C-N), 758 (m, CH_{arom}).

Deuteriobenzaldehyde (S1)

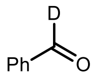

S1
C_7H_5DO
M = 107.13 g mol^{-1}

TLC (Hex:EtOAc = 10:1): R_f = 0.61 [UV] [KMnO4].

^{1}H-NMR (400 MHz, $CDCl_3$, 300 K): δ [ppm] = 7.90 – 7.88 (m, 2H), 7.68 – 7.60 (m, 1H), 7.55 – 7.52 (m, 2H).

^{13}C-NMR (101 MHz, $CDCl_3$, 300 K): δ [ppm] = 192.1 (t, J = 26.8 Hz), 136.4 (t, J = 3.5 Hz), 134.5, 129.8, 129.1.

Cinnamic-3-*d* acid (S2)

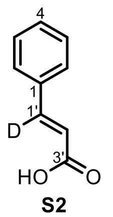

S2
$C_9H_7DO_2$
M = 149.17 g mol^{-1}

TLC (Hex:EtOAc = 2:1): R_f = 0.45 [UV] [KMnO4].

M.p.: 136 °C.

^{1}H-NMR (400 MHz, $CDCl_3$, 300 K): δ [ppm] = 7.60 – 7.53 (m, 2H, H3’, H5’), 7.44 – 7.40 (m, 3H, H2’, H6’, H4’), 6.46 (s, 1H, H2’).

^{13}C-NMR (101 MHz, $CDCl_3$, 300 K): δ [ppm] = 172.7 (C3’), 146.9 (t, J = 24.2 Hz, C1’), 134.2 (C1), 130.9 (C4), 129.1 (C2, C6), 128.5 (C3, C5), 117.4 (C2’).

HRMS (ESI) *m/z*: calculated for $[M+H]^+$: 150.0660; found: 150.0661.

IR (film): $\tilde{\nu}_{max}/cm^{-1}$ = 3067 (m, CH_{arom}), 2981 (m, CH_{aliph}), 2924 (m, CH_{aliph}), 2555 (m, OH), 1668 (s, C=O), 1616 (m, $C=C_{arom}$), 1418(m, $C=C_{arom}$), 1300 (m, C-O), 895(m, $C_{alkenyl}$-H), 693 (m, CH_{arom}).

4-Phenyl-3,4-dihydroquinolin-2(1*H*)-one-3,3-d$_2$ (*rac*-1a-*d$_2$*)

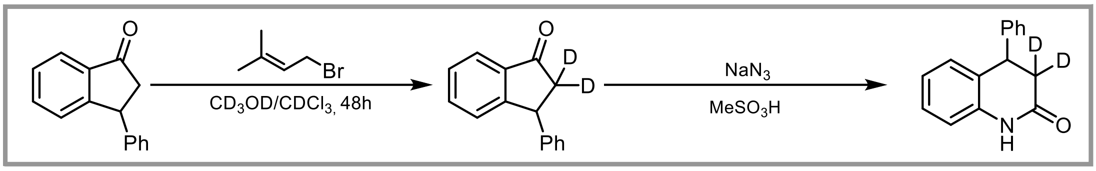

According to a modified procedure[22], a 25 mL reaction flask equipped with a glass stopper was charged with 3-phenyl-1-indanone (5 mmol), CD_3OD (10 mL), CD_3Cl (2 mL), and prenyl bromide (5 mol%). The reaction mixture was stirred at room temperature for 48 h. Upon completion, the reaction mixture was evaporated under reduced pressure to remove the catalyst and solvents, affording the deuterated ketone **S3** as a rufous solid in quantitative yield with >96% deuterium incorporation.

The resulting deuterated ketone **S3** was dissolved in methanesulfonic acid (10 mL), and sodium azide (12.31 mmol) was added at 0 °C. The reaction mixture was then allowed to warm to room temperature and stirred for an additional 3 h. After completion, the reaction mixture was poured onto ice and extracted with ethyl acetate (3 × 100 mL). The combined organic layers were washed with brine (150 mL), dried over anhydrous Na_2SO_4, filtered, and concentrated under reduced pressure to afford the crude product. Purification by column chromatography over silica gel yielded the desired deuterated product *rac*-**1a**-*d$_2$* as a white solid (528 mg, 2.35 mmol, 47% yield, >96% D/H).

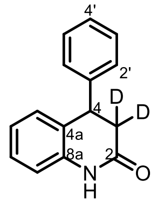

rac-**1a**-*d$_2$*
$C_{15}H_{11}D_2NO$
M = 225.11 g mol^{-1}

TLC (Hex:EtOAc = 2:1): R_f = 0.38 [UV] [$KMnO_4$].

M.p.: 183°C.

^{1}H-NMR (400 MHz, $CDCl_3$, 300 K): δ [ppm] = 8.79 (s, 1H, NH), 7.37 – 7.31 (m, 2H, H3',H5'), 7.30 – 7.25 (m, 1H, H4'), 7.23 – 7.18 (m, 3H, H2', H4', H7), 7.00 – 6.91 (m, 2H, H6, H5), 6.88 (dd, ^3J = 7.9 Hz, ^4J = 1.2 Hz, 1H, H8), 4.31 – 4.29 (m, 1H, H4), 2.96 – 2.91 (m, 0.08, H3a,H3b).

^{13}C-NMR (101 MHz, $CDCl_3$, 300 K): δ [ppm] = 170.9 (C2), 141.6 (C1'), 137.2 (C8a), 129.1(C3', C5'), 128.6 (C5), 128.2 (C7), 128.0 (C2', C4'), 127.4 (C4'), 126.8 (C4a), 123.5 (C6), 115.8 (C8), 42.0 (C3).

HRMS (ESI) *m/z*: calculated for $[M+H]^+$: 226.1195; found:226.1194.

IR (film): $\tilde{\nu}_{max}/cm^{-1}$ = 3190 (m, NH), 3055 (m, CH_{arom}), 2980 (m, CH_{aliph}), 2892 (m, CH_{aliph}), 1666 (s, C=O), 15943 (m, $C{=}C_{arom}$), 1487(m, $C{=}C_{arom}$), 1378 (m, C-N), 759 (m, CH_{arom}).

3-Phenyl-2,3-dihydro-1*H*-inden-1-one-2,2-*d₂* (S3)

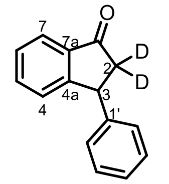

S3
$C_{15}H_{10}D_2O$
M = 210.27 g mol^{-1}

TLC (Hex:EtOAc = 10:1): R_f = 0.45 [UV] [KMnO4].

M.p.: 78 °C.

^{1}H-NMR (500 MHz, $CDCl_3$, 300 K): δ [ppm] = δ 7.81 (dd, J = 7.8, 1.0 Hz, 1H, H7), 7.57 (*virt*. td, $^3J \approx {}^3J$ = 7.4 Hz, ^4J = 1.3 Hz, 1H, H5), 7.46 – 7.39 (m, 1H, H6), 7.35 – 7.21 (m, 4H, H4, H3', H4'), 7.14 – 7.11 (m, 2H, H2'), 4.57 (s, 1H, H3), 3.25 – 3.19 (m, 0.04 H, H2a), 2.69 – 2.67 (m, 0.03 H, H2b).

^{13}C-NMR (101 MHz, $CDCl_3$, 300 K): δ [ppm] = δ 206.2 (C1), 158.1 (C4a), 143.8 (C1'), 137.0 (C7a), 135.2 (C5), 129.1 (C3'), 128.0 (C6), 127.8 (C2'), 127.1 (C4' or C4), 127.0 (C4' or C4), 123.6 (C7), 44.5 (C3).

HRMS (ESI) m/z: calculated for $[M+H]^+$: 211.1086; found: 211.1086.

IR (film): $\tilde{\nu}$max/cm^{-1} = 1702 (s, C=O), 1599 (m, $C{=}C_{arom}$), 1452(m, $C{=}C_{arom}$), 697 (m, CH_{arom}).

7. Photochemical Deracemization Reactions

(*R*)-4-Phenyl-3,4-dihydroquinolin-2(1*H*)-one (1a)

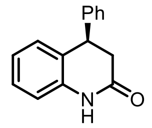

1a
$C_{15}H_{13}NO$
M = 223.28 g mol^{-1}

According to GP E, under condition A, a degassed solution of *rac*-**1a** (8.93 mg, 40.0 µmol, 1.00 equiv.), enantiomerically pure (−)-benzophenone **2** (805 µg, 2.0 µmol, 5.0 mol%) $CoCl_2$(dmgH)($dmgH_2$) (361 µg, 1.0 µmol, 2.5 mol%) and $(TripS)_2$ (188 µg, 0.4 µmol, 1 mol%) in 4 mL acetone was irradiated at λ = 366 nm for 5 h. After irradiation, the solvent was evaporated and the residue was purified by FCC (SiO_2, *n*-pentane/EtOAc) to obtain compound **1a** (4.11 mg, 18.4 µmol, 46%, 96% *ee*) as a white solid.

TLC (Hex:EtOAc = 2:1): R_f = 0.38 [UV] [$KMnO_4$].

Optical Rotation: $[\alpha]_D^{25}$: −68 (*c* = 1.0, CH_2Cl_2) [96% *ee*].

Chiral HPLC: 96% *ee* (OD-RH 150 × 4.6 mm, MeCN/water = 20/80 → 100/0 over 30 min, 1 ml/min, λ = 210 nm); t_R = 15.56 min (minor, *ent*-**1a**), 16.75 min (major, **1a**).

Under condition B, a degassed solution of *rac*-**1a** (8.93 mg, 40.0 µmol, 1.00 equiv.), enantiomerically pure (−)-benzophenone **2** (1.61 mg, 4.0 µmol, 10.0 mol%) $CoCl_2$(dmgH)($dmgH_2$) (722 µg, 2.0 µmol, 5.0 mol%) and $(TripS)_2$ (942 µg, 2.0 µmol, 5 mol%) in 4 mL acetone was irradiated at λ = 366 nm for 3 h. After irradiation, the solvent was evaporated and the residue was purified by FCC (SiO_2, *n*-pentane/EtOAc) to obtain compound **1a** (4.73 mg, 21.2 µmol, 53%, 92% *ee*) as a white solid.

TLC (Hex:EtOAc = 2:1): R_f = 0.38 [UV] [$KMnO_4$].

Chiral HPLC: 92% *ee* (OD-RH 150 × 4.6 mm, MeCN/water = 20/80 → 100/0 over 30 min, 1 ml/min, λ = 210 nm); t_R = 15.54 min (minor, *ent*-**1a**), 16.75 min (major, **1a**).

0.5 mmol scale:

According to GP E, a degassed solution of *rac*-**1a** (111.7 mg, 0.5 mmol, 1.00 equiv.) and enantiomerically (−)-benzophenone **2** (10.1 mg, 250 µmol, 5.0 mol%) $CoCl_2$(dmgH)($dmgH_2$) (4.51 mg, 125 µmol, 2.5 mol%) and $(TripS)_2$ (2.35 mg, 50 µmol, 1 mol%) in 50 mL acetone was divided into 12 sets and irradiated at λ = 366 nm for 5 h. After irradiation, the solvent was evaporated and the residue was purified by flash column chromatography (SiO_2, EtOAc) to obtain compound **1a** (54.1 mg, 242 µmol, 48%, 96% *ee*) as a colorless solid and dehydrogenated product **6a** (43.4 mg, 196 µmol, 39%).

TLC (Hex:EtOAc = 2:1): R_f = 0.38 [UV] [$KMnO_4$].

Chiral HPLC: 96% *ee* (OD-RH 150 × 4.6 mm, MeCN/water = 20/80 → 100/0 over 30 min, 1 ml/min, λ = 210 nm); t_R = 15.57 min (minor, *ent*-**1a**), 16.77 min (major, **1a**).

4-Phenylquinolin-2(1*H*)-one (6a)

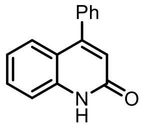

6a

$C_{15}H_{11}NO$

M = 221.26 g mol^{-1}

TLC (Hex:EtOAc = 2:1): R_f = 0.25 [UV] [KMnO4].

^{1}H-NMR (400 MHz, $CDCl_3$, 300 K): δ [ppm] = 12.27 (s, 1H), 7.60 – 7.45 (m, 8H), 7.19 (ddd, ^3J = 8.2 Hz, ^3J = 6.7 Hz, ^4J = 1.6 Hz, 1H), 6.71 (s, 1H).

^{13}C-NMR (101 MHz, $CDCl_3$, 300 K): δ [ppm] = 164.2, 153.7, 139.1, 137.3, 130.9, 129.0, 129.0, 128.8, 126.9, 122.8, 120.9, 119.8, 116.8.

Spectral data matched those reported in the literature.[12]

(*R*)-4-(4-Methoxyphenyl)-3,4-dihydroquinolin-2(1*H*)-one (1b)

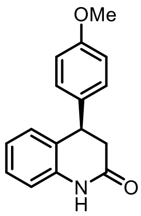

1b

$C_{16}H_{15}NO_2$

M = 253.30 g mol^{-1}

According to GP E, under condition A, a degassed solution of *rac*-**1b** (10.1 mg, 40.0 µmol, 1.00 equiv.), enantiomerically pure (−)-benzophenone **2** (805 µg, 2.0 µmol, 5.0 mol%) $CoCl_2$(dmgH)($dmgH_2$) (361 µg, 1.0 µmol, 2.5 mol%) and $(TripS)_2$ (188 µg, 0.4 µmol, 1 mol%) in 4 mL acetone was irradiated at λ = 366 nm for 3 h. After irradiation, the solvent was evaporated and the residue was purified by FCC (SiO_2, *n*-pentane/EtOAc) to obtain compound **1b** (3.44 mg, 13.6 µmol, 34%, 95% *ee*) as a white solid.

TLC (Hex:EtOAc = 2:1): R_f = 0.31 [UV] [KMnO4].

Optical Rotation: $[\alpha]_D^{25}$: −52 (c = 1.0, CH_2Cl_2) [95% *ee*].

Chiral HPLC: 95% *ee* (IC 250 × 4.6 mm, *n*-Hep/*iso*-PrOH = 70/30, 1 ml/min, λ = 210 nm); t_R = 11.55 min (minor, *ent*-**1b**), 16.49 min (major, **1b**).

(*R*)-4-(*p*-Tolyl)-3,4-dihydroquinolin-2(1*H*)-one (1c)

According to GP E, under condition A, a degassed solution of *rac*-**1c** (9.49 mg, 40.0 µmol, 1.00 equiv.), enantiomerically pure (−)-benzophenone **2** (805 µg, 2.0 µmol, 5.0 mol%) $CoCl_2$(dmgH)($dmgH_2$) (361 µg, 1.0 µmol, 2.5 mol%) and $(TripS)_2$ (188 µg, 0.4 µmol, 1 mol%) in 4 mL acetone was irradiated at λ = 366 nm for 5 h. After irradiation, the solvent was

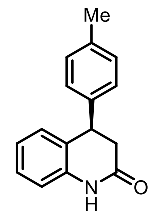

1c
$C_{16}H_{15}NO$
M = 237.30 g mol^{-1}

evaporated and the residue was purified by FCC (SiO_2, *n*-pentane/EtOAc) to obtain compound **1c** (3.82 mg, 16.1 µmol, 40%, 92% *ee*) as a white solid.

TLC (Hex:EtOAc = 2:1): R_f = 0.39 [UV] [$KMnO_4$].

Optical Rotation: $[\alpha]_D^{25}$: –48 (*c* = 1.0, CH_2Cl_2) [92% *ee*].

Chiral HPLC: 92% *ee* (OD-RH 150 × 4.6 mm, MeCN/water = 20/80 → 100/0 over 30 min, 1 ml/min, λ = 210 nm); t_R = 16.70 min (minor, *ent*-**1c**), 17.79 min (major, **1c**).

(*R*)-4-(4-Fluorophenyl)-3,4-dihydroquinolin-2(1*H*)-one (1d)

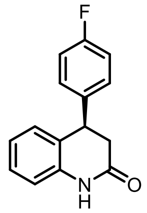

1d
$C_{15}H_{12}FNO_2$
M = 241.27 g mol^{-1}

According to GP E, under condition A, a degassed solution of *rac*-**1d** (9.65 mg, 40.0 µmol, 1.00 equiv.), enantiomerically pure (–)-benzophenone **2** (805 µg, 2.0 µmol, 5.0 mol%) $CoCl_2$(dmgH)(dmgH_2) (361 µg, 1.0 µmol, 2.5 mol%) and $(TripS)_2$ (188 µg, 0.4 µmol, 1 mol%) in 4 mL acetone was irradiated at λ = 366 nm for 5 h. After irradiation, the solvent was evaporated and the residue was purified by FCC (SiO_2, *n*-pentane/EtOAc) to obtain compound **1d** (3.86 mg, 16.0 µmol, 40%, 97% *ee*) as a white solid.

TLC (Hex:EtOAc = 2:1): R_f = 0.37 [UV] [$KMnO_4$].

Optical Rotation: $[\alpha]_D^{25}$: –40 (*c* = 1.0, CH_2Cl_2) [97% *ee*].

Chiral HPLC: 97% *ee* (OD-RH 150 × 4.6 mm, MeCN/water = 20/80 → 100/0 over 30 min, 1 ml/min, λ = 210 nm); t_R = 15.29 min (minor, *ent*-**1d**), 17.16 min (major, **1d**).

(*R*)-4-(4-Chlorophenyl)-3,4-dihydroquinolin-2(1*H*)-one (1e)

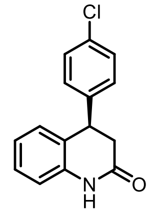

1e
$C_{15}H_{12}ClNO$
M = 257.72 g mol^{-1}

According to GP E, under condition A, a degassed solution of *rac*-**1e** (10.3 mg, 40.0 µmol, 1.00 equiv.), enantiomerically pure (–)-benzophenone **2** (805 µg, 2.0 µmol, 5.0 mol%) $CoCl_2$(dmgH)(dmgH_2) (361 µg, 1.0 µmol, 2.5 mol%) and $(TripS)_2$ (188 µg, 0.4 µmol, 1 mol%) in 4 mL acetone was irradiated at λ = 366 nm for 5 h. After irradiation, the solvent was evaporated and the residue was purified by FCC (SiO_2, *n*-pentane/EtOAc) to obtain compound **1e** (4.36 mg, 16.9 µmol, 42%, 93% *ee*) as a white solid.

TLC (Hex:EtOAc = 2:1): R_f = 0.37 [UV] [$KMnO_4$].

Optical Rotation: $[\alpha]_D^{25}$: –56 (*c* = 1.0, CH_2Cl_2) [93% *ee*].

Chiral HPLC: 93% *ee* (OD-RH 150 × 4.6 mm, MeCN/water = 20/80 → 100/0 over 30 min, 1 ml/min, λ = 210 nm); t_R = 17.42 min (minor, *ent*-**1e**), 18.82 min (major, **1e**).

(*R*)-4-(4-Bromophenyl)-3,4-dihydroquinolin-2(1*H*)-one (1f)

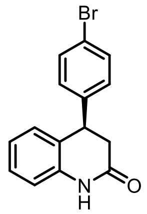

1h

$C_{15}H_{12}BrNO$
M = 302.17 g mol^{-1}

According to GP E, under condition B, a degassed solution of *rac*-**1f** (12.1 mg, 40.0 µmol, 1.00 equiv.), enantiomerically pure (−)-benzophenone **2** (1.61 mg, 4.0 µmol, 10.0 mol%) $CoCl_2(dmgH)(dmgH_2)$ (722 µg, 2.0 µmol, 5.0 mol%) and $(TripS)_2$ (942 µg, 2.0 µmol, 5 mol%) in 4 mL acetone was irradiated at λ = 366 nm for 5 h. After irradiation, the solvent was evaporated and the residue was purified by FCC (SiO_2, *n*-pentane/EtOAc) to obtain compound **1f** (5.08 mg, 16.8 µmol, 42%, 94% *ee*) as a white solid.

TLC (Hex:EtOAc = 2:1): R_f = 0.36 [UV] [$KMnO_4$].

Optical Rotation: $[\alpha]_D^{25}$: −28 (*c* = 1.0, CH_2Cl_2) [94% *ee*].

Chiral HPLC: 94% *ee* (OD-RH 150 × 4.6 mm, MeCN/water = 20/80 → 100/0 over 30 min, 1 ml/min, λ = 210 nm); t_R = 18.20 min (minor, *ent*-**1f**), 19.32 min (major, **1f**).

(*R*)-4-(3-Chlorophenyl)-3,4-dihydroquinolin-2(1*H*)-one (1g)

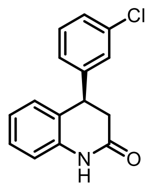

1g

$C_{15}H_{12}ClNO$
M = 257.72 g mol^{-1}

According to GP E, under condition A, a degassed solution of *rac*-**1g** (10.3 mg, 40.0 µmol, 1.00 equiv.), enantiomerically pure (−)-benzophenone **2** (805 µg, 2.0 µmol, 5.0 mol%) $CoCl_2(dmgH)(dmgH_2)$ (361 µg, 1.0 µmol, 2.5 mol%) and $(TripS)_2$ (188 µg, 0.4 µmol, 1 mol%) in 4 mL acetone was irradiated at λ = 366 nm for 5 h. After irradiation, the solvent was evaporated and the residue was purified by FCC (SiO_2, *n*-pentane/EtOAc) to obtain compound **1g** (3.84 mg, 14.9 µmol, 37%, 95% *ee*) as a white solid.

TLC (Hex:EtOAc = 2:1): R_f = 0.36 [UV] [$KMnO_4$].

Optical Rotation: $[\alpha]_D^{25}$: −38 (*c* = 1.0, CH_2Cl_2) [95% *ee*].

Chiral HPLC: 95% *ee* (OD-RH 150 × 4.6 mm, MeCN/water = 20/80 → 100/0 over 30 min, 1 ml/min, λ = 210 nm); t_R = 17.69 min (minor, *ent*-**1g**), 18.51 min (major, **1g**).

(*R*)-6-Fluoro-4-phenyl-3,4-dihydroquinolin-2(1*H*)-one (1h)

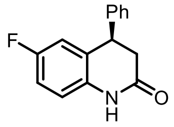

1h
$C_{15}H_{12}FNO$
M = 241.27 g mol^{-1}

According to GP E, under condition B, a degassed solution of *rac*-**1h** (9.65 mg, 40.0 µmol, 1.00 equiv.), enantiomerically pure (−)-benzophenone **2** (1.61 mg, 4.0 µmol, 10.0 mol%) $CoCl_2(dmgH)(dmgH_2)$ (722 µg, 2.0 µmol, 5.0 mol%) and $(TripS)_2$ (942 µg, 2.0 µmol, 5 mol%) in 4 mL acetone was irradiated at λ = 366 nm for 15 h. After irradiation, the solvent was evaporated and the residue was purified by FCC (SiO_2, *n*-pentane/EtOAc) to obtain compound **1h** (3.09 mg, 12.8 µmol, 32%, 96% *ee*) as a white solid.

TLC (Hex:EtOAc = 2:1): $R_f = 0.36$ [UV] [$KMnO_4$].

Optical Rotation: $[\alpha]_D^{25}$: −56 (*c* = 1.0, CH_2Cl_2) [96% *ee*].

Chiral HPLC: 96% *ee* (AD-H, 250 × 4.6 mm, *n*-Hep/*iso*-PrOH = 90/10, 1 ml/min, λ = 210 nm); t_R = 11.53 min (major, **1h**), 12.46 min (minor, *ent*-**1h**).

(*R*)-7-Methyl-4-phenyl-3,4-dihydroquinolin-2(1H)-one (1i)

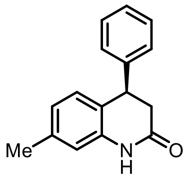

1i
$C_{16}H_{15}NO$
M = 237.30 g mol^{-1}

According to GP E, under condition B, a degassed solution of *rac*-**1i** (9.49 mg, 40.0 µmol, 1.00 equiv.), enantiomerically pure (-)-benzophenone **2** (1.61 mg, 4.0 µmol, 10.0 mol%) $CoCl_2(dmgH)(dmgH_2)$ (722 µg, 2.0 µmol, 5.0 mol%) and $(TripS)_2$ (942 µg, 2.0 µmol, 5 mol%) in 4 mL acetone was irradiated at λ = 366 nm for 5 h. After irradiation, the solvent was evaporated and the residue was purified by FCC (SiO_2, *n*-pentane/EtOAc) to obtain compound **1i** (2.94 mg, 12.4 µmol, 31%, 98% *ee*) as a white solid.

TLC (Hex:EtOAc = 2:1): $R_f = 0.36$ [UV] [$KMnO_4$].

Optical Rotation: $[\alpha]_D^{28}$: -22 (*c* = 1.0, CH_2Cl_2) [98% *ee*].

Chiral HPLC: 98% *ee* (OJ-RH 150 × 4.6 mm, MeCN/water = 20/80 → 100/0 over 30 min, 1 ml/min, λ = 210 nm); t_R = 13.04 min (major, **1i**), 14.43 min (minor, *ent*-**1i**).

(*R*)-7-Methoxy-4-phenyl-3,4-dihydroquinolin-2(1H)-one (1j)

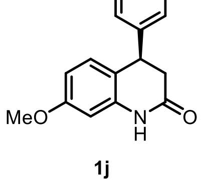

1j
$C_{16}H_{15}NO_2$
M = 253.30 g mol^{-1}

According to GP E, under condition B, a degassed solution of *rac*-**1j** (10.1 mg, 40.0 µmol, 1.00 equiv.), enantiomerically pure (-)-benzophenone **2** (1.61 mg, 4.0 µmol, 10.0 mol%) $CoCl_2(dmgH)(dmgH_2)$ (722 µg, 2.0 µmol, 5.0 mol%) and $(TripS)_2$ (942 µg, 2.0 µmol, 5 mol%) in 4 mL acetone was irradiated at λ = 366 nm for 3 h. After irradiation, the solvent was evaporated

and the residue was purified by FCC (SiO_2, *n*-pentane/EtOAc) to obtain compound **1j** (3.65 mg, 14.4 µmol, 36%, 96% *ee*) as a white solid.

TLC (Hex:EtOAc = 2:1): R_f = 0.31 [UV] [$KMnO_4$].

Optical Rotation: $[\alpha]_D^{28}$: -20 (*c* = 1.0, CH_2Cl_2) [96% *ee*].

Chiral HPLC: 96% *ee* (OJ-RH 150 × 4.6 mm, MeCN/water = 20/80 → 100/0 over 30 min, 1 ml/min, λ = 210 nm); t_R = 12.94 min (major, **1j**), 13.69 min (minor, *ent*-**1j**).

(*R*)-7-Chloro-4-phenyl-3,4-dihydroquinolin-2(1*H*)-one (1k)

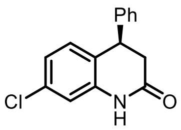

1k
$C_{15}H_{12}ClNO$
M = 257.72 g mol^{-1}

According to GP E, under condition B, a degassed solution of *rac*-**1k** (10.3 mg, 40.0 µmol, 1.00 equiv.), enantiomerically pure (–)-benzophenone **2** (1.61 mg, 4.0 µmol, 10.0 mol%) $CoCl_2(dmgH)(dmgH_2)$ (722 µg, 2.0 µmol, 5.0 mol%) and $(TripS)_2$ (942 µg, 2.0 µmol, 5 mol%) in 4 mL acetone was irradiated at λ = 366 nm for 5 h. After irradiation, the solvent was evaporated and the residue was purified by FCC (SiO_2, *n*-pentane/EtOAc) to obtain compound **1k** (3.81 mg, 14.8 µmol, 37%, 97% *ee*) as a white solid.

TLC (Hex:EtOAc = 2:1): R_f = 0.36 [UV] [$KMnO_4$].

Optical Rotation: $[\alpha]_D^{25}$: –56 (*c* = 1.0, CH_2Cl_2) [97% *ee*].

Chiral HPLC: 97% *ee* (OD-RH 150 × 4.6 mm, MeCN/water = 20/80 → 100/0 over 30 min, 1 ml/min, λ = 210 nm); t_R = 19.31 min (minor, *ent*-**1k**), 23.27 min (major, **1k**).

(*S*)-4-Methyl-3,4-dihydroquinolin-2(1*H*)-one (1l)

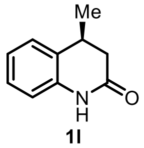

1l
$C_{10}H_{11}NO$
M = 161.20 g mol^{-1}

According to GP E, under condition A, a degassed solution of *rac*-**1l** (6.45 mg, 40.0 µmol, 1.00 equiv.), enantiomerically pure (–)-benzophenone **2** (805 µg, 2.0 µmol, 5.0 mol%) $CoCl_2(dmgH)(dmgH_2)$ (361 µg, 1.0 µmol, 2.5 mol%) and $(TripS)_2$ (188 µg, 0.4 µmol, 1 mol%) in 4 mL acetone was irradiated at λ = 366 nm for 3 h. After irradiation, the solvent was evaporated and the residue was purified by FCC (SiO_2, *n*-pentane/EtOAc) to obtain compound **1l** (2.53 mg, 15.7 µmol, 39%, 99% *ee*) as a white solid and dehydrogenated product **6l** (3.37 mg, 21.2 µmol, 53%).

TLC (Hex:EtOAc = 2:1): R_f = 0.37 [UV] [$KMnO_4$].

Optical Rotation: $[\alpha]_D^{25}$: –28 (*c* = 1.0, CH_2Cl_2) [99% *ee*].

Chiral HPLC: 99% *ee* (AS-RH 150 × 4.6 mm, MeCN/water = 20/80 → 100/0 over 30 min, 1 ml/min, λ = 210 nm); t_R = 10.49 min (major, **1l**), 11.46 min (minor, *ent*-**1l**).

4-Methylquinolin-2(1*H*)-one (6l)

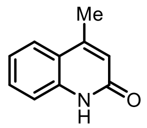

6l

C_9H_9NO

M = 159.19 g mol^{-1}

TLC (Hex:EtOAc = 2:1): R_f = 0.25 [UV] [KMnO4].

^{1}H-NMR (400 MHz, $CDCl_3$, 300 K): δ [ppm] = 12.10 (s, 1H), 7.70 (dd, ^3J = 8.1 Hz, ^4J = 1.3 Hz, 1H), 7.52 (ddd, ^3J = 8.4 Hz, ^3J =7.0, ^4J = 1.4 Hz, 1H), 7.44 (dd, ^3J = 8.2 Hz, ^4J = 1.3 Hz, 1H), 7.30 – 7.22 (m, 1H), 6.61 (d, ^4J = 1.3 Hz, 1H), 2.53 (d, ^4J = 1.2 Hz, 3H).

^{13}C-NMR (101 MHz, $CDCl_3$, 300 K): δ [ppm] = 164.2, 149.6, 138.3, 130.7, 124.6, 122.8, 120.8, 120.6, 116.7, 19.3.

Spectral data matched those reported in the literature.[22]

(*S*)-4-Ethyl-3,4-dihydroquinolin-2(1*H*)-one (1m)

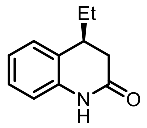

1m

$C_{11}H_{13}NO$

M = 175.23 g mol^{-1}

According to GP E, under condition A, a degassed solution of *rac*-**1m** (7.01 mg, 40.0 µmol, 1.00 equiv.), enantiomerically pure (−)-benzophenone **2** (805 µg, 2.0 µmol, 5.0 mol%) $CoCl_2$(dmgH)(dmgH_2) (361 µg, 1.0 µmol, 2.5 mol%) and $(TripS)_2$ (188 µg, 0.4 µmol, 1 mol%) in 4 mL acetone was irradiated at λ = 366 nm for 3 h. After irradiation, the solvent was evaporated and the residue was purified by FCC (SiO_2, *n*-pentane/EtOAc) to obtain compound **1m** (3.31 mg, 18.9 µmol, 47%, 96% *ee*) as a white solid.

TLC (Hex:EtOAc = 2:1): R_f = 0.38 [UV] [KMnO4].

Optical Rotation: $[\alpha]_D^{26}$: −22 (c = 1.0, CH_2Cl_2) [96% *ee*].

Chiral HPLC: 96% *ee* (AS-RH 150 × 4.6 mm, MeCN/water = 20/80 → 100/0 over 30 min, 1 ml/min, λ = 210 nm); t_R = 12.84 min (minor, *ent*-**1m**), 13.87 min (major, **1m**).

(*S*)-4-Propyl-3,4-dihydroquinolin-2(1*H*)-one (1n)

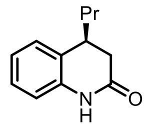

1n

$C_{12}H_{15}NO$

M = 189.26 g mol^{-1}

According to GP E, under condition A, a degassed solution of *rac*-**1n** (7.57 mg, 40.0 µmol, 1.00 equiv.), enantiomerically pure (−)-benzophenone **2** (805 µg, 2.0 µmol, 5.0 mol%) $CoCl_2$(dmgH)(dmgH_2) (361 µg, 1.0 µmol, 2.5 mol%) and $(TripS)_2$ (188 µg, 0.4 µmol, 1 mol%) in 4 mL acetone was irradiated at λ = 366

nm for 3 h. After irradiation, the solvent was evaporated and the residue was purified by FCC (SiO_2, *n*-pentane/EtOAc) to obtain compound **1n** (3.81 mg, 20.1 µmol, 50%, 92% *ee*) as a white solid and dehydrogenated product **6n** (2.91 mg, 15.5 µmol, 39%).

TLC (Hex:EtOAc = 2:1): R_f = 0.37 [UV] [$KMnO_4$].

Optical Rotation: $[\alpha]_D^{26}$: −8 (*c* = 1.0, CH_2Cl_2) [92% *ee*].

Chiral HPLC: 92% *ee* (AS-RH 150 × 4.6 mm, MeCN/water = 20/80 → 100/0 over 30 min, 1 ml/min, λ = 210 nm); t_R = 14.81 min (minor, *ent*-**1n**), 15.90 min (major, **1n**).

(*S*)-4-Cyclopropyl-3,4-dihydroquinolin-2(1*H*)-one (1o)

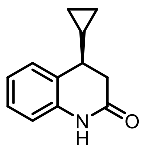

1o
$C_{12}H_{13}NO$
M = 187.24 g mol^{-1}

According to GP E, under condition A, a degassed solution of *rac*-**1o** (7.49 mg, 40.0 µmol, 1.00 equiv.), enantiomerically pure (−)-benzophenone **2** (805 µg, 2.0 µmol, 5.0 mol%) $CoCl_2$(dmgH)(dmgH$_2$) (361 µg, 1.0 µmol, 2.5 mol%) and $(TripS)_2$ (188 µg, 0.4 µmol, 1 mol%) in 4 mL acetone was irradiated at λ = 366 nm for 5 h. After irradiation, the solvent was evaporated and the residue was purified by FCC (SiO_2, *n*-pentane/EtOAc) to obtain compound **1o** (3.46 mg, 18.5 µmol, 46%, 94% *ee*) as a light yellow solid and **6o** (2.07 mg, 11.2 µmol, 28%) as a light-yellow solid.

TLC (Hex:EtOAc = 2:1): R_f = 0.37 [UV] [$KMnO_4$].

Optical Rotation: $[\alpha]_D^{26}$: −68 (*c* = 1.0, CH_2Cl_2) [94% *ee*].

Chiral HPLC: 94% *ee* (IC 250 × 4.6 mm, *n*-Hep/*iso*-PrOH = 70/30, 1 ml/min, λ = 210 nm); t_R = 7.78 min (major, **1o**), 8.57 min (minor, *ent*-**1o**).

4-Cyclopropylquinolin-2(1*H*)-one (6o)

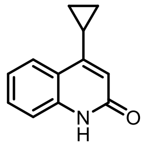

6o
$C_{12}H_{11}NO$
M = 185.23 g mol^{-1}

TLC (Hex:EtOAc = 2:1): R_f = 0.25 [UV] [$KMnO_4$].

^{1}H-NMR (500 MHz, $CDCl_3$, 300 K): *δ* [ppm] = 12.00 (s, 1H), 8.05 (dd, ^3J = 8.2 Hz, ^4J = 1.2 Hz, 1H), 7.58 – 7.50 (*virt.* t, ^3J = 7.8 Hz, 1H), 7.43 (d, ^3J = 8.2 Hz, 1H), 7.28 (*virt.* t, $^3J \approx {}^3J$ = 7.8 Hz, 1H), 6.43 (s, 1H), 2.25 – 2.18 (m, 1H), 1.17 – 1.10 (m, 2H), 0.88 – 0.81 (m, 2H).

^{13}C-NMR (101 MHz, $CDCl_3$, 300 K): *δ* [ppm] = 164.5, 155.0, 138.1, 130.7, 124.8, 122.9, 121.3, 116.8, 116.2, 12.7, 7.7.

HRMS (ESI) *m/z*: calculated for $[M+H]^+$: 186.0913; found:186.0911.

(*S*)-4-Isopropyl-3,4-dihydroquinolin-2(1*H*)-one (1p)

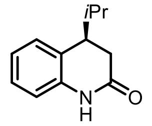

1p

$C_{12}H_{15}NO$

M = 189.26 g mol^{-1}

According to GP M, under condition A, a degassed solution of *rac*-**1p** (7.57 mg, 40.0 μmol, 1.00 equiv.), enantiomerically pure (–)-benzophenone **2** (805 μg, 2.0 μmol, 5.0 mol%) $CoCl_2(dmgH)(dmgH_2)$ (361 μg, 1.0 μmol, 2.5 mol%) and $(TripS)_2$ (188 μg, 0.4 μmol, 1 mol%) in 4 mL acetone was irradiated at λ = 366 nm for 15 h. After irradiation, the solvent was evaporated and the residue was purified by FCC (SiO_2, *n*-pentane/EtOAc) to obtain compound **1p** (3.12 mg, 16.5 μmol, 41%, 95% *ee*) as a white solid.

TLC (Hex:EtOAc = 2:1): R_f = 0.38 [UV] [$KMnO_4$].

Optical Rotation: $[\alpha]_D^{26}$: –2 (*c* = 1.0, CH_2Cl_2) [95% *ee*].

Chiral HPLC: 95% *ee* (AS-RH 150 × 4.6 mm, MeCN/water = 20/80 → 100/0 over 30 min, 1 ml/min, λ = 210 nm); t_R = 14.15 min (minor, *ent*-**1p**), 16.33 min (major, **1p**).

(*S*)-4-(4,4,4-Trifluorobutyl)-3,4-dihydroquinolin-2(1*H*)-one (1q)

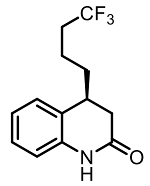

1q

$C_{13}H_{14}F_3NO$

M = 257.26 g mol^{-1}

According to GP E, under condition A, a degassed solution of *rac*-**1q** (10.3 mg, 40.0 μmol, 1.00 equiv.), enantiomerically pure (–)-benzophenone **2** (805 μg, 2.0 μmol, 5.0 mol%) $CoCl_2(dmgH)(dmgH_2)$ (361 μg, 1.0 μmol, 2.5 mol%) and $(TripS)_2$ (188 μg, 0.4 μmol, 1 mol%) in 4 mL acetone was irradiated at λ = 366 nm for 5 h. After irradiation, the solvent was evaporated and the residue was purified by FCC (SiO_2, *n*-pentane/EtOAc) to obtain compound **1q** (4.84 mg, 18.8 μmol, 47%, 98% *ee*) as a white solid.

TLC (Hex:EtOAc = 2:1): R_f = 0.35 [UV] [$KMnO_4$].

Optical Rotation: $[\alpha]_D^{26}$: –38 (*c* = 1.0, CH_2Cl_2) [98% *ee*].

Chiral HPLC: 98% *ee* (IC 250 × 4.6 mm, *n*-Hep/*iso*-PrOH = 70/30, 1 ml/min, λ = 210 nm); t_R = 6.14 min (major, **1q**), 7.71 min (minor, *ent*-**1q**).

(*S*)-4-(2-(Trimethylsilyl)ethyl)-3,4-dihydroquinolin-2(1*H*)-one (1r)

According to GP E, under condition A, a degassed solution of *rac*-**1r** (9.90 mg, 40.0 μmol, 1.00 equiv.), enantiomerically pure (–)-benzophenone **2** (805 μg, 2.0 μmol, 5.0 mol%) $CoCl_2(dmgH)(dmgH_2)$ (361 μg, 1.0 μmol, 2.5 mol%) and $(TripS)_2$ (188 μg, 0.4 μmol, 1 mol%)

in 4 mL acetone was irradiated at λ = 366 nm for 3 h. After irradiation, the solvent was evaporated and the residue was purified by FCC (SiO_2, *n*-pentane/EtOAc) to obtain compound **1r** (4.06 mg, 16.4 µmol, 41%, 98% *ee*) as a white solid.

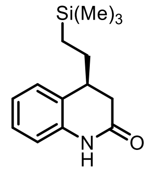

1r
$C_{14}H_{21}NOSi$
M = 247.41 g mol^{-1}

TLC (Hex:EtOAc = 2:1): R_f = 0.38 [UV] [$KMnO_4$].

Optical Rotation: $[\alpha]_D^{26}$: –30 (c = 1.0, CH_2Cl_2) [98% *ee*].

Chiral HPLC: 98% *ee* (OD-RH 150 × 4.6 mm, MeCN/water = 20/80 → 100/0 over 30 min, 1 ml/min, λ = 210 nm); t_R = 17.55 min (major, **1r**), 18.53 min (minor, *ent*-**1r**).

(*S*)-Butyl 2-oxo-1,2,3,4-tetrahydroquinoline-4-carboxylate (1s)

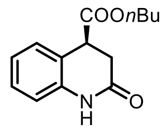

1s
$C_{14}H_{17}NO_3$
M = 247.29 g mol^{-1}

According to GP E, under condition B, a degassed solution of *rac*-**1s** (9.89 mg, 40.0 µmol, 1.00 equiv.), enantiomerically pure (−)-benzophenone **2** (1.61 mg, 4.0 µmol, 10.0 mol%) $CoCl_2(dmgH)(dmgH_2)$ (722 µg, 2.0 µmol, 5.0 mol%) and $(TripS)_2$ (942 µg, 2.0 µmol, 5 mol%) in 4 mL acetone was irradiated at λ = 366 nm for 24 h. After irradiation, the solvent was evaporated and the residue was purified by FCC (SiO_2, *n*-pentane/EtOAc) to obtain coumpound **1s** (4.06 mg, 16.4 µmol, 41%, 90% *ee*) as a white solid.

TLC (Hex:EtOAc = 2:1): R_f = 0.39 [UV] [$KMnO_4$].

Optical Rotation: $[\alpha]_D^{26}$: +8 (c = 1.0, CH_2Cl_2) [90% *ee*].

Chiral HPLC: 90% *ee* (IA 250 × 4.6 mm, *n*-Hep/*iso*-PrOH = 90/10, 1 ml/min, λ = 210 nm); t_R = 13.05 min (major, **1s**), 25.04 min (minor, *ent*-**1s**).

(*R*)-4-Methyl-1,4-dihydro-2*H*-benzo[*d*][1,3]oxazin-2-one e (8)

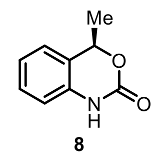

8
$C_9H_9NO_2$
M = 163.18 g mol^{-1}

According to GP E, under condition B, a degassed solution of *rac*-**8** (6.53 mg, 40.0 µmol, 1.00 equiv.), enantiomerically pure (−)-benzophenone **2** (1.61 mg, 4.0 µmol, 10.0 mol%) $CoCl_2(dmgH)(dmgH_2)$ (722 µg, 2.0 µmol, 5.0 mol%) and $(TripS)_2$ (942 µg, 2.0 µmol, 5 mol%) in 4 mL acetone was irradiated at λ = 366 nm for 5 h. After irradiation, the solvent was evaporated and the residue was purified by FCC (SiO_2, *n*-pentane/EtOAc) to obtain compound **8** (3.67 mg, 22.5 µmol, 56%, 98% *ee*) as a white solid.

TLC (Hex:EtOAc = 2:1): R_f = 0.41 [UV] [$KMnO_4$].

Optical Rotation: $[\alpha]_D^{26}$: +8 (c = 1.0, CH_2Cl_2) [98% *ee*].

Chiral HPLC: 98% *ee* (IC 250 × 4.6 mm, *n*-Hep/*iso*-PrOH = 70/30, 1 ml/min, λ = 210 nm); t_R = 11.20 min (major, **8**), 13.57 min (minor, *ent*-**8**).

(*S*)-2-Methyl-2*H*-benzo[*b*][1,4]oxazin-3(4*H*)-one (9)

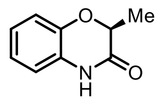

9

$C_9H_9NO_2$

M = 163.18 g mol^{-1}

According to GP E, under condition A, a degassed solution of *rac*-**9** (6.53 mg, 40.0 µmol, 1.00 equiv.), enantiomerically pure (−)-benzophenone **2** (805 µg, 2.0 µmol, 5.0 mol%) $CoCl_2(dmgH)(dmgH_2)$ (361 µg, 1.0 µmol, 2.5 mol%) and $(TripS)_2$ (188 µg, 0.4 µmol, 1 mol%) in 4 mL acetone was irradiated at λ = 366 nm for 5 h. After irradiation, the solvent was evaporated and the residue was purified by FCC (SiO_2, *n*-pentane/EtOAc) to obtain compound **9** (3.28 mg, 20.1 µmol, 50%, 90% *ee*) as a white solid.

TLC (Hex:EtOAc = 2:1): R_f = 0.46 [UV] [$KMnO_4$].

Optical Rotation: $[\alpha]_D^{26}$: +14 (c = 1.0, CH_2Cl_2) [90% *ee*].

Chiral HPLC: 90% *ee* (AS-RH 150 × 4.6 mm, MeCN/water = 20/80 → 100/0 over 30 min, 1 ml/min, λ = 210 nm); t_R = 9.46 min (minor, *ent*-**9**), 10.79 min (major, **9**).

(*R*)-1-Methyl-4-phenyl-3,4-dihydroquinolin-2(1*H*)-one (10)

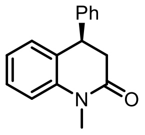

10

$C_{16}H_{15}NO$

M = 237.30 g mol^{-1}

According to GP E, under condition A, a degassed solution of *rac*-**10** (9.49 mg, 40.0 µmol, 1.00 equiv.), enantiomerically pure (−)-benzophenone **2** (805 µg, 2.0 µmol, 5.0 mol%) $CoCl_2(dmgH)(dmgH_2)$ (361 µg, 1.0 µmol, 2.5 mol%) and $(TripS)_2$ (188 µg, 0.4 µmol, 1 mol%) in 4 mL acetone was irradiated at λ = 366 nm for 5 h. After irradiation, the solvent was evaporated and the residue was purified by FCC (SiO_2, *n*-pentane/EtOAc) to obtain compound **10** (5.24 mg, 22.1 µmol, 55%, 2% *ee*) as a white solid and **11**(3.58 mg, 15.2 µmol, 38%) as a white solid.

TLC (Hex:EtOAc = 4:1): R_f = 0.25 [UV] [$KMnO_4$].

Chiral HPLC: 2% *ee* (AS-RH 150 × 4.6 mm, MeCN/water = 20/80 → 100/0 over 30 min, 1 ml/min, λ = 210 nm); t_R = 15.67 min (minor, *ent*-**10**), 16.63 min (major, **10**).

1-Methyl-4-phenylquinolin-2(1*H*)-one (11)

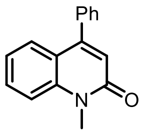

11

$C_{16}H_{13}NO$

M = 235.29 g mol^{-1}

TLC (Hex:EtOAc = 2:1): R_f = 0.41 [UV] [KMnO4].

^{1}H-NMR (500 MHz, $CDCl_3$, 300 K): δ [ppm] = 7.62 – 7.54 (m, 2H), 7.54 – 7.40 (m, 6H), 7.18 (*virt*. t, $^3J \approx {}^3J$ = 7.5 Hz, 1H), 6.69 (s, 1H), 3.79 (s, 3H).

^{13}C-NMR (101 MHz, $CDCl_3$, 300 K): δ [ppm] = 162.1, 151.1, 140.5, 137.3, 130.8, 129.1, 128.8, 128.7, 127.9, 122.1, 121.4, 120.7, 114.6, 29.6.

Spectral data matched those reported in the literature.[19]

Downstream Synthetic Transformations

(*3S*,*4R*)-3-Phenyl-4-phenyl-3,4-dihydroquinolin-2(1*H*)-one (12)

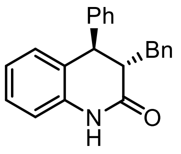

12
$C_{22}H_{19}NO$
M = 313.40 g mol^{-1}

According to a modified procedure[24], to a solution of **1a** (17.9 mg, 80.0 µmol, 1.00 equiv.) in tetrahydrofuran (1 mL), 1.6 M *n*-BuLi in hexanes (2.2 equiv) was added at 0 °C. The resulting brown solution was stirred at 0 °C for 30 minutes. Afterwards, benzyl bromide (2.0 equiv) was added at 0 °C, and the reaction was quenched within 1 minute by the addition of an excess of methanol (0.5 mL). The solvent was evaporated under reduced pressure, and the residue was purified by flash column chromatography (FCC) on silica gel using a solvent system of *n*-pentane/EtOAc to yield compound **12** diastereomerically pure (> 20/1 *dr*) (20.5 mg, 65.3 µmol, 82% yield, 97% *ee*) as a white solid.

TLC (Hex:EtOAc = 2:1): R_f = 0.49 [UV] [$KMnO_4$].

^{1}H-NMR (400 MHz, $CDCl_3$, 300 K): δ [ppm] = 8.34 (s, 1H), 7.35 – 7.30 (m, 2H), 7.28 – 7.13 (m, 7H), 7.07 – 7.02 (m, 2H), 6.93 – 6.86 (m, 3H), 3.96 (d, ^3J = 2.9 Hz, 1H), 3.17 – 3.09 (m, 2H), 2.70 (td, ^3J = 12.1 Hz, ^3J = 3.3 Hz, 1H).

^{13}C-NMR (101 MHz, $CDCl_3$, 300 K): δ [ppm] = 172.5, 142.0, 138.4, 136.7, 130.1, 129.4, 128.9, 128.8, 128.3, 127.6, 127.0, 126.9, 124.5, 123.9, 115.6, 50.5, 45.2, 36.4.

Optical Rotation: $[\alpha]_D^{26}$: −98 (*c* = 1.0, CH_2Cl_2) [97% *ee*].

Chiral HPLC: 97% *ee* (AS-RH 150 × 4.6 mm, MeCN/water = 20/80 → 100/0 over 30 min, 1 ml/min, λ = 210 nm); t_R = 18.66 min (major, **12**), 20.06 min (minor, *ent*-**12**).

(*R*)-4-Phenyl-1,2,3,4-tetrahydroquinoline (13)

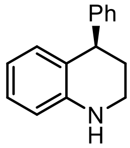

13
$C_{15}H_{15}O$
M = 209.29 g mol^{-1}

According to a modified procedure[24], to a solution of **1a** (17.9 mg, 80.0 µmol, 1.00 equiv.) in tetrahydrofuran (1 mL), 1.0 M BH_3·THF in THF (5.0 equiv) was added. The mixture was refluxed for 12 hours. After completion, the reaction was quenched by the addition of MeOH (0.5 mL) under ice-water cooling, and the solvents were removed under reduced pressure. A 5% aqueous solution of hydrochloric acid (1 mL) was then added to the residue, and the mixture was refluxed for 1 hour. Following this, saturated Na_2CO_3 (2 mL) and saturated NaCl (2 mL) were added, and the mixture was extracted with $CHCl_3$ (3 × 10 mL). The combined organic extracts were dried over Na_2SO_4, filtered, and concentrated under reduced pressure. The residue

was purified by FCC (SiO_2, *n*-pentane/EtOAc) to obtain compound **13** (11.9 mg, 56.9 µmol, 71%, 95% *ee*) as a white solid.

TLC (Hex:EtOAc = 4:1): R_f = 0.51 [UV] [$KMnO_4$].

^{1}H-NMR (500 MHz, $CDCl_3$, 300 K): δ [ppm] = 7.34 – 7.29 (m, 2H), 7.25 – 7.21 (m, 1H), 7.19 – 7.15 (m, 2H), 7.04 (*virt.* td, $^3J \approx {}^3J$ = 7.6 Hz, ^4J = 1.6 Hz, 1H), 6.77 (d, ^3J = 7.5 Hz, 1H), 6.61 – 6.57 (m, 2H), 4.17 (t, ^3J = 6.1 Hz, 1H), 3.80 (bs, 1H), 3.32 (ddd, ^2J = 11.0 Hz, ^3J = 7.0 Hz, ^3J = 3.7 Hz, 1H), 3.29 (ddd, ^2J = 11.5 Hz, ^3J = 8.2 Hz, ^3J = 3.5 Hz, 1H), 2.29 – 2.19 (m, 1H), 2.08 (dtd, ^2J = 13.3 Hz, ^3J = 6.9 Hz, ^3J = 3.6 Hz, 1H).

^{13}C-NMR (101 MHz, $CDCl_3$, 300 K): δ [ppm] = 146.8, 145.1, 130.6, 128.8, 128.4, 127.4, 126.2, 123.6, 117.2, 114.3, 43.0, 39.3, 31.2.

Optical Rotation: $[\alpha]_D^{26}$: –106 (c = 1.0, CH_2Cl_2) [95% *ee*].

Chiral HPLC: 95% *ee* (OJ-RH 150 × 4.6 mm, MeCN/water = 20/80 → 100/0 over 30 min, 1 ml/min, λ = 210 nm); t_R = 21.23 min (major, **13**), 24.02 min (minor, *ent*-**13**).

(*R*)-1,4-Diphenyl-3,4-dihydroquinolin-2(1*H*)-one (14)

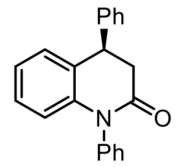

14

$C_{21}H_{17}NO$

M = 299.37 g mol^{-1}

According to a modified procedure[25], to a stirred mixture of **1a** (17.9 mg, 80.0 µmol, 1.00 equiv.), K_2CO_3 (2.1 equiv.), *trans*-1,2-cyclohexanediamine (0.2 equiv.), and bromobenzene (3.0 equiv.) in 1,4-dioxane (2 mL), copper(I) iodide (CuI, 0.2 equiv.) was added. The reaction mixture was refluxed overnight under an argon atmosphere at 125 °C. After cooling to room temperature, the mixture was extracted with ethyl acetate (3 × 10 mL). The combined organic layers were dried over Na_2SO_4, filtered, and concentrated under reduced pressure. The residue was purified by FCC (SiO_2, *n*-pentane/EtOAc) to yield the desired product **14** (19.2 mg, 64.1µmol, 80%, 94%) as a white solid.

TLC (Hex:EtOAc = 4:1): R_f = 0.25 [UV] [$KMnO_4$].

^{1}H NMR (500 MHz, $CDCl_3$, 300 K): δ [ppm] = 7.55 – 7.50 (m, 2H), 7.46 – 7.41 (m, 1H), 7.40 – 7.36 (m, 2H), 7.33 – 7.21 (m, 5H), 7.09 (*virt.* td, $^3J \approx {}^3J$ = 8.1 Hz, ^4J = 1.9 Hz, 1H), 7.04 – 7.00 (m, 2H), 6.47 (d, ^3J = 8.1 Hz, 1H), 4.42 (*virt.* t, $^3J \approx {}^3J$ = 6.9 Hz, 1H), 3.21 – 3.12 (m, 2H).

^{13}C-NMR (101 MHz, $CDCl_3$, 300 K): δ [ppm] = 169.1, 141.6, 141.3, 138.5, 130.0, 129.1, 129.1, 128.7, 128.4, 128.3, 127.9, 127.7, 127.4, 123.4, 117.5, 41.9, 39.5.

Optical Rotation: $[\alpha]_D^{26}$: –4 (c = 1.0, CH_2Cl_2) [94% *ee*].

Chiral HPLC: 94% *ee* (OJ-RH 150 × 4.6 mm, MeCN/water = 20/80 → 100/0 over 30 min, 1 ml/min, λ = 210 nm); t_R = 18.23 min (minor, *ent*-**14**), 20.62 min (major, **14**)

(*R*)-4-Phenyl-3,4-dihydroquinoline-2(1*H*)-thione (15)

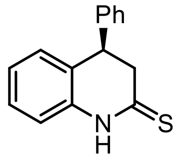

15
$C_{15}H_{13}NS$
M = 239.34 g mol^{-1}

According to a modified procedure[26], a mixture of **1a** (17.9 mg, 80.0 µmol, 1.00 equiv.) and Lawesson's reagent (0.5 equiv.) in toluene (3 mL) was heated to reflux for 15 min. After cooling to room temperature, the mixture was extracted with ethyl acetate (3 × 5 mL). The combined organic layers were dried over Na_2CO_3, filtered, and concentrated under reduced pressure. The residue was purified by FCC (SiO_2, *n*-pentane/EtOAc) to obtain compound **15** (12.5 mg, 52.2 µmol, 65%, 95% *ee*) as a white solid.

TLC (Hex:EtOAc = 5:1): R_f = 0.65 [UV] [$KMnO_4$].

^{1}H-NMR (400 MHz, $CDCl_3$, 300 K): δ [ppm] = 9.84 (s, 1H), 7.35 – 7.32 (m, 2H), 7.29 – 7.23 (m, 2H), 7.21 – 7.17 (m, 2H), 7.06 (*virt.* td, $^3J \approx {}^3J$ = 7.5 Hz, ^4J = 1.2 Hz, 1H), 6.98 – 6.91 (m, 2H), 4.25 – 4.18 (dd, ^3J = 9.0 Hz, ^3J = 6.5 Hz, 1H), 3.44 – 3.32 (m, 2H).

^{13}C-NMR (101 MHz, $CDCl_3$, 300 K): δ [ppm] = 199.5, 141.0, 136.2, 129.1, 129.1, 128.8, 128.4, 128.3, 128.0, 127.5, 125.3, 115.8, 53.6, 46.4, 41.4.

Optical Rotation: $[\alpha]_D^{26}$: –126 (*c* = 1.0, CH_2Cl_2) [95% *ee*].

Chiral HPLC: 95% *ee* (AS-RH 150 × 4.6 mm, MeCN/water = 20/80 → 100/0 over 30 min, 1 ml/min, λ = 210 nm); t_R = 18.49 min (major, **15**), 22.06 min (minor, *ent*-**15**).

8. NMR Spectra

4-Phenyl-3,4-dihydroquinolin-2(1*H*)-one (*rac*-1a)

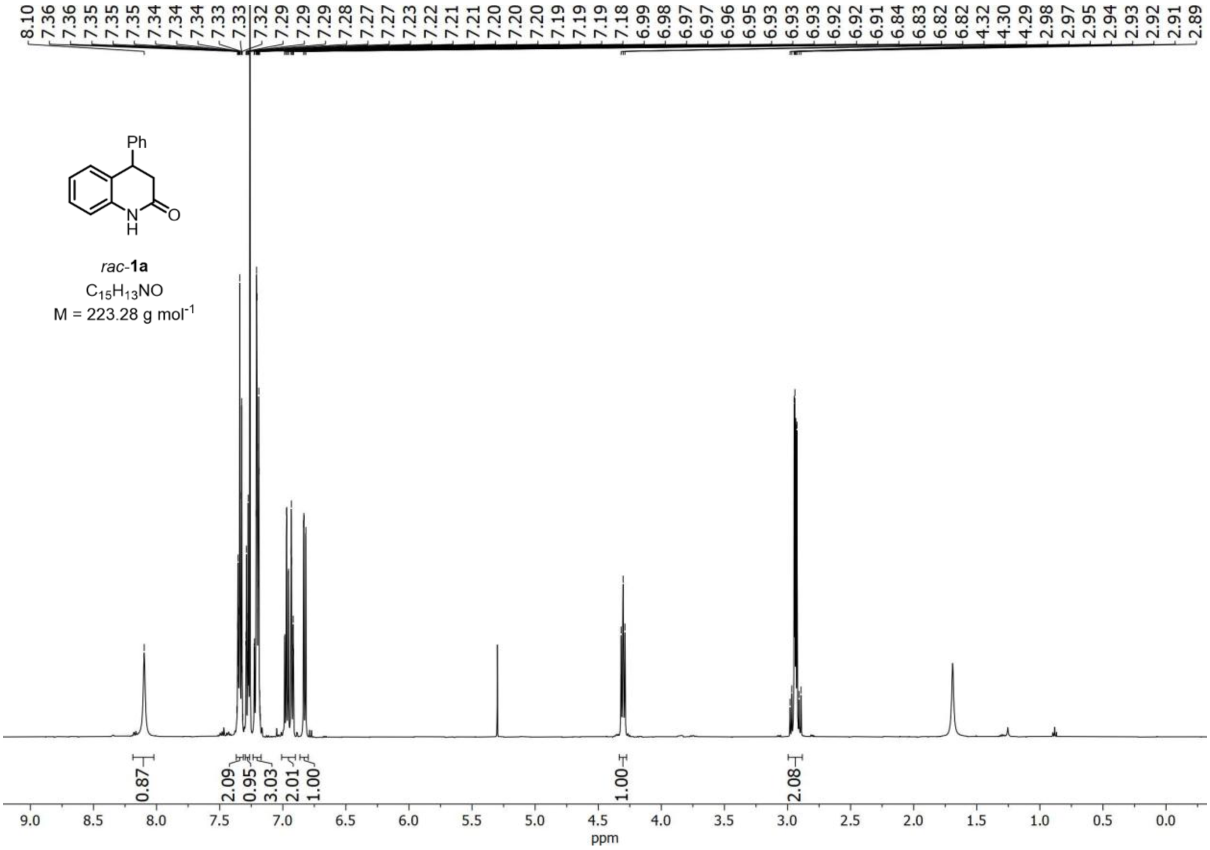

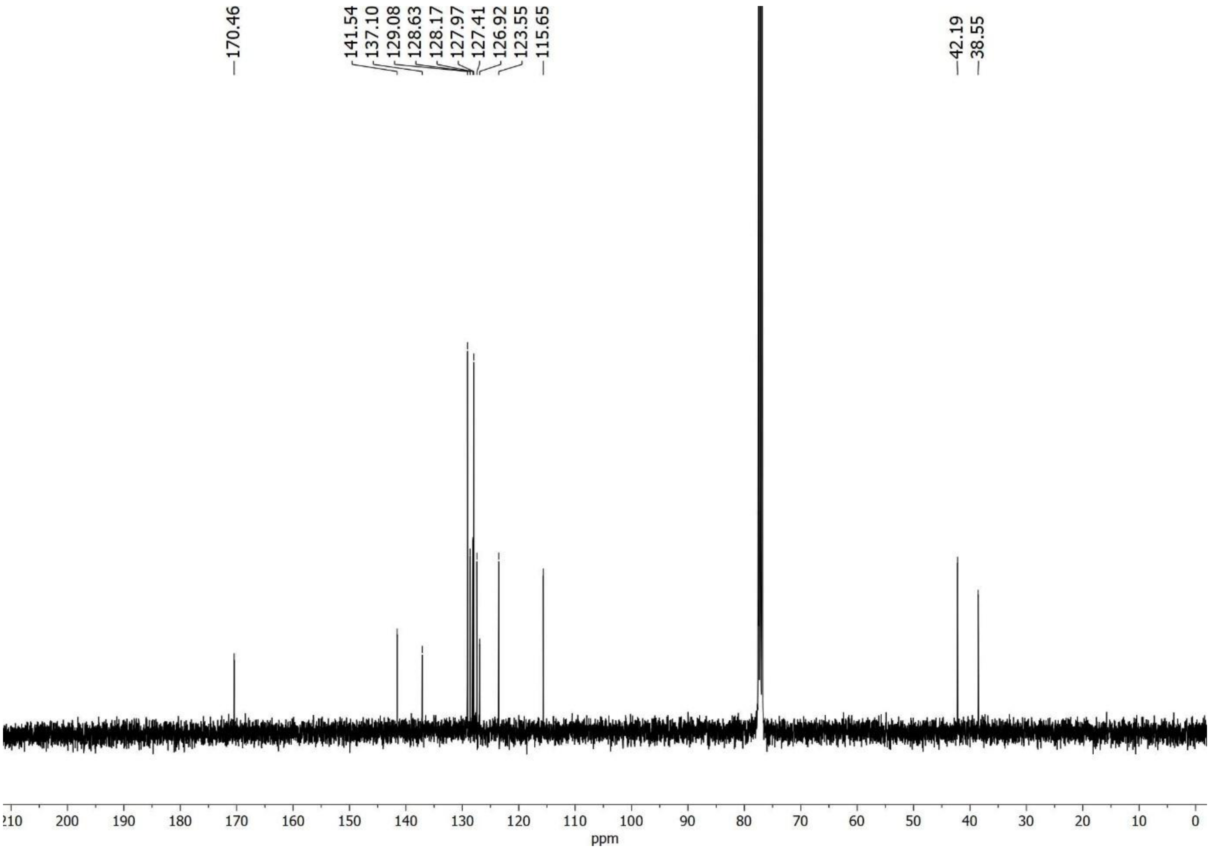

4-(4-Methoxyphenyl)-3,4-dihydroquinolin-2(1*H*)-one (*rac*-1b)

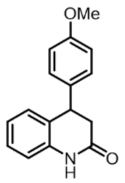

rac-**1b**

$C_{16}H_{15}NO_2$

M = 253.30 g mol^{-1}

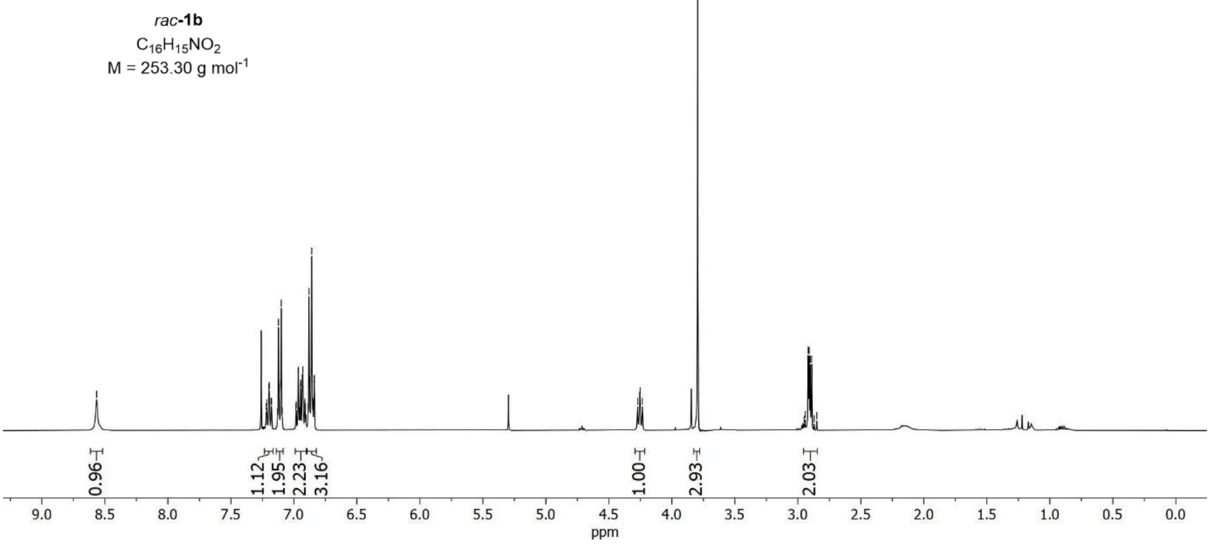

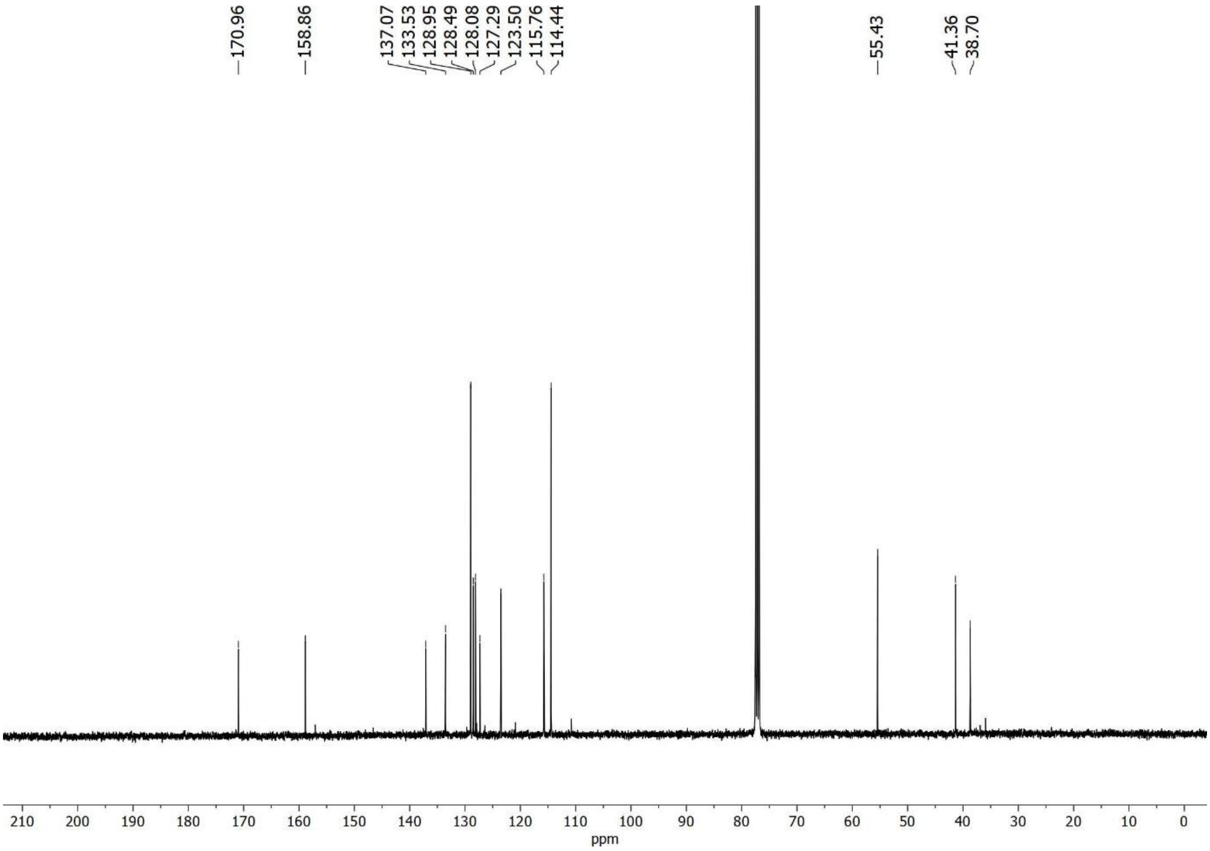

4-(*p*-Tolyl)-3,4-dihydroquinolin-2(1*H*)-one (*rac*-1c)

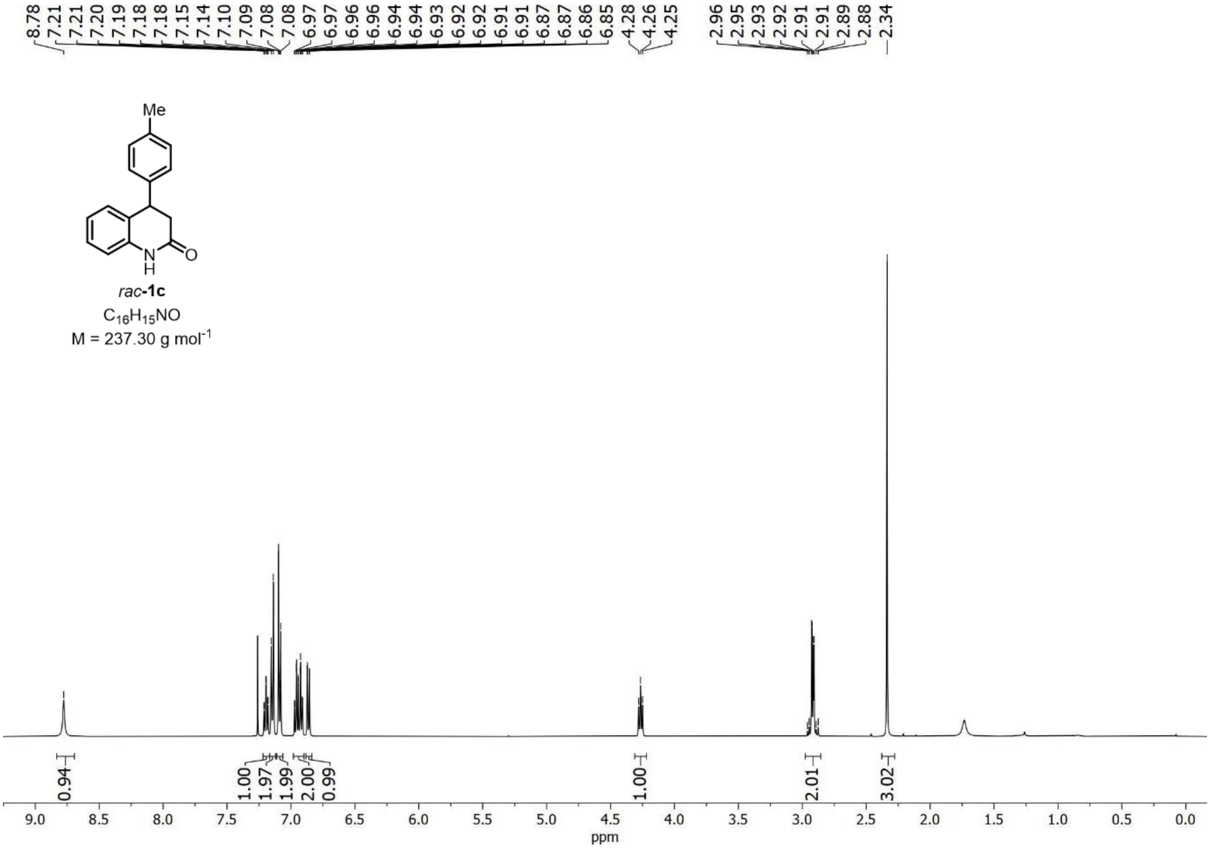

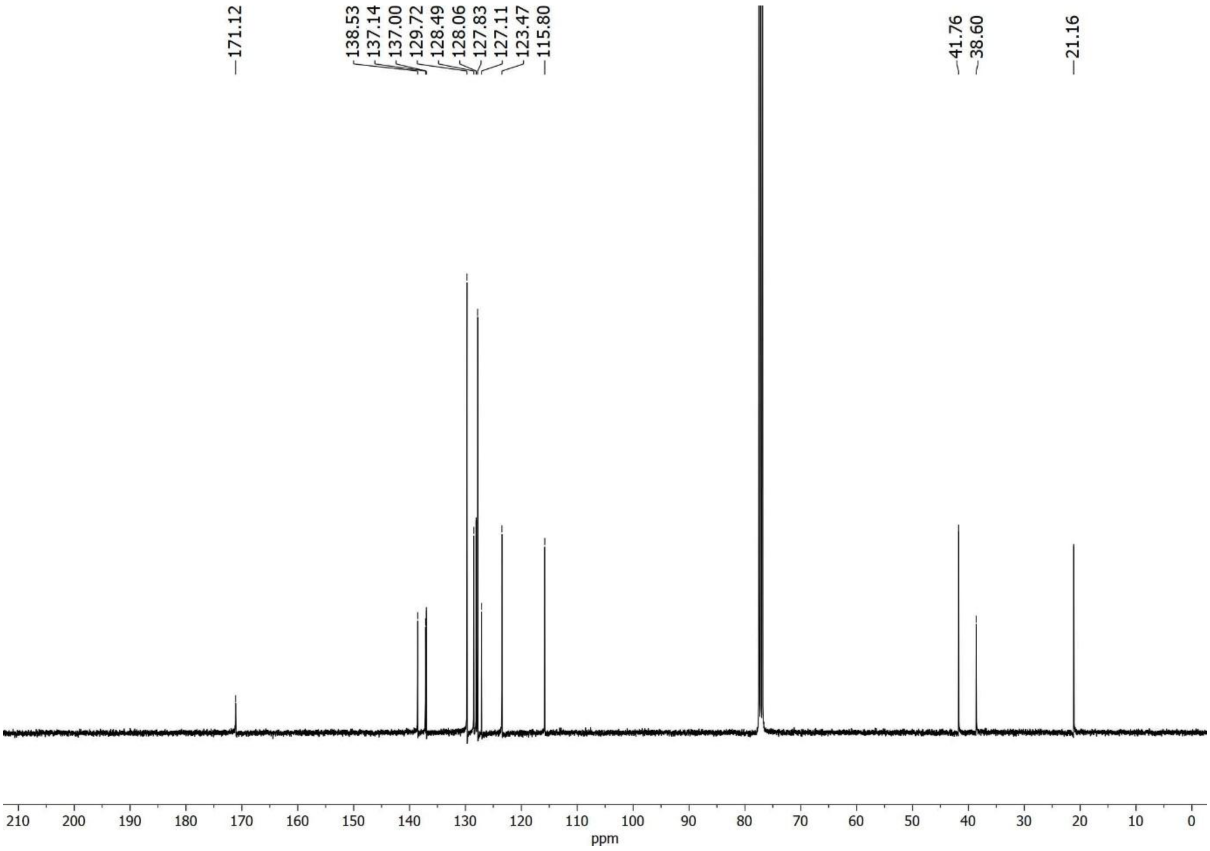

4-(4-Fluorophenyl)-3,4-dihydroquinolin-2(1*H*)-one (*rac*-1d)

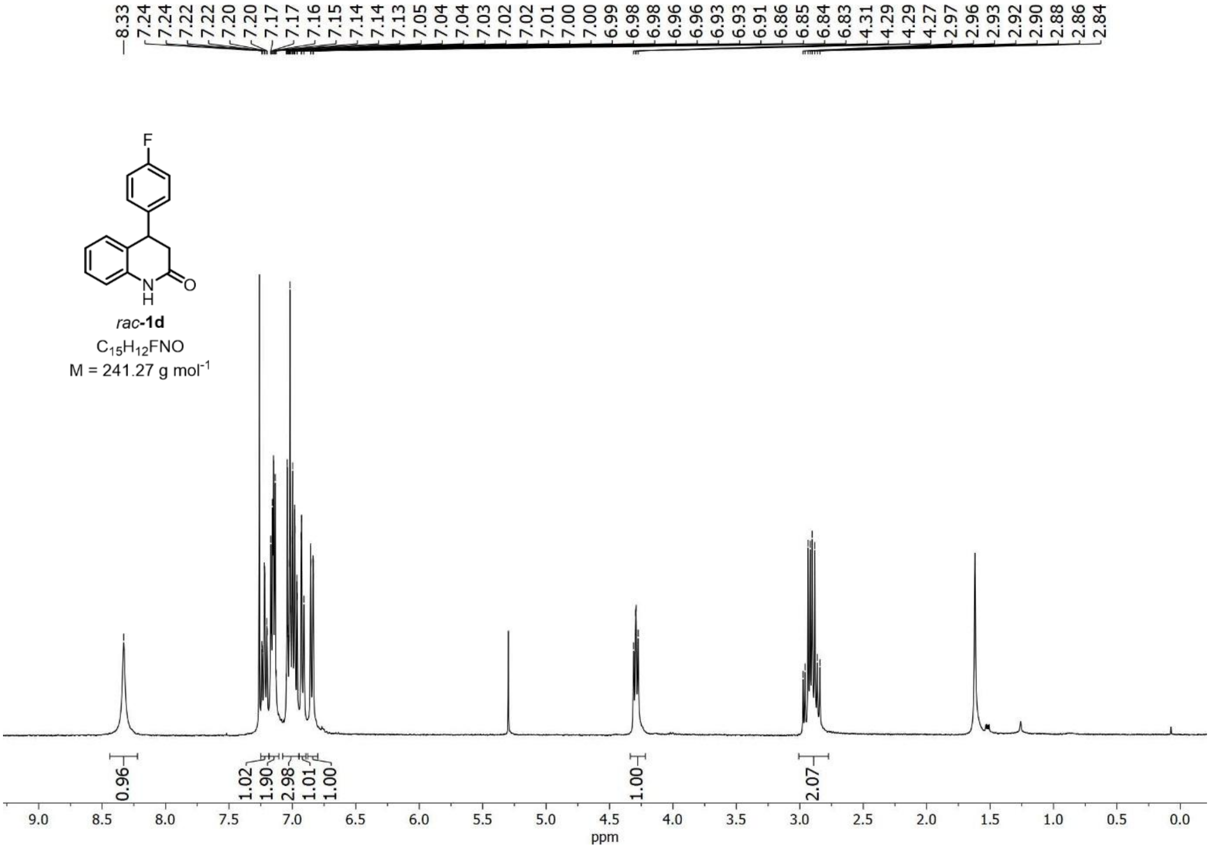

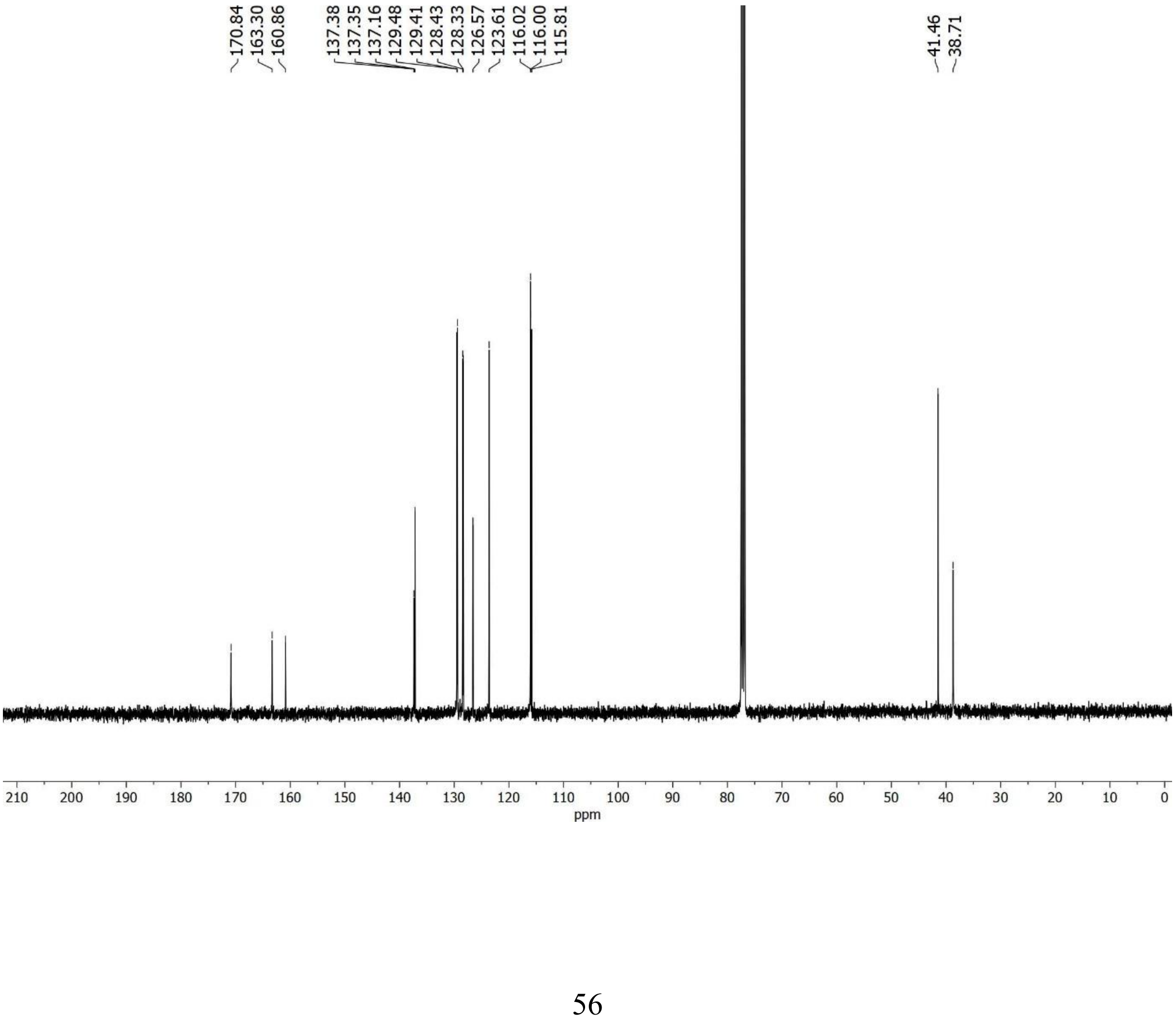

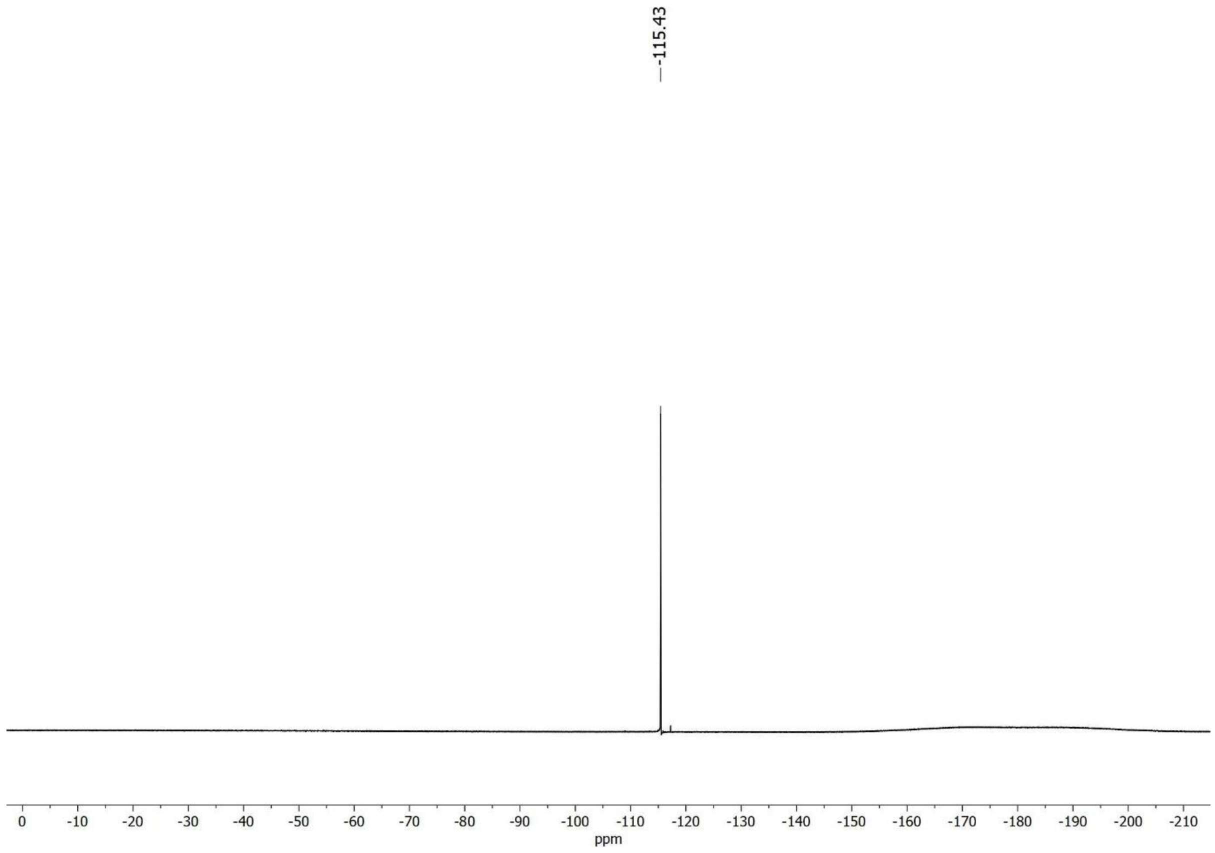

-115.43
0
-10
-20
-30
-40
-50
-60
-70
-80
-90
-100
-110
-120
-130
-140
-150
-160
-170
-180
-190
-200
-210
ppm

4-(4-Chlorophenyl)-3,4-dihydroquinolin-2(1*H*)-one (*rac*-1e)

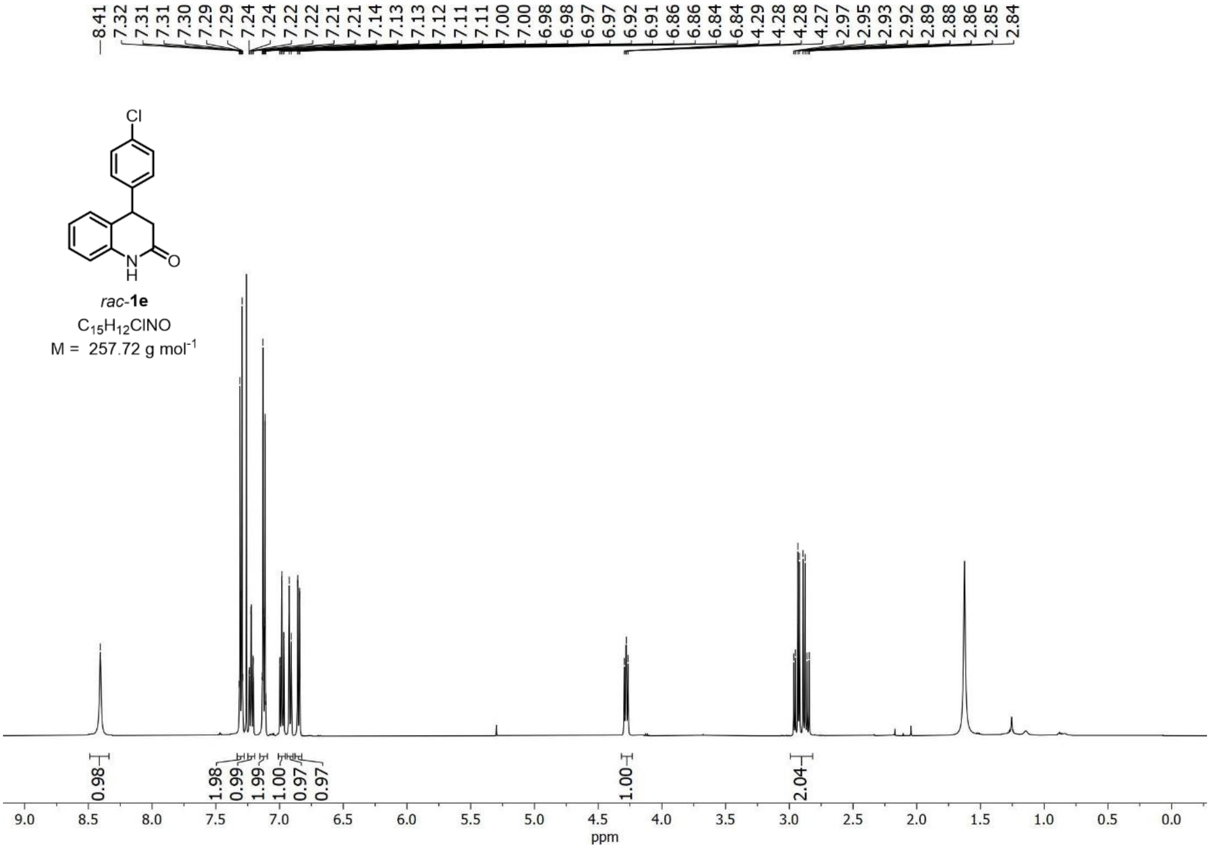

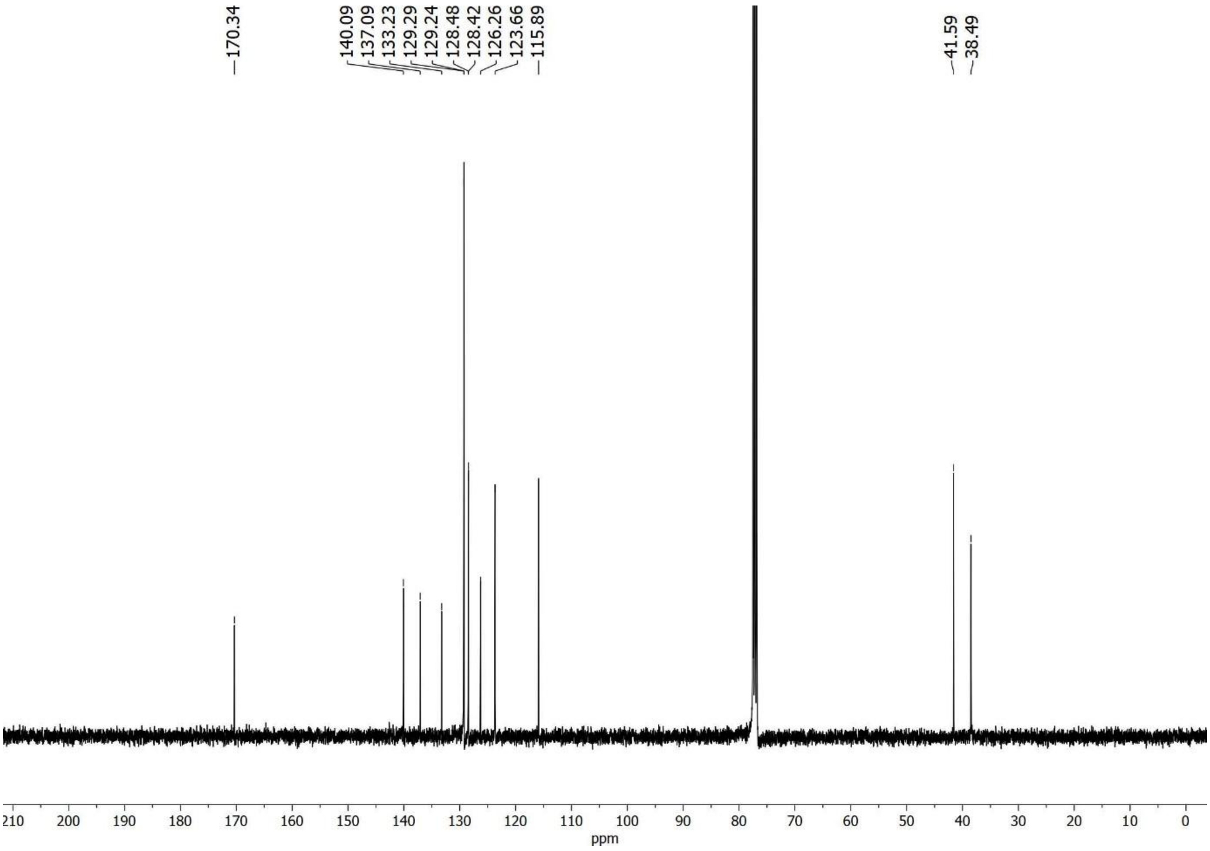

4-(4-Bromophenyl)-3,4-dihydroquinolin-2(1*H*)-one (*rac*-1f)

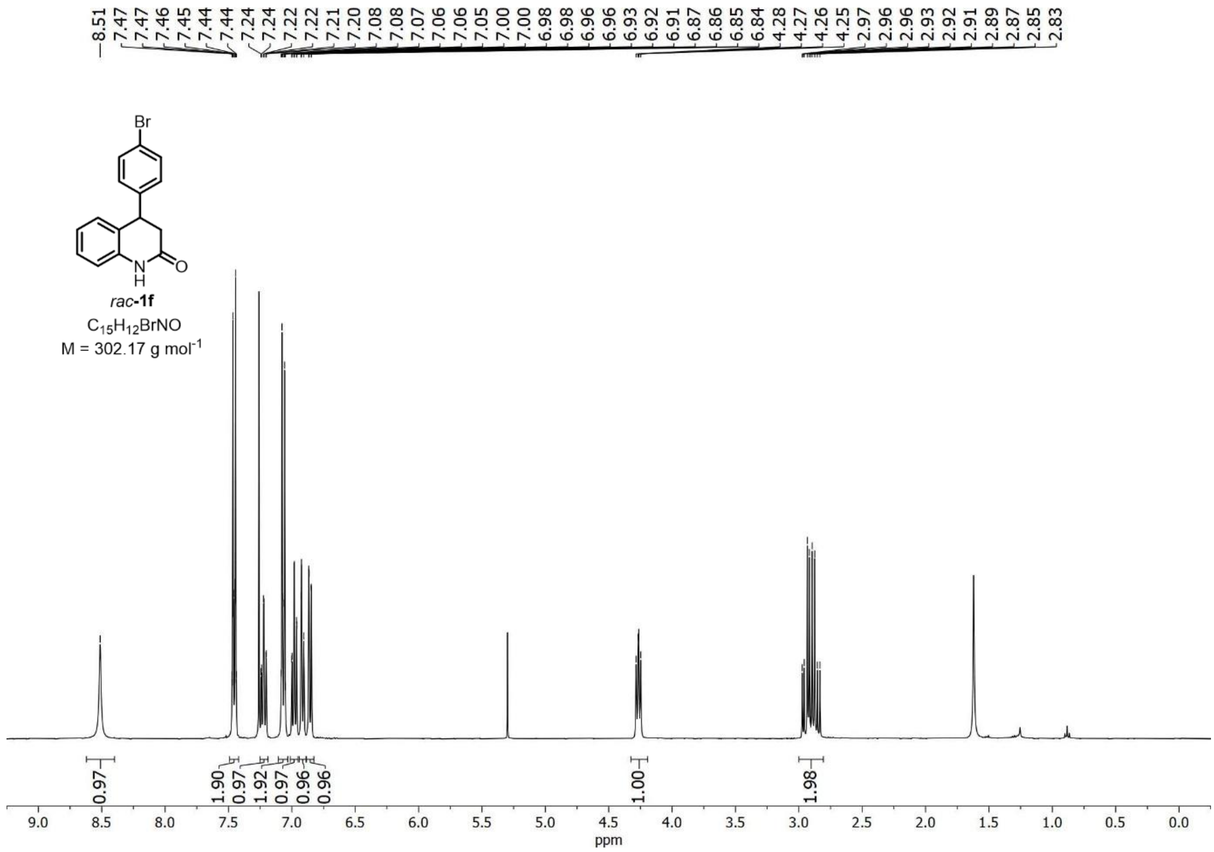

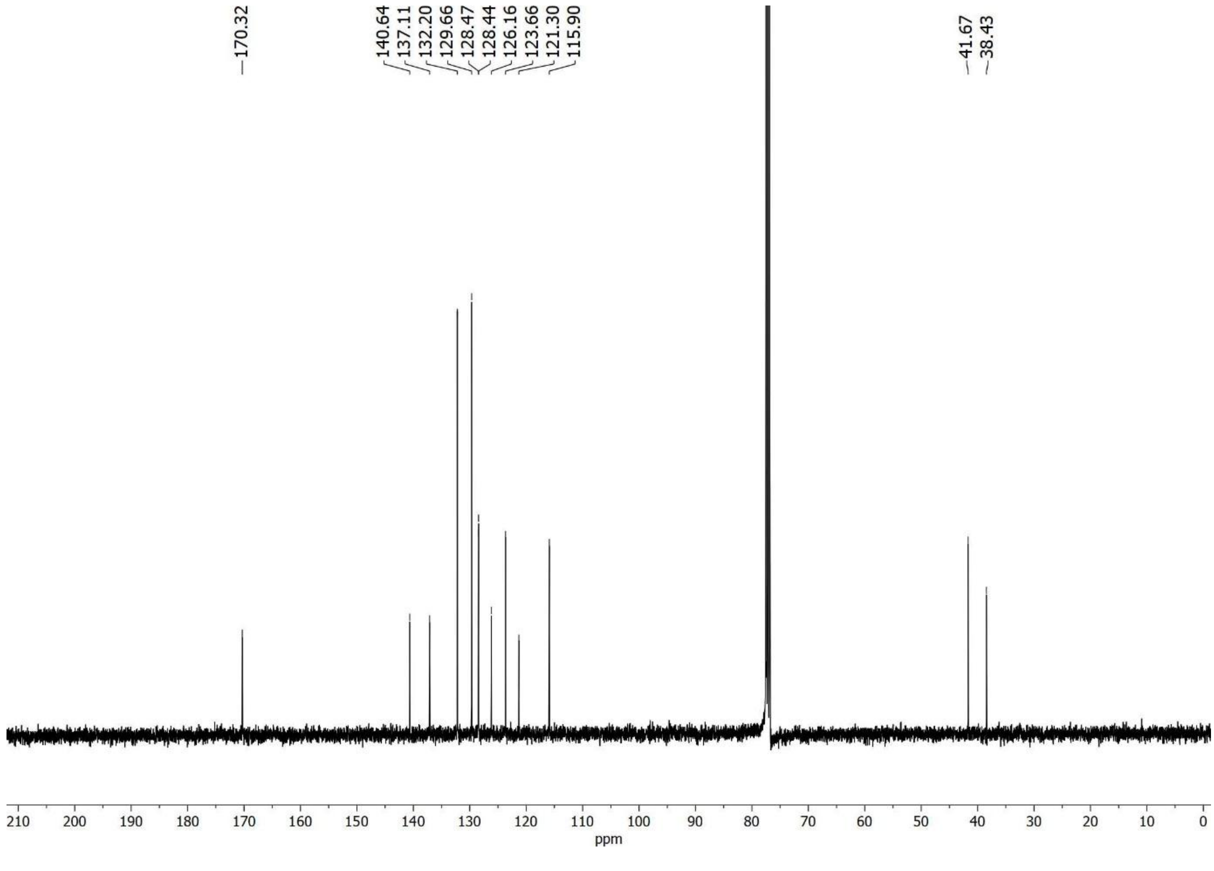

4-(3-Chlorophenyl)-3,4-dihydroquinolin-2(1*H*)-one (*rac*-1g)

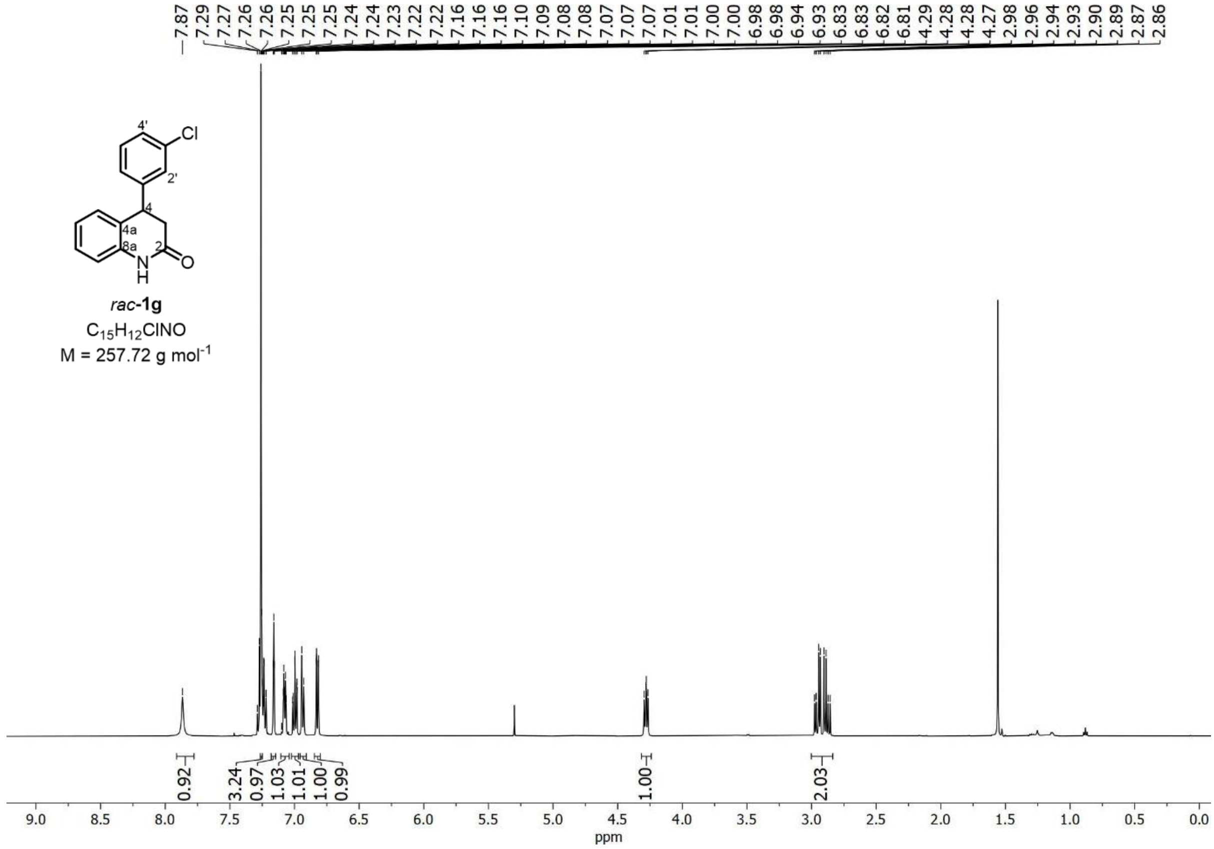

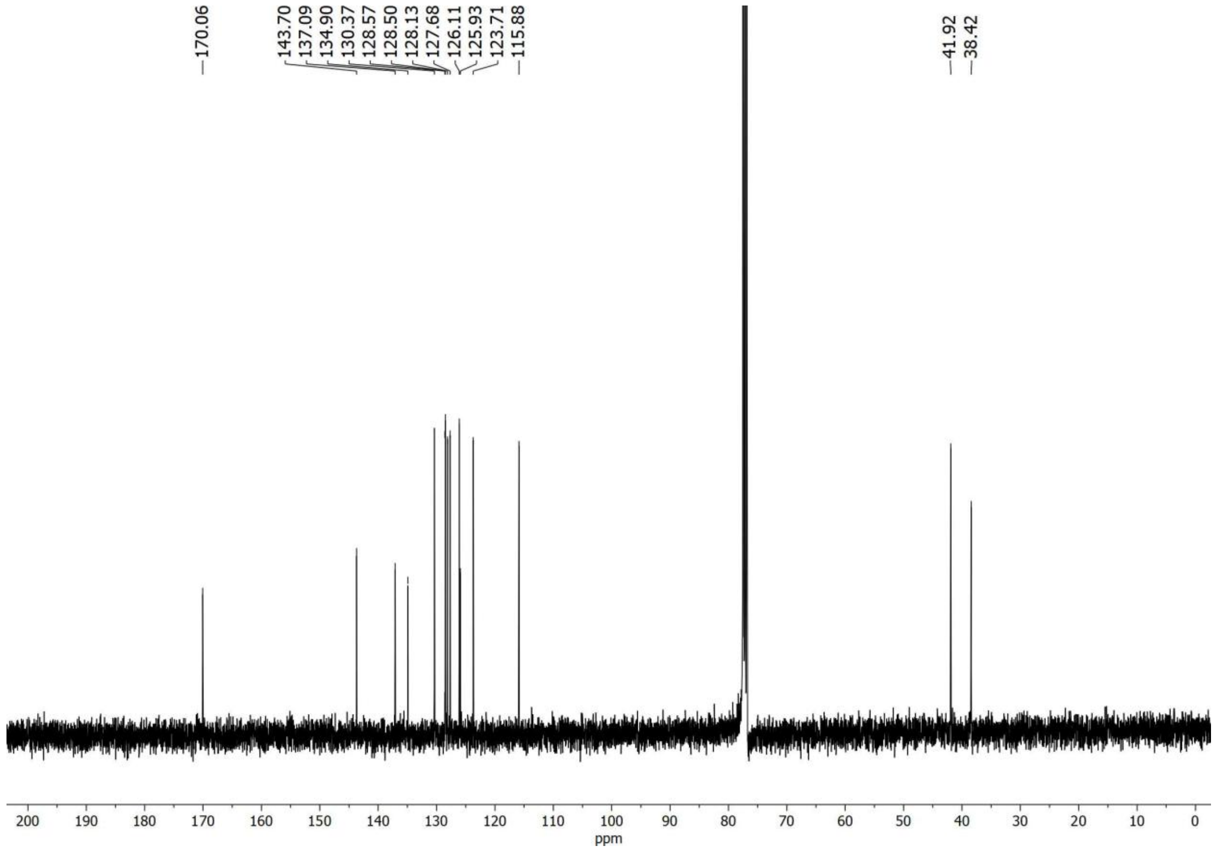

6-Fluoro-4-phenyl-3,4-dihydroquinolin-2(1*H*)-one (*rac*-1h)

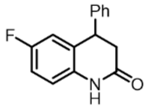

rac-**1h**
$C_{15}H_{12}FNO$
M = 241.27 g mol^{-1}

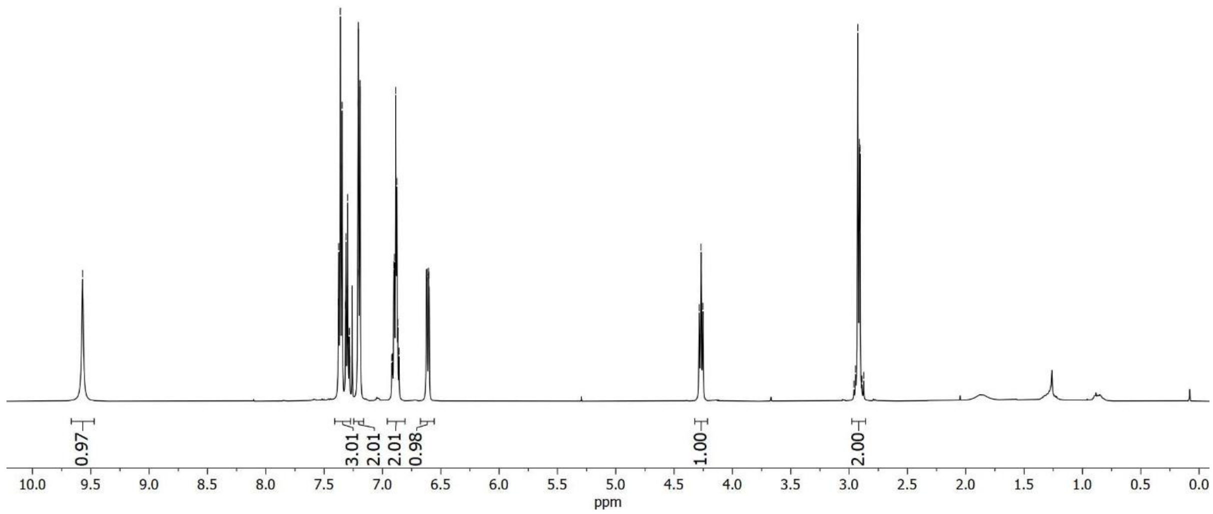

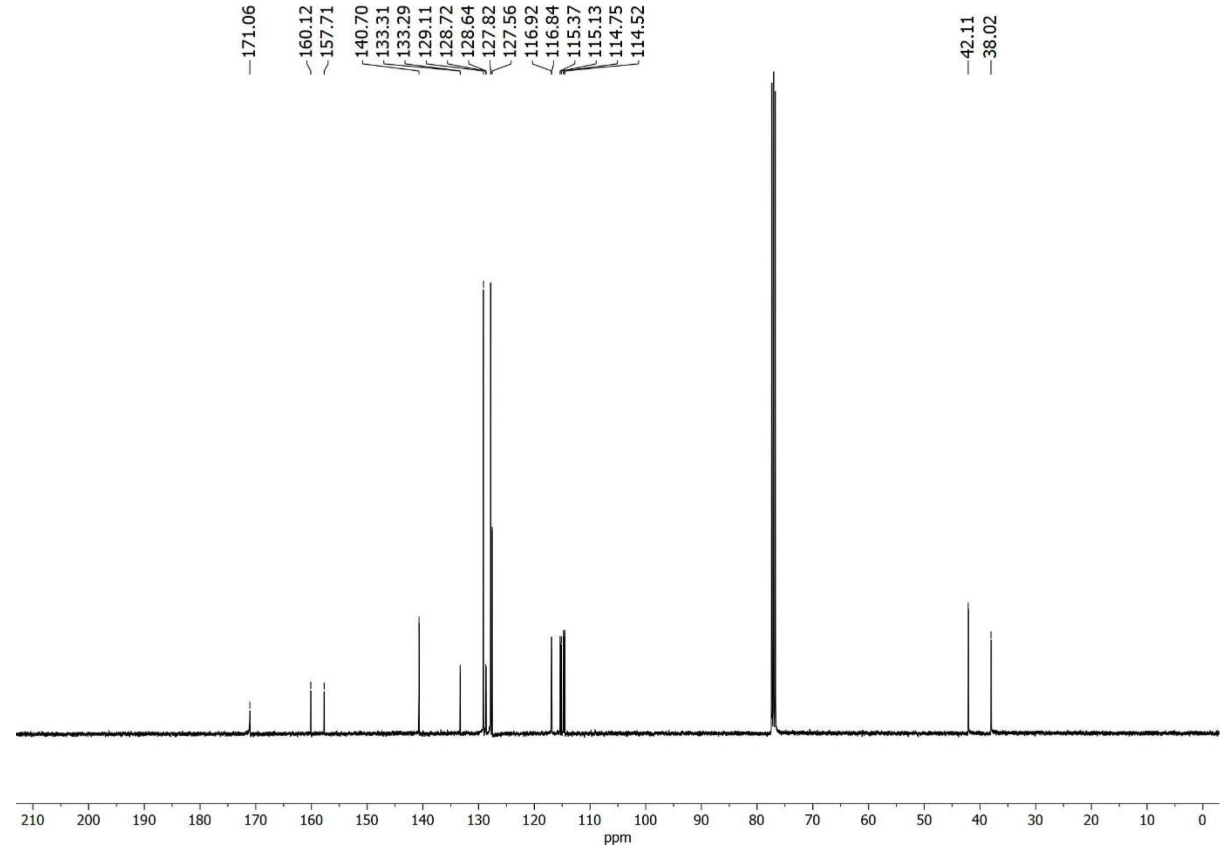

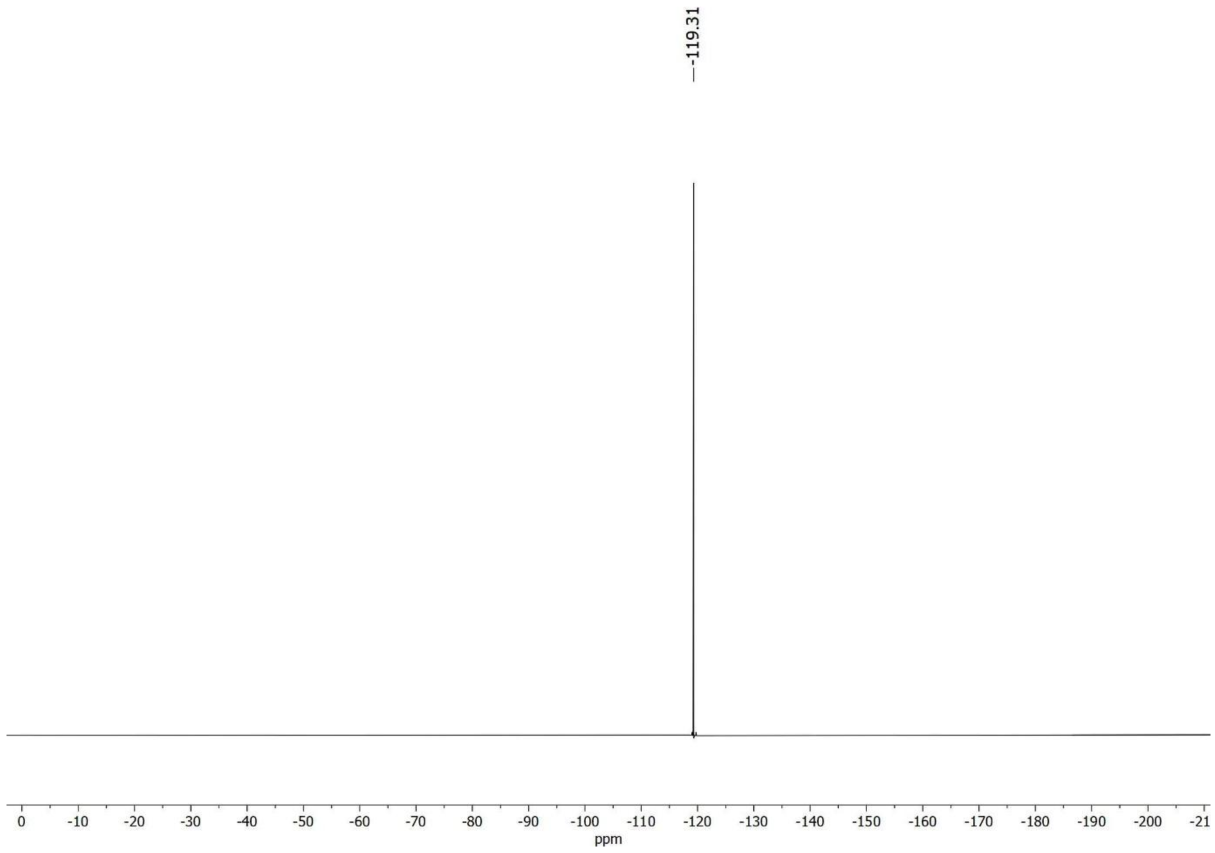
-119.31
0
-10
-20
-30
-40
-50
-60
-70
-80
-90
-100
-110
-120
-130
-140
-150
-160
-170
-180
-190
-200
-21
ppm

7-Methyl-4-phenyl-3,4-dihydroquinolin-2(1H)-one (*rac*-1i)

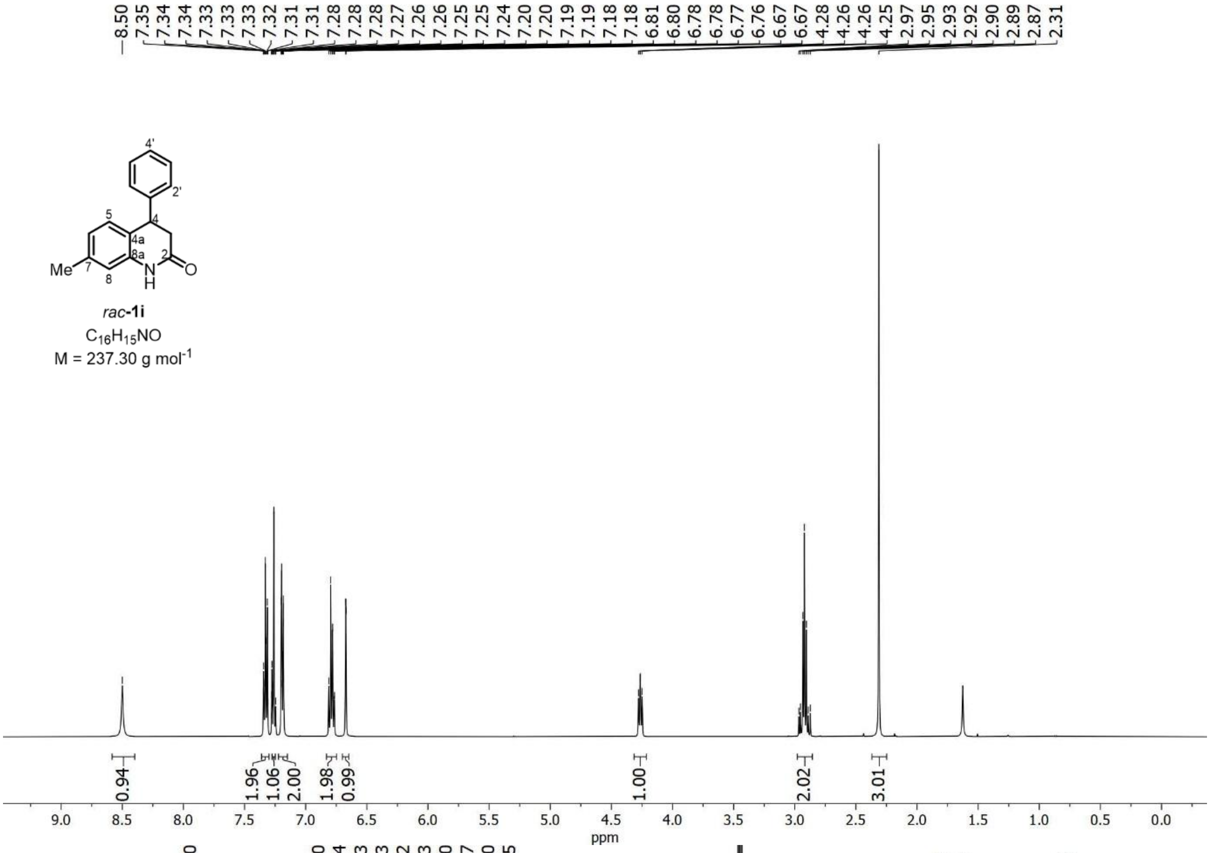

rac-**1i**
$C_{16}H_{15}NO$
M = 237.30 g mol^{-1}

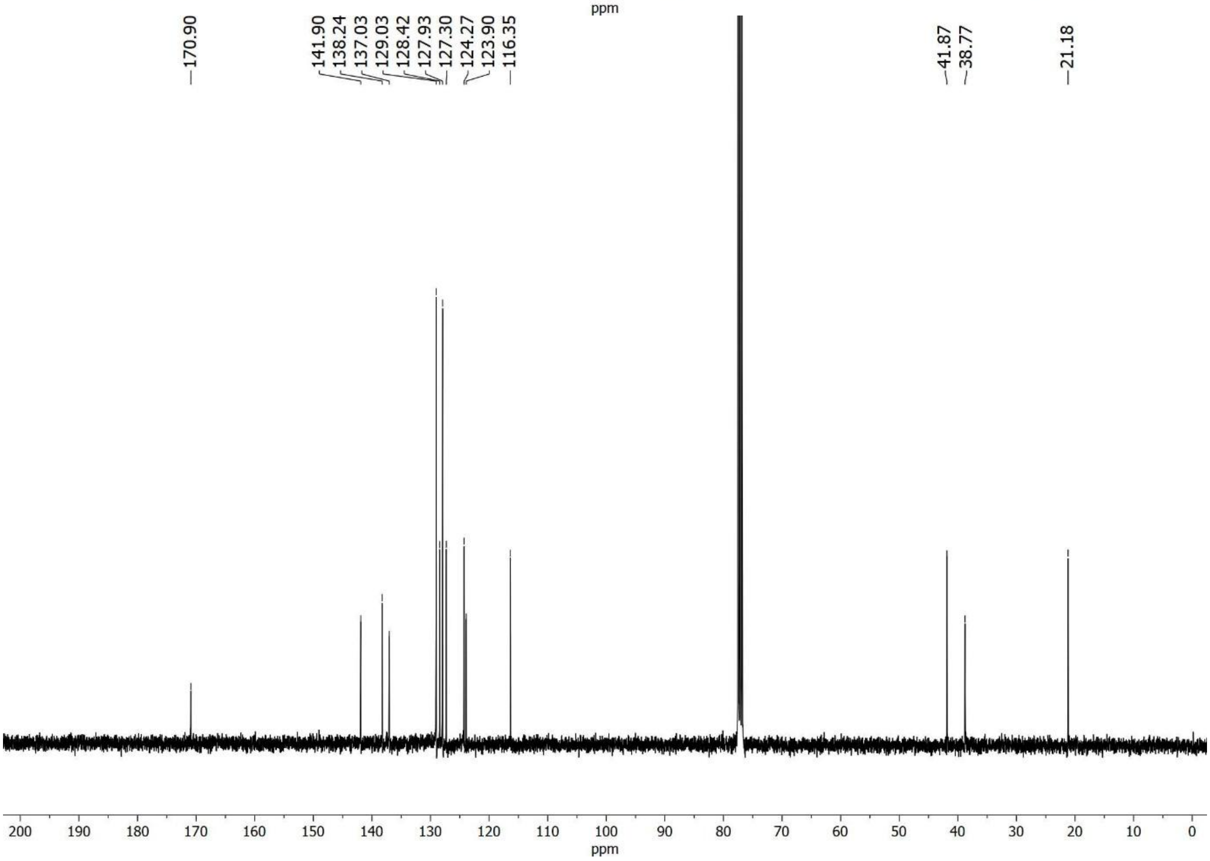

7-Methoxy-4-phenyl-3,4-dihydroquinolin-2(1H)-one (*rac*-1j)

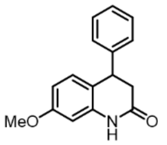

rac-**1j**
$C_{16}H_{15}NO_2$
M = 253.30 g mol^{-1}

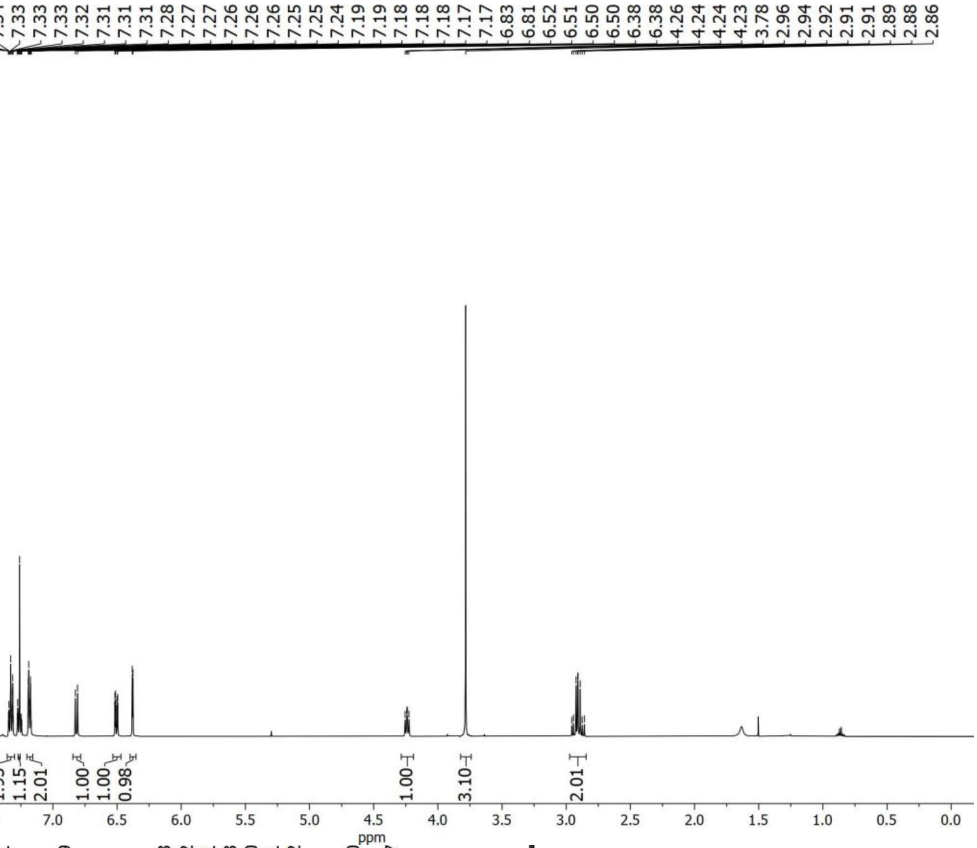

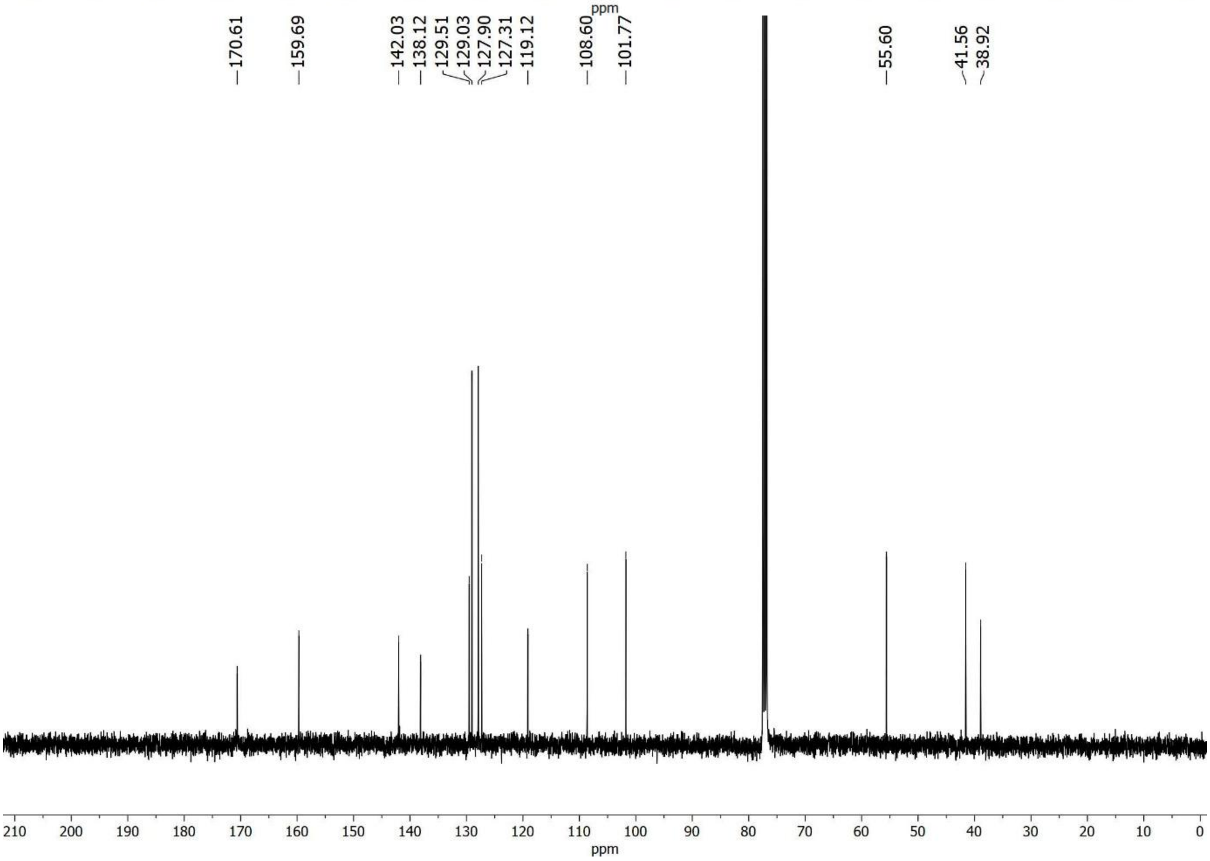

7-Chloro-4-phenyl-3,4-dihydroquinolin-2(1*H*)-one (*rac*-1k)

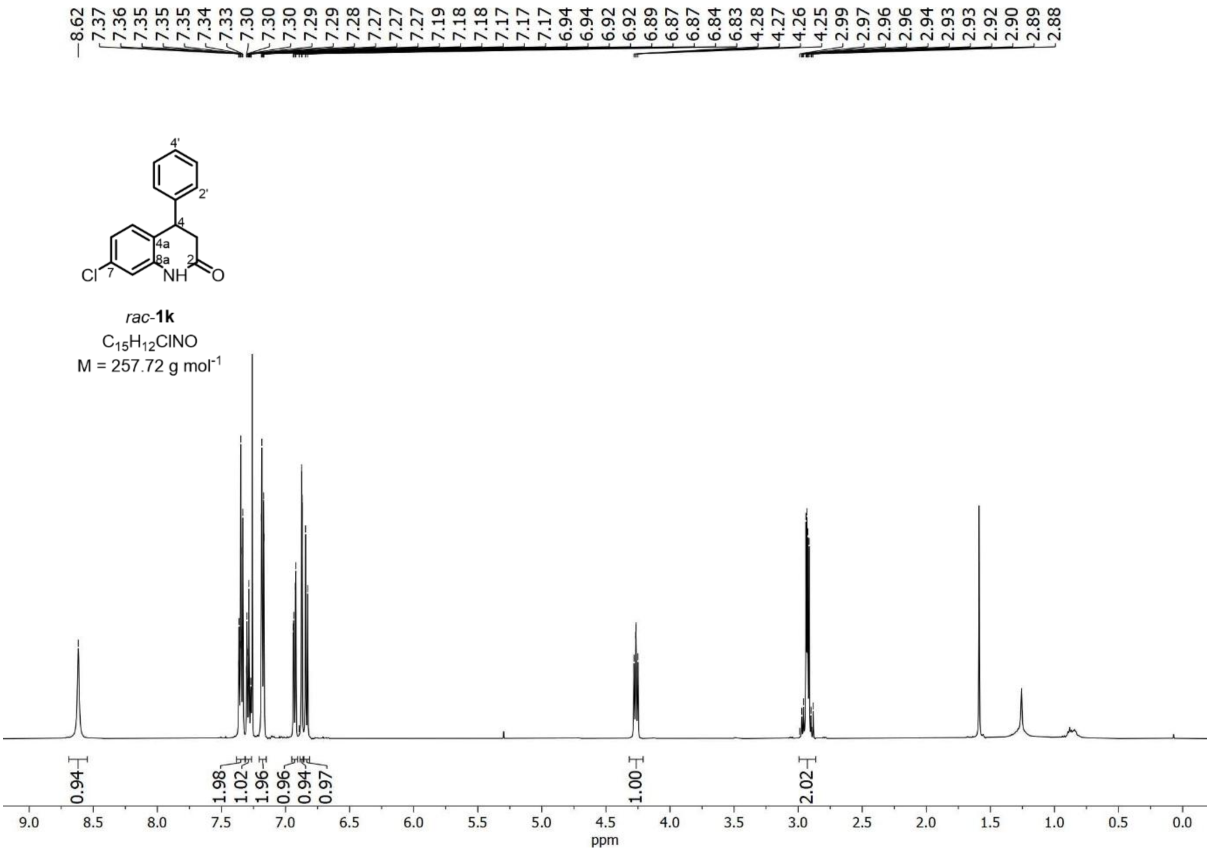

rac-**1k**
$C_{15}H_{12}ClNO$
M = 257.72 g mol^{-1}

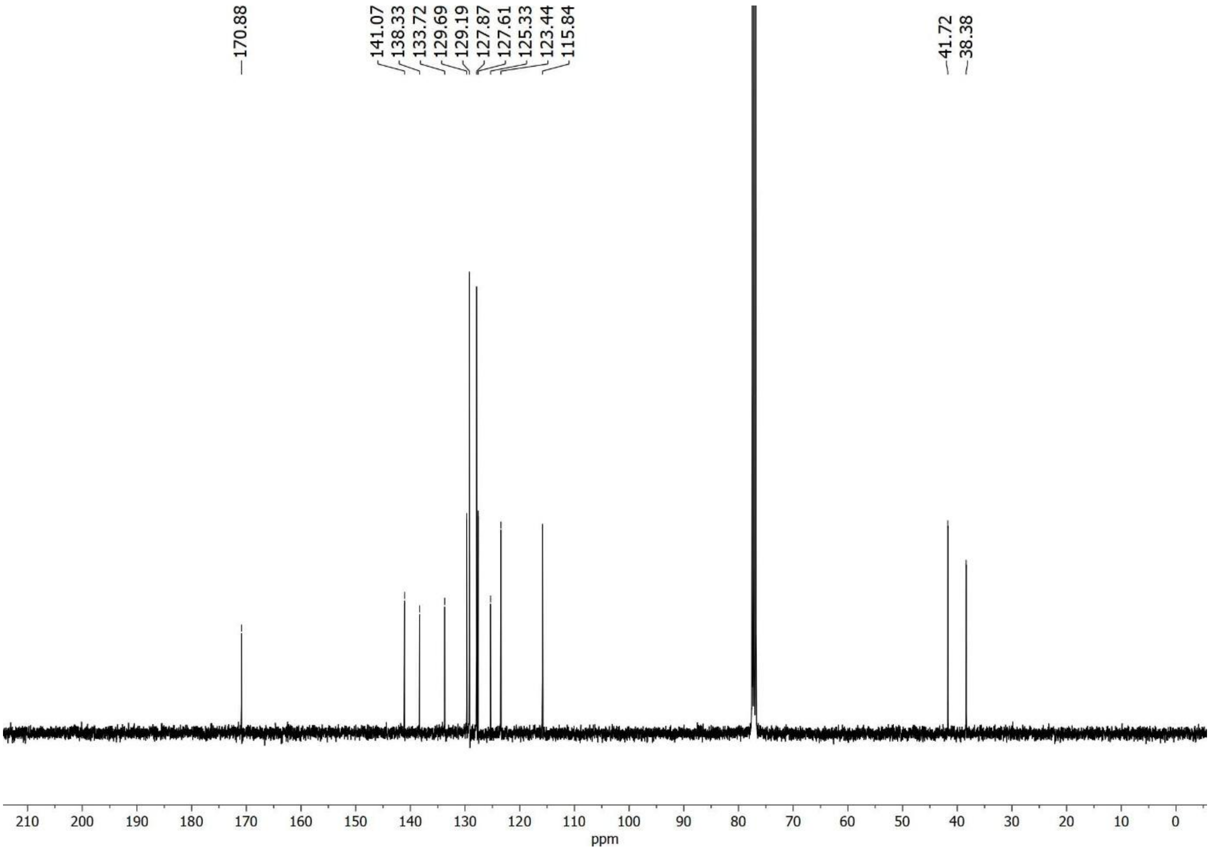

4-Methyl-3,4-dihydroquinolin-2(1*H*)-one (*rac*-1l)

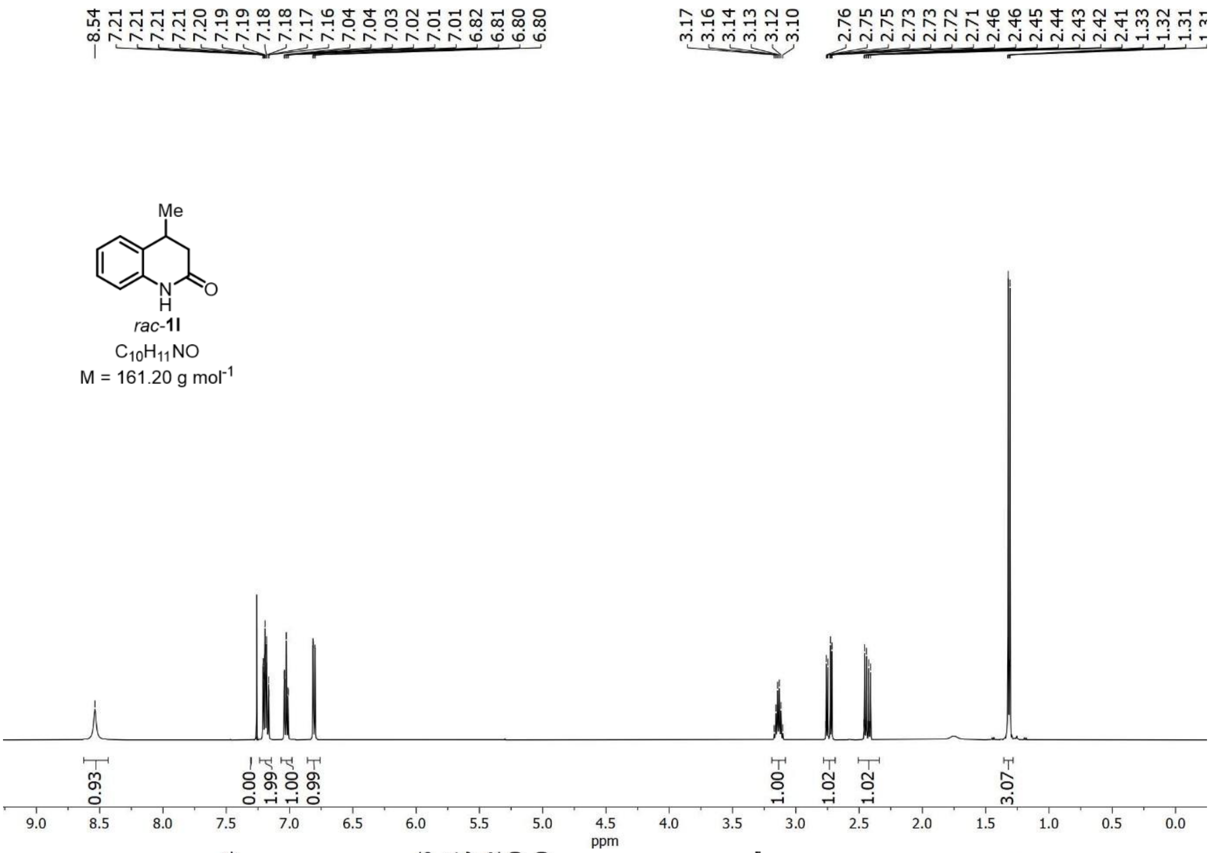

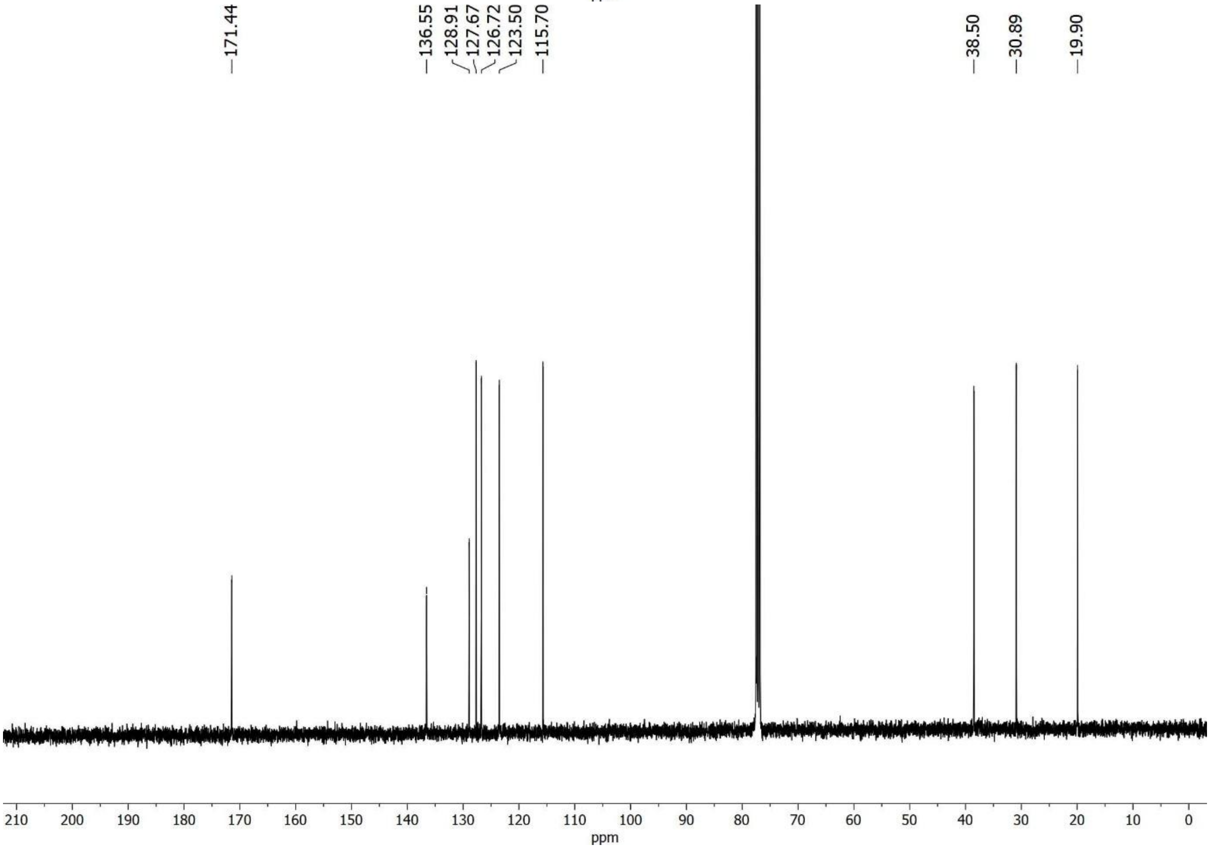

4-Ethyl-3,4-dihydroquinolin-2(1*H*)-one (*rac*-1m)

rac-**1m**

$C_{11}H_{13}NO$

M = 175.23 g mol^{-1}

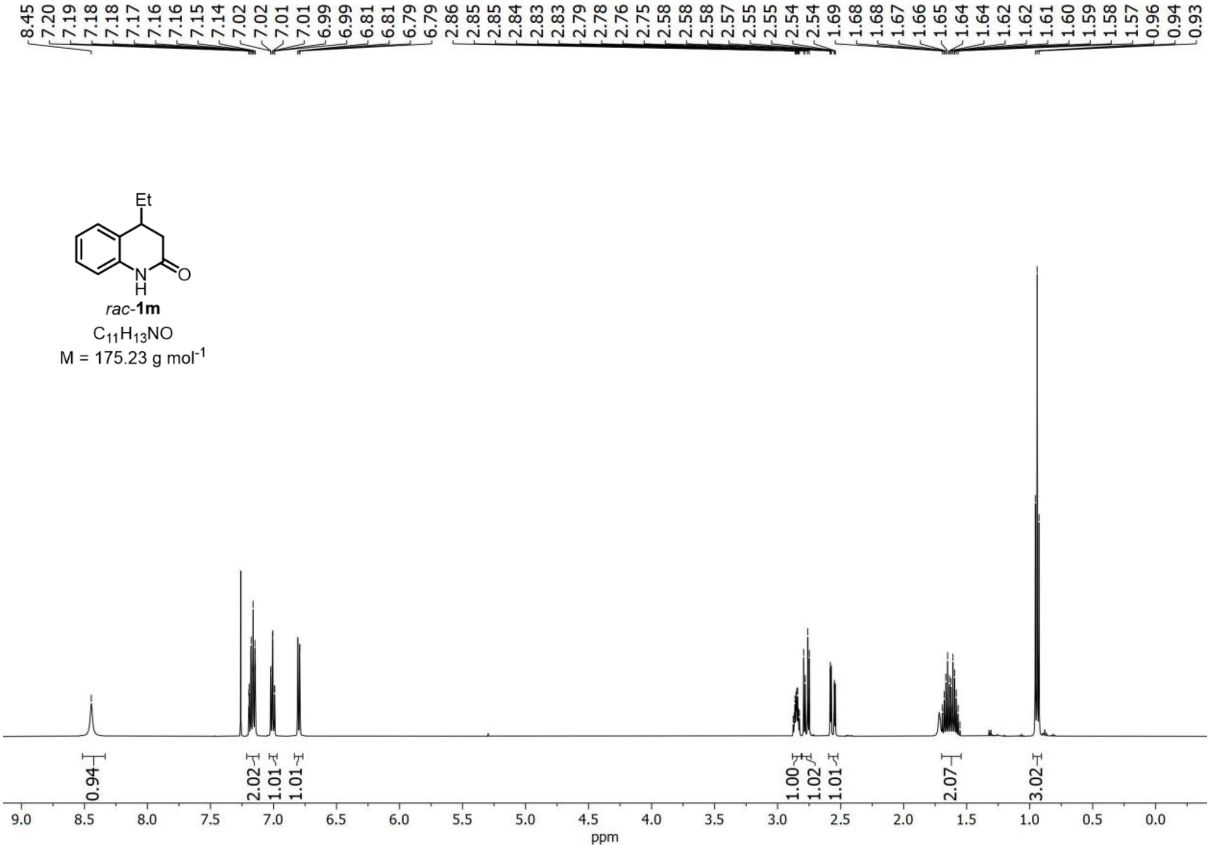

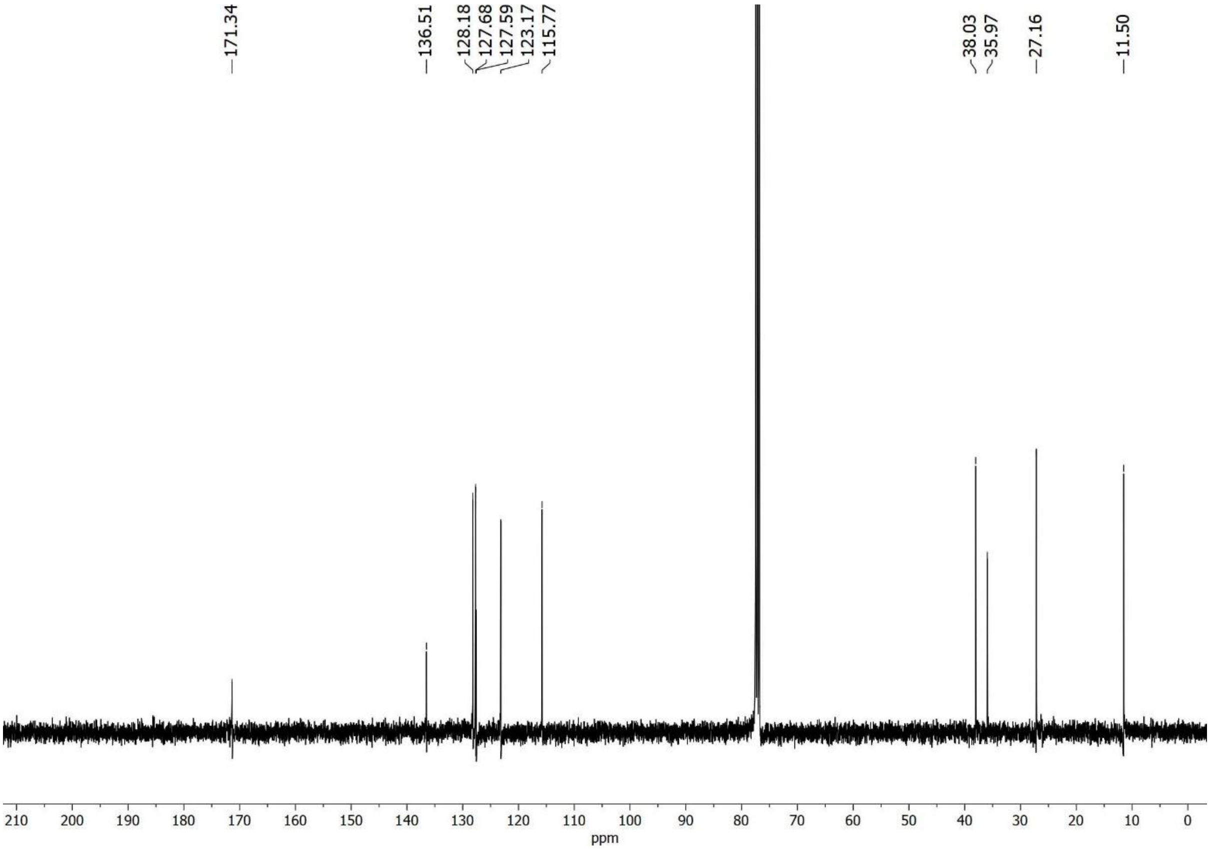

4-Propyl-3,4-dihydroquinolin-2(1*H*)-one (*rac*-1n)

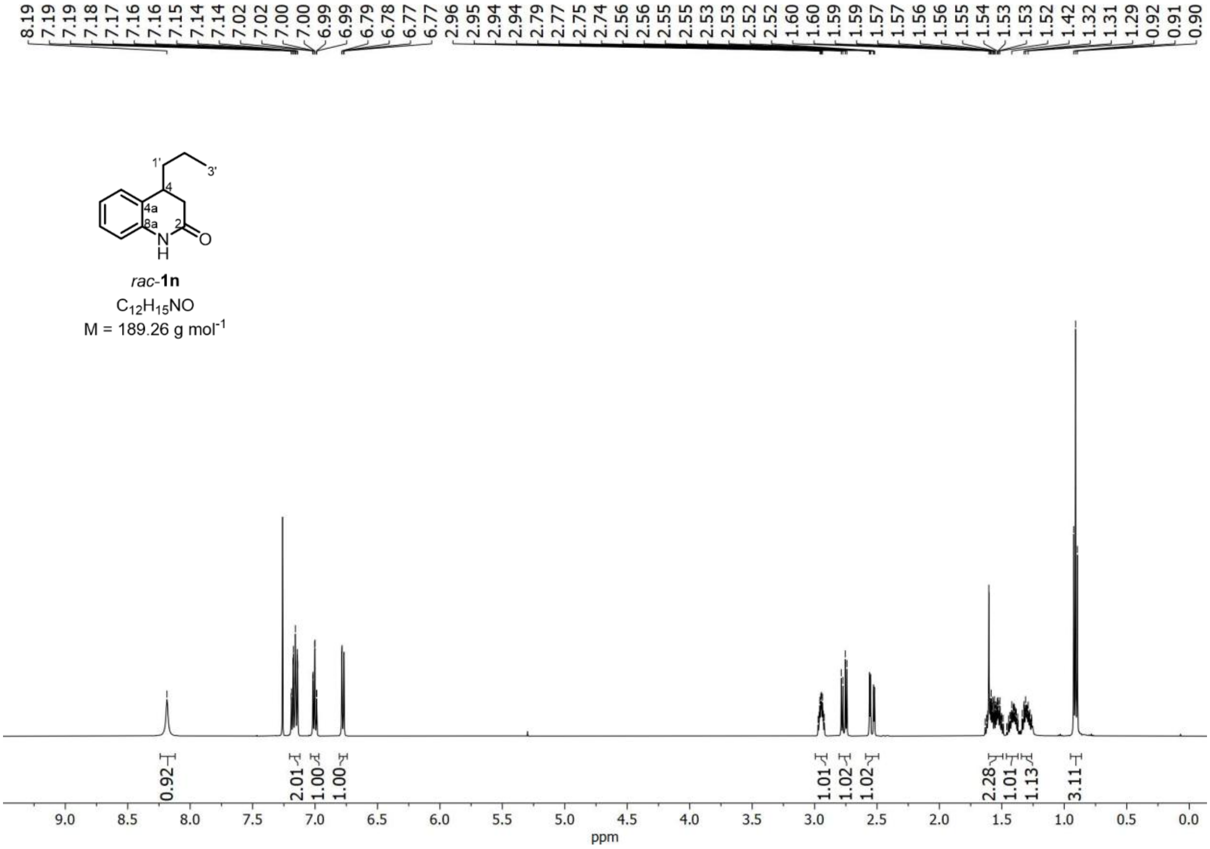

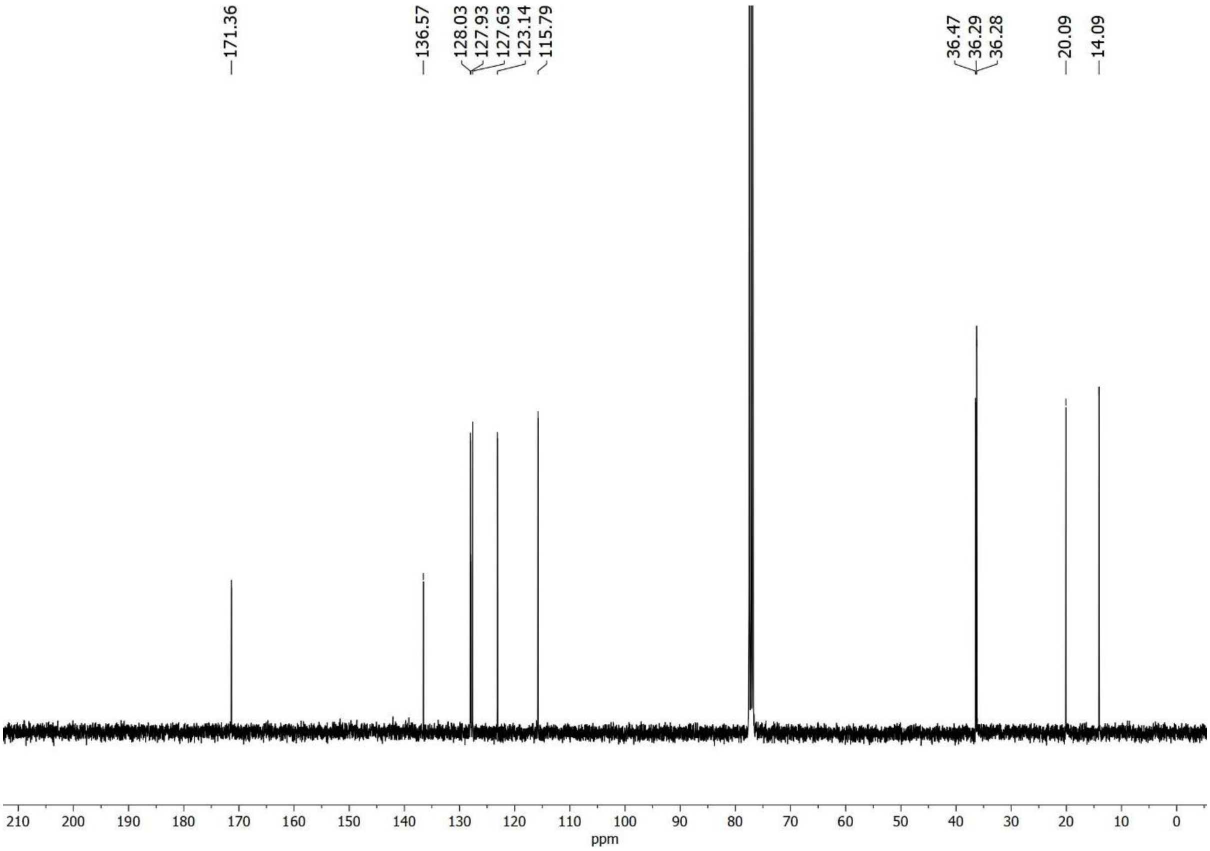

4-Cyclopropyl-3,4-dihydroquinolin-2(1*H*)-one (*rac*-1o)

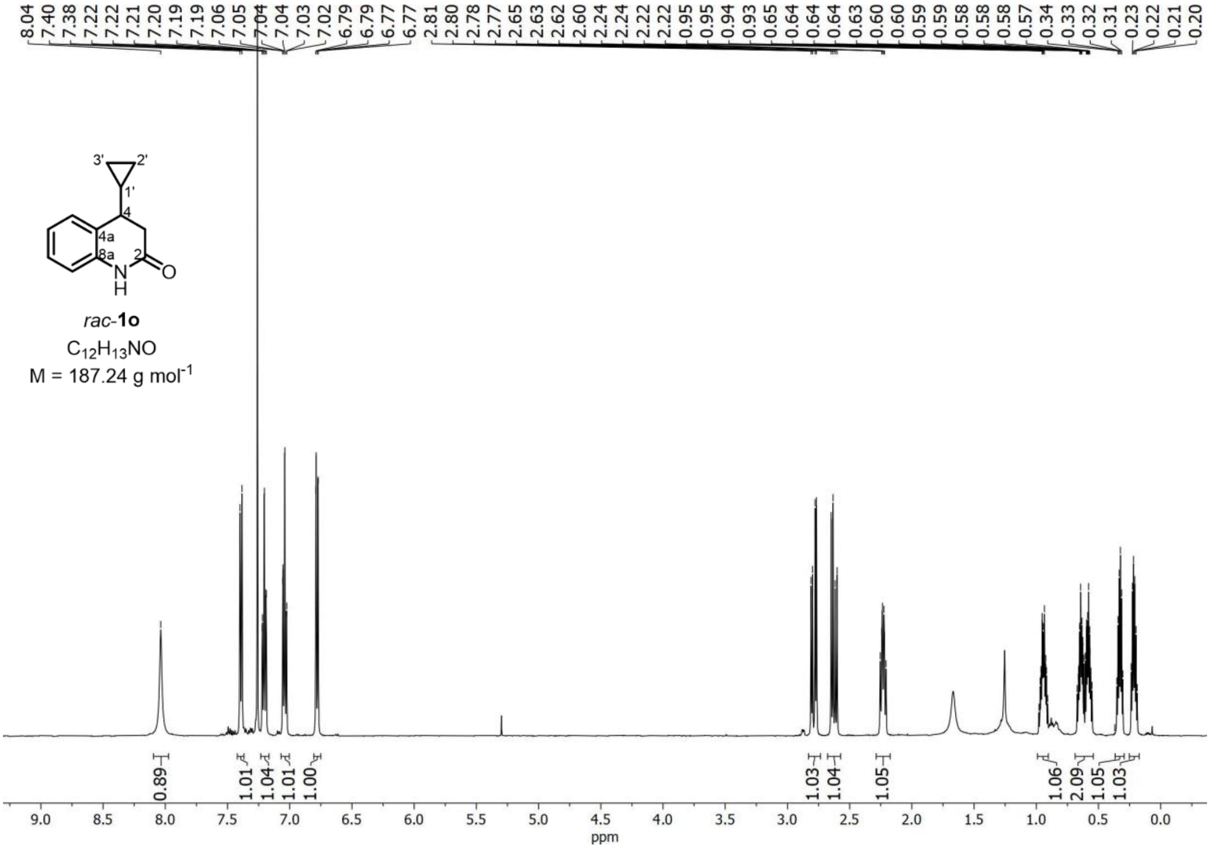

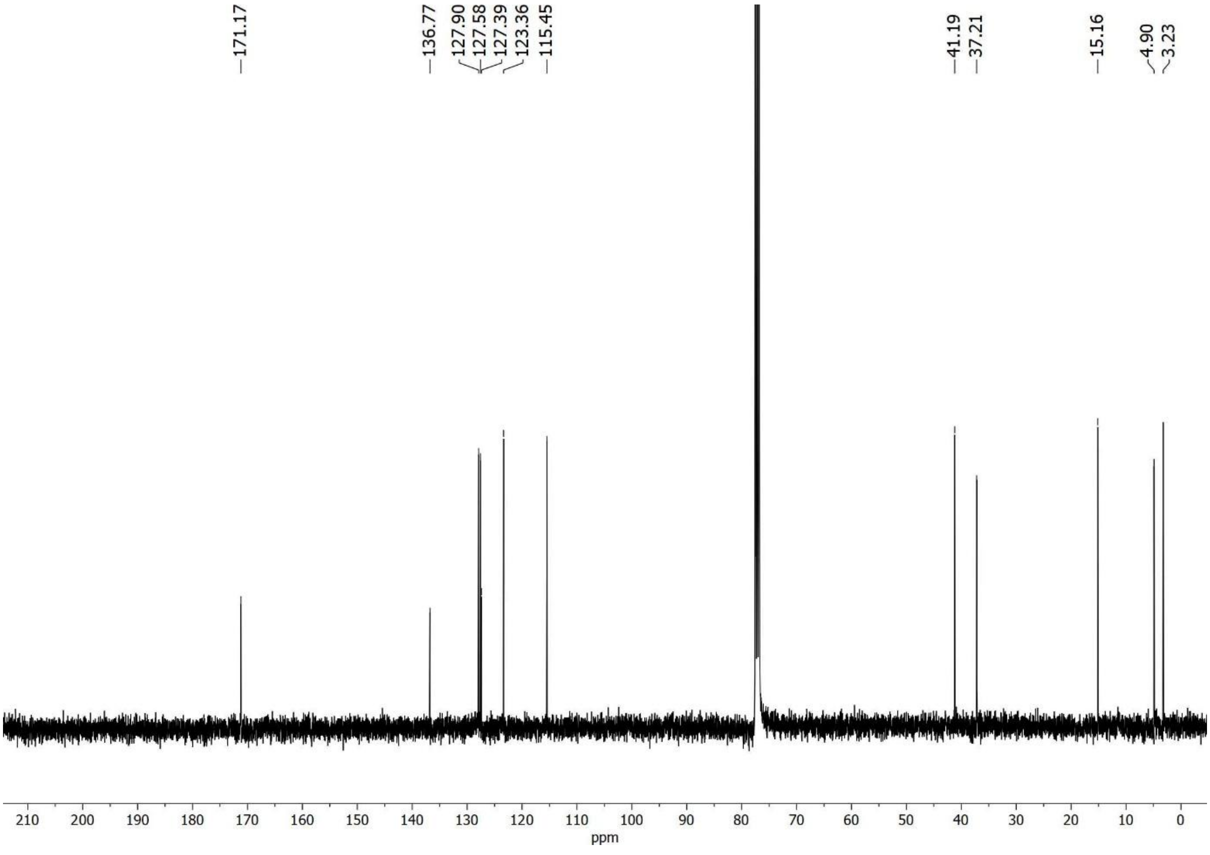

4-Isopropyl-3,4-dihydroquinolin-2(1*H*)-one (*rac*-1p)

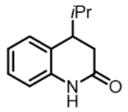

rac-**1p**
$C_{12}H_{15}NO$
M = 189.26 g mol^{-1}

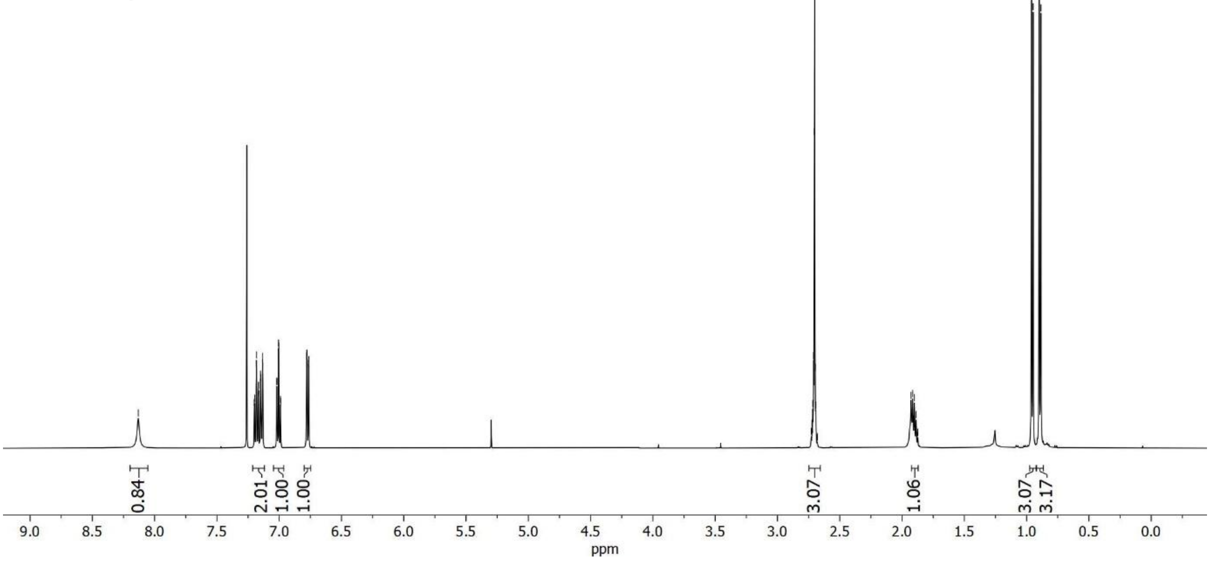

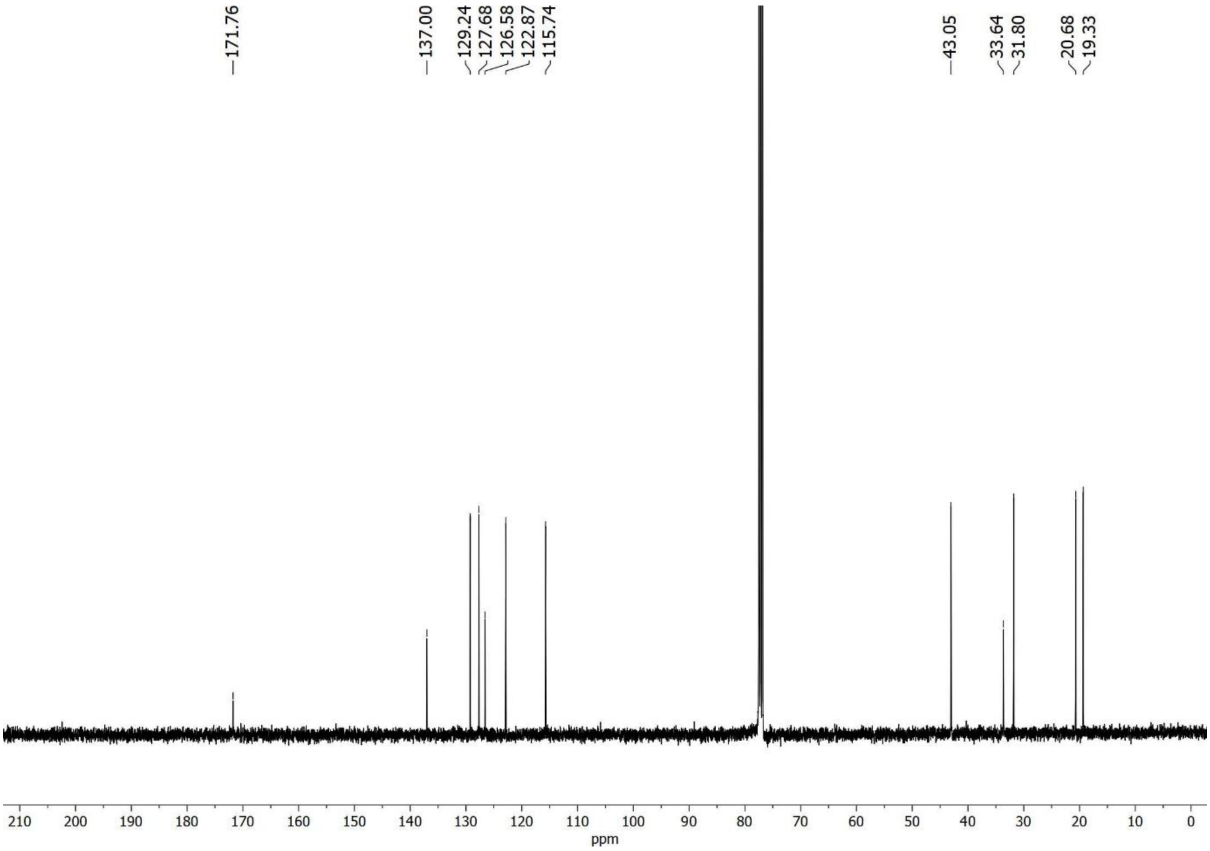

4-(4,4,4-Trifluorobutyl)-3,4-dihydroquinolin-2(1*H*)-one (*rac*-1q)

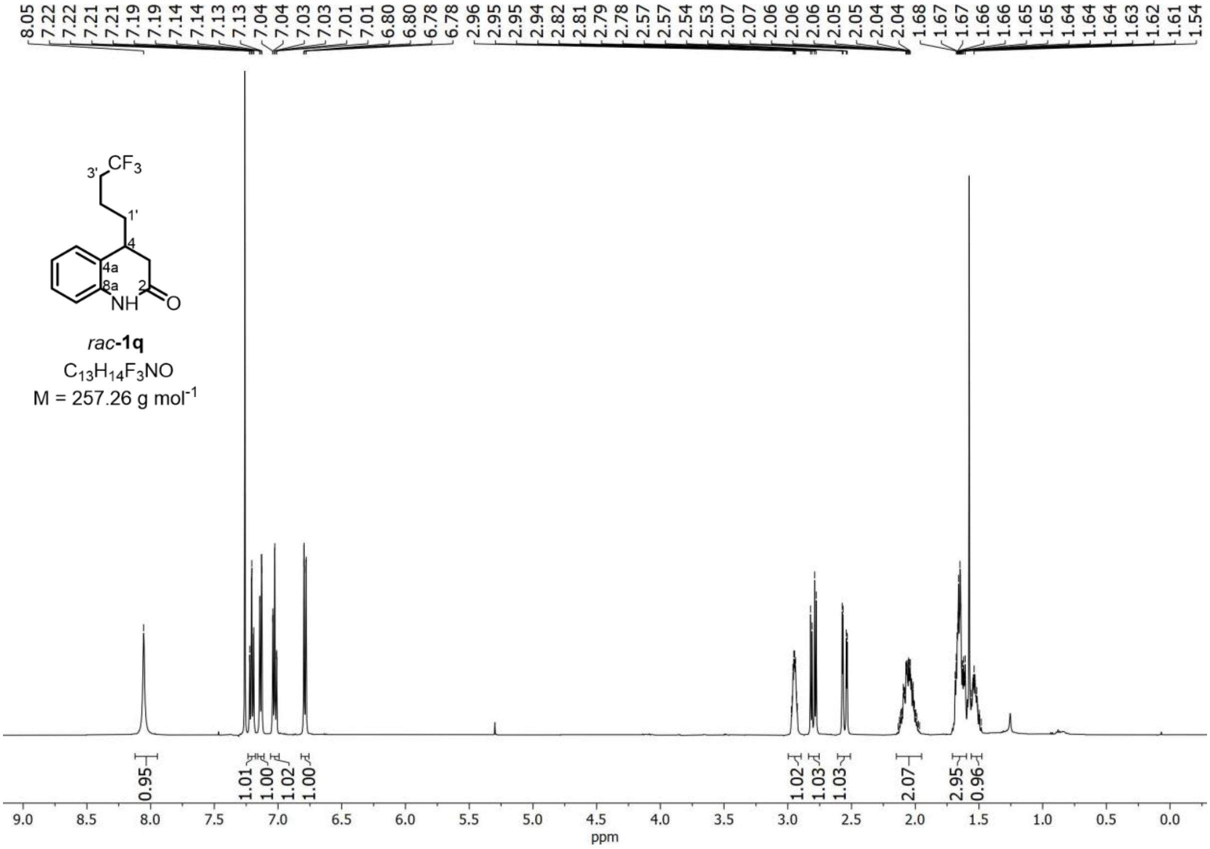

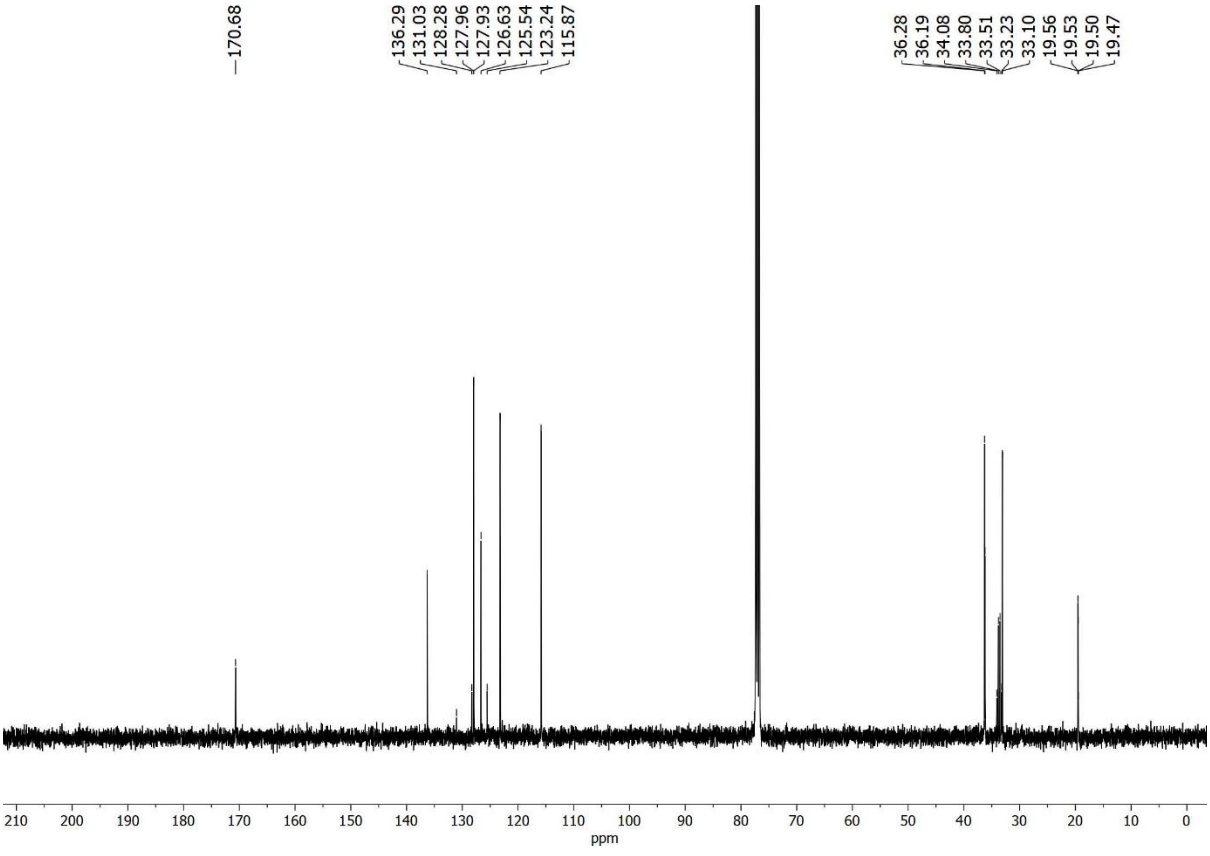

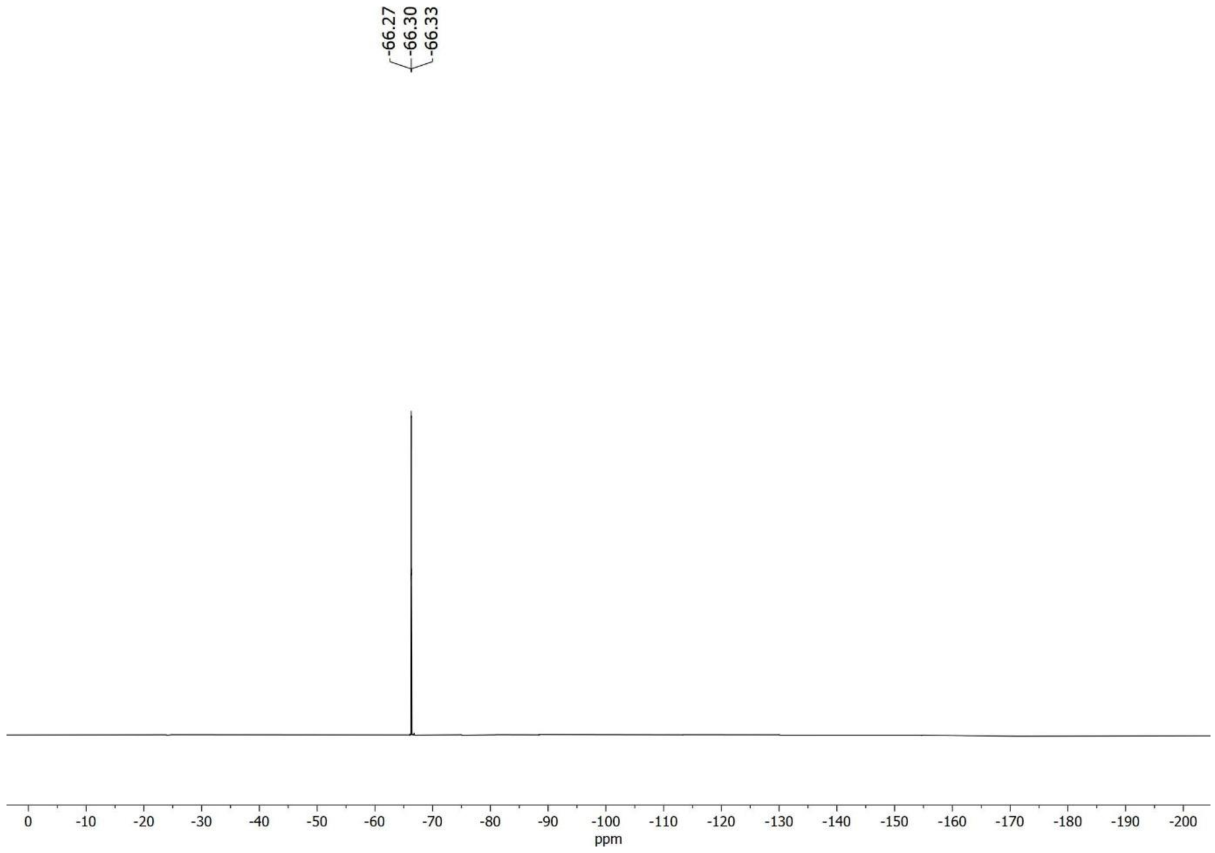
-66.27
-66.30
-66.33
0
-10
-20
-30
-40
-50
-60
-70
-80
-90
-100
-110
-120
-130
-140
-150
-160
-170
-180
-190
-200
ppm

4-(2-(Trimethylsilyl)ethyl)-3,4-dihydroquinolin-2(1*H*)-one (*rac*-1r)

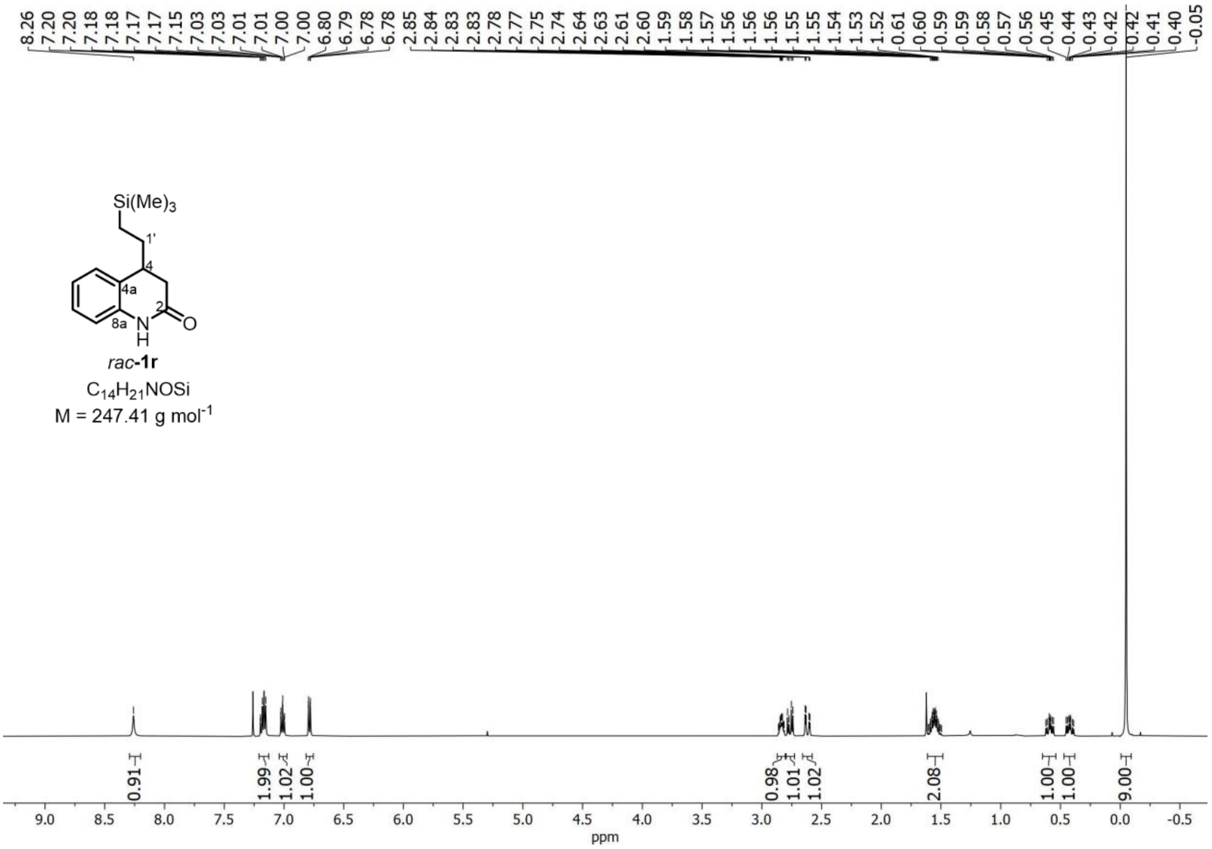

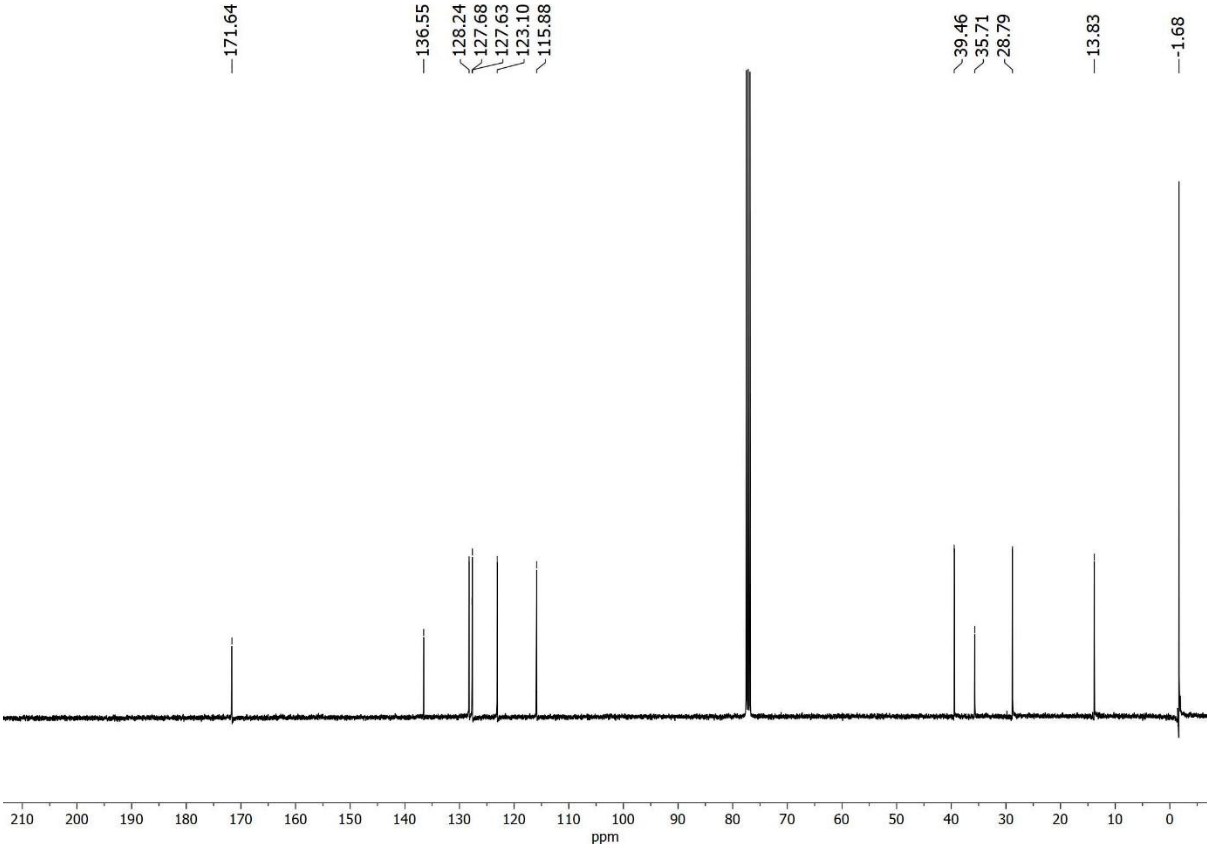

Butyl 2-oxo-1,2,3,4-tetrahydroquinoline-4-carboxylate (*rac*-1s)

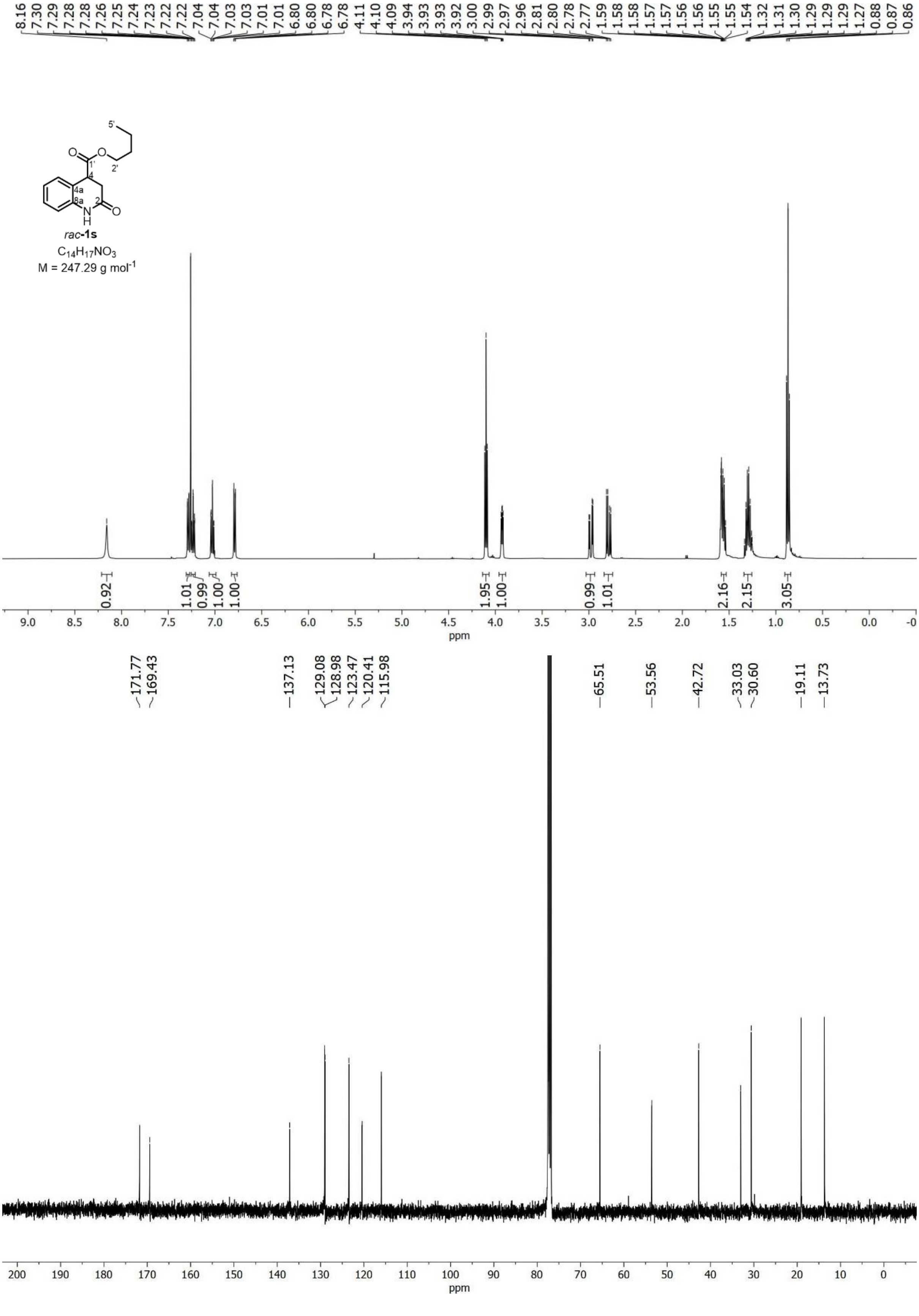

4-Methyl-1,4-dihydro-2*H*-benzo[*d*][1,3]oxazin-2-one (*rac*-8)

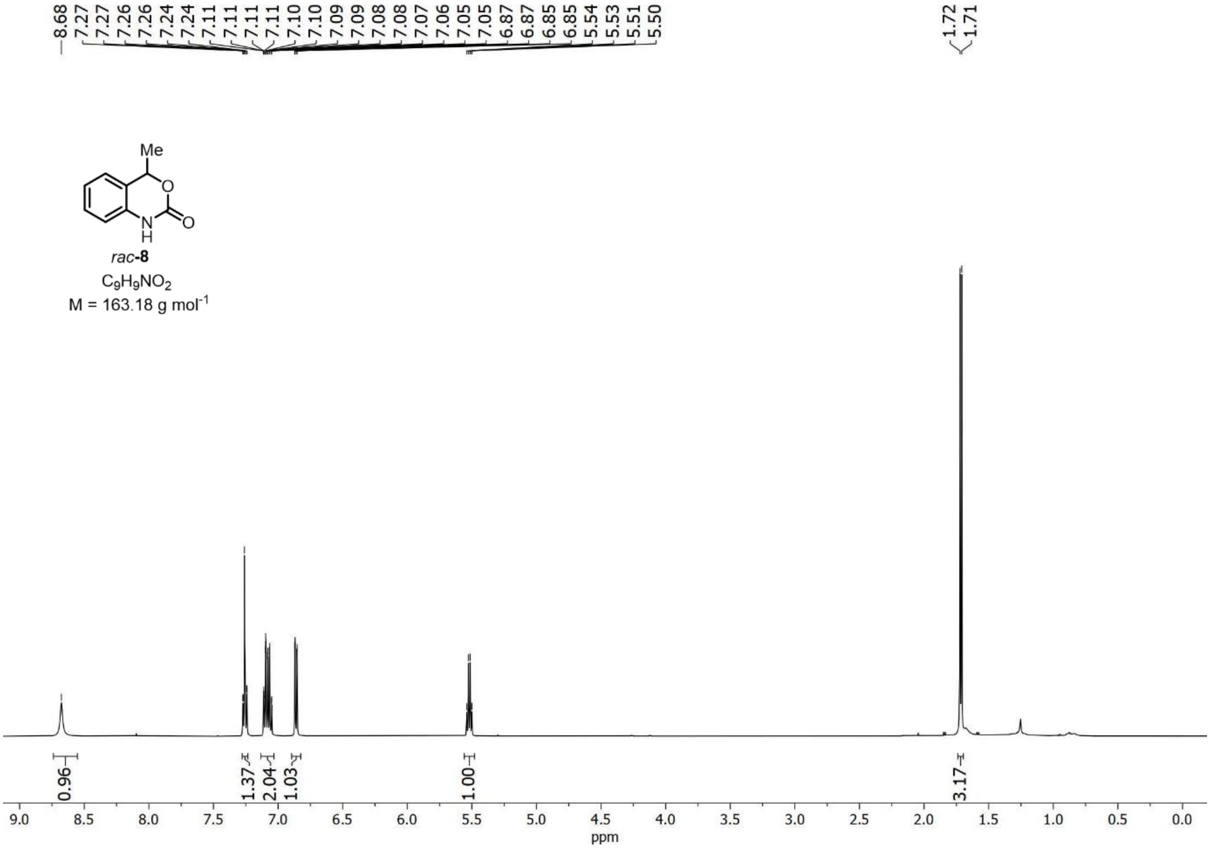

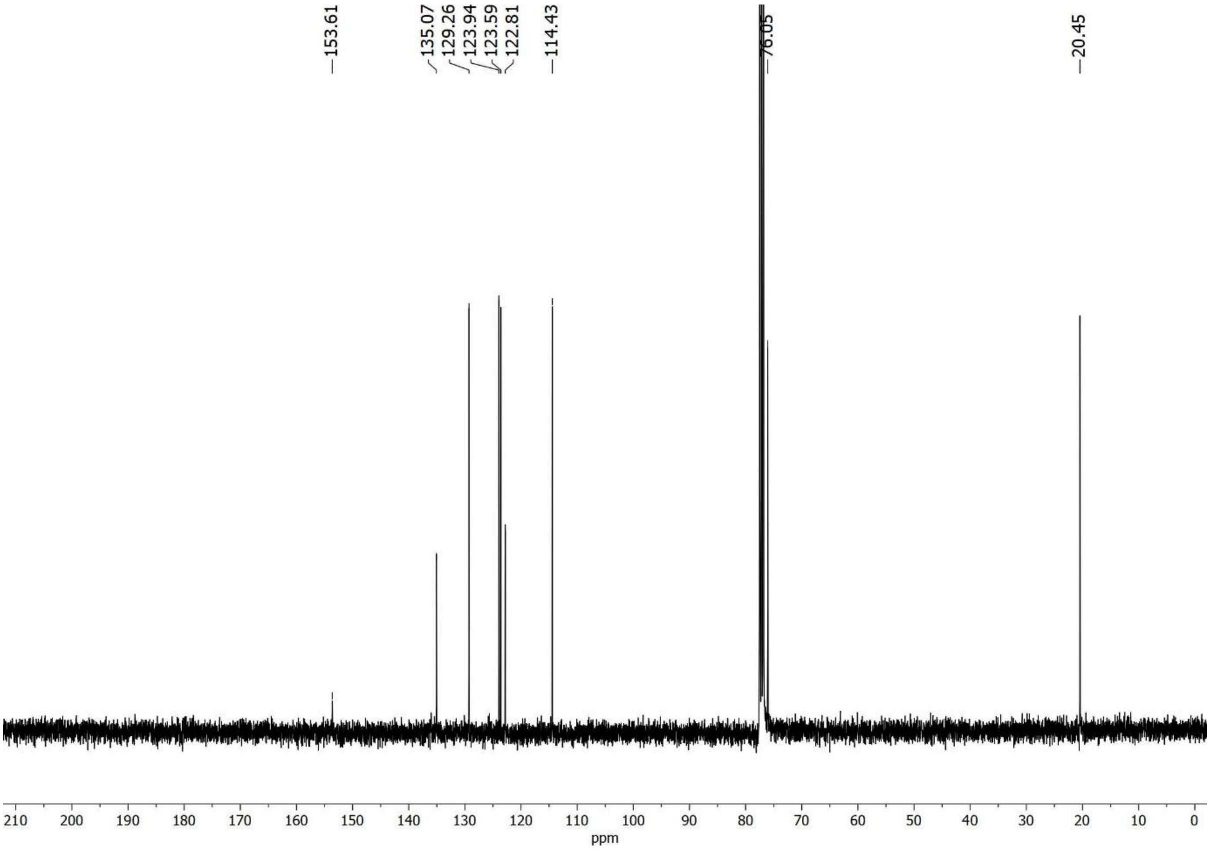

2-Methyl-2*H*-benzo[*b*][1,4]oxazin-3(4*H*)-one (*rac*-9)

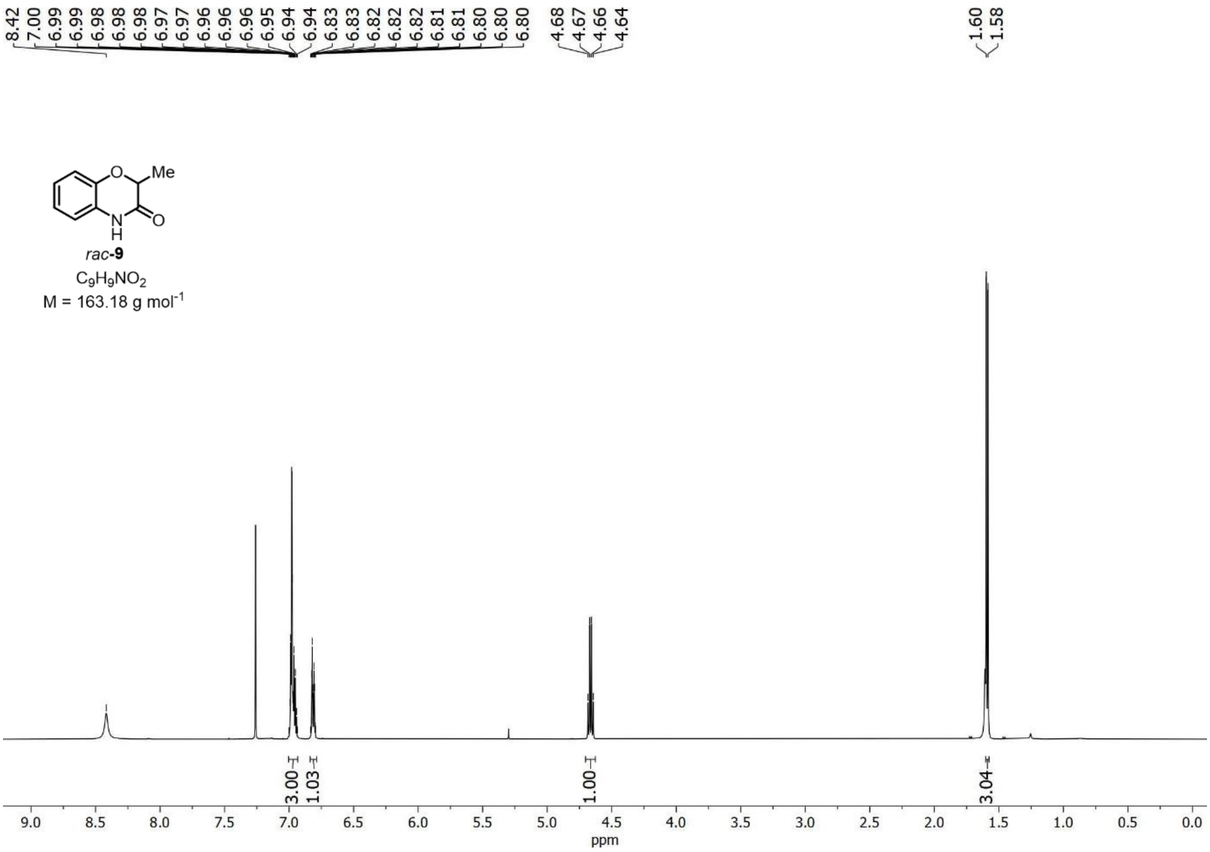

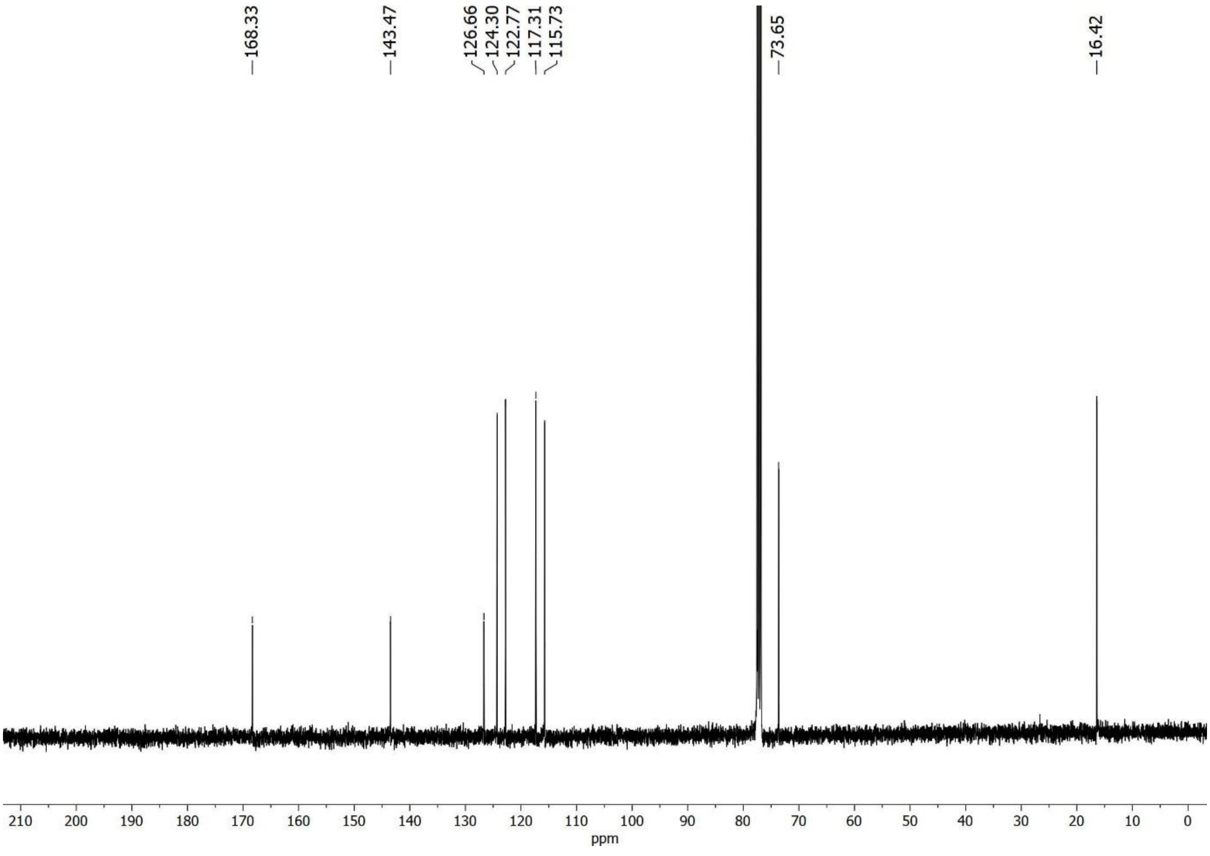

1-Methyl-4-phenyl-3,4-dihydroquinolin-2(1*H*)-one (*rac*-10)

rac-**10**

$C_{16}H_{15}NO$

M = 237.30 g mol^{-1}

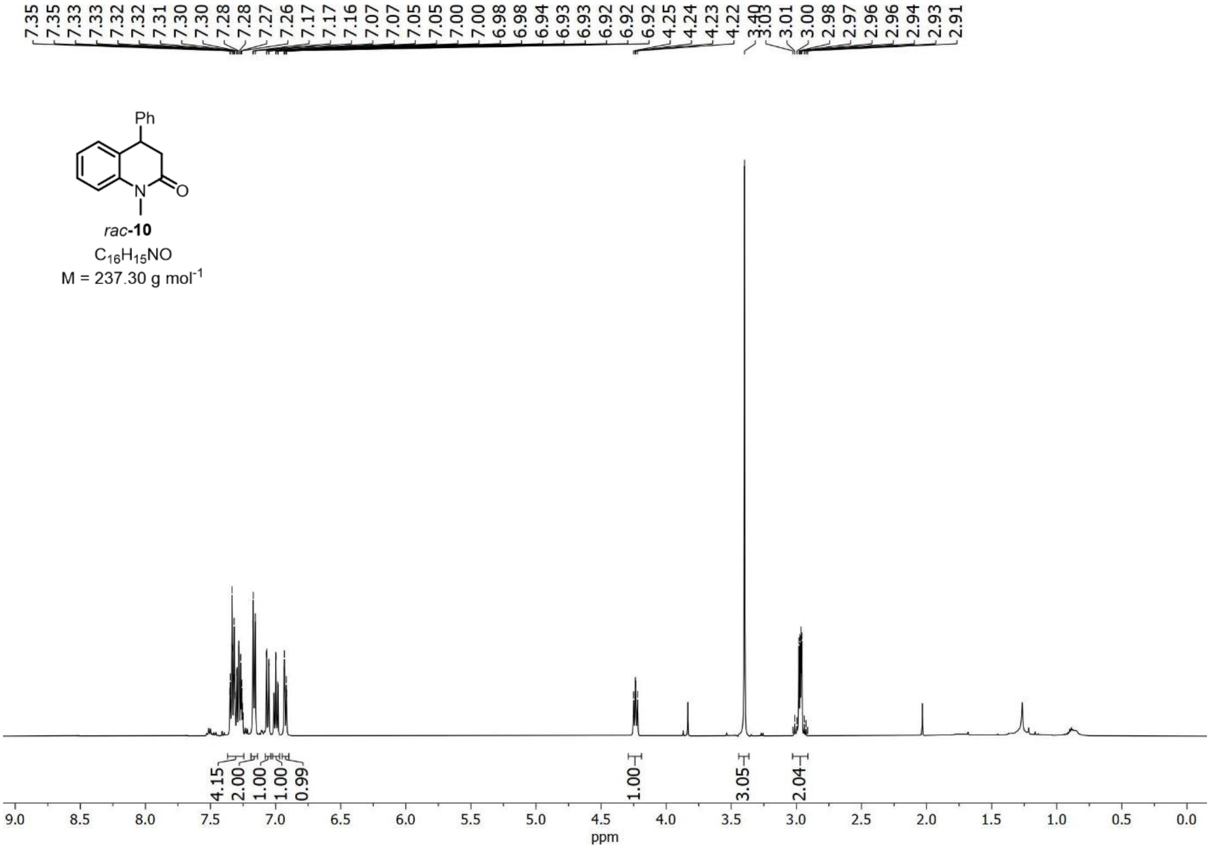

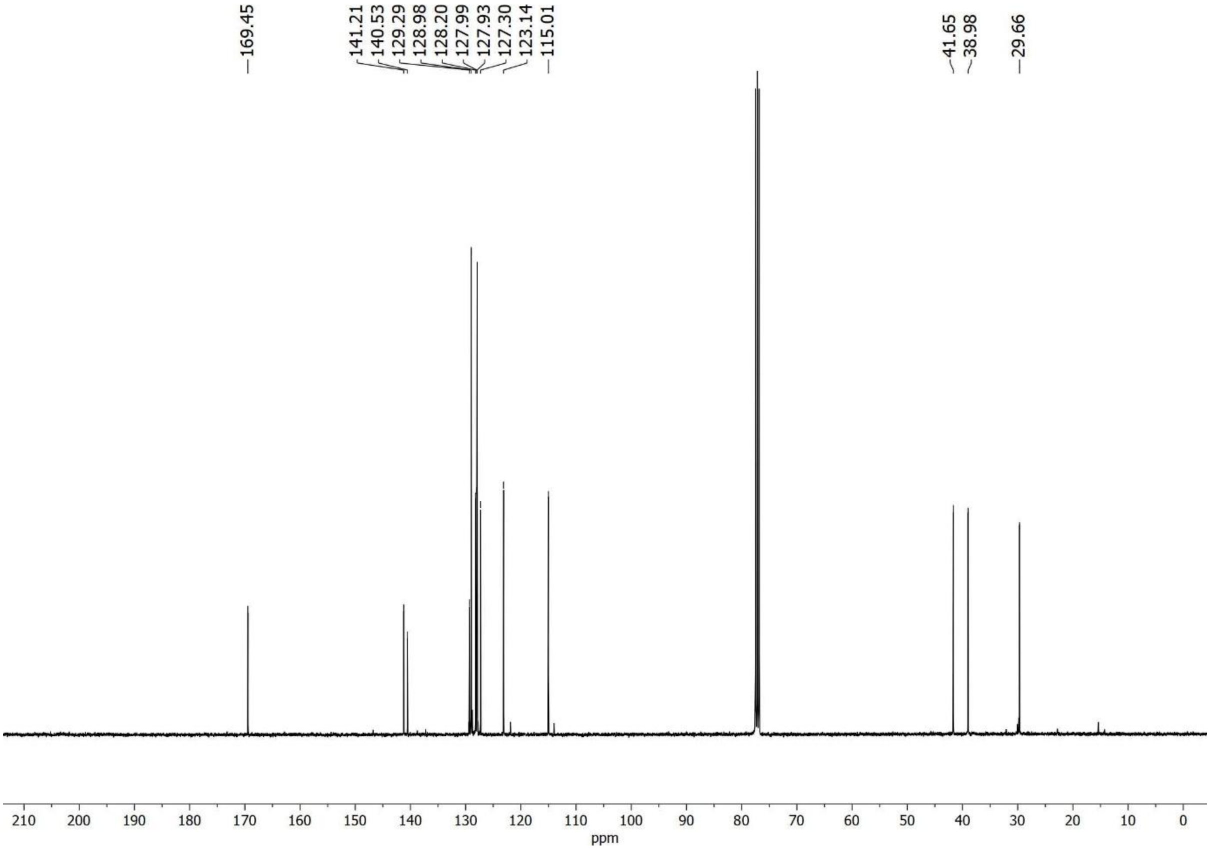

4-Phenyl-3,4-dihydroquinolin-2(1*H*)-one-4-*d* (*rac*-1a-d_1)

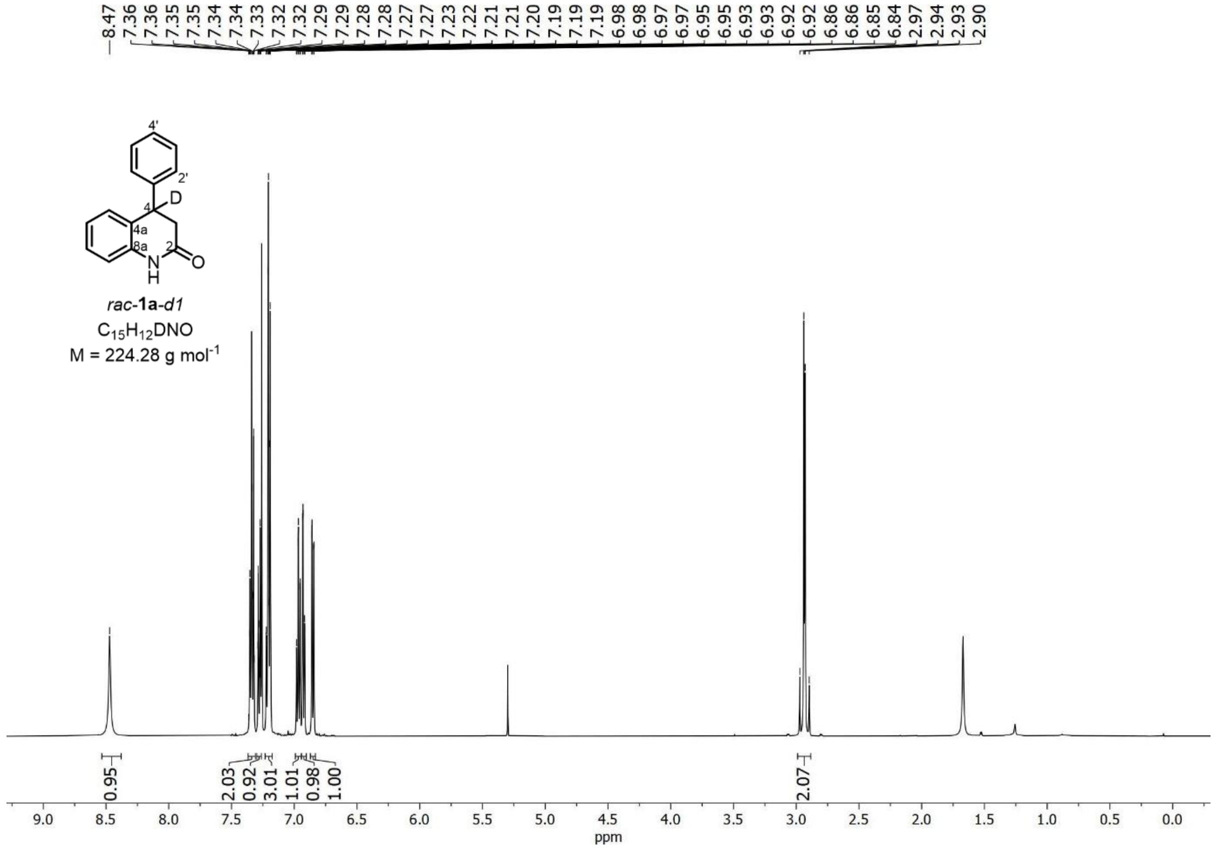

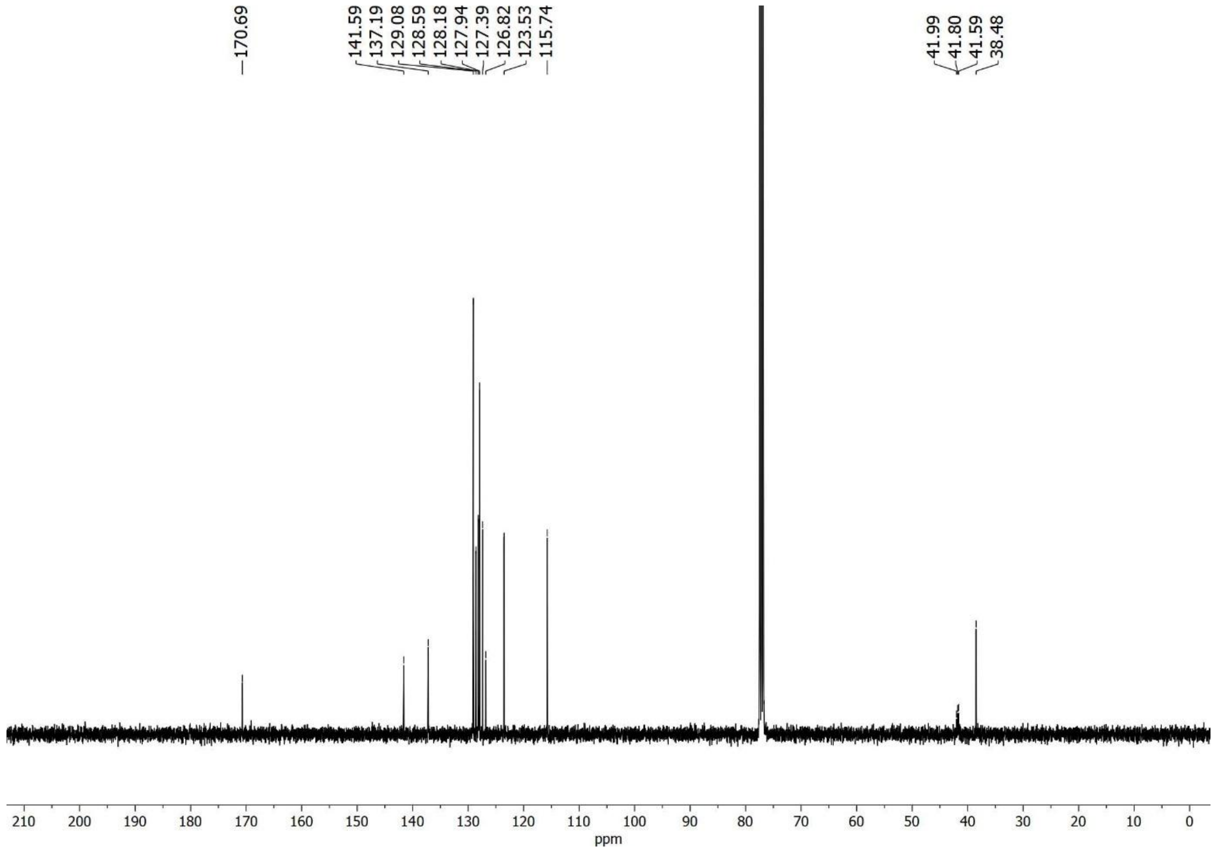

Deuteriobenzaldehyde (S1)

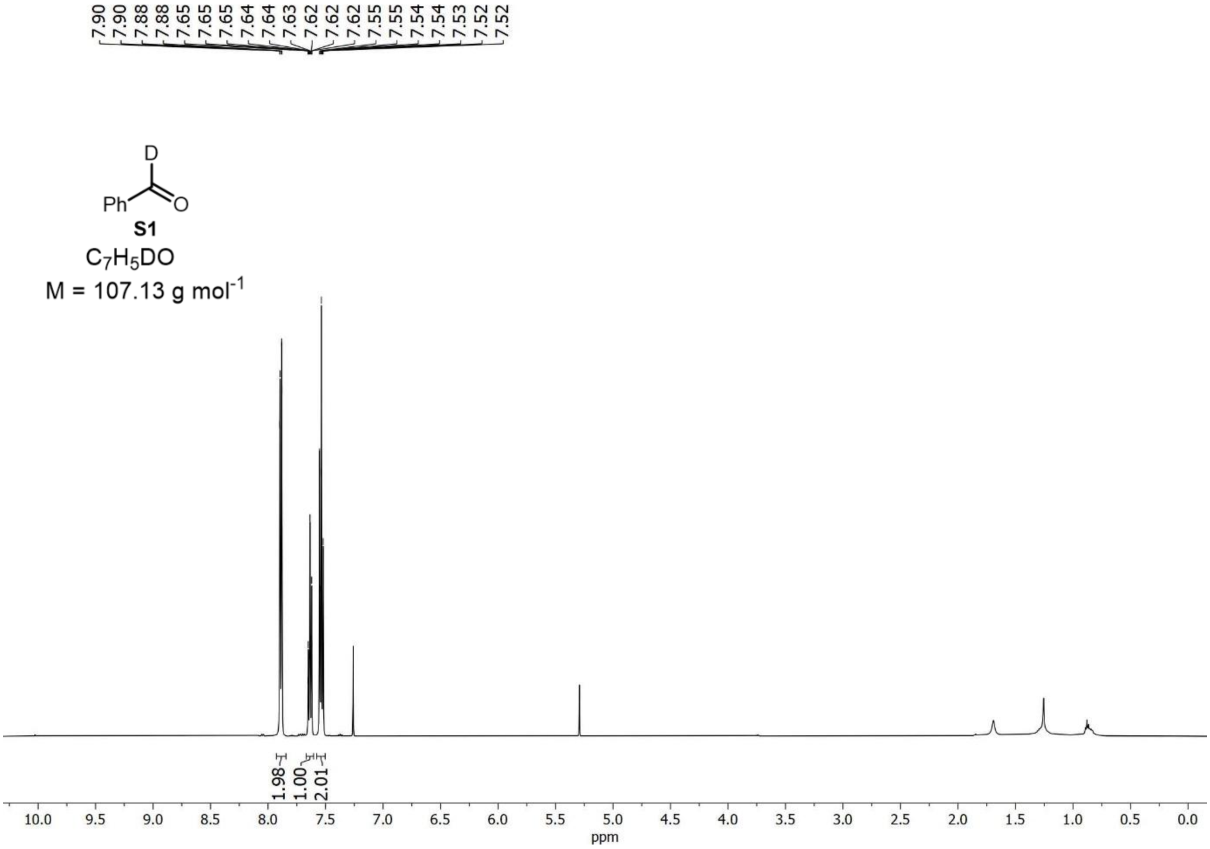

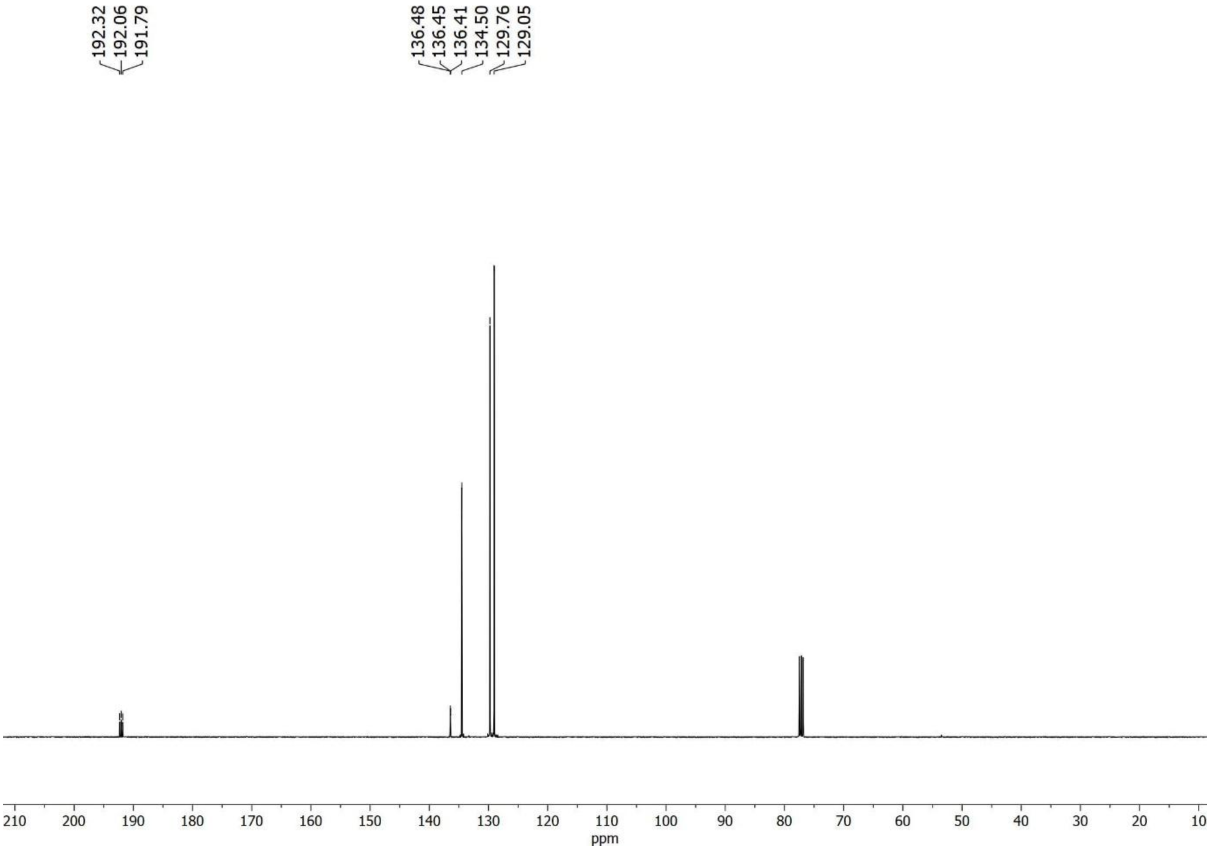

Cinnamic-3-*d* acid (S2)

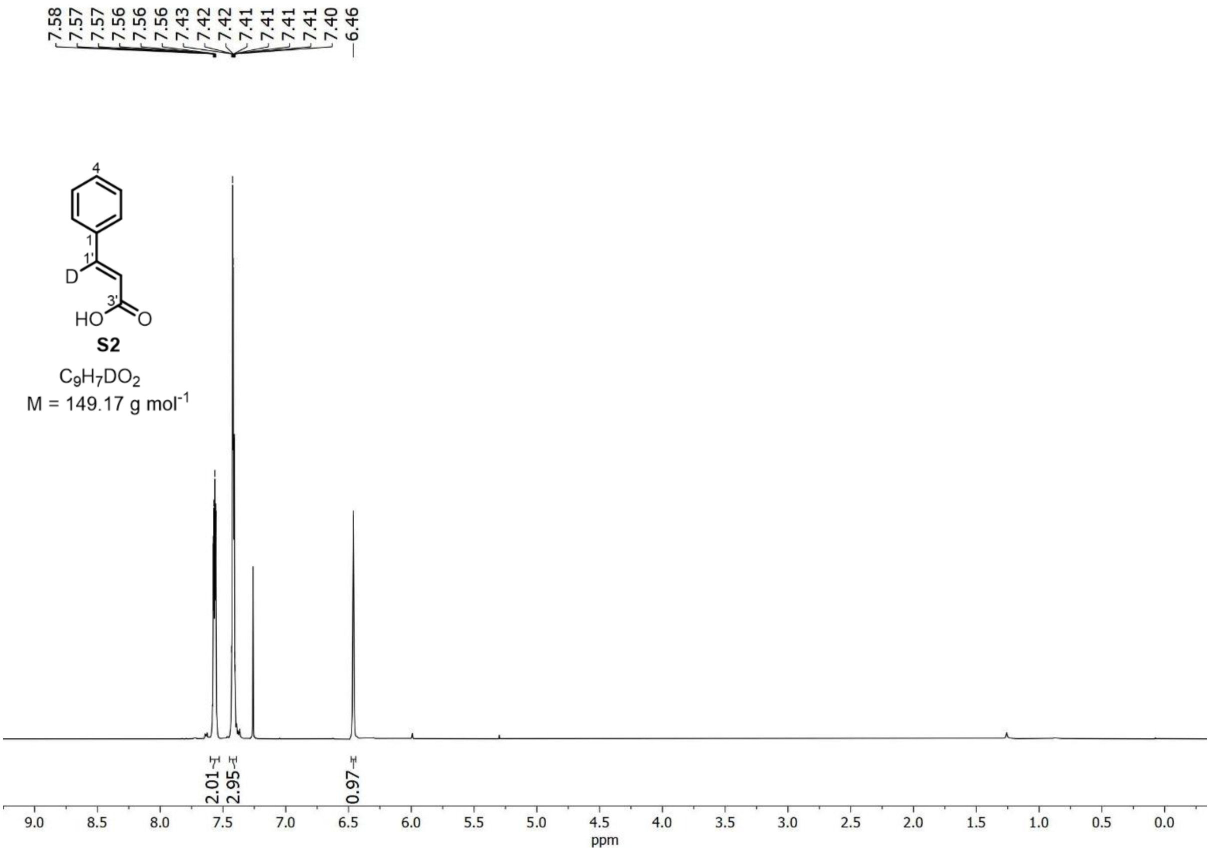

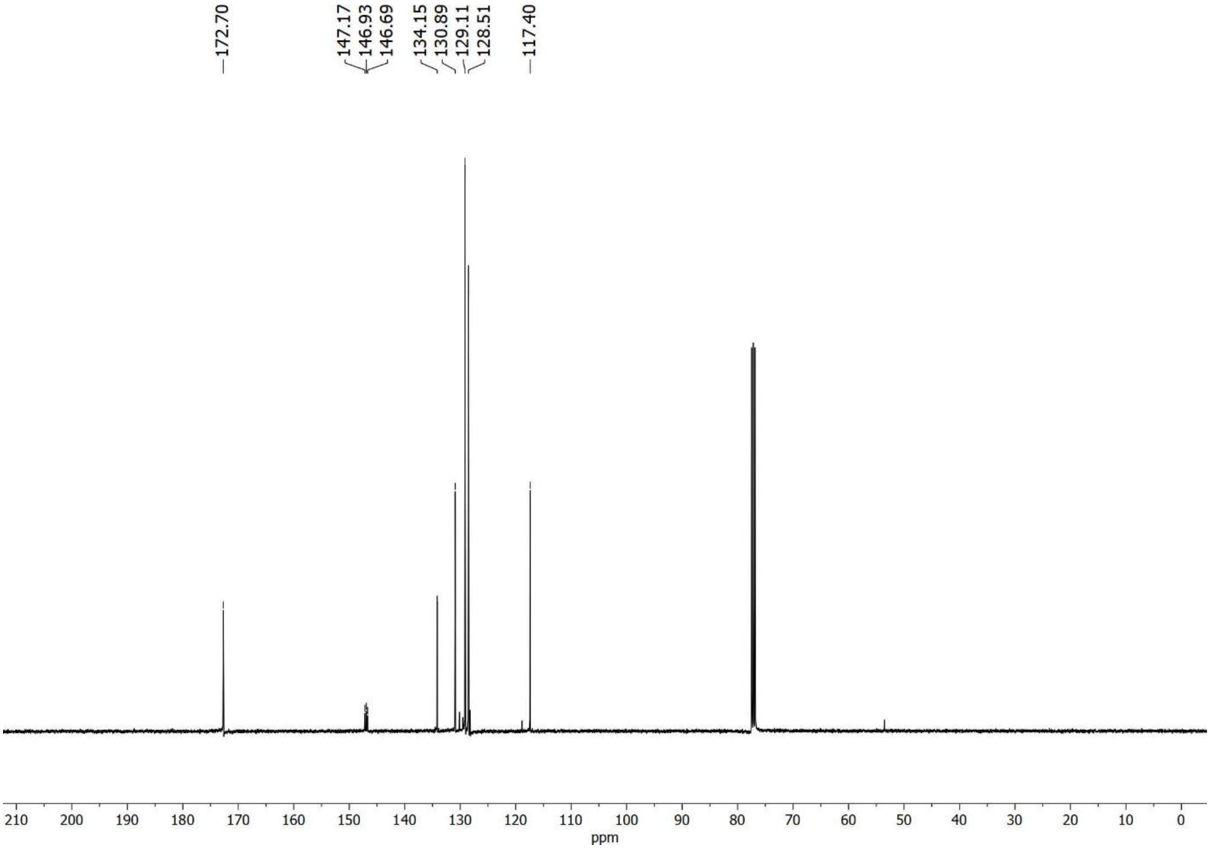

4-Phenyl-3,4-dihydroquinolin-2(1*H*)-one-3,3-*d*$_2$ (*rac*-1a-*d*$_2$)

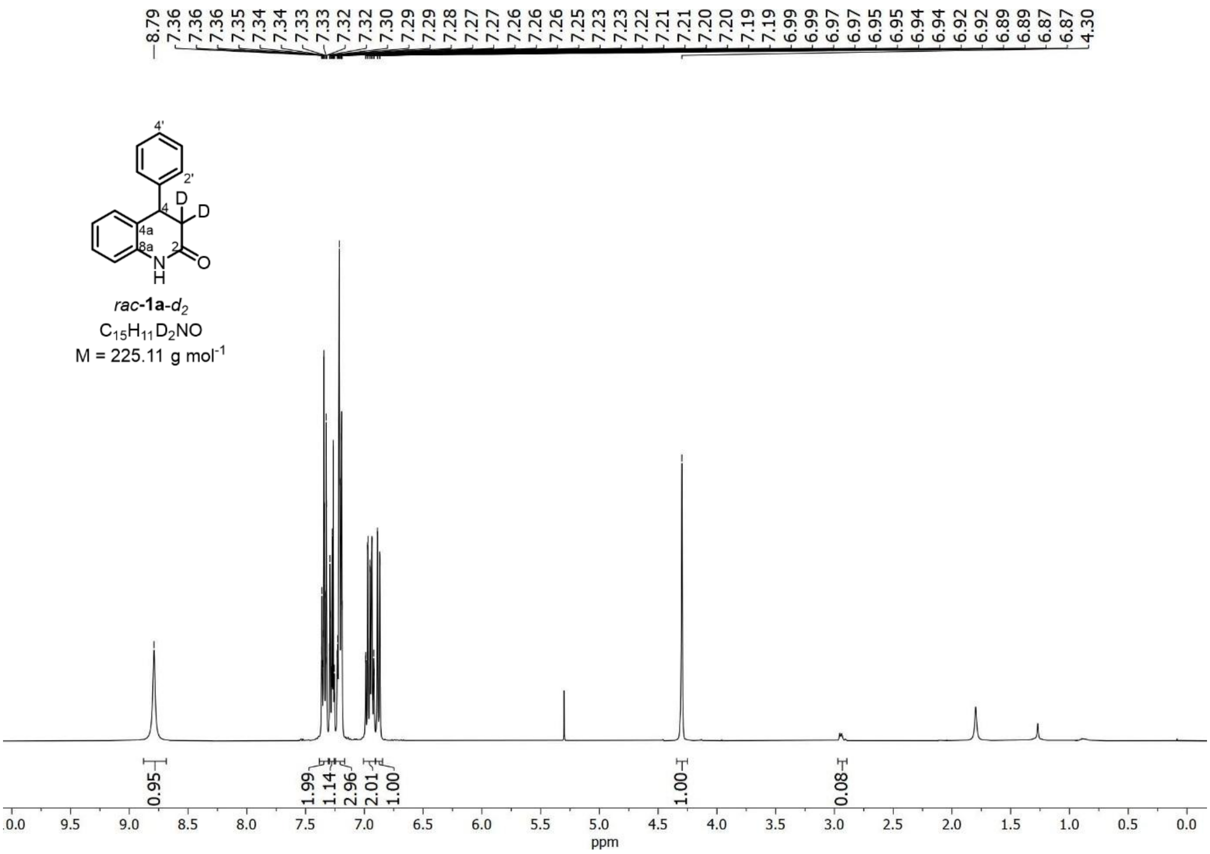

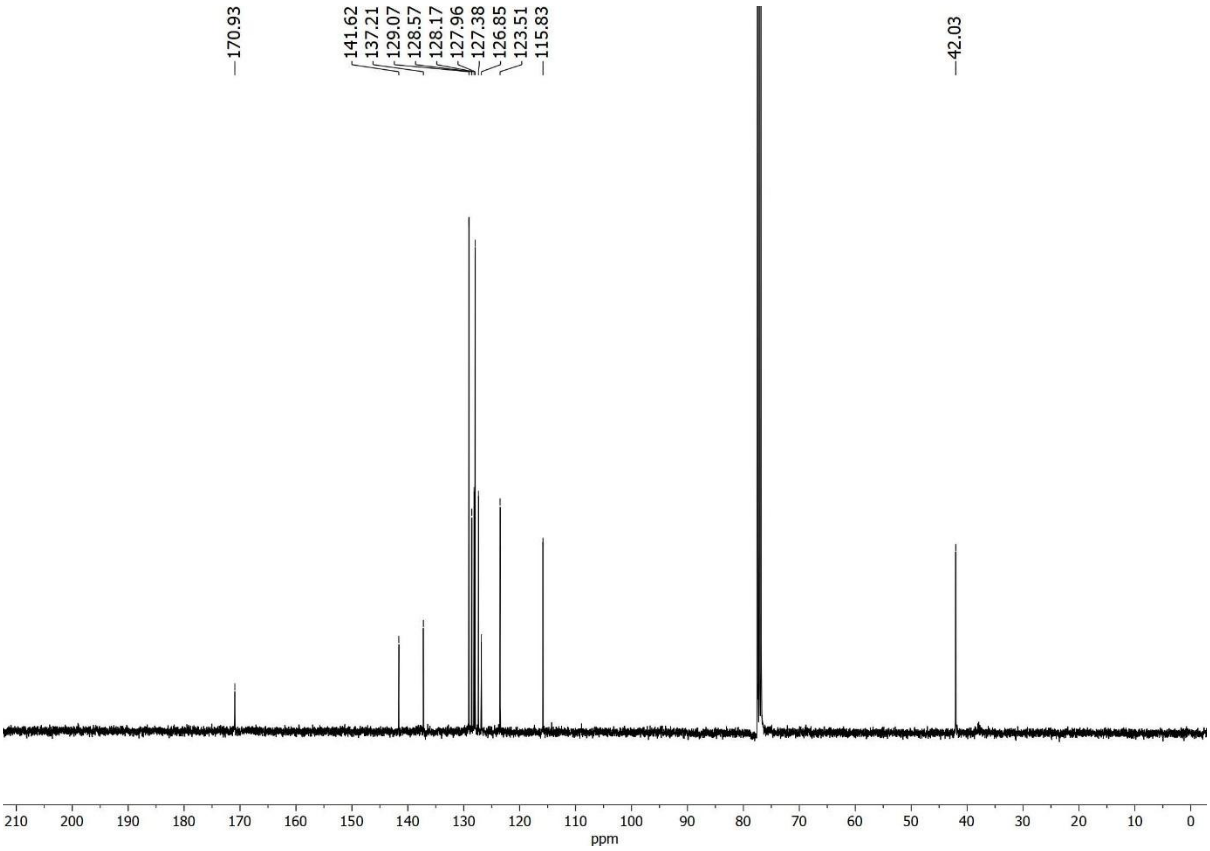

3-Phenyl-2,3-dihydro-1*H*-inden-1-one-2,2-*d*$_2$ (S3)

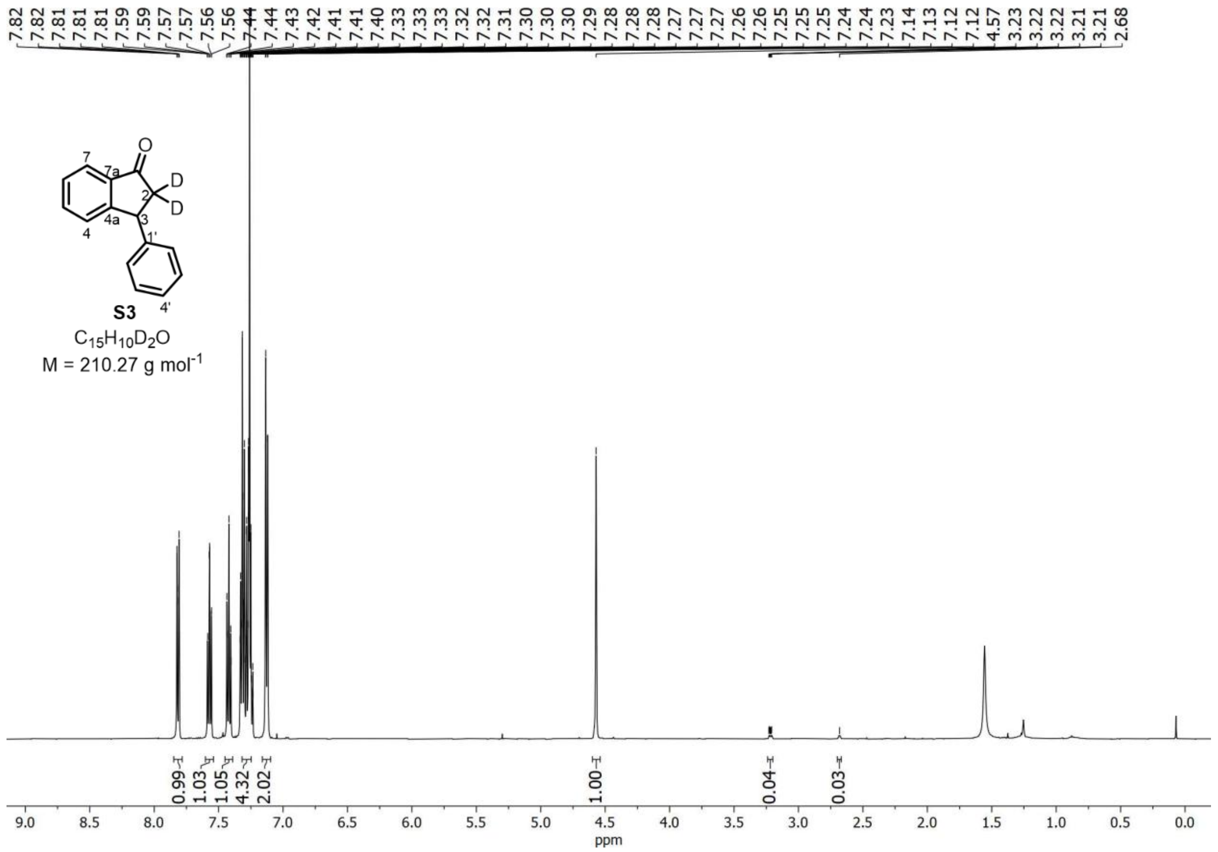

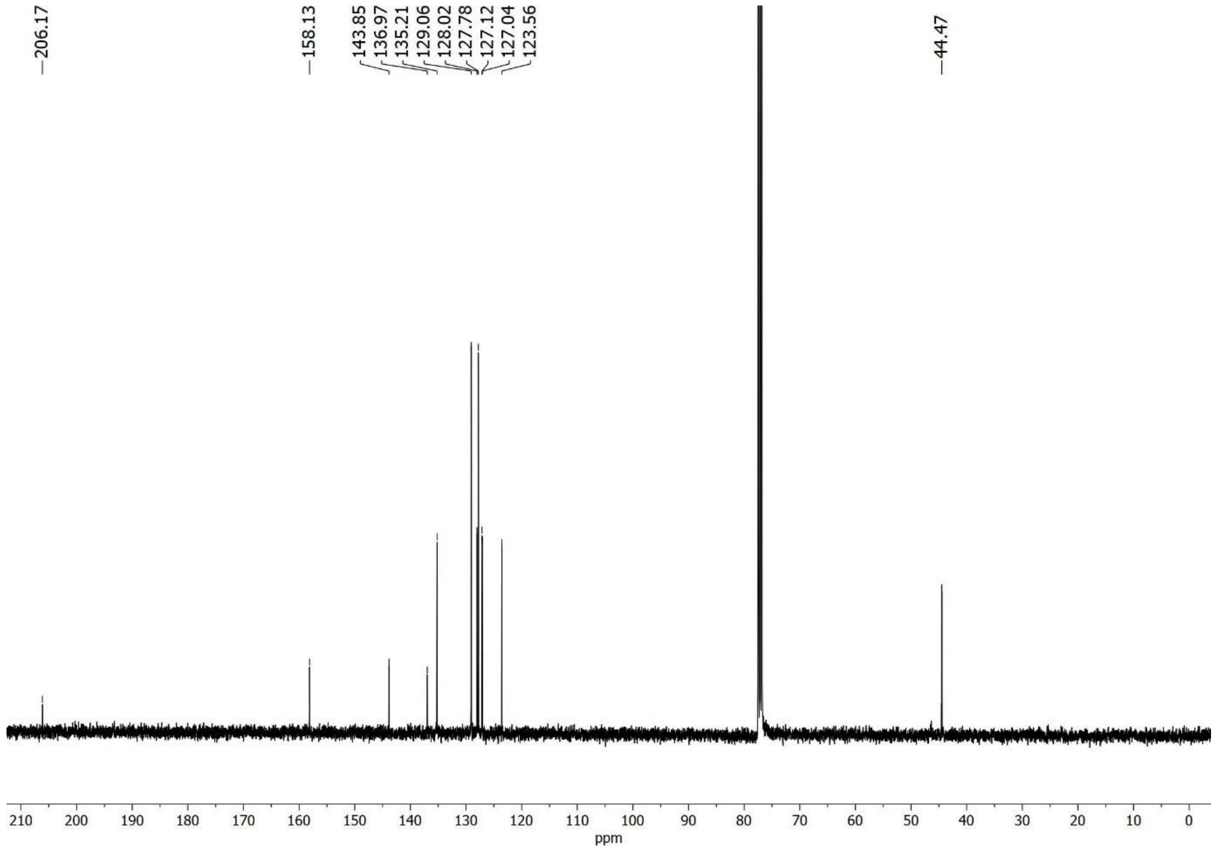

4-Phenylquinolin-2(1*H*)-one (6a)

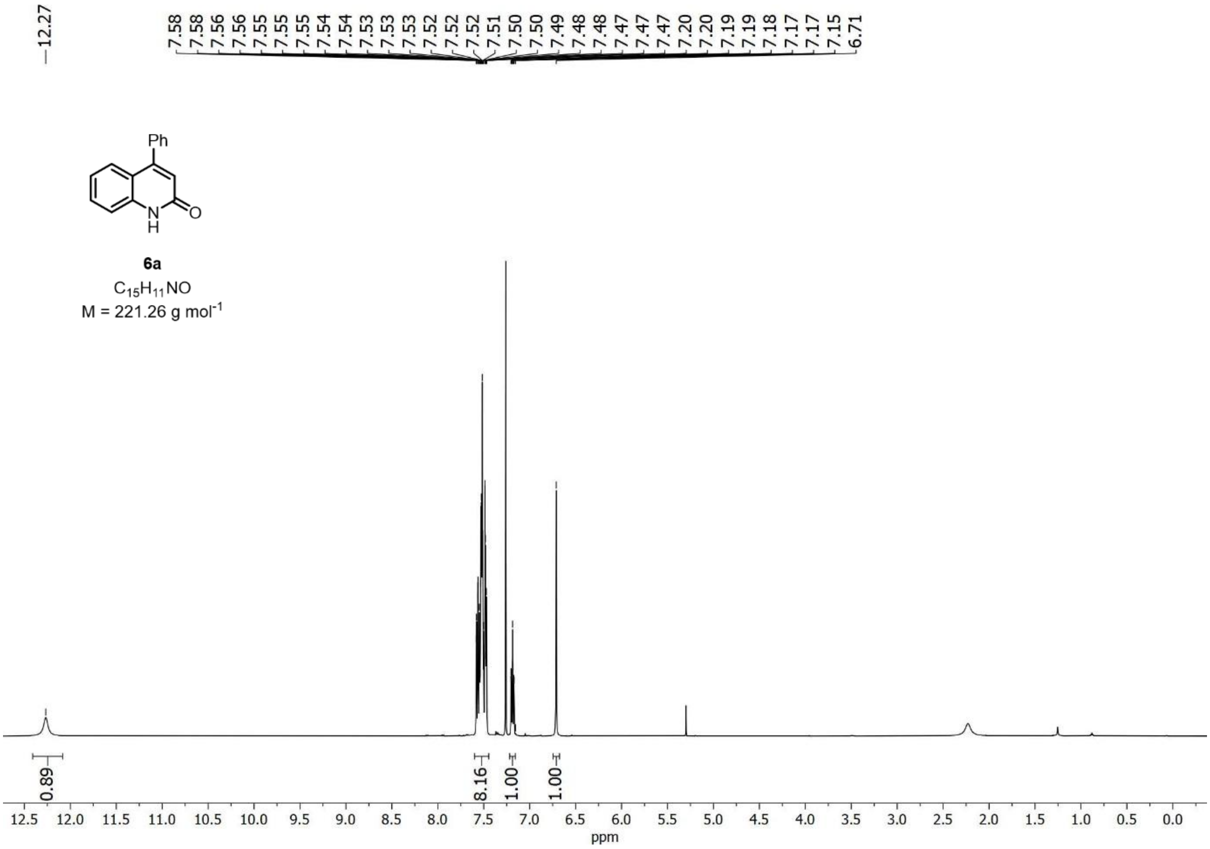

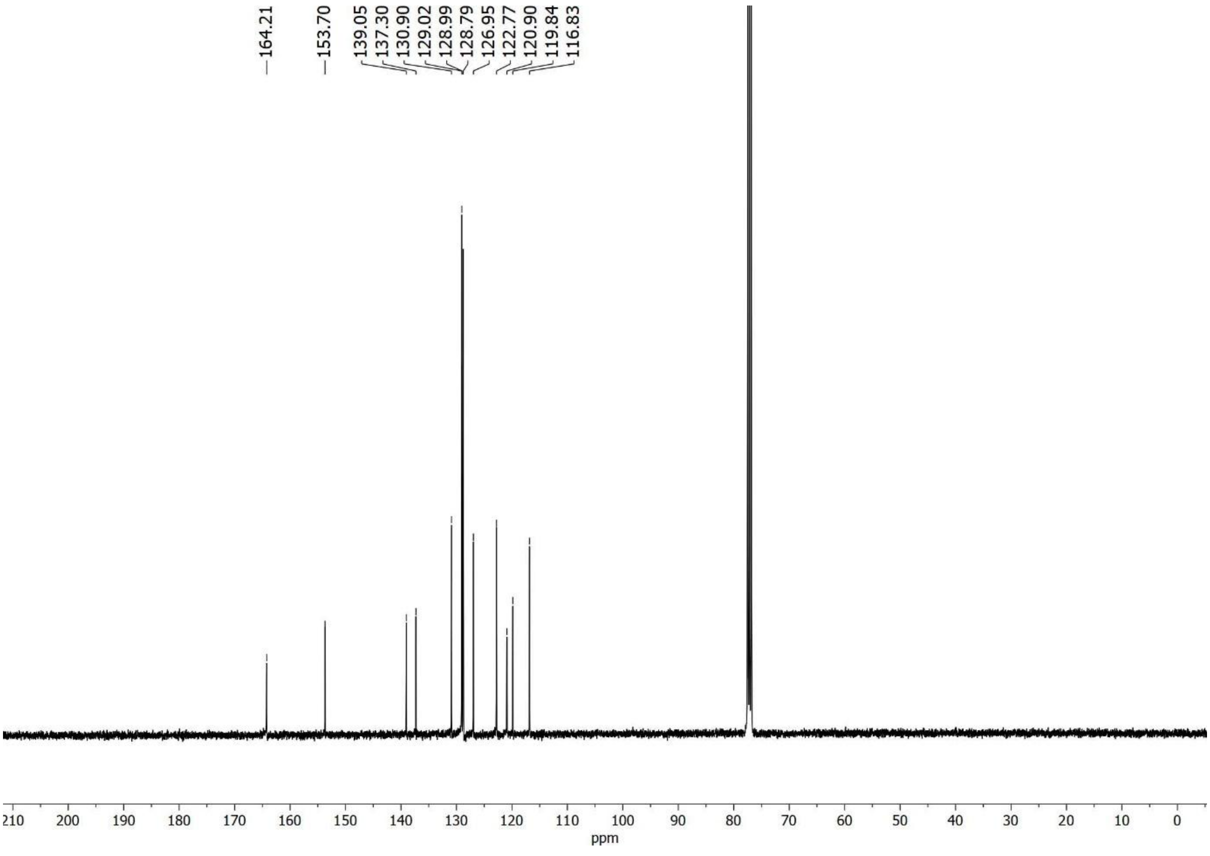

4-Methylquinolin-2(1*H*)-one (6l)

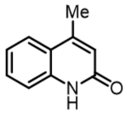

6l

C_9H_9NO

M = 159.19 g mol^{-1}

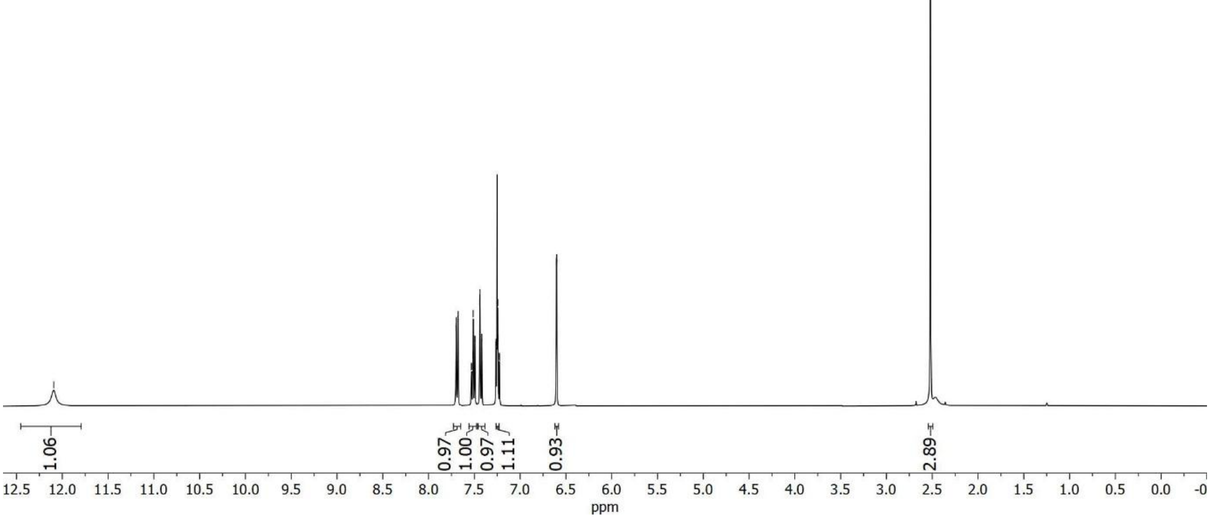

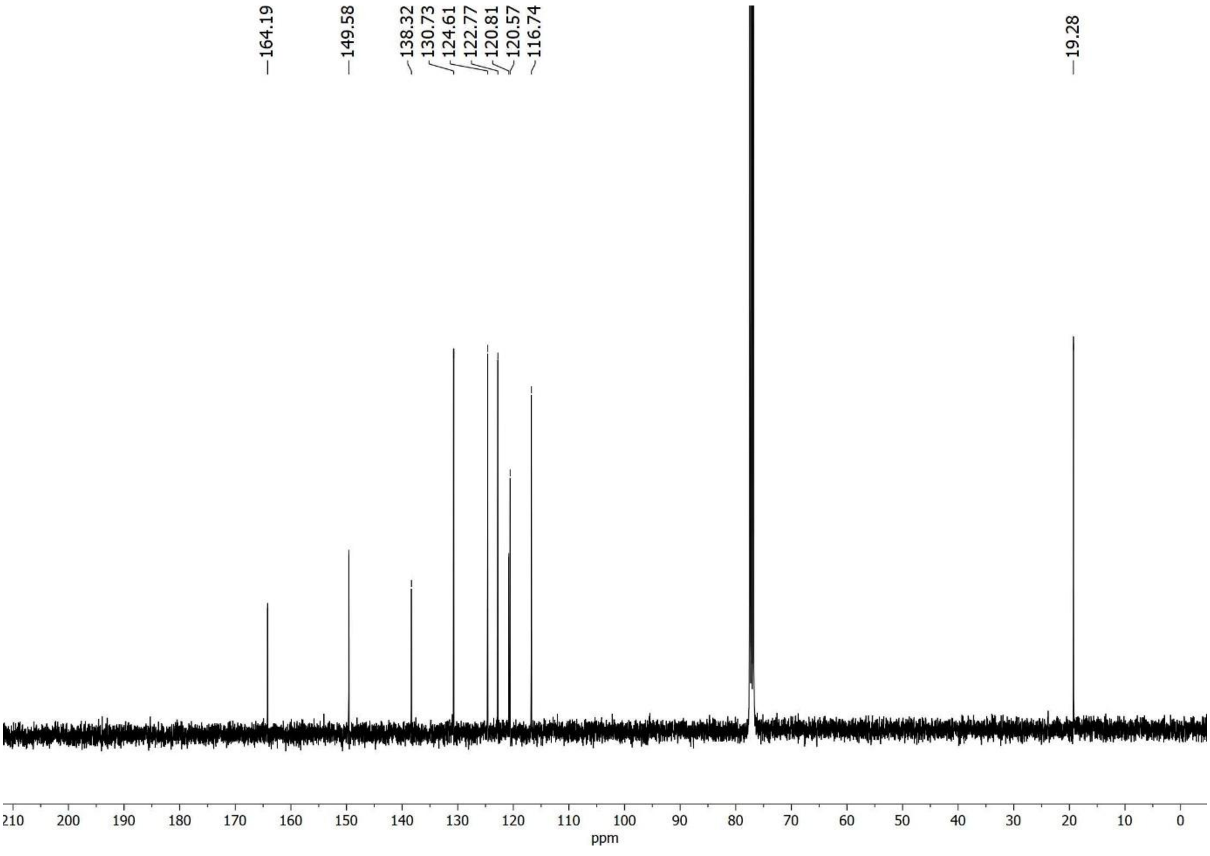

4-Ethylquinolin-2(1*H*)-one (6m)

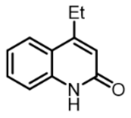

6m

$C_{11}H_{11}NO$

M = 173.22 g mol[-1]

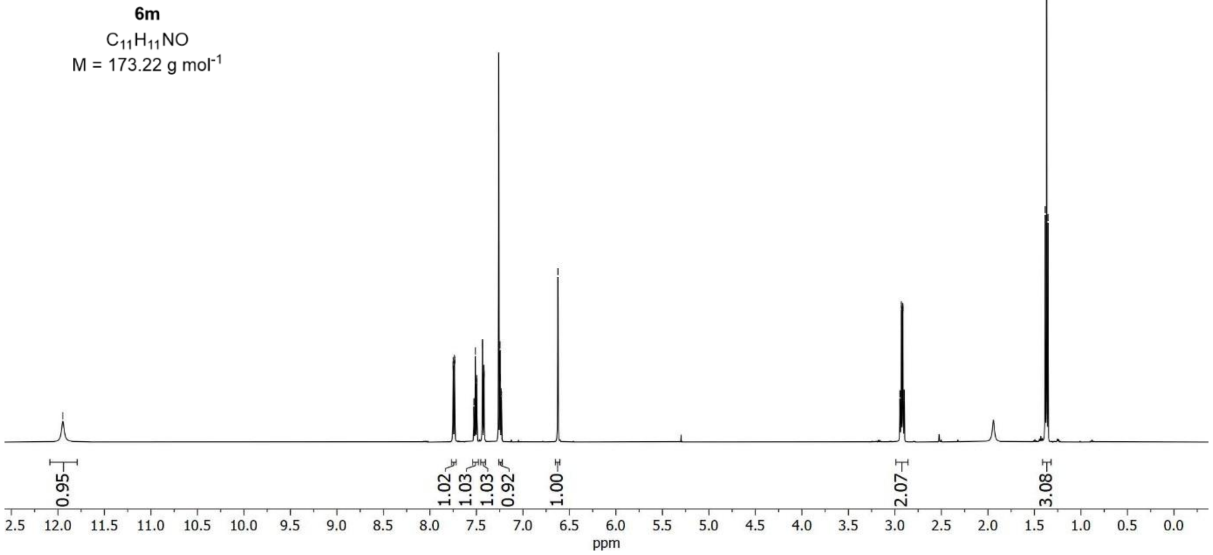

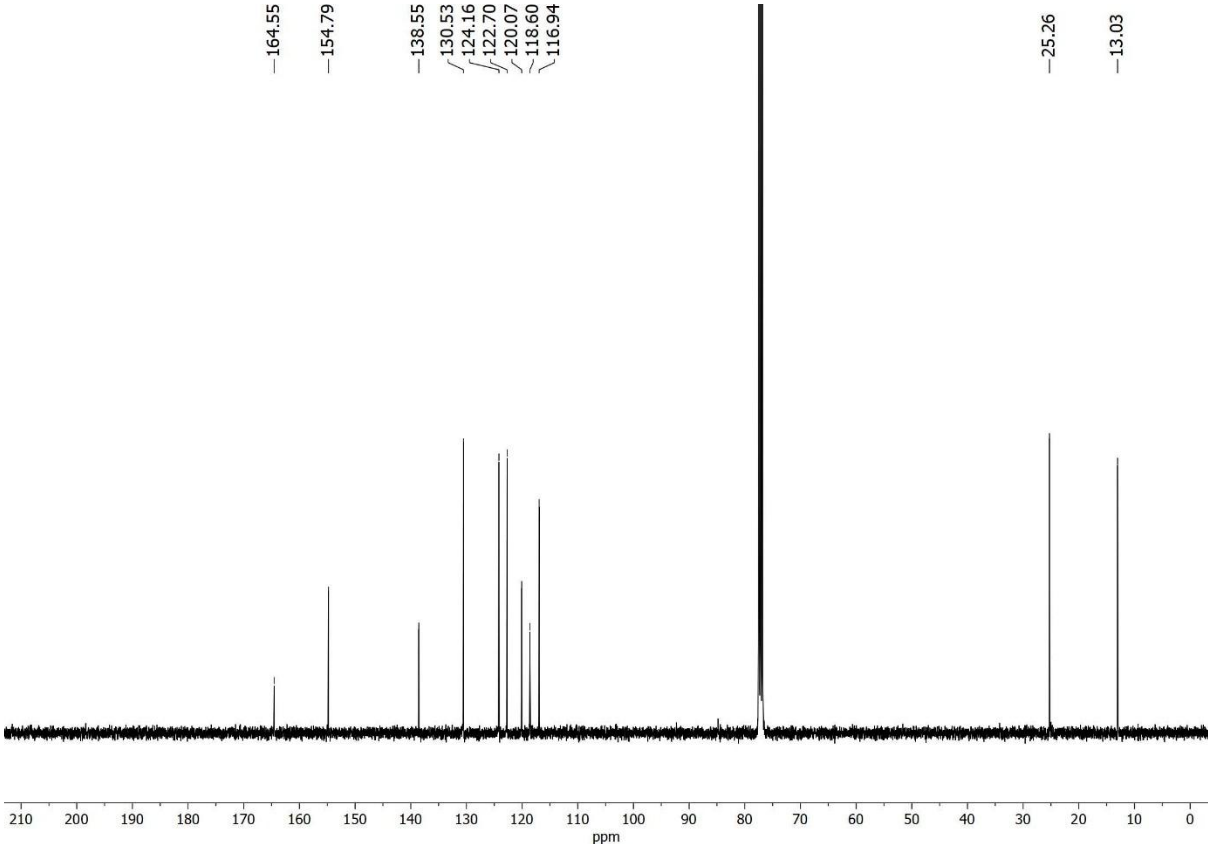

4-Propylquinolin-2(1*H*)-one (6n)

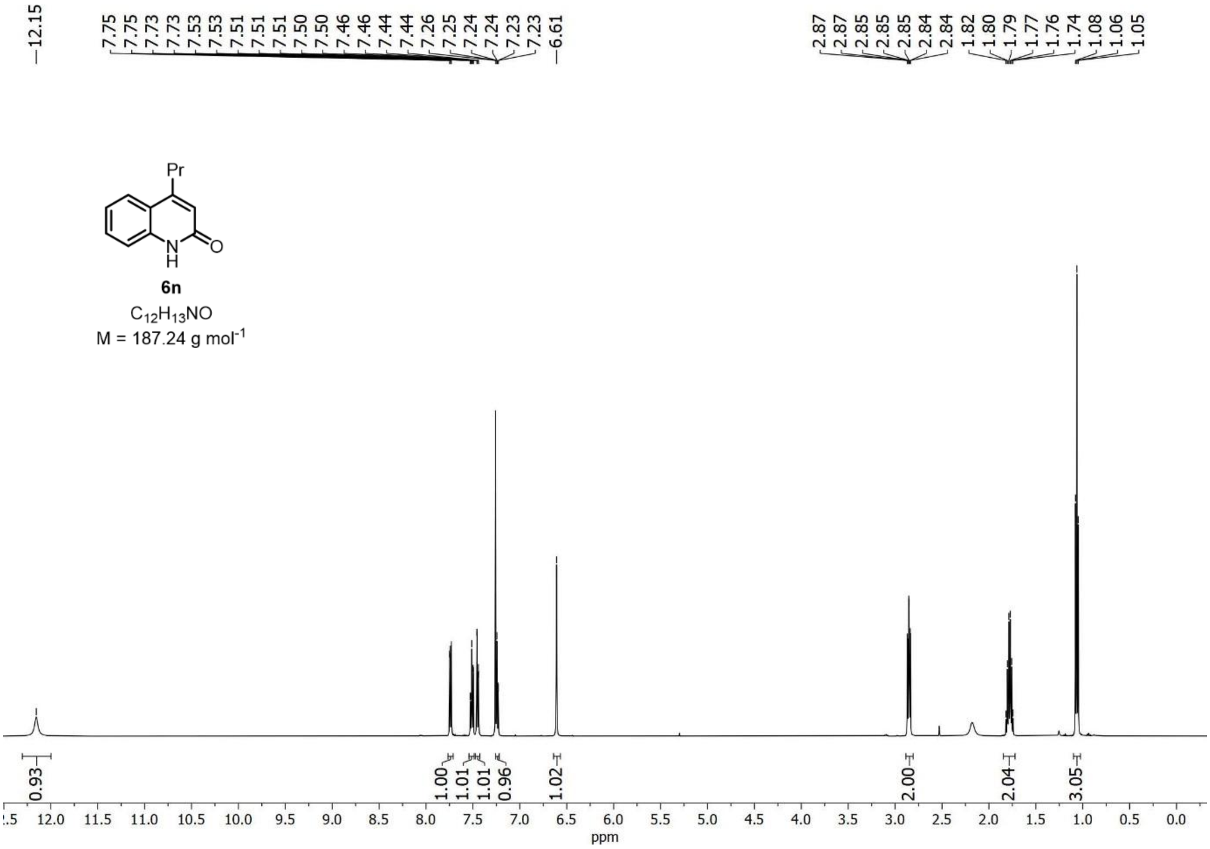

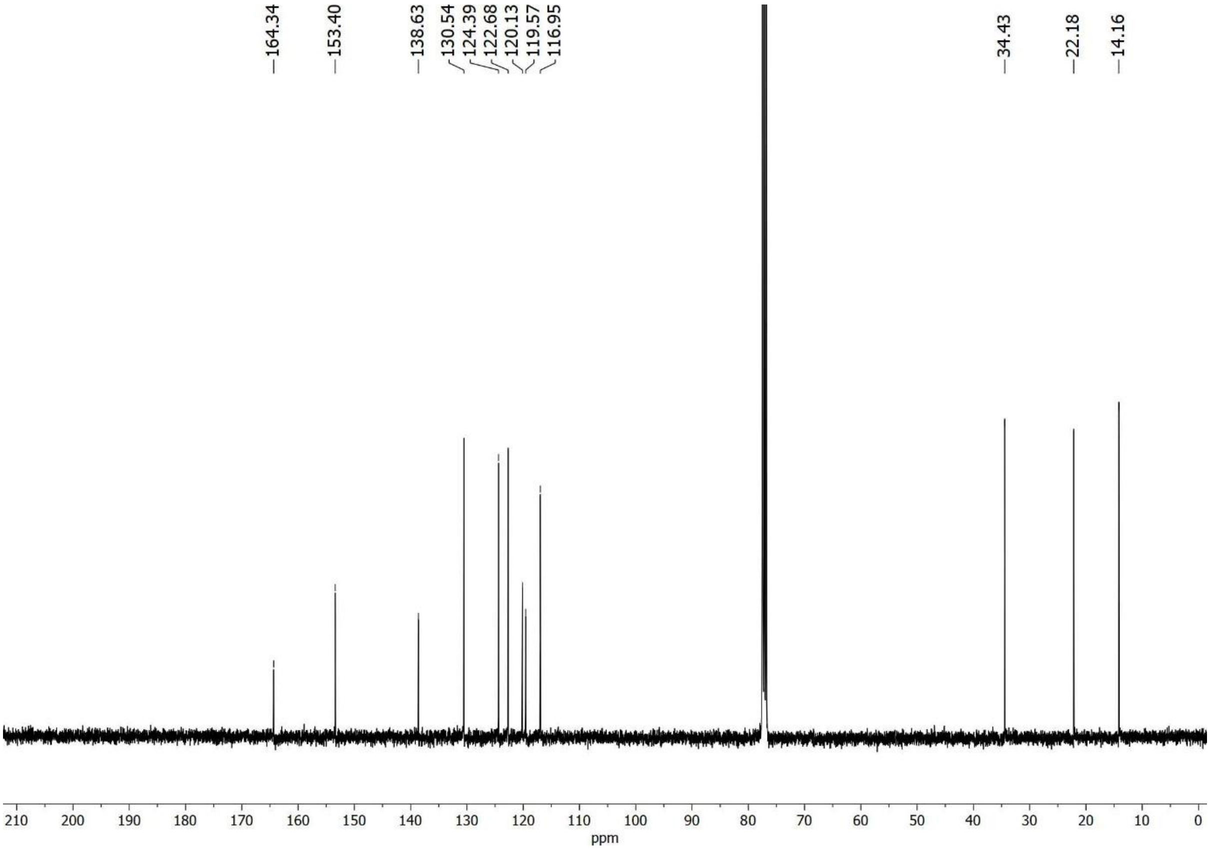

4-Cyclopropylquinolin-2(1*H*)-one (6o)

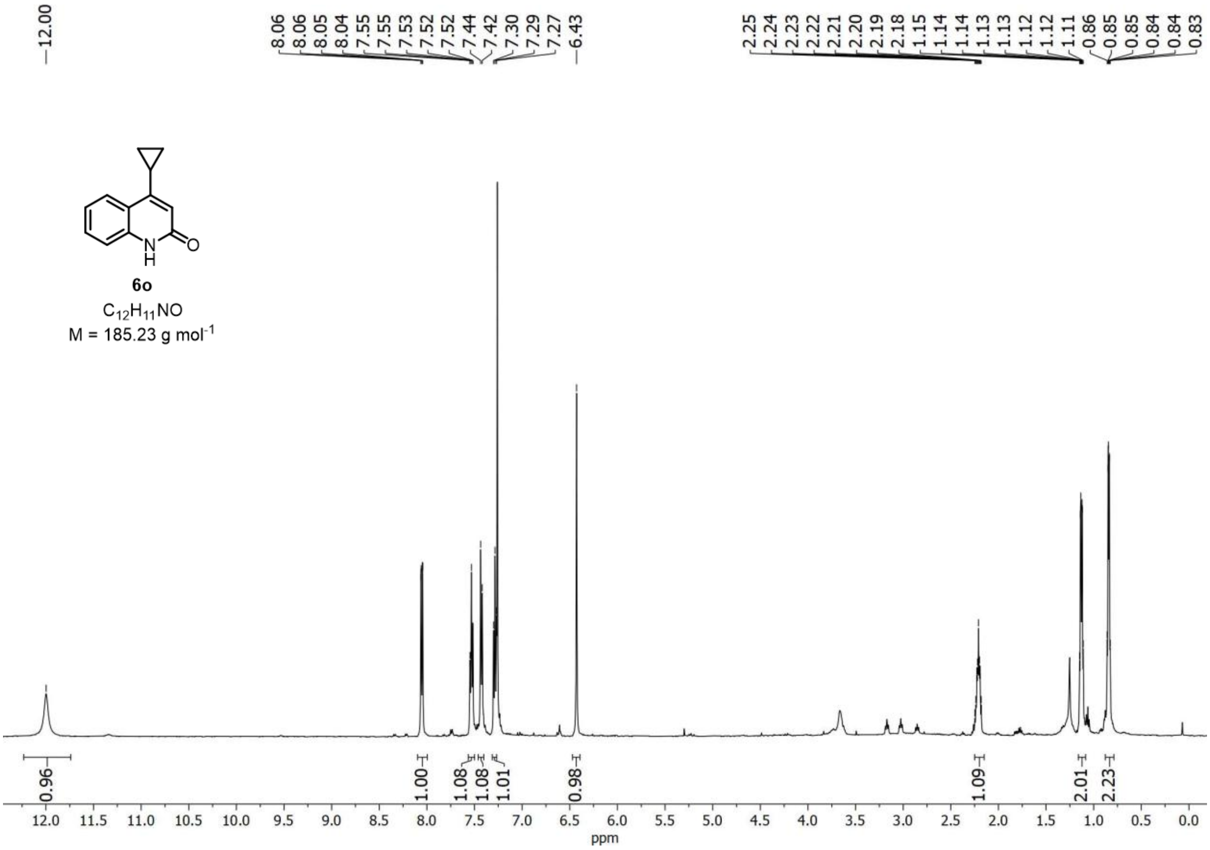

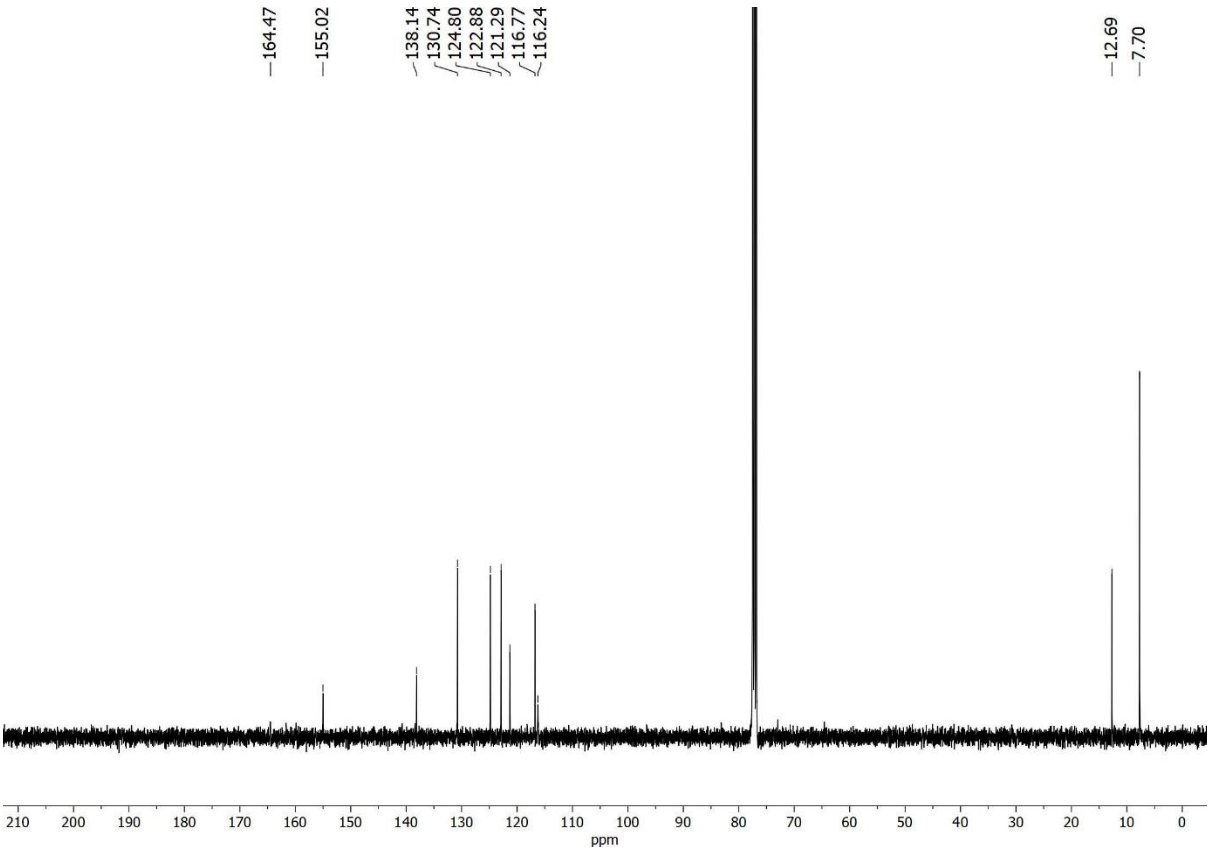

4-(4,4,4-Trifluorobutyl)quinolin-2(1*H*)-one (6q)

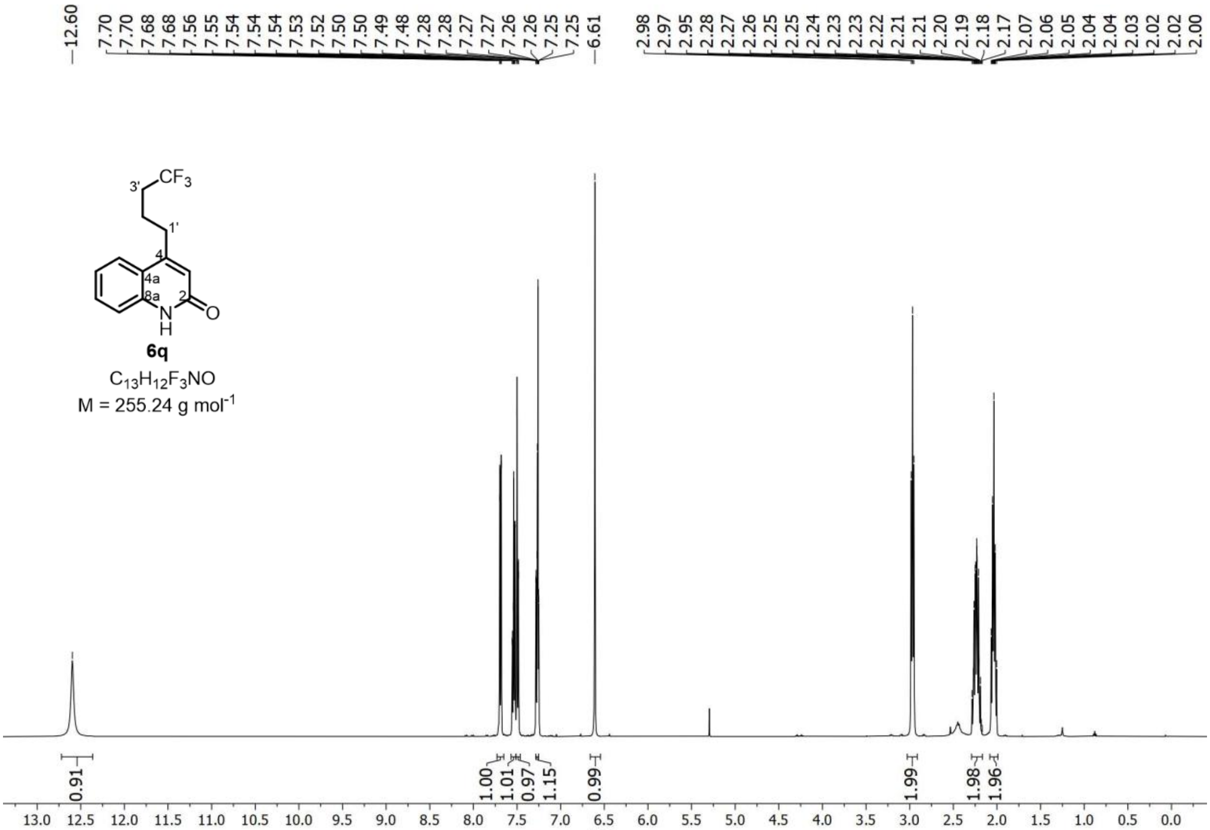

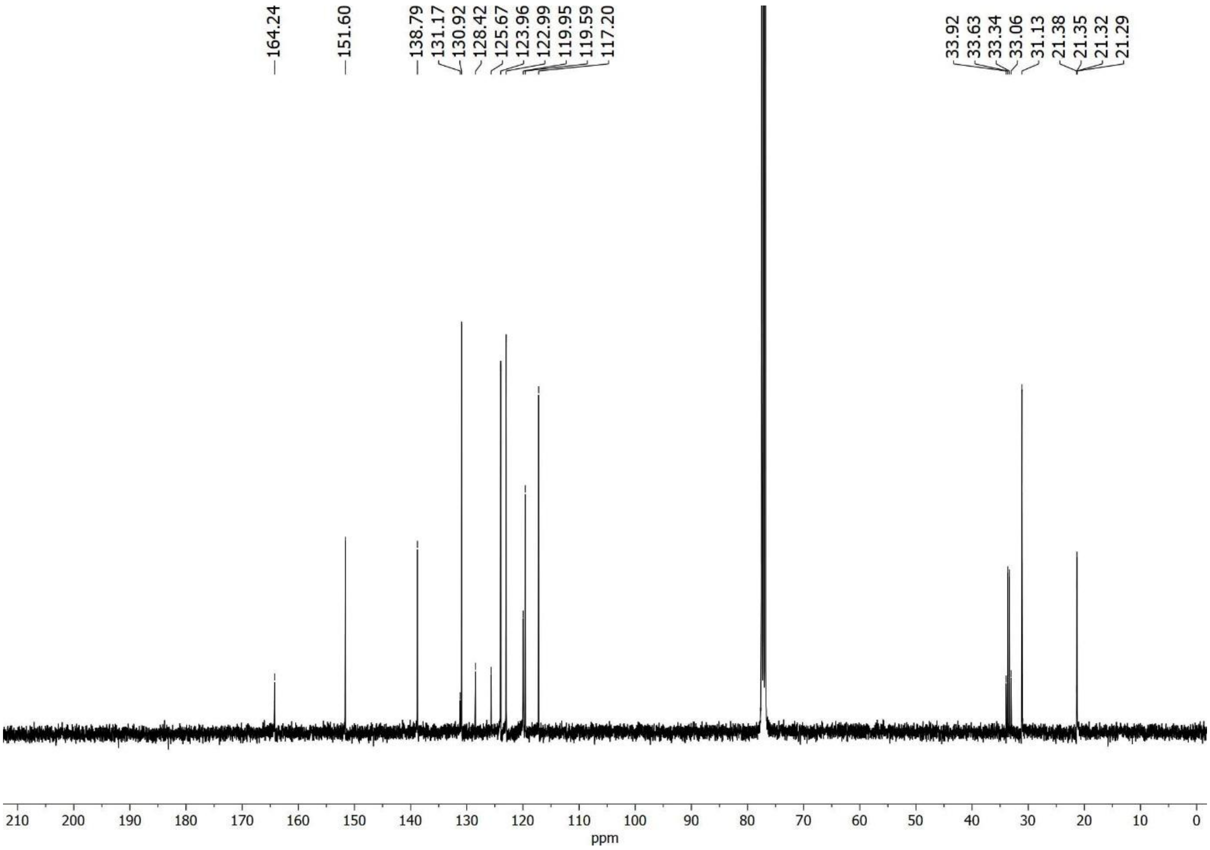

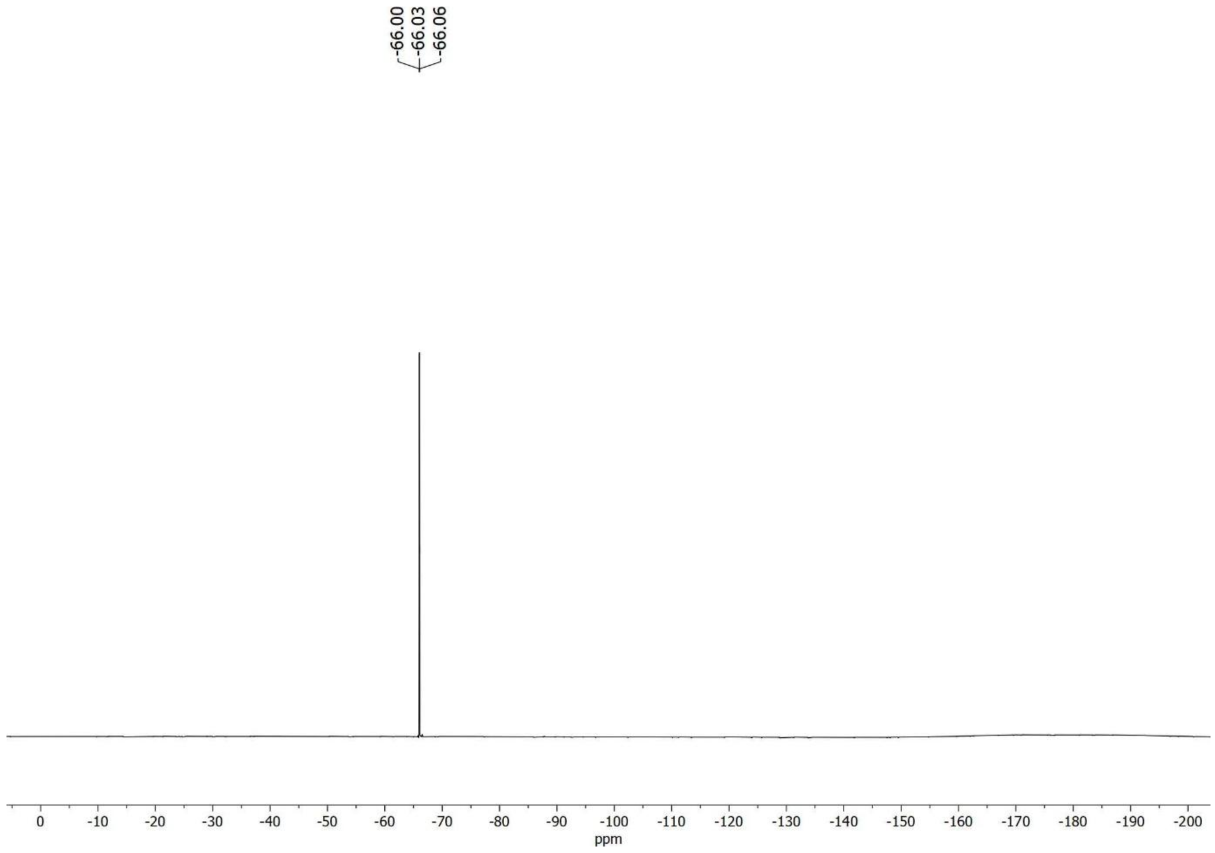

-66.00
-66.03
-66.06
0 -10 -20 -30 -40 -50 -60 -70 -80 -90 -100 -110 -120 -130 -140 -150 -160 -170 -180 -190 -200
ppm

4-(2-(Trimethylsilyl)ethyl)quinolin-2(1*H*)-one (6r)

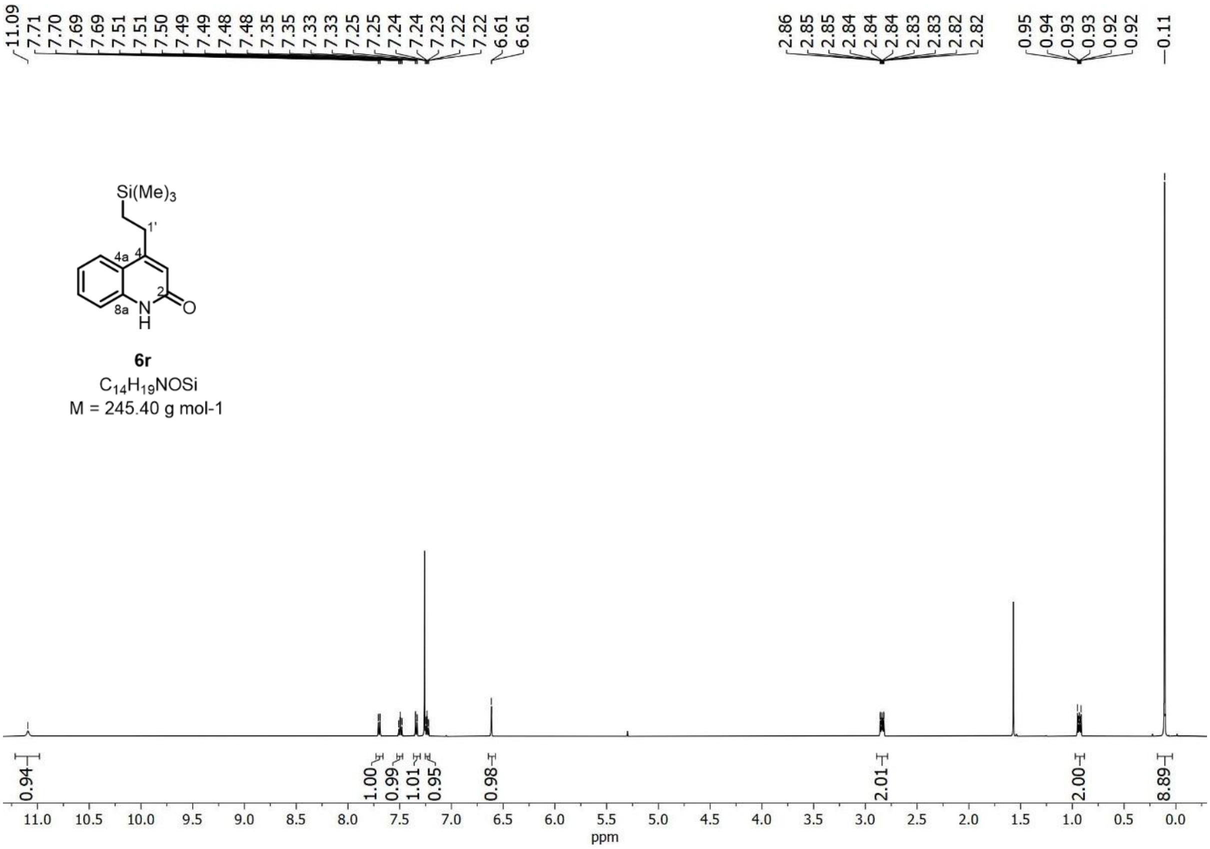

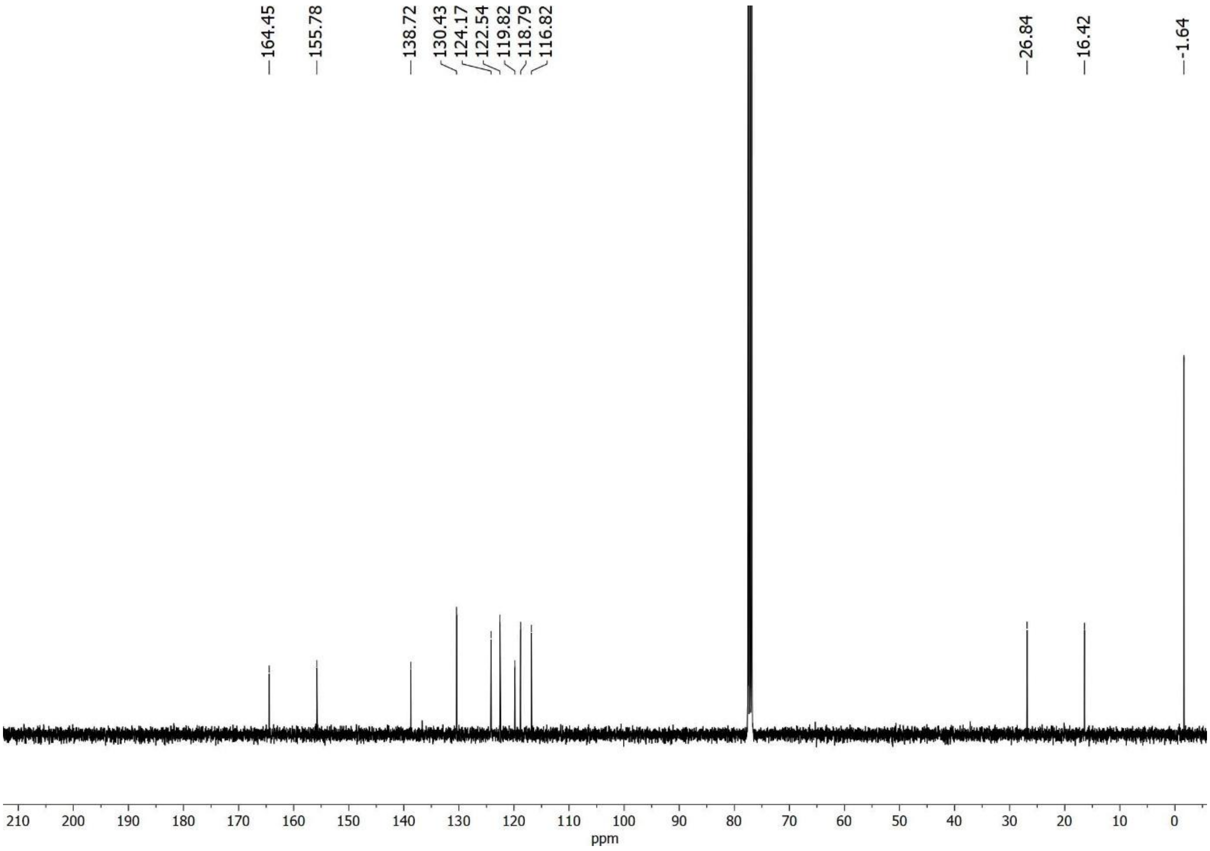

Butyl 2-oxo-1,2-dihydroquinoline-4-carboxylate (6s)

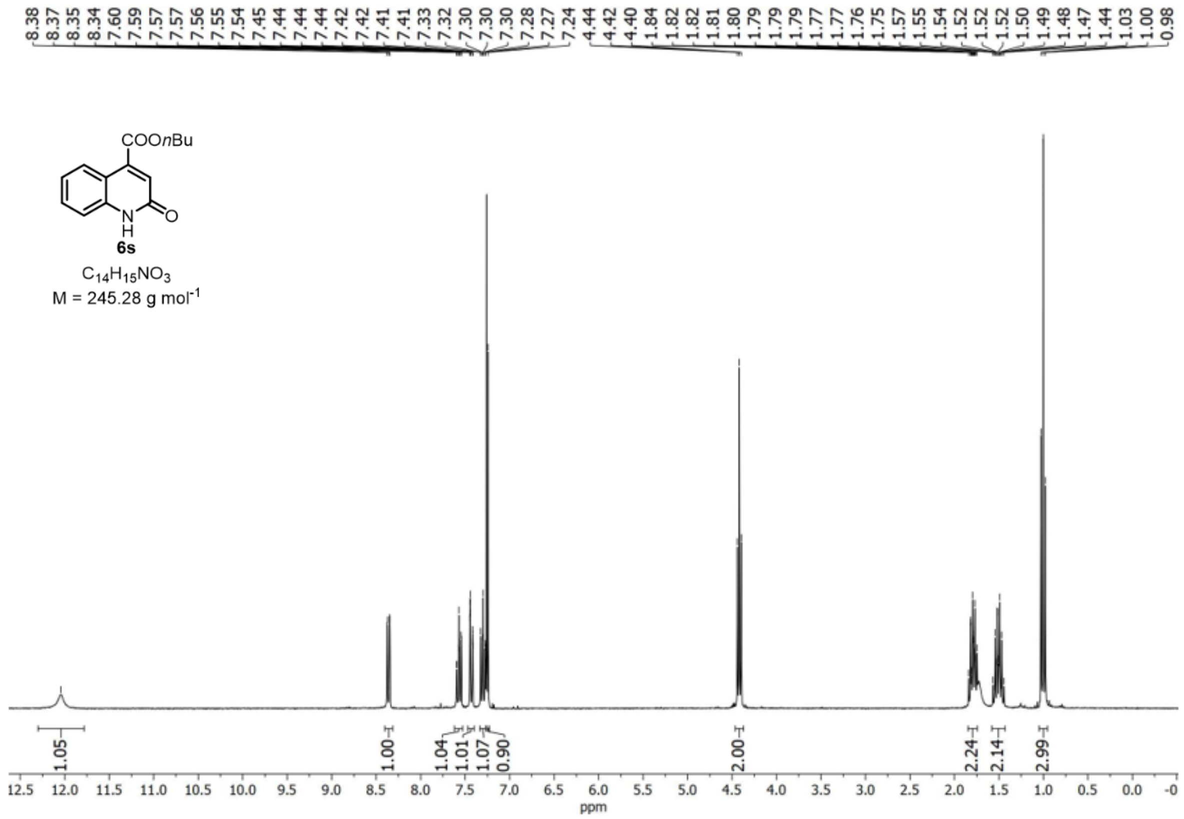

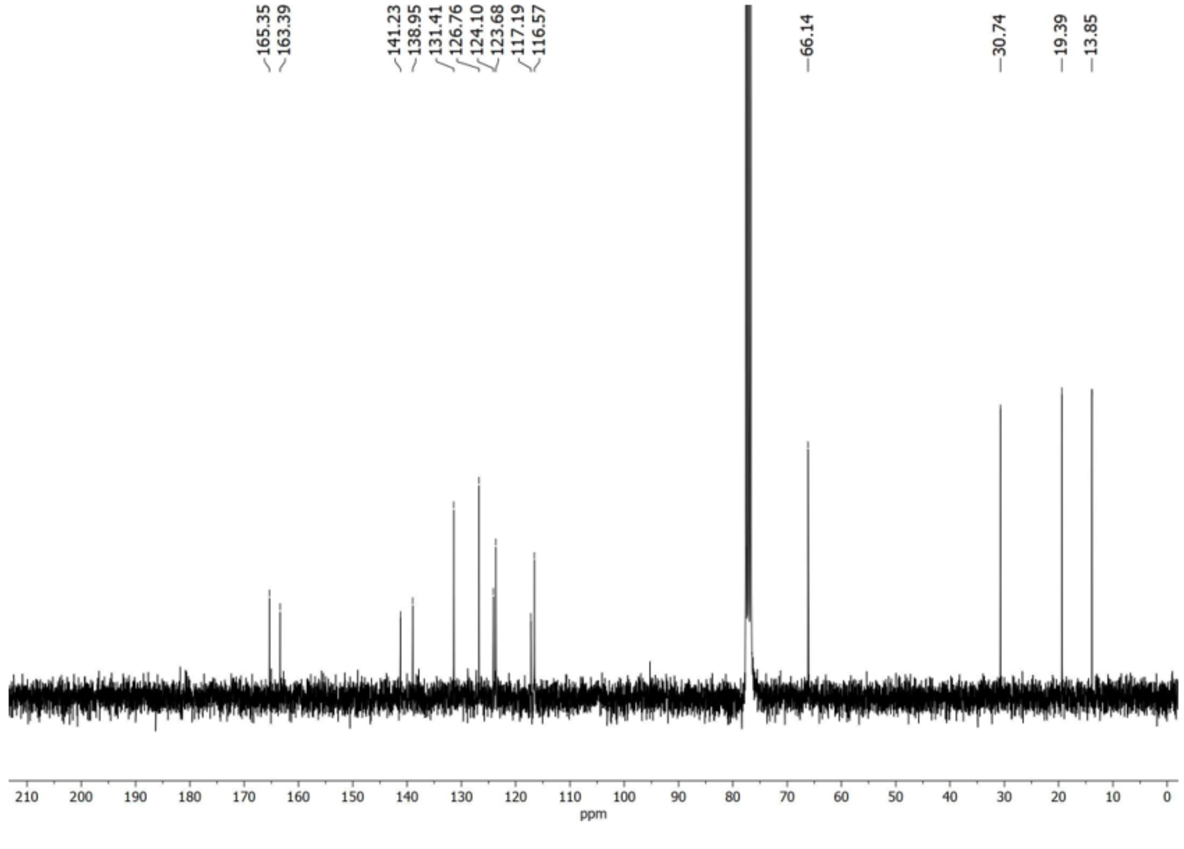

1-Methyl-4-phenylquinolin-2(1*H*)-one (11)

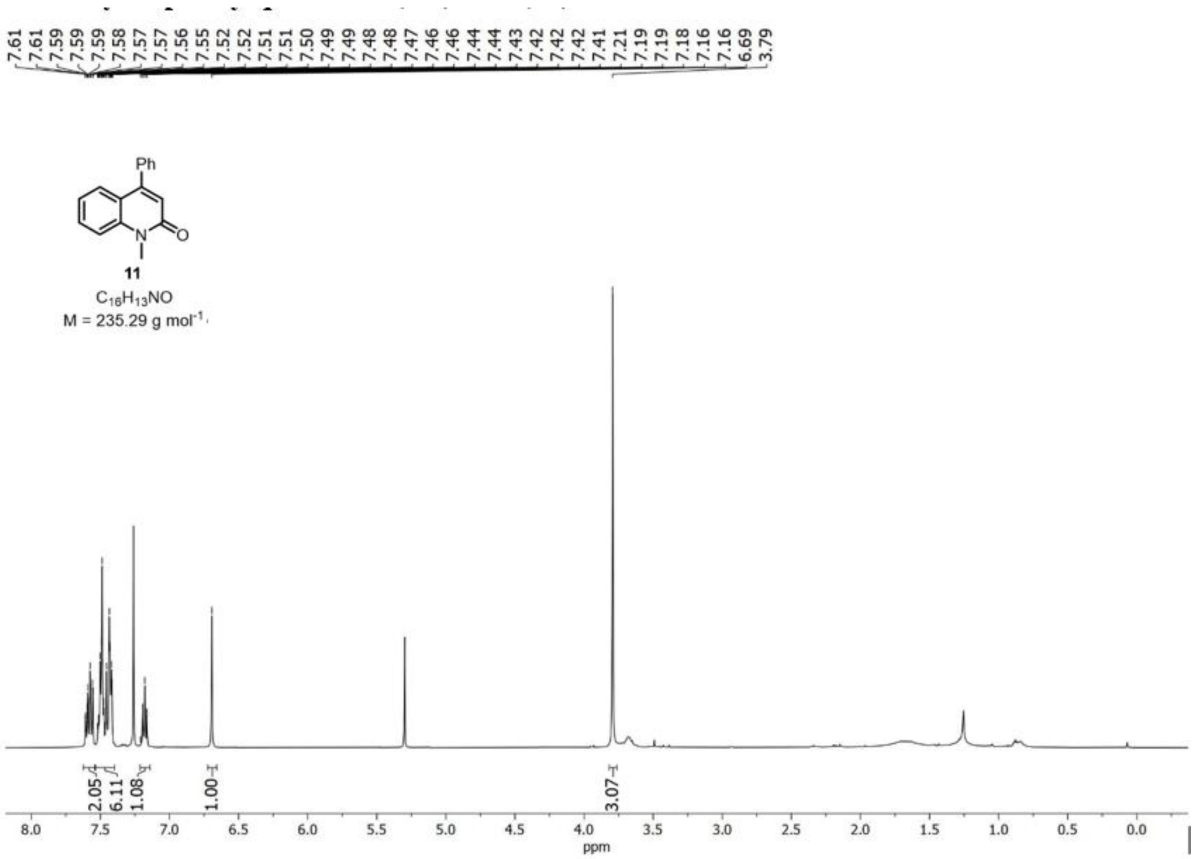

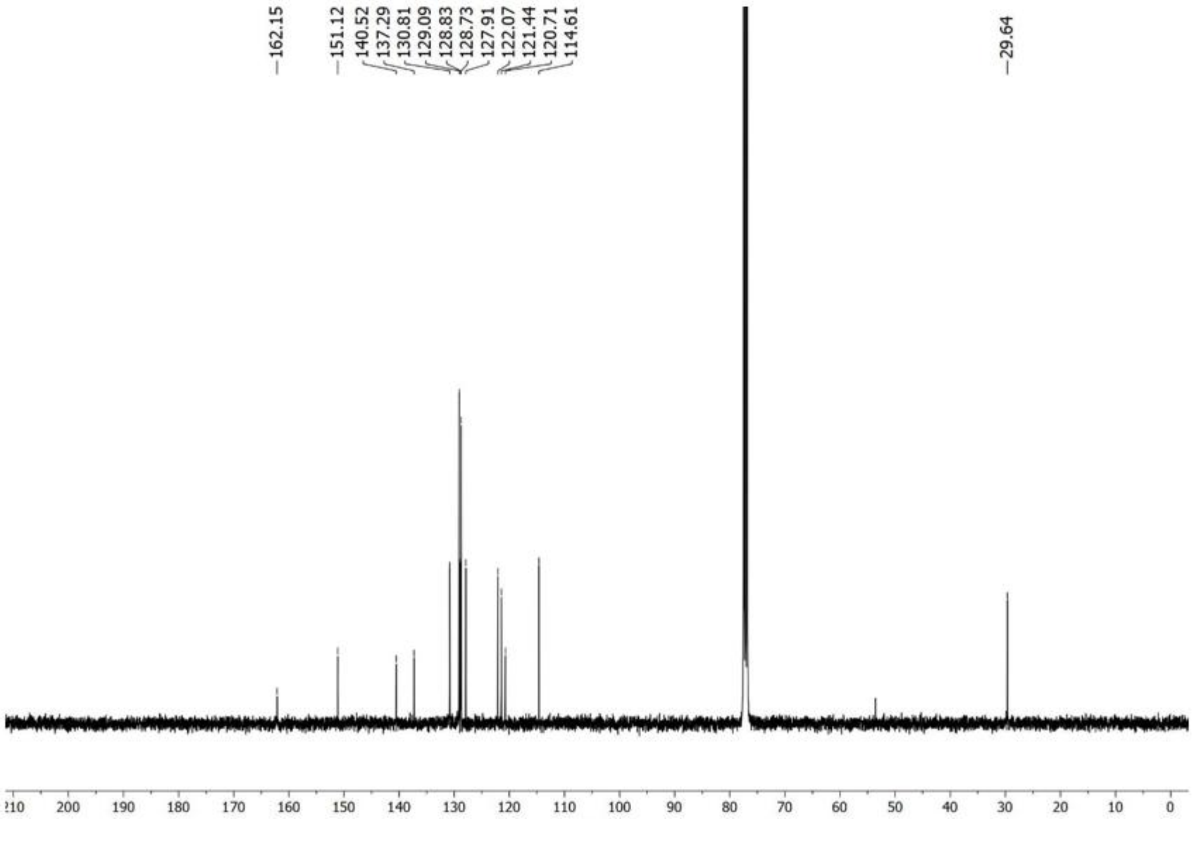

(*3S*,*4R*)-3-Phenyl-4-phenyl-3,4-dihydroquinolin-2(1*H*)-one (12)

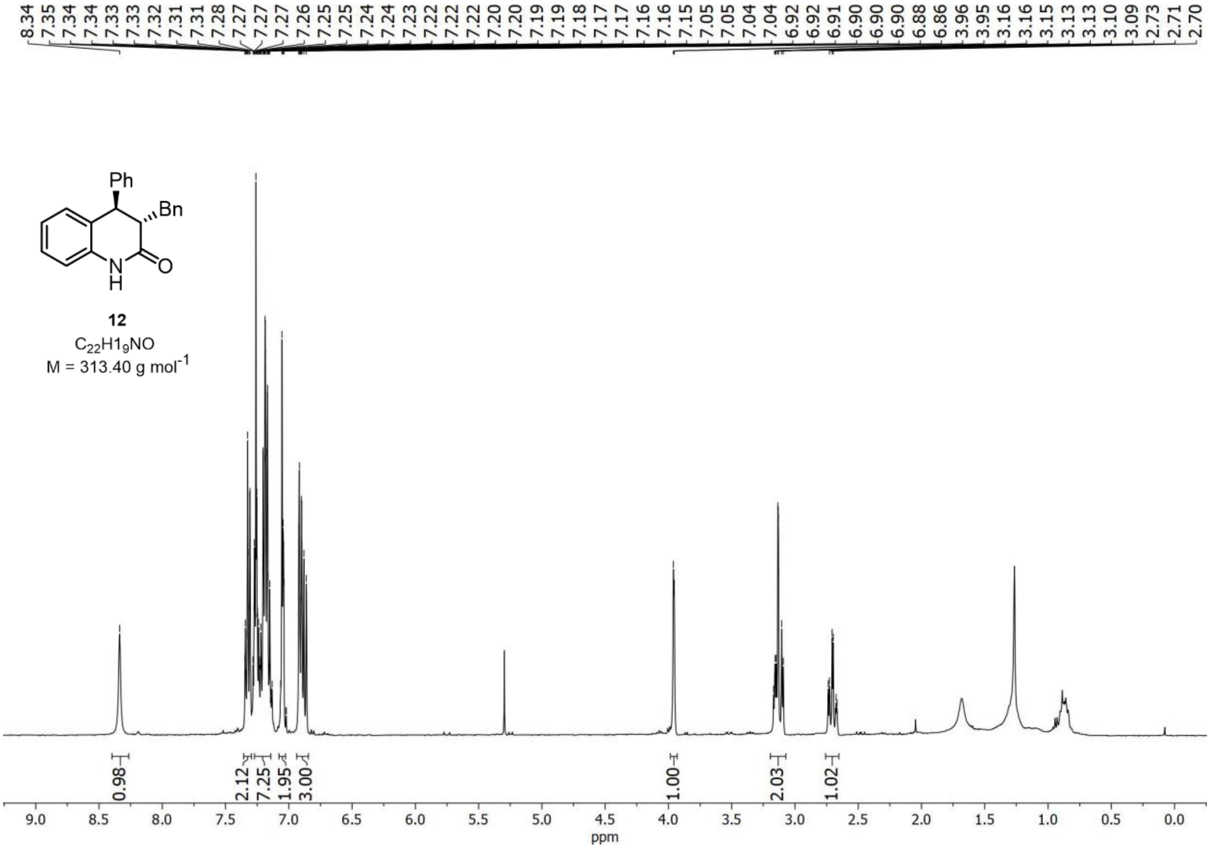

12
$C_{22}H1_{9}NO$
M = 313.40 g mol^{-1}

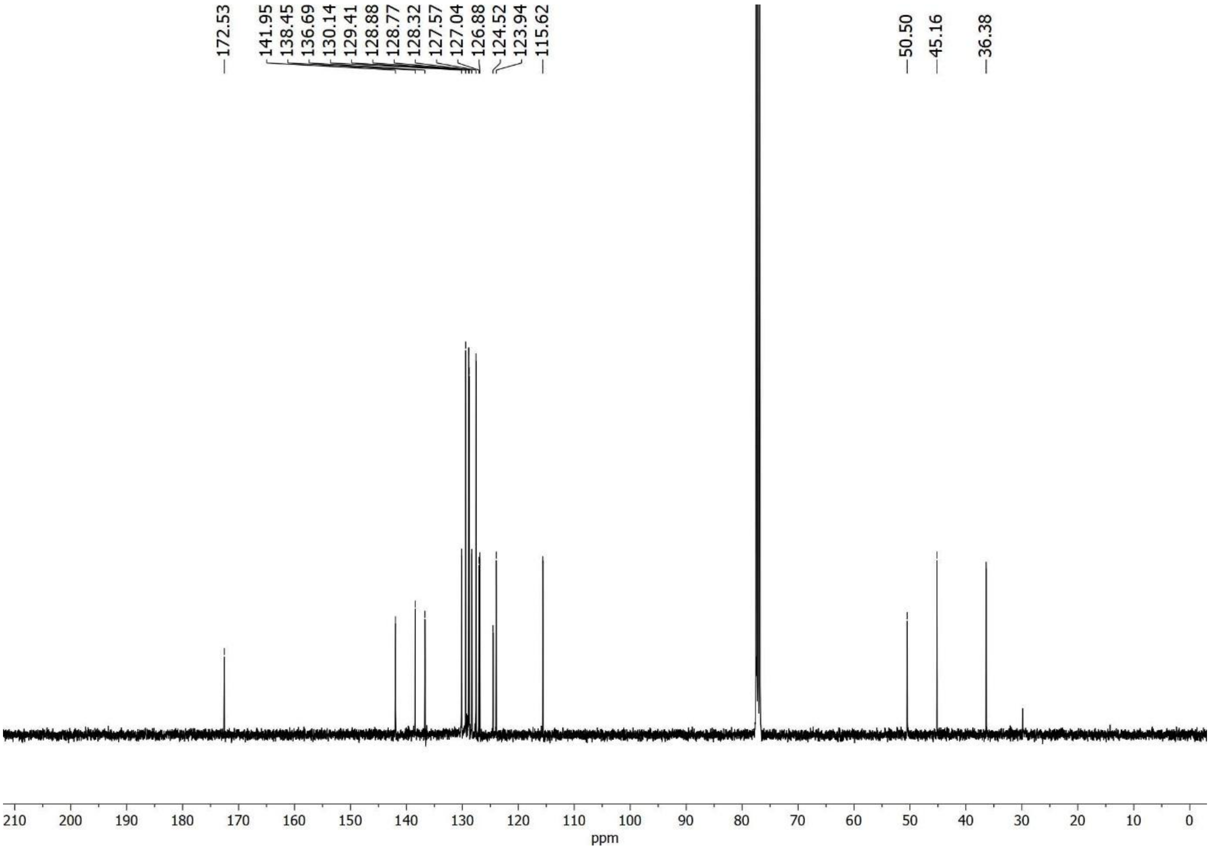

(*R*)-4-Phenyl-1,2,3,4-tetrahydroquinoline (13)

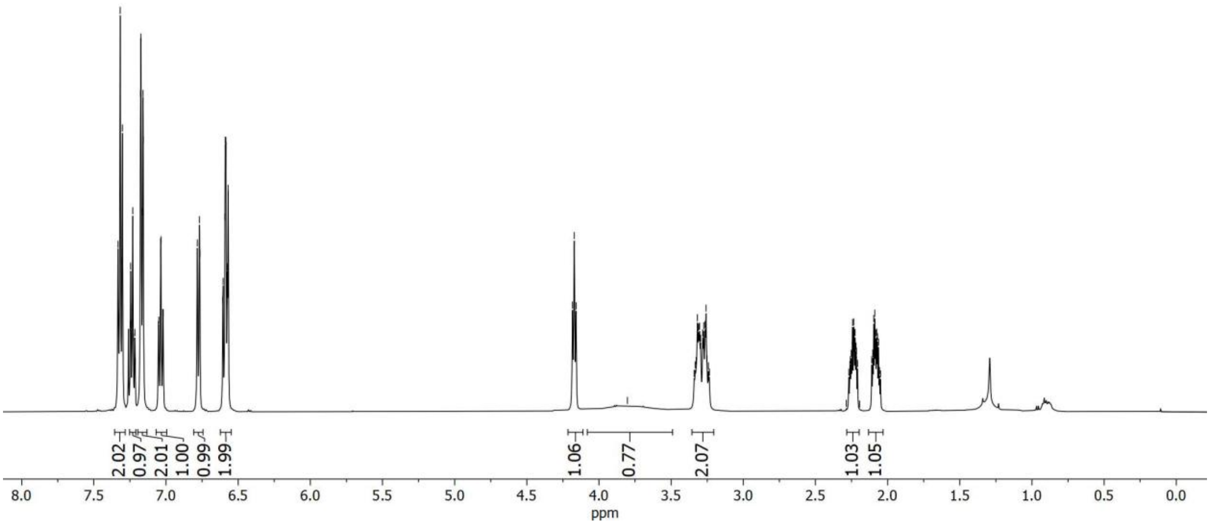

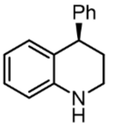

13

$C_{15}H_{15}O$

M = 209.29 g mol^{-1}

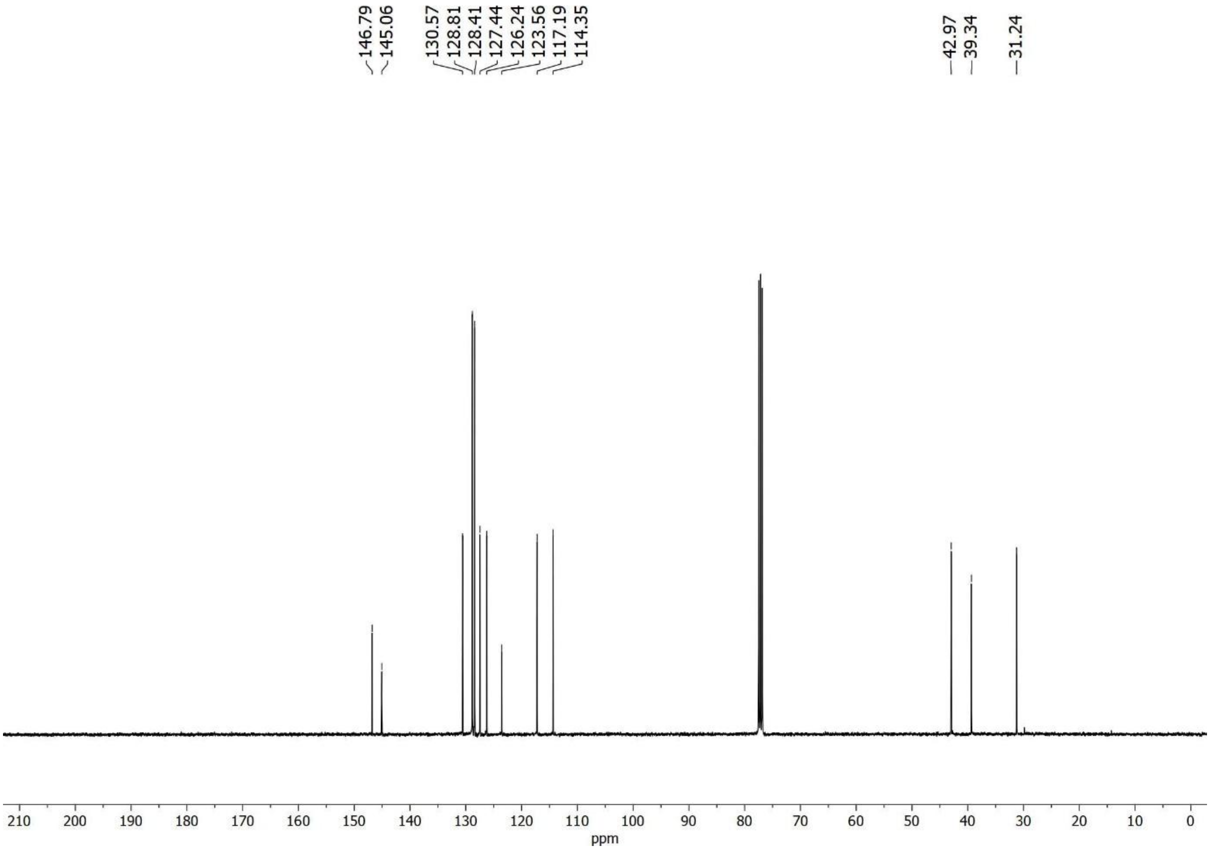

(*R*)-1,4-Diphenyl-3,4-dihydroquinolin-2(1*H*)-one (14)

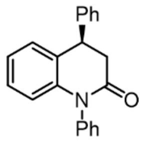

14

$C_{21}H_{17}NO$

M = 299.37 g mol^{-1}

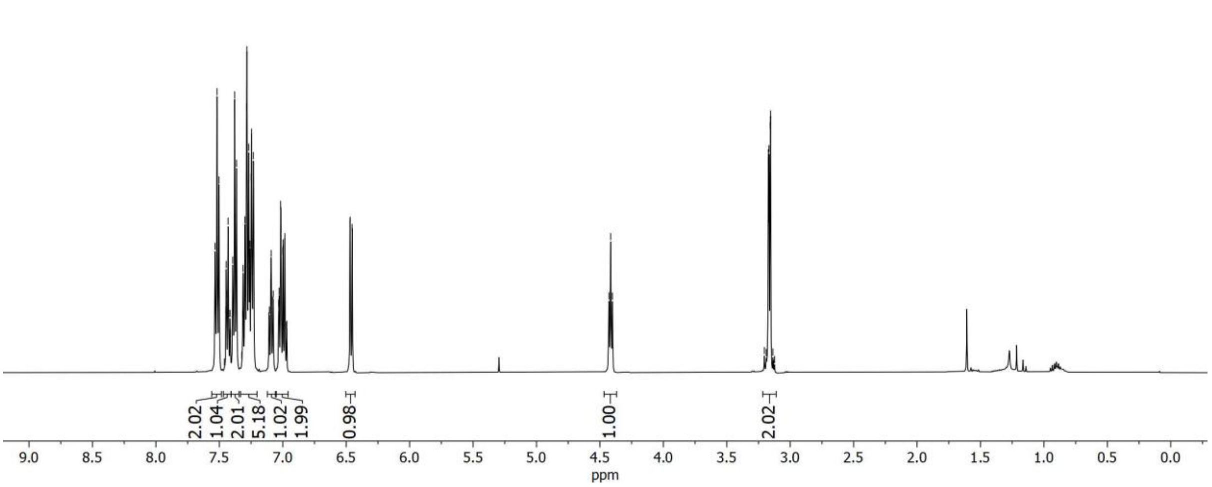

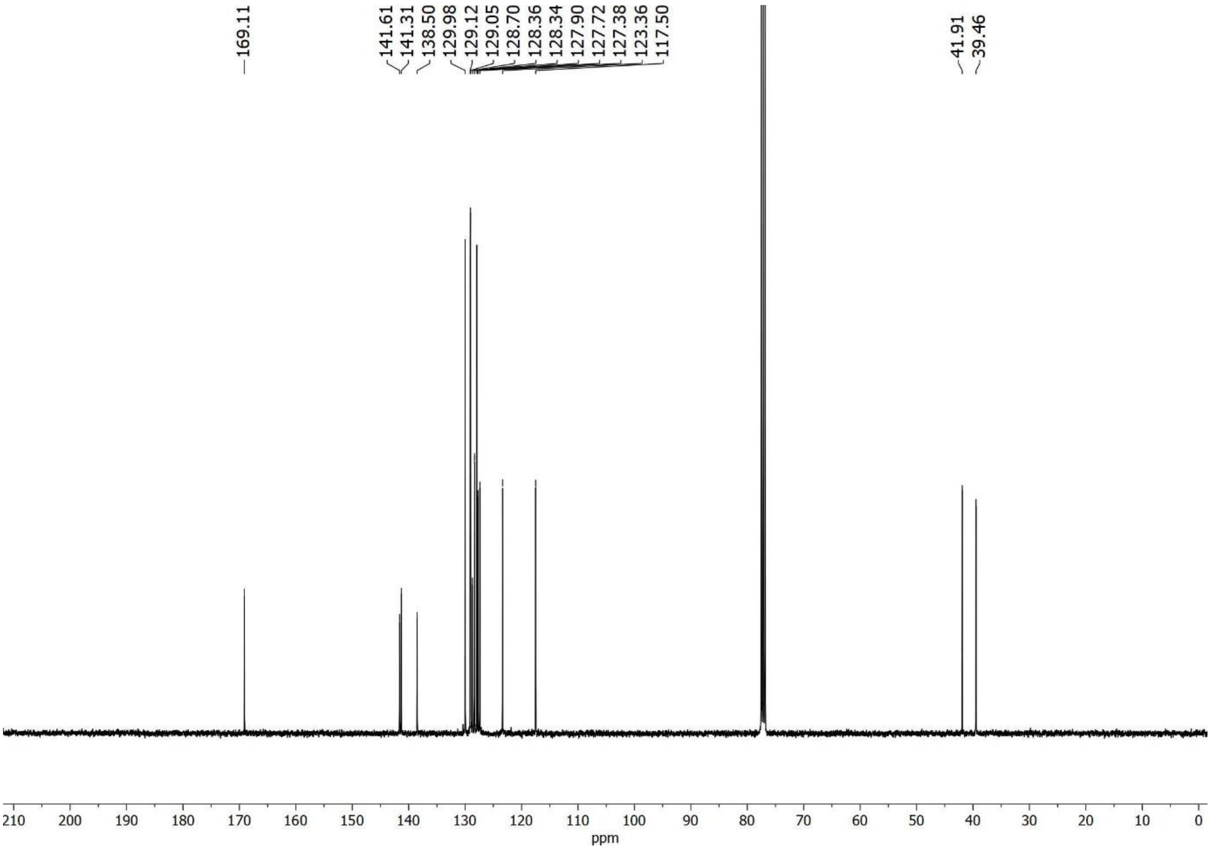

(*R*)-4-Phenyl-3,4-dihydroquinoline-2(1*H*)-thione (15)

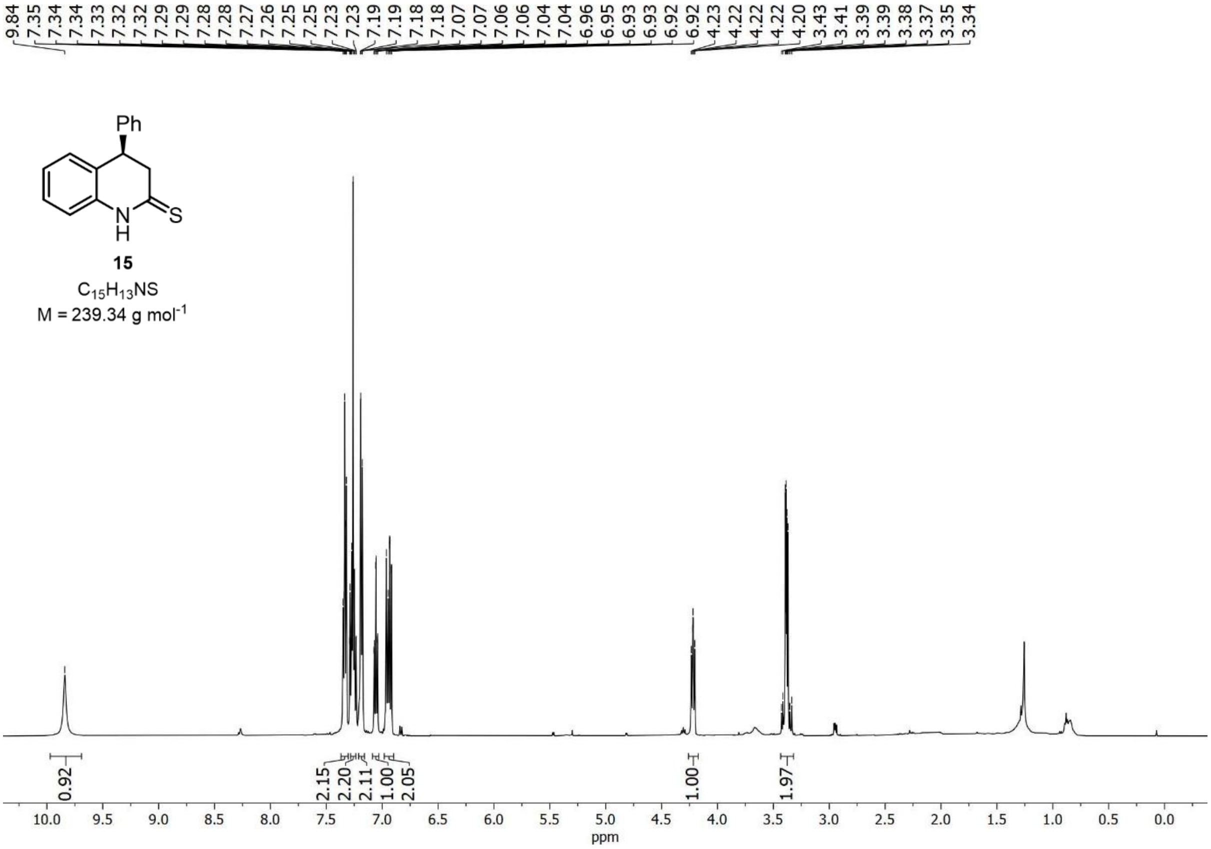

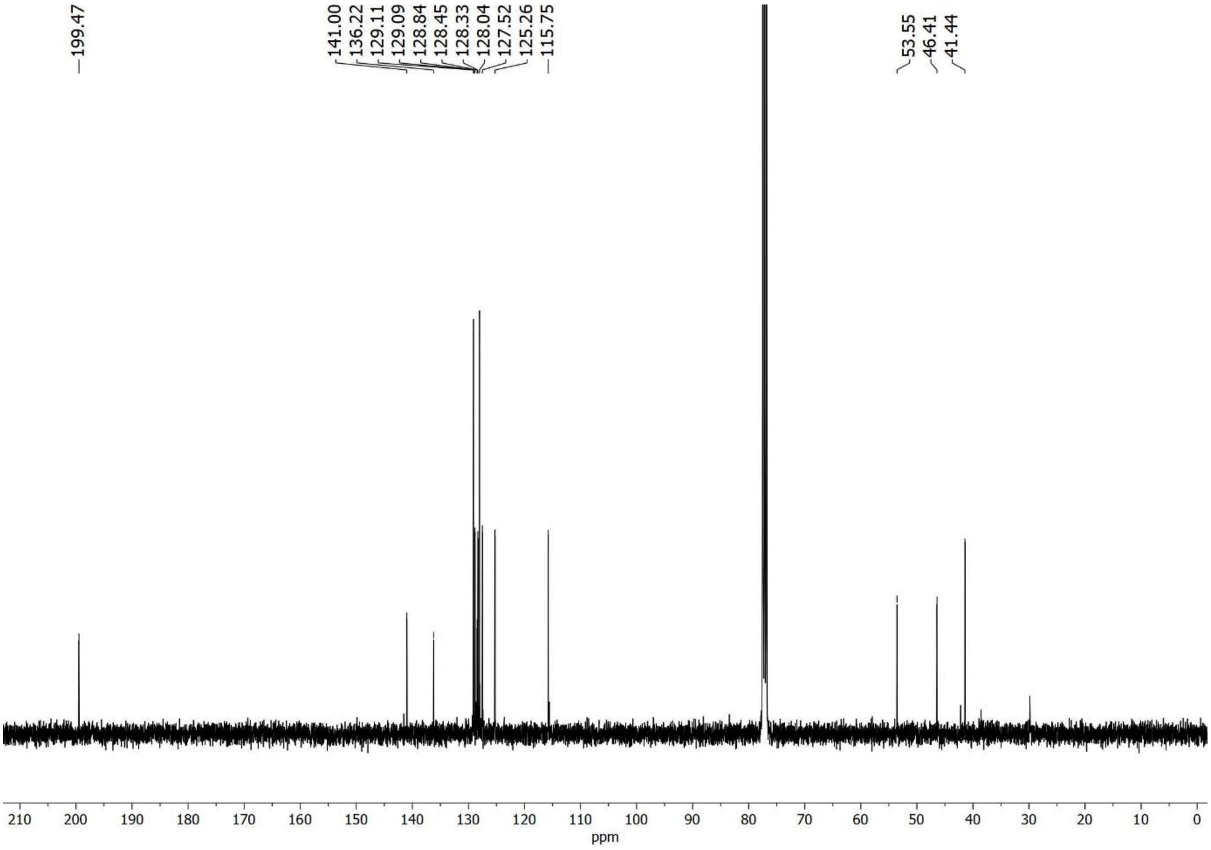

9. Chiral HPLC Traces

(*R*)-4-Phenyl-3,4-dihydroquinolin-2(1*H*)-one (1a)

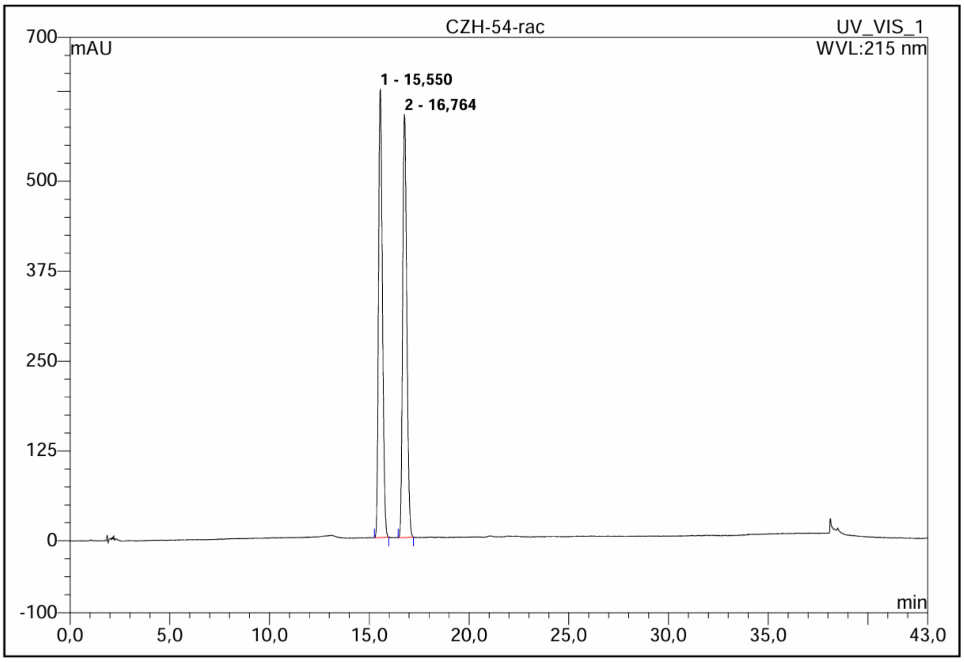

No.	Ret.Time min	Peak Name	Height mAU	Area mAU*min	Rel.Area %	Amount	Type
1	15,55	n.a.	623,778	142,095	49,93	n.a.	BMB
2	16,76	n.a.	588,238	142,498	50,07	n.a.	BMB
Total:			1212,016	284,593	100,00	0,000	

Condition A:

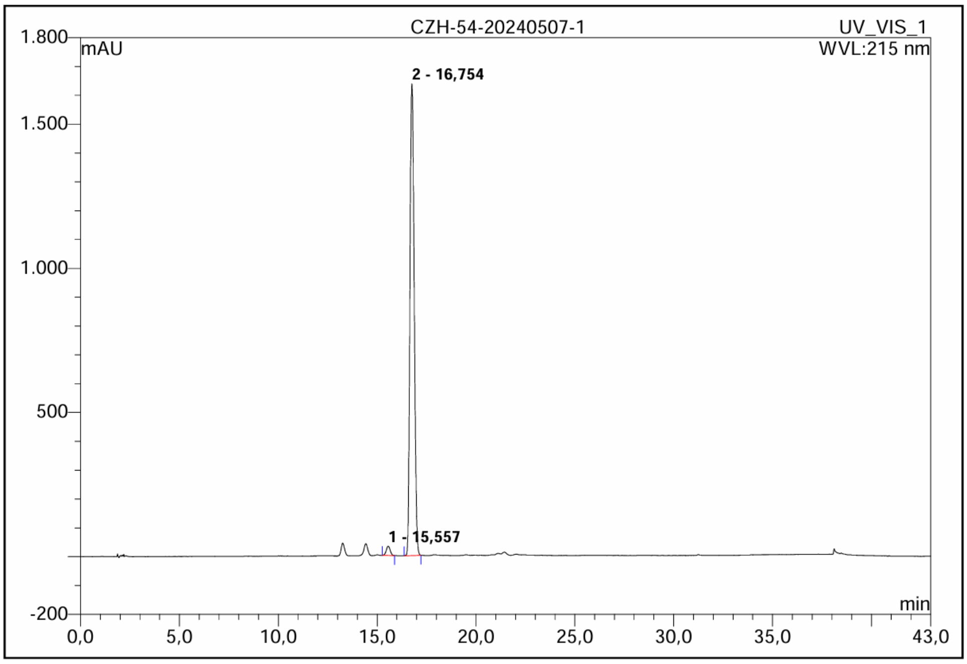

No.	Ret.Time min	Peak Name	Height mAU	Area mAU*min	Rel.Area %	Amount	Type
1	15,56	n.a.	32,426	7,448	1,79	n.a.	BMB*
2	16,75	n.a.	1636,517	407,656	98,21	n.a.	BMB
Total:			1668,942	415,104	100,00	0,000	

Condition B:

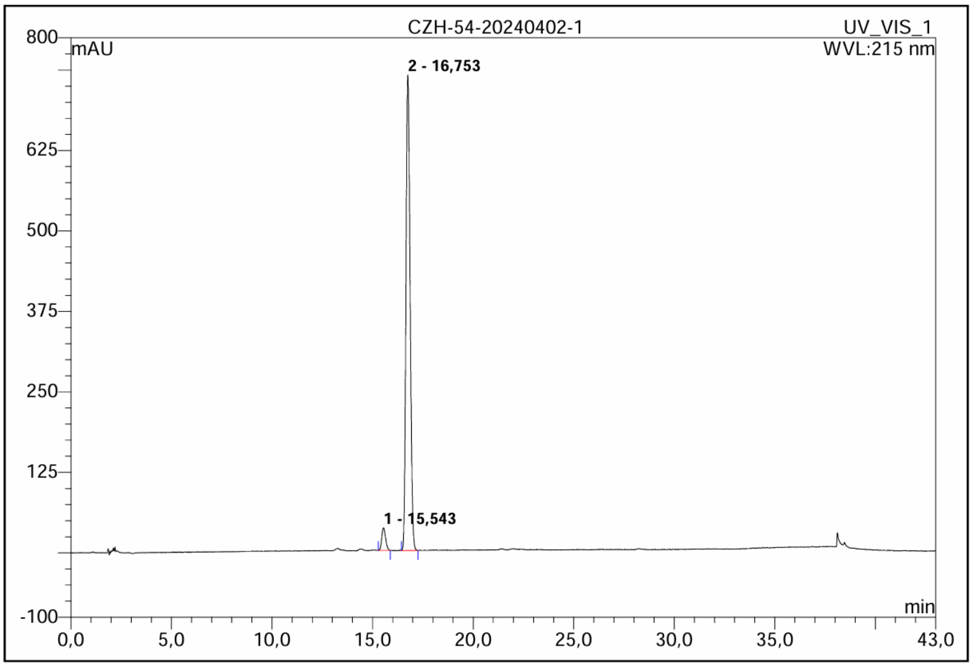

No.	Ret.Time min		Peak Name	Height mAU	Area mAU*min	Rel.Area %	Amount	Type
1	15,54	n.a.		34,882	7,700	4,15	n.a.	BMB*
2	16,75	n.a.		738,549	177,656	95,85	n.a.	BMB
Total:				773,430	185,355	100,00	0,000	

0.5 mmol scale:

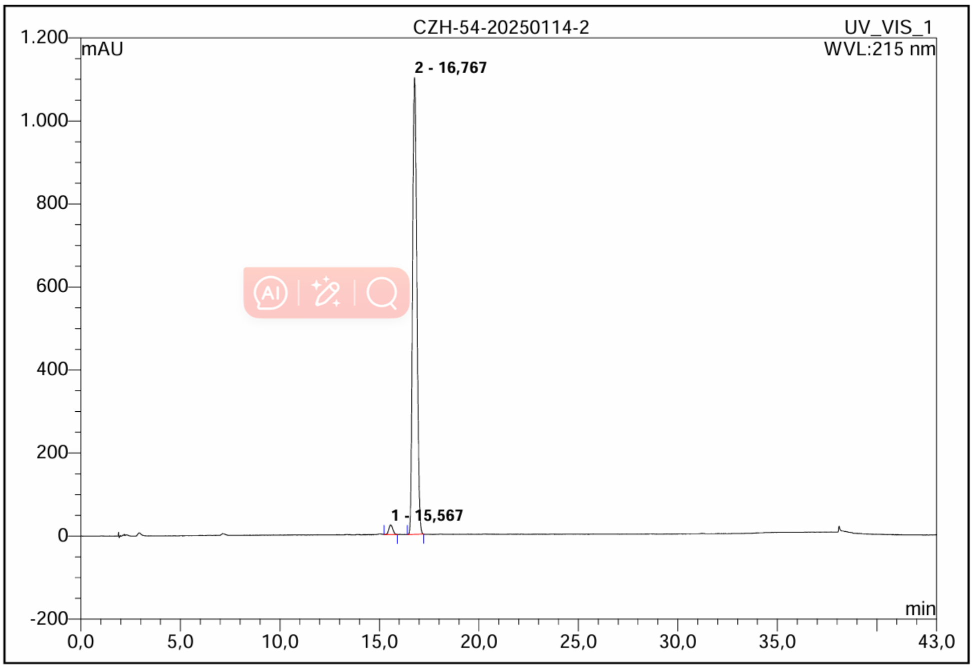

No.	Ret.Time min		Peak Name	Height mAU	Area mAU*min	Rel.Area %	Amount	Type
1	15,57	n.a.		23,505	5,368	1,91	n.a.	BMB*
2	16,77	n.a.		1100,380	275,906	98,09	n.a.	BMB
Total:				1123,886	281,274	100,00	0,000	

(*R*)-4-(4-Methoxyphenyl)-3,4-dihydroquinolin-2(1*H*)-one (1b)

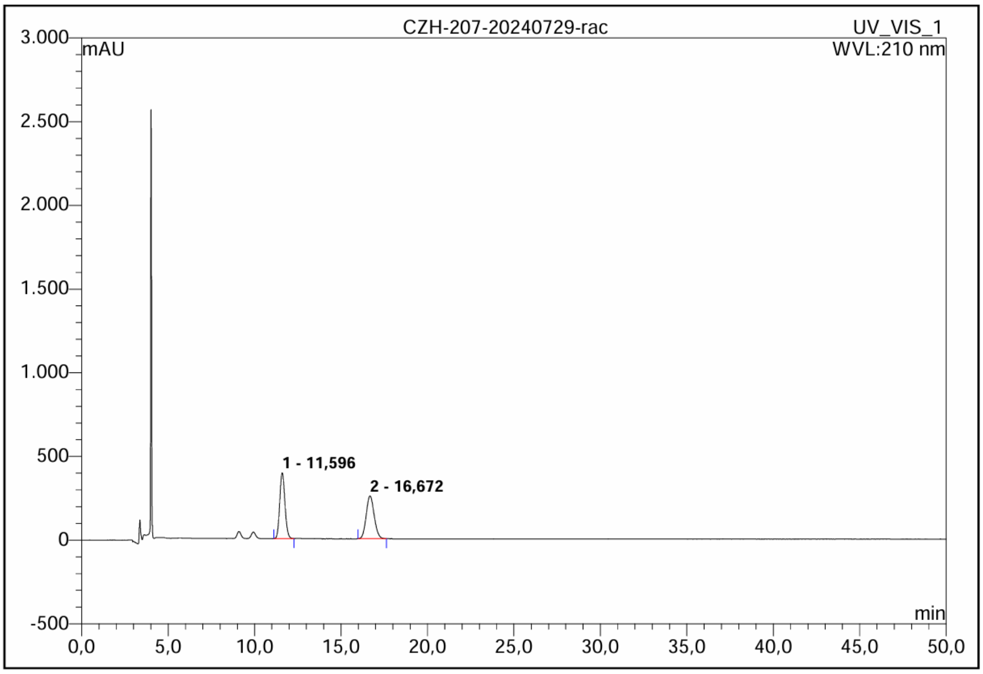

No.	Ret.Time min	Peak Name	Height mAU	Area mAU*min	Rel.Area %	Amount	Type
1	11,60	n.a.	393,038	137,522	50,02	n.a.	BMB
2	16,67	n.a.	255,974	137,405	49,98	n.a.	BMB
Total:			649,012	274,926	100,00	0,000	

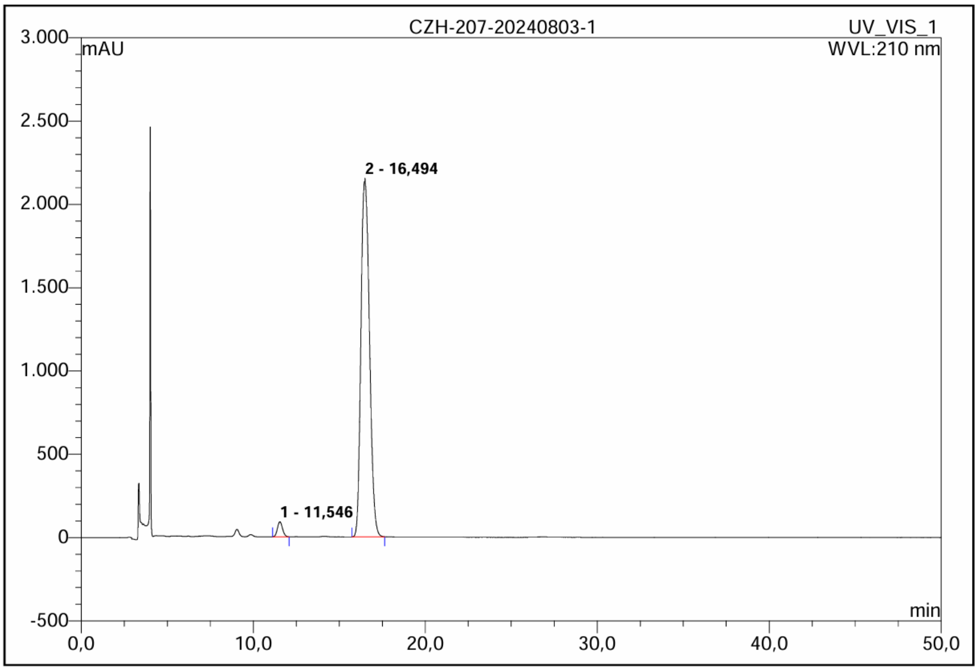

No.	Ret.Time min	Peak Name	Height mAU	Area mAU*min	Rel.Area %	Amount	Type
1	11,55	n.a.	90,142	31,259	2,47	n.a.	BMB
2	16,49	n.a.	2154,078	1234,836	97,53	n.a.	BMB
Total:			2244,219	1266,094	100,00	0,000	

(*R*)-4-(*p*-Tolyl)-3,4-dihydroquinolin-2(1*H*)-one (1c)

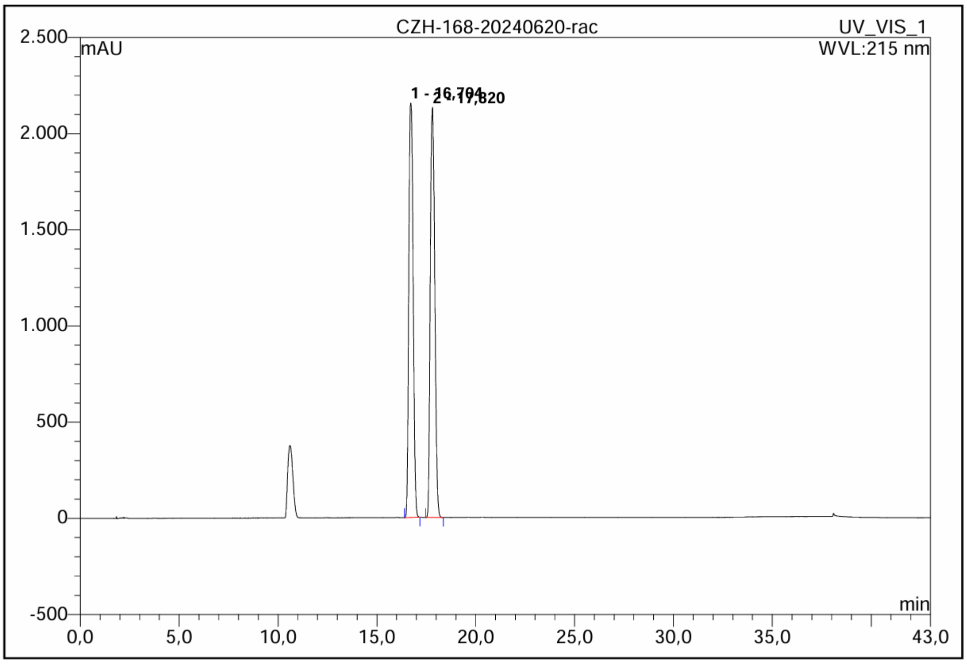

No.	Ret.Time min	Peak Name	Height mAU	Area mAU*min	Rel.Area %	Amount	Type
1	16,70	n.a.	2155,354	549,853	49,37	n.a.	BMB
2	17,82	n.a.	2132,569	563,908	50,63	n.a.	BMB
Total:			4287,923	1113,761	100,00	0,000	

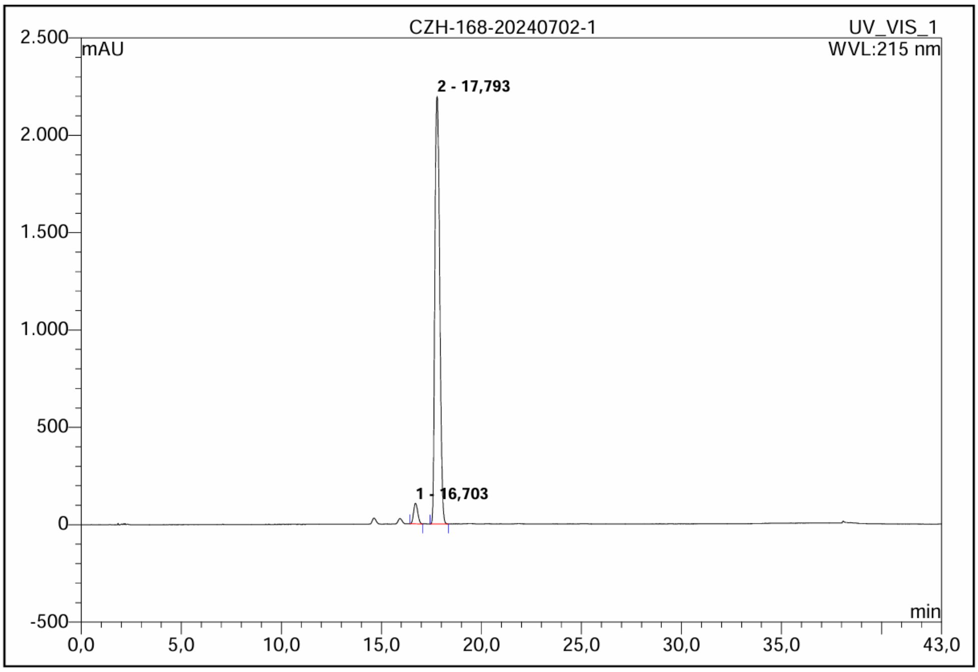

No.	Ret.Time min	Peak Name	Height mAU	Area mAU*min	Rel.Area %	Amount	Type
1	16,70	n.a.	105,528	25,259	3,94	n.a.	BMB
2	17,79	n.a.	2197,211	615,115	96,06	n.a.	BMB
Total:			2302,739	640,374	100,00	0,000	

(*R*)-4-(4-Fluorophenyl)-3,4-dihydroquinolin-2(1*H*)-one (1d)

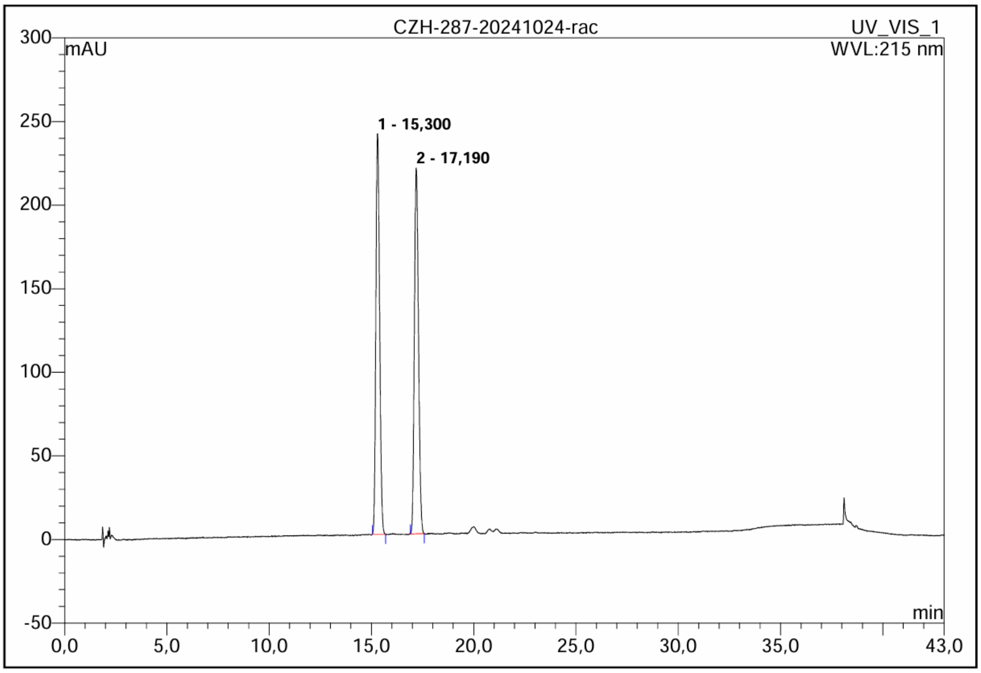

No.	Ret.Time min	Peak Name	Height mAU	Area mAU*min	Rel.Area %	Amount	Type
1	15,30	n.a.	239,655	50,858	50,08	n.a.	BMB
2	17,19	n.a.	218,758	50,700	49,92	n.a.	BMB
Total:			458,413	101,558	100,00	0,000	

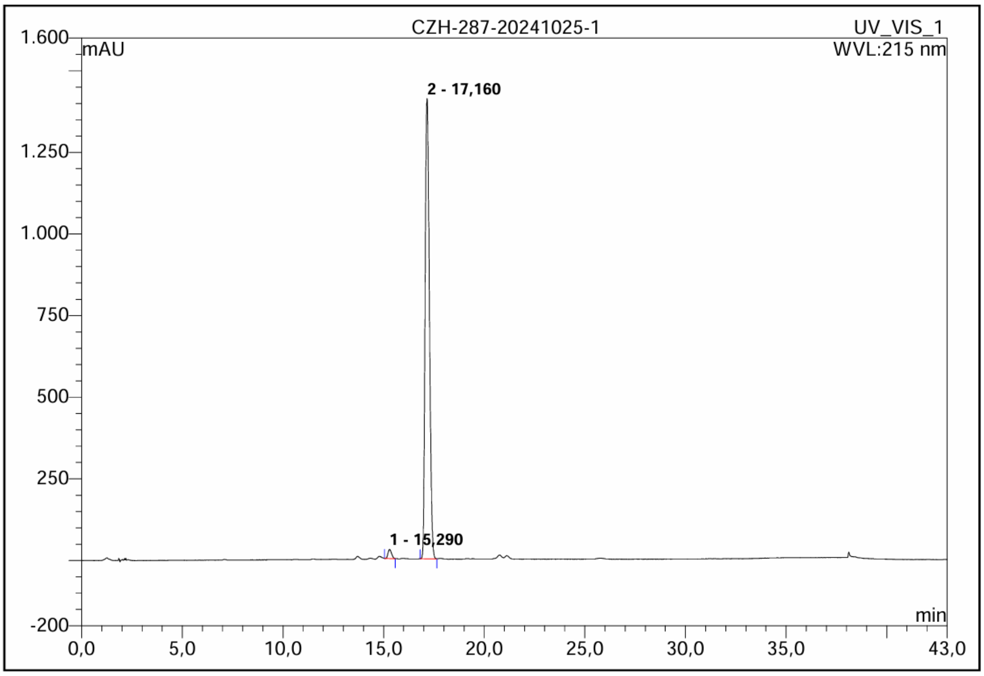

No.	Ret.Time min	Peak Name	Height mAU	Area mAU*min	Rel.Area %	Amount	Type
1	15,29	n.a.	27,778	5,688	1,61	n.a.	BMB*
2	17,16	n.a.	1409,657	346,783	98,39	n.a.	BMB
Total:			1437,435	352,471	100,00	0,000	

(*R*)-4-(4-Chlorophenyl)-3,4-dihydroquinolin-2(1*H*)-one (1e)

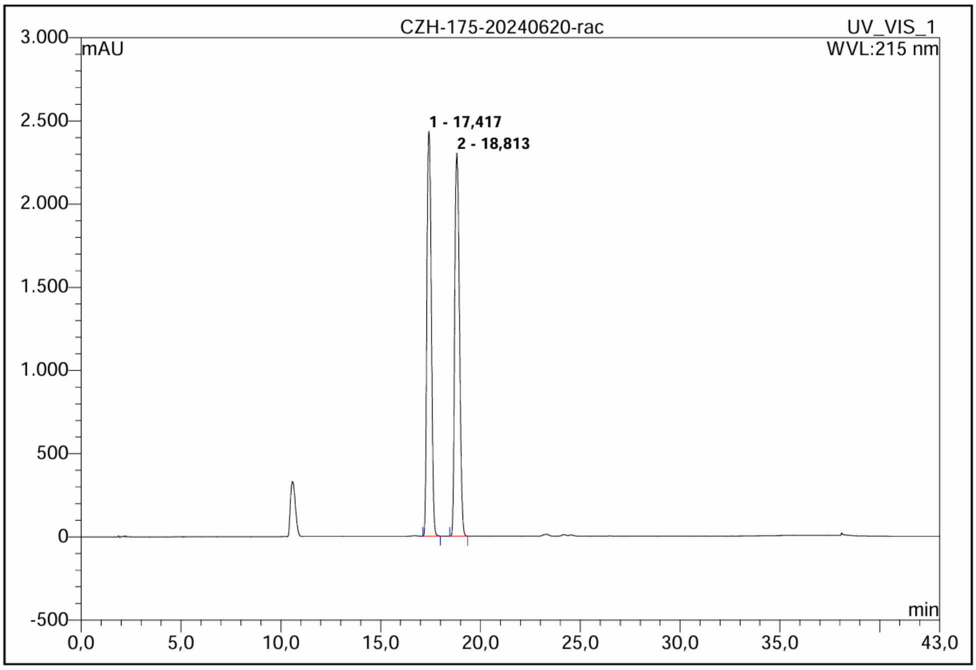

No.	Ret.Time min	Peak Name	Height mAU	Area mAU*min	Rel.Area %	Amount	Type
1	17,42	n.a.	2433,628	625,845	49,44	n.a.	BMB
2	18,81	n.a.	2302,983	639,915	50,56	n.a.	BMB
Total:			4736,611	1265,760	100,00	0,000	

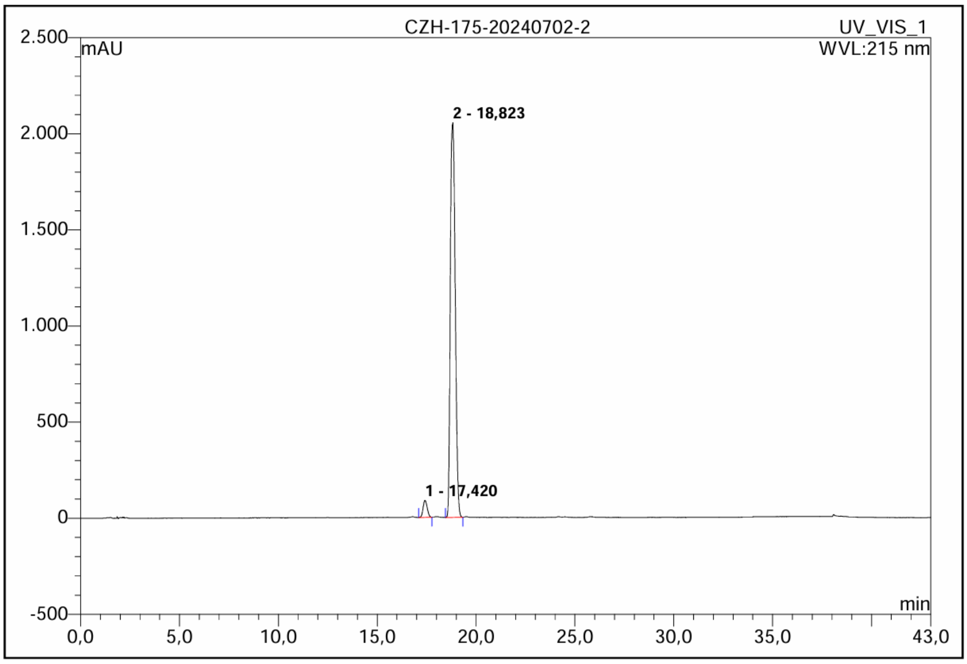

No.	Ret.Time min	Peak Name	Height mAU	Area mAU*min	Rel.Area %	Amount	Type
1	17,42	n.a.	88,125	21,160	3,43	n.a.	BMB
2	18,82	n.a.	2053,573	595,457	96,57	n.a.	BMB
Total:			2141,697	616,617	100,00	0,000	

(*R*)-4-(4-Bromophenyl)-3,4-dihydroquinolin-2(1*H*)-one (1f)

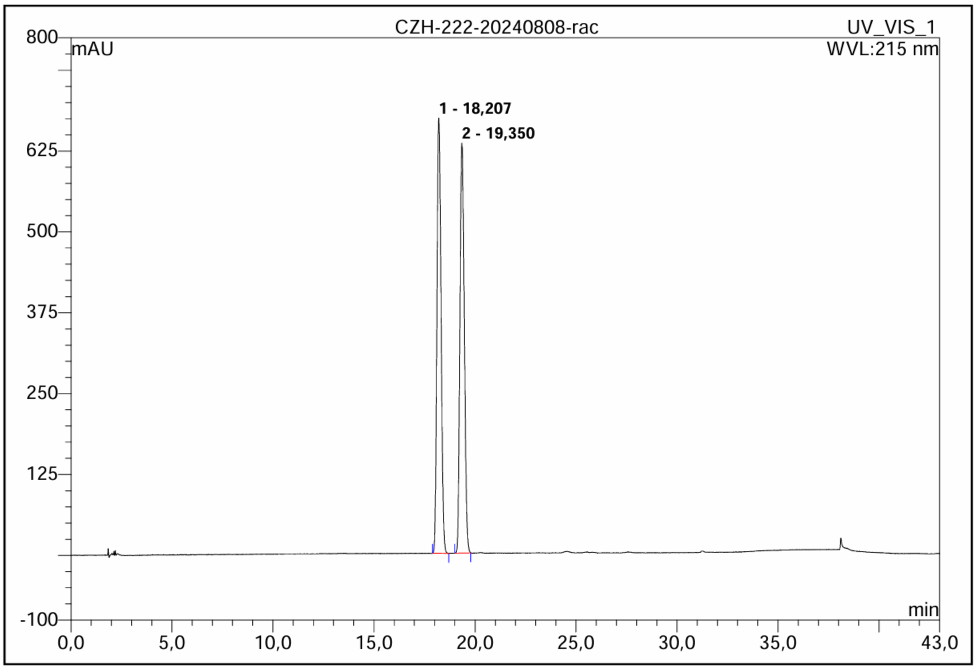

No.	Ret.Time min	Peak Name	Height mAU	Area mAU*min	Rel.Area %	Amount	Type
1	18,21	n.a.	672,682	159,316	49,98	n.a.	BMB
2	19,35	n.a.	633,496	159,457	50,02	n.a.	BMB
Total:			1306,178	318,774	100,00	0,000	

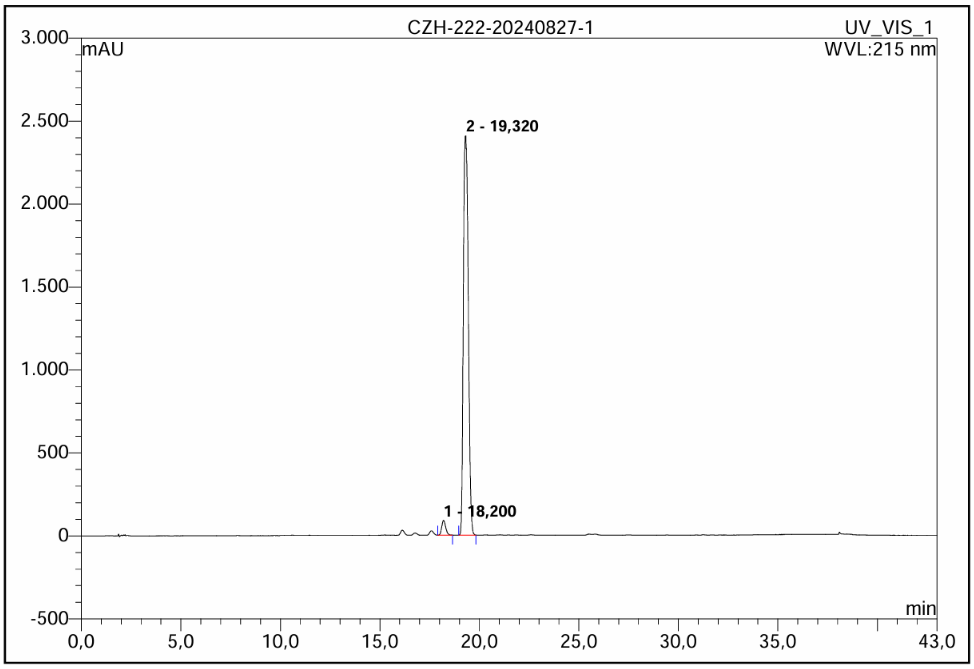

No.	Ret.Time min	Peak Name	Height mAU	Area mAU*min	Rel.Area %	Amount	Type
1	18,20	n.a.	88,618	20,677	2,91	n.a.	BMB
2	19,32	n.a.	2407,317	691,001	97,09	n.a.	BMB
Total:			2495,936	711,678	100,00	0,000	

(*R*)- 4-(3-Chlorophenyl)-3,4-dihydroquinolin-2(1*H*)-one (1g)

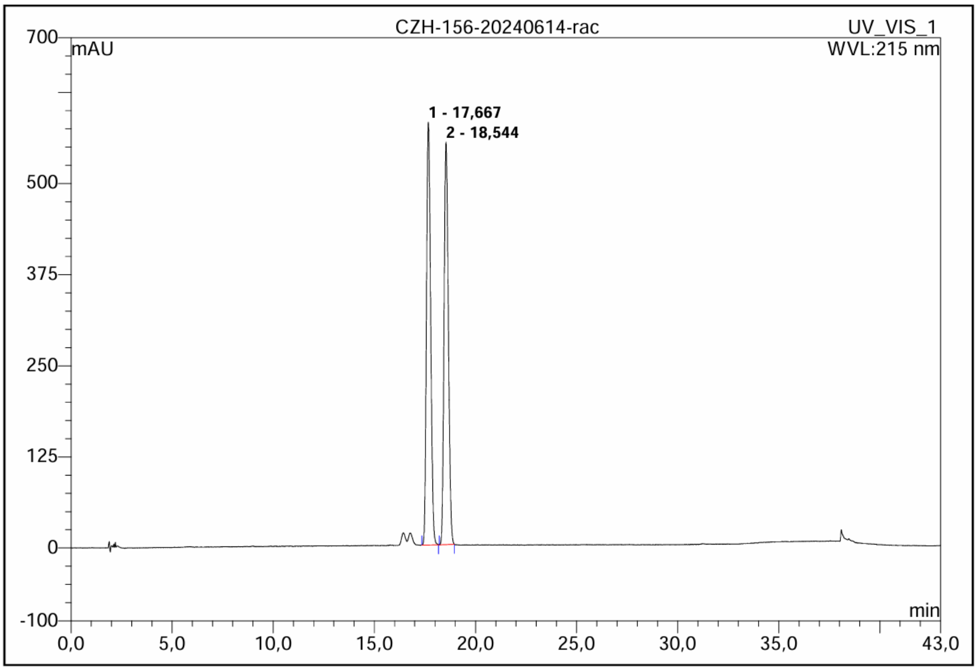

No.	Ret.Time min	Peak Name	Height mAU	Area mAU*min	Rel.Area %	Amount	Type
1	17,67	n.a.	579,794	138,251	50,19	n.a.	BMB
2	18,54	n.a.	552,286	137,190	49,81	n.a.	BMB
Total:			1132,080	275,442	100,00	0,000	

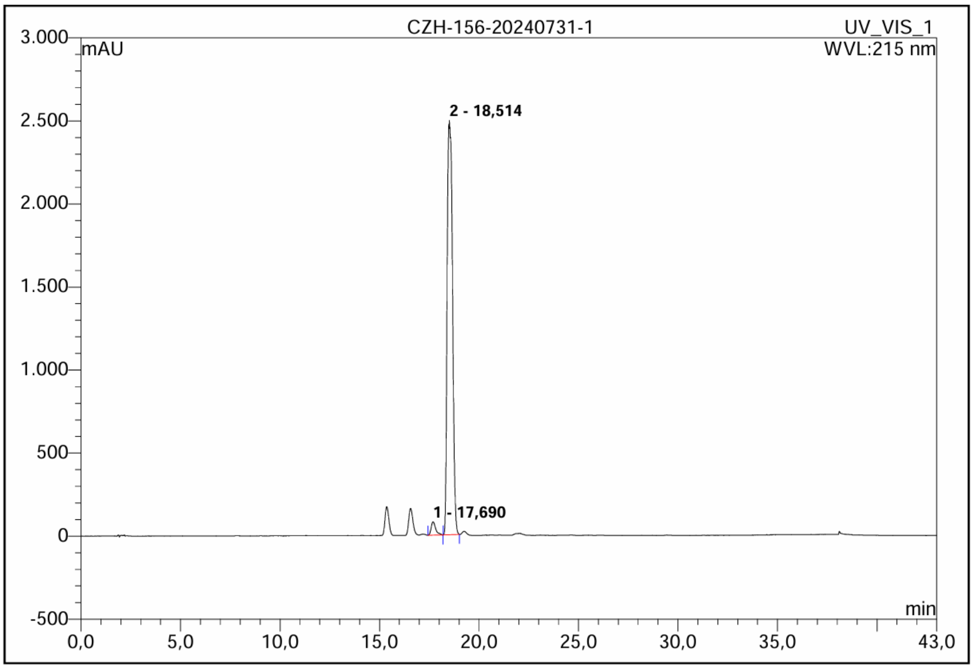

No.	Ret.Time min	Peak Name	Height mAU	Area mAU*min	Rel.Area %	Amount	Type
1	17,69	n.a.	80,249	21,261	2,69	n.a.	BMB
2	18,51	n.a.	2493,457	770,219	97,31	n.a.	BMB*
Total:			2573,706	791,480	100,00	0,000	

(*R*)-6-Fluoro-4-phenyl-3,4-dihydroquinolin-2(1*H*)-one (1h)

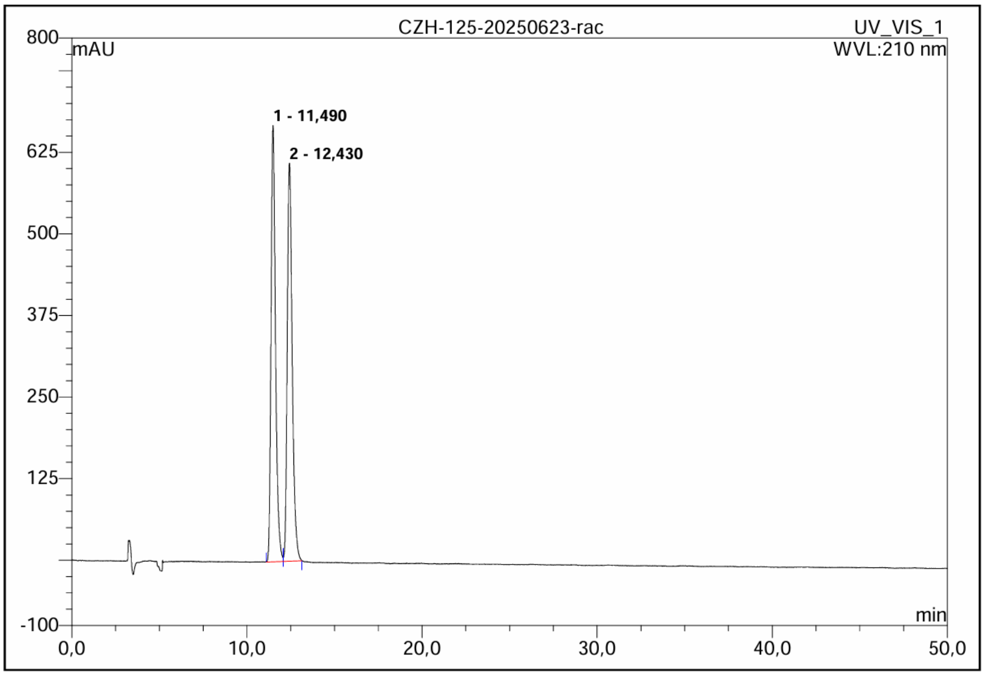

No.	Ret.Time min	Peak Name	Height mAU	Area mAU*min	Rel.Area %	Amount	Type
1	11,49	n.a.	668,627	200,113	50,00	n.a.	BM
2	12,43	n.a.	609,749	200,143	50,00	n.a.	MB
Total:			1278,376	400,256	100,00	0,000	

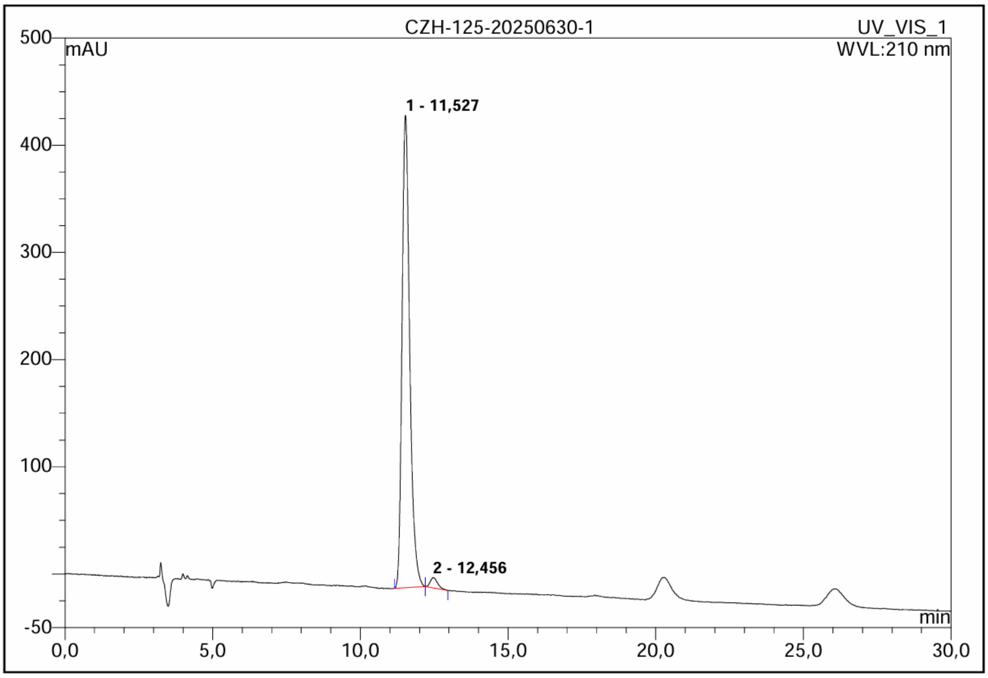

No.	Ret.Time min	Peak Name	Height mAU	Area mAU*min	Rel.Area %	Amount	Type
1	11,53	n.a.	440,616	132,254	97,93	n.a.	BMB
2	12,46	n.a.	9,621	2,791	2,07	n.a.	BMB*
Total:			450,237	135,045	100,00	0,000	

(*R*)-7-Methyl-4-phenyl-3,4-dihydroquinolin-2(1H)-one (1i)

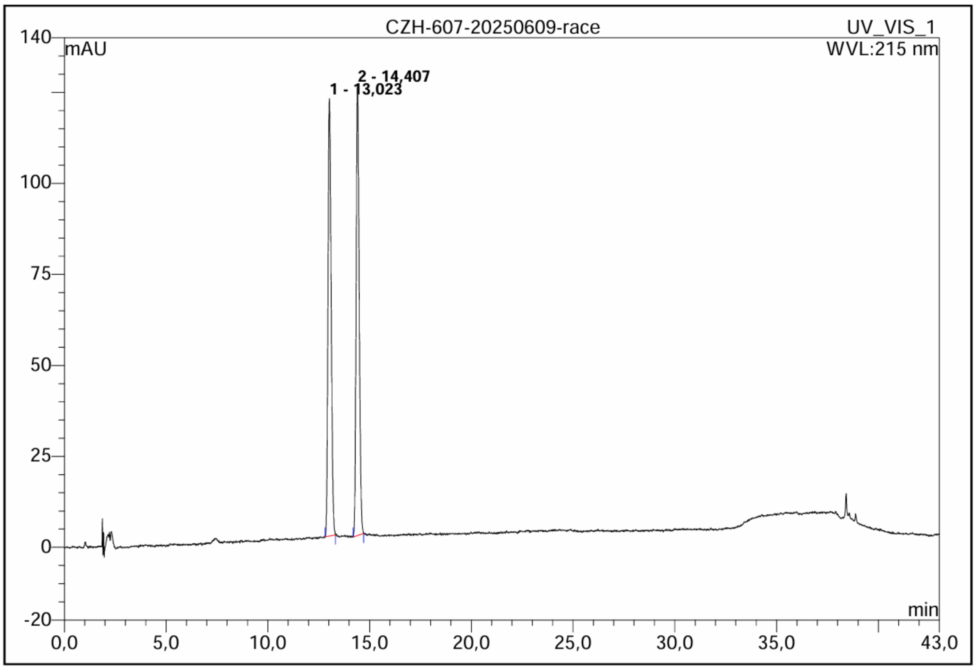

No.	Ret.Time min	Peak Name	Height mAU	Area mAU*min	Rel.Area %	Amount	Type
1	13,02	n.a.	120,236	21,711	49,90	n.a.	BMB
2	14,41	n.a.	123,605	21,797	50,10	n.a.	BMB
Total:			243,842	43,508	100,00	0,000	

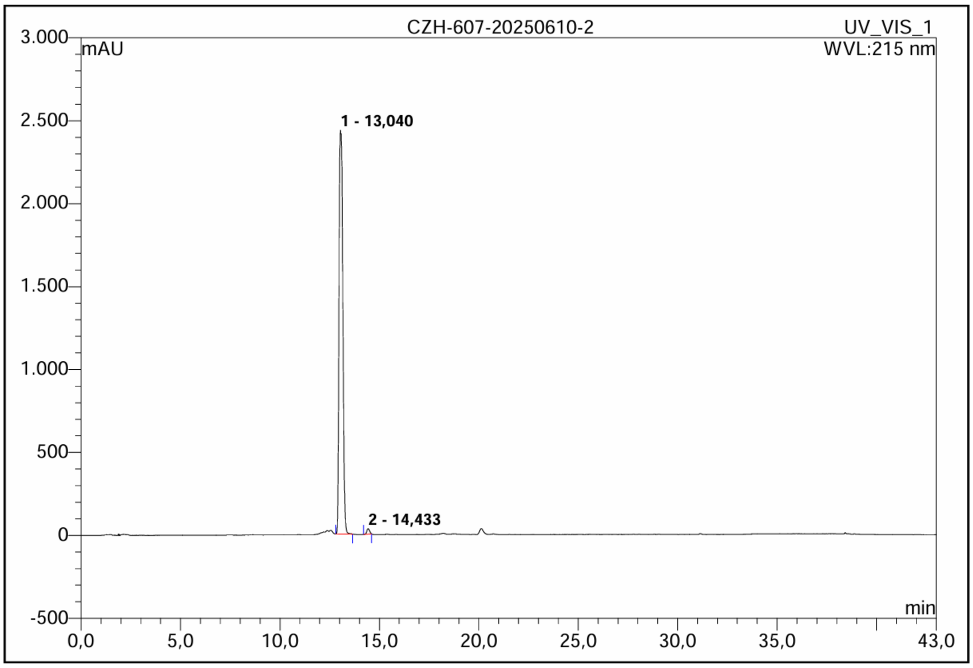

No.	Ret.Time min	Peak Name	Height mAU	Area mAU*min	Rel.Area %	Amount	Type
1	13,04	n.a.	2434,665	534,780	99,01	n.a.	BMB
2	14,43	n.a.	32,759	5,354	0,99	n.a.	BMB*
Total:			2467,424	540,135	100,00	0,000	

(*R*)-7-Methoxy-4-phenyl-3,4-dihydroquinolin-2(1H)-one (1j)

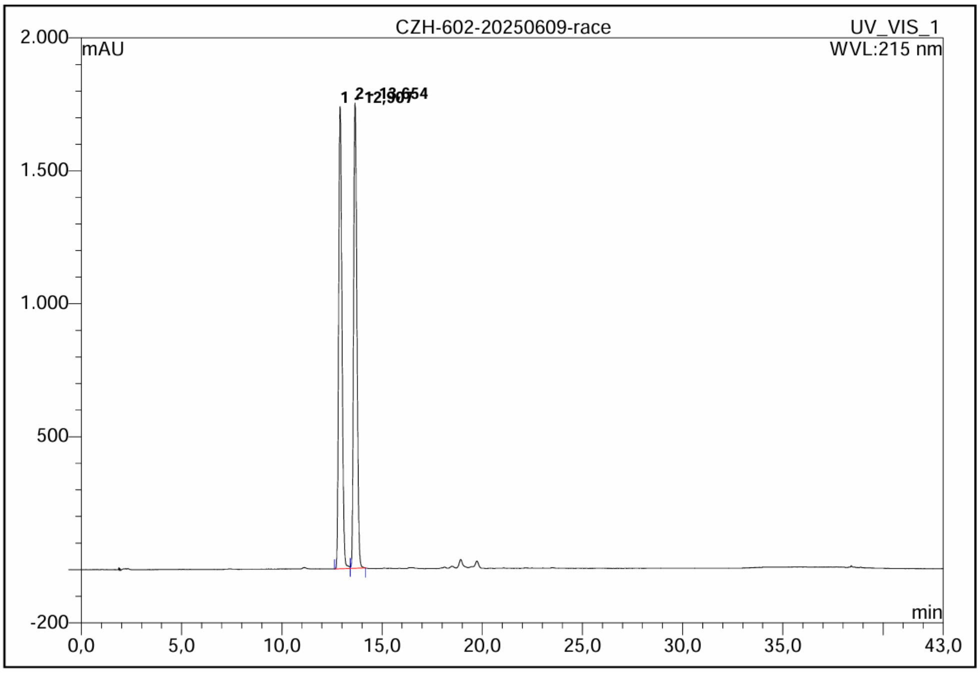

No.	Ret.Time min	Peak Name	Height mAU	Area mAU*min	Rel.Area %	Amount	Type
1	12,91	n.a.	1737,859	334,805	50,00	n.a.	BM
2	13,65	n.a.	1749,867	334,785	50,00	n.a.	MB
Total:			3487,726	669,590	100,00	0,000	

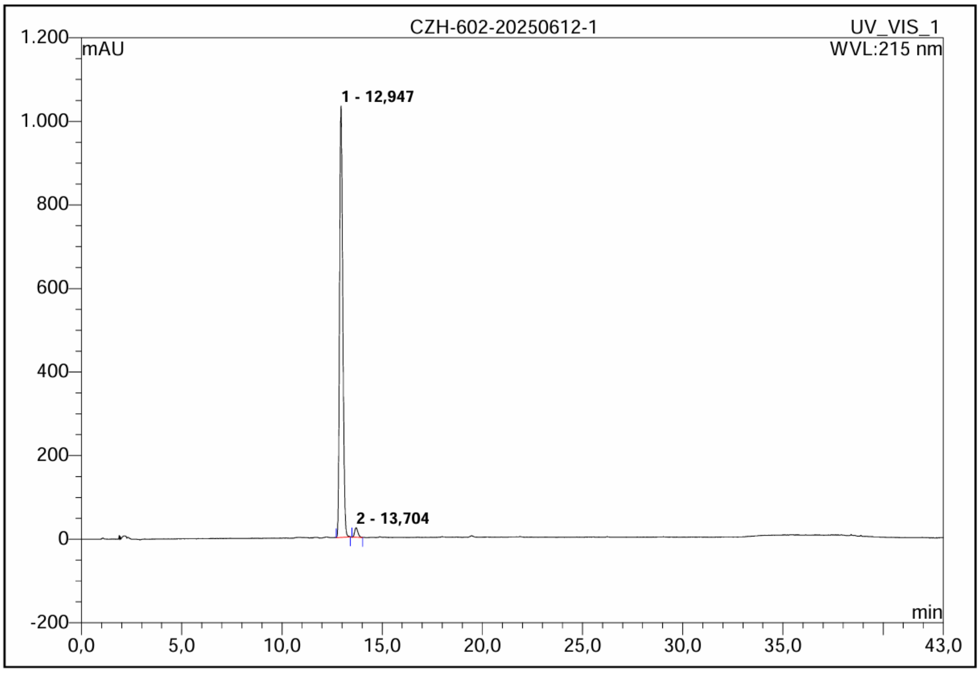

No.	Ret.Time min	Peak Name	Height mAU	Area mAU*min	Rel.Area %	Amount	Type
1	12,95	n.a.	1032,516	191,464	97,85	n.a.	BMB
2	13,70	n.a.	22,578	4,216	2,15	n.a.	BMB*
Total:			1055,095	195,680	100,00	0,000	

(*R*)-7-Chloro-4-phenyl-3,4-dihydroquinolin-2(1*H*)-one (1k)

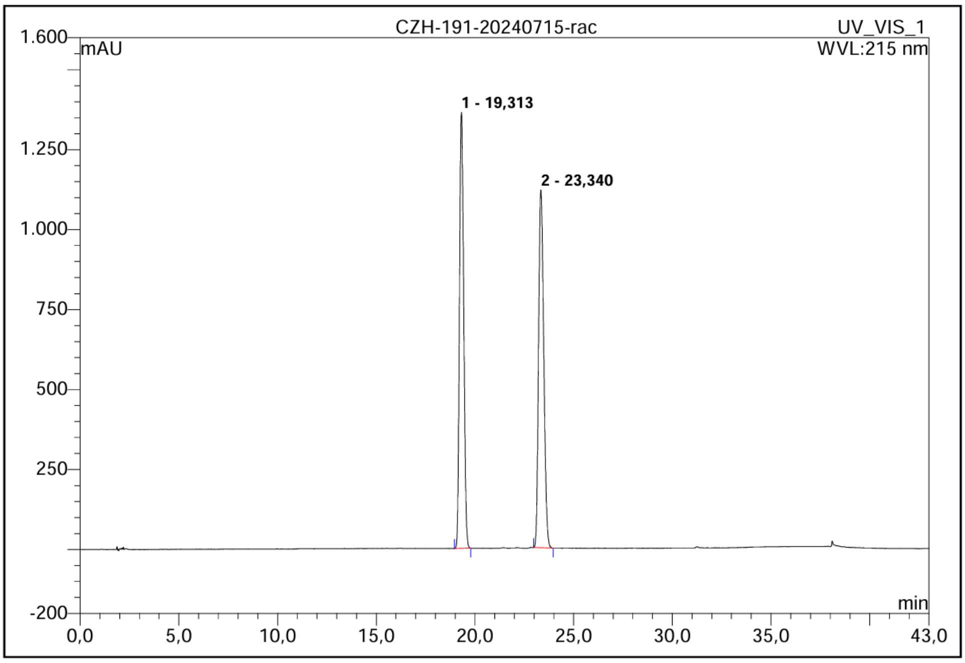

No.	Ret.Time min	Peak Name	Height mAU	Area mAU*min	Rel.Area %	Amount	Type
1	19,31	n.a.	1362,791	339,096	49,85	n.a.	BMB
2	23,34	n.a.	1118,427	341,201	50,15	n.a.	BMB
Total:			2481,218	680,297	100,00	0,000	

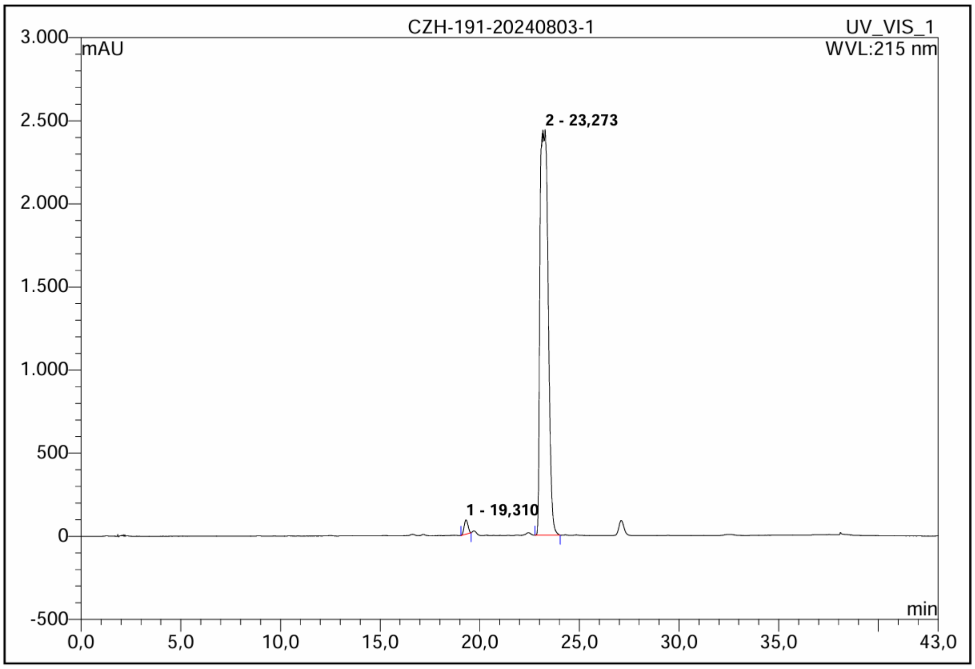

No.	Ret.Time min	Peak Name	Height mAU	Area mAU*min	Rel.Area %	Amount	Type
1	19,31	n.a.	85,473	19,054	1,62	n.a.	BMB
2	23,27	n.a.	2439,917	1157,321	98,38	n.a.	BMB
Total:			2525,390	1176,374	100,00	0,000	

(*S*)-4-Methyl-3,4-dihydroquinolin-2(1*H*)-one (1l)

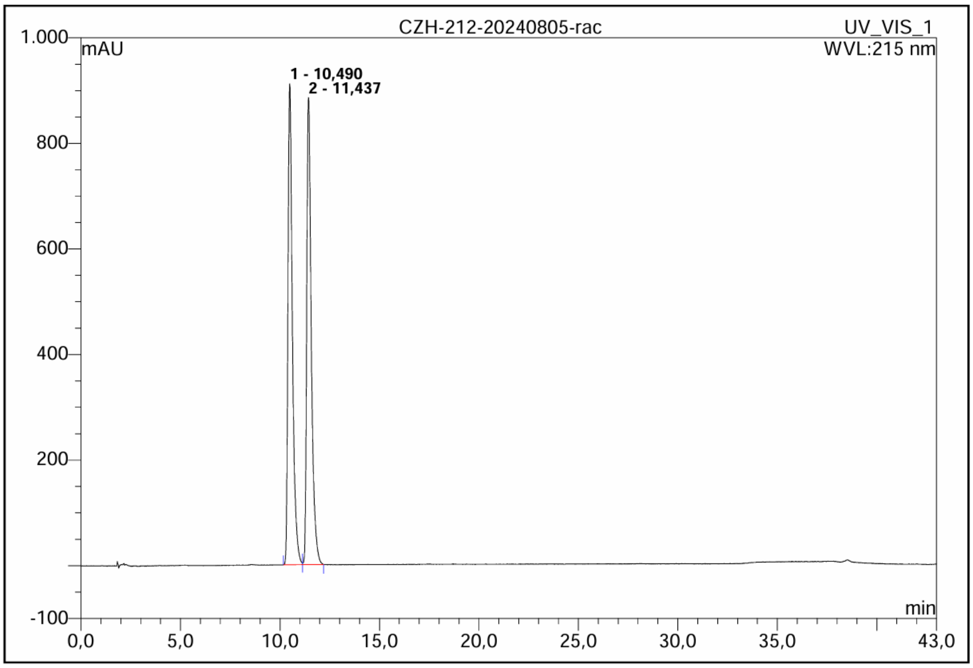

No.	Ret.Time min	Peak Name	Height mAU	Area mAU*min	Rel.Area %	Amount	Type
1	10,49	n.a.	911,293	236,593	50,05	n.a.	BM
2	11,44	n.a.	884,733	236,103	49,95	n.a.	MB
Total:			1796,026	472,696	100,00	0,000	

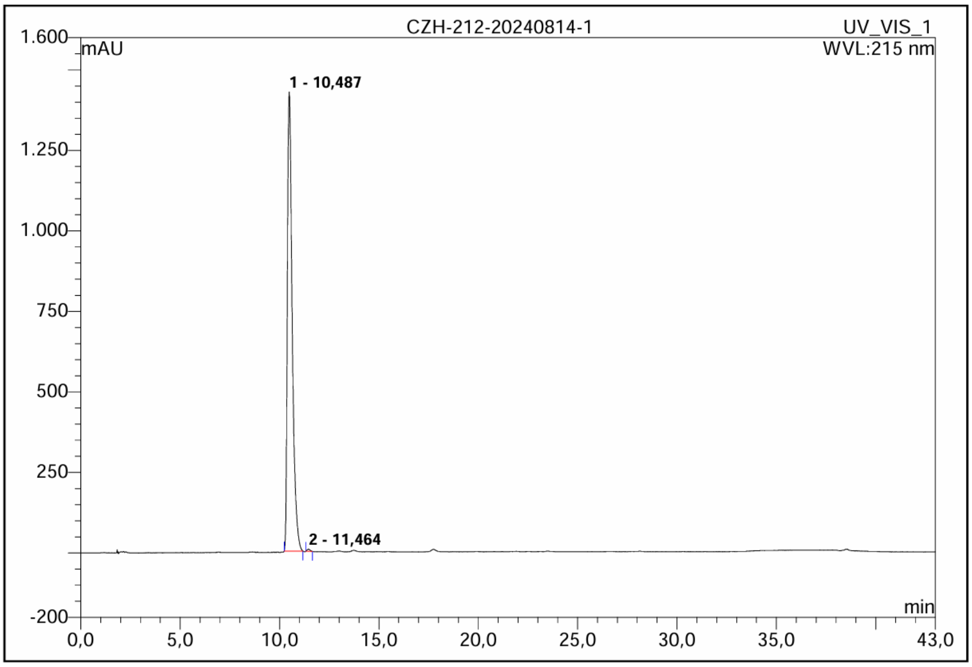

No.	Ret.Time min	Peak Name	Height mAU	Area mAU*min	Rel.Area %	Amount	Type
1	10,49	n.a.	1425,738	402,784	99,73	n.a.	BMB*
2	11,46	n.a.	6,414	1,087	0,27	n.a.	BMB*
Total:			1432,152	403,871	100,00	0,000	

(*S*)-4-Ethyl-3,4-dihydroquinolin-2(1*H*)-one (1m)

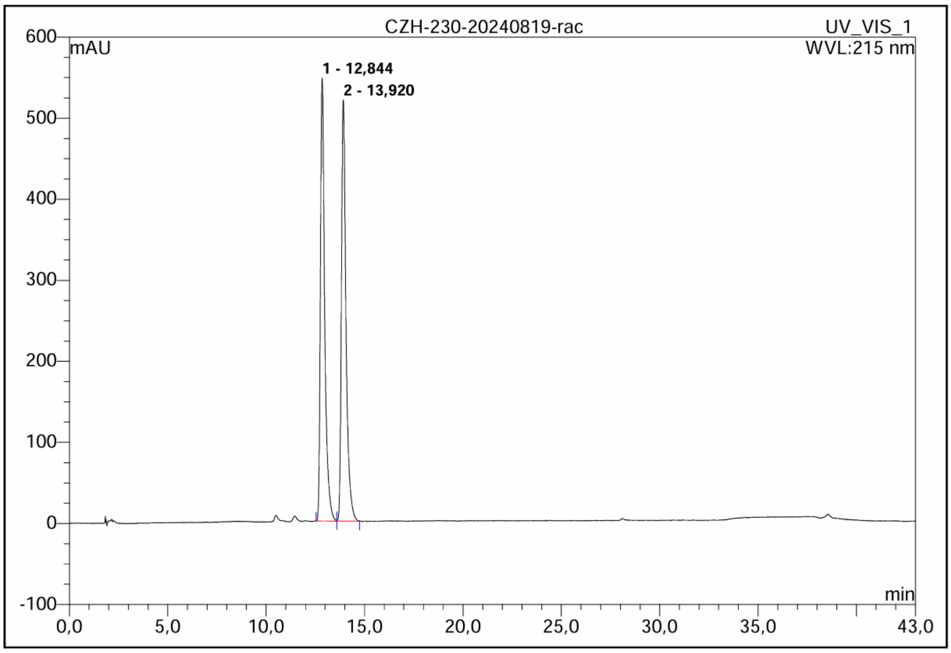

No.	Ret.Time min	Peak Name	Height mAU	Area mAU*min	Rel.Area %	Amount	Type
1	12,84	n.a.	546,602	139,895	49,96	n.a.	BM
2	13,92	n.a.	519,753	140,116	50,04	n.a.	MB
**Total:**			1066,355	280,011	100,00	0,000	

This work:

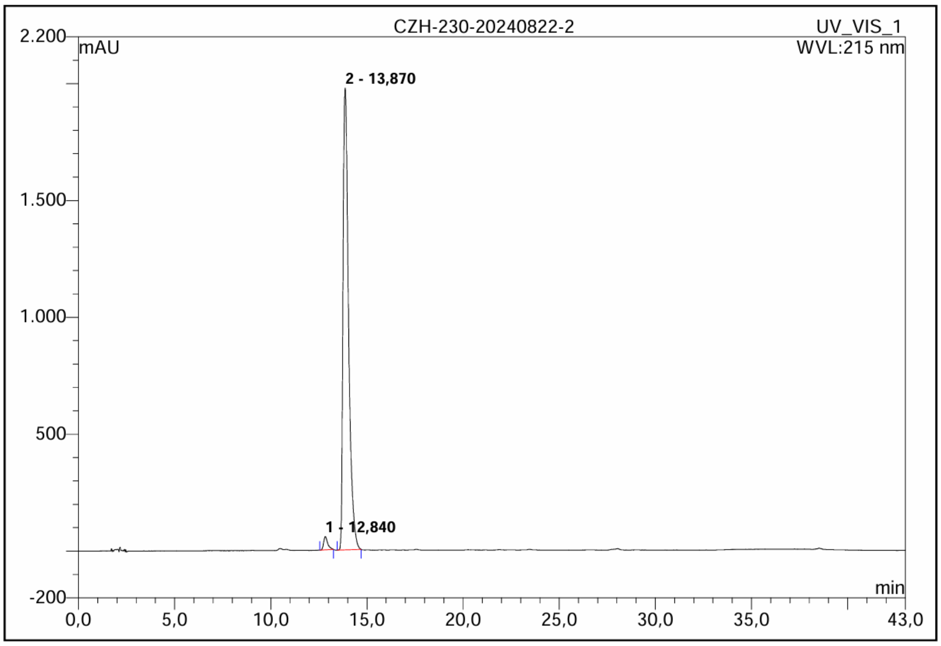

No.	Ret.Time min	Peak Name	Height mAU	Area mAU*min	Rel.Area %	Amount	Type
1	12,84	n.a.	57,455	14,046	2,07	n.a.	BMB*
2	13,87	n.a.	1975,365	663,349	97,93	n.a.	BMB*
**Total:**			2032,820	677,396	100,00	0,000	

Previous work[3]:

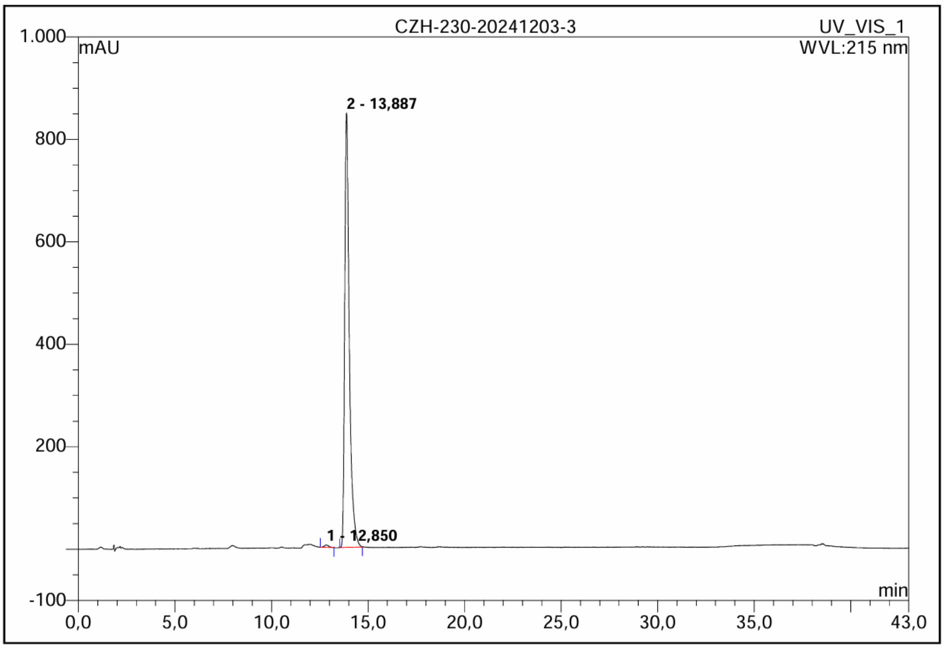

No.	Ret.Time min	Peak Name	Height mAU	Area mAU*min	Rel.Area %	Amount	Type
1	12,85	n.a.	4,805	1,303	0,55	n.a.	BMB*
2	13,89	n.a.	848,314	236,425	99,45	n.a.	BMB
Total:			853,119	237,728	100,00	0,000	

(*S*)-4-Propyl-3,4-dihydroquinolin-2(1*H*)-one (1n)

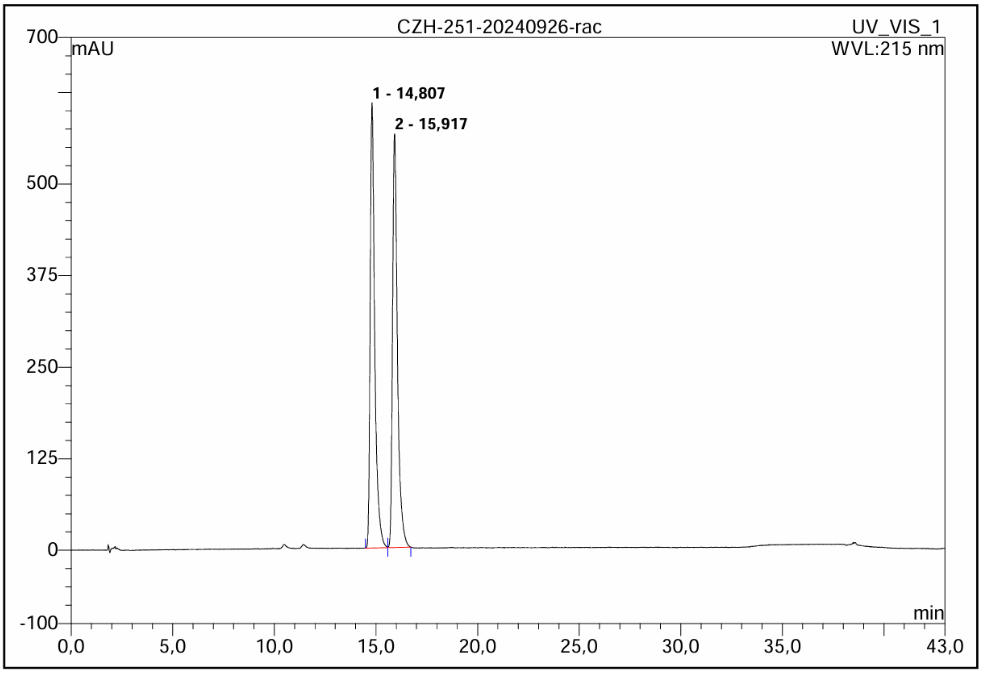

No.	Ret.Time min	Peak Name	Height mAU	Area mAU*min	Rel.Area %	Amount	Type
1	14,81	n.a.	607,567	159,929	50,23	n.a.	BM
2	15,92	n.a.	564,752	158,466	49,77	n.a.	MB
Total:			1172,319	318,395	100,00	0,000	

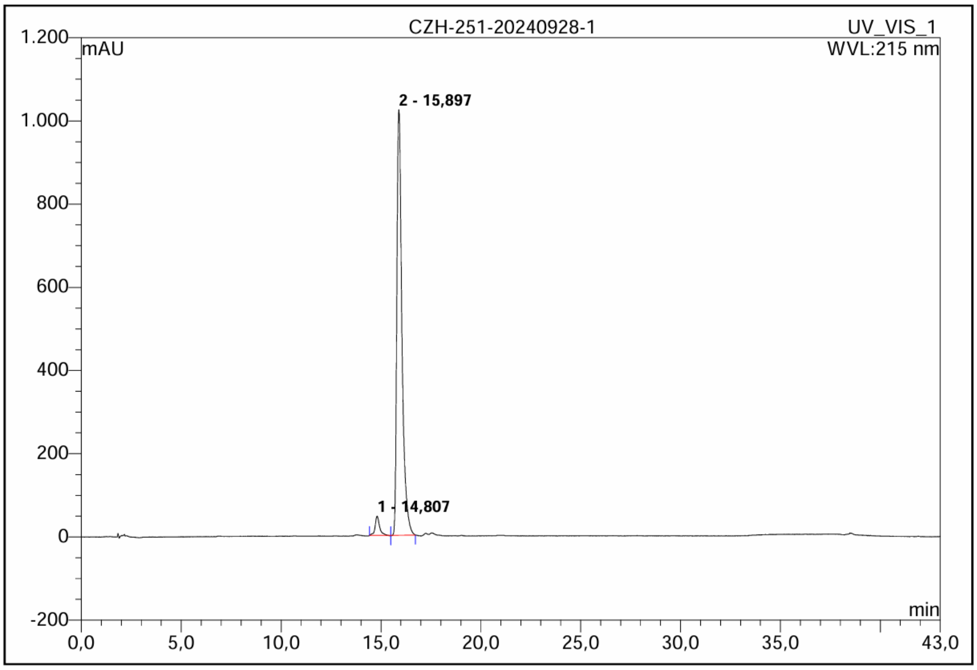

No.	Ret.Time min	Peak Name	Height mAU	Area mAU*min	Rel.Area %	Amount	Type
1	14,81	n.a.	46,632	11,977	3,80	n.a.	BM
2	15,90	n.a.	1023,238	302,879	96,20	n.a.	MB
Total:			1069,870	314,856	100,00	0,000	

(*S*)-4-Cyclopropyl-3,4-dihydroquinolin-2(1*H*)-one (1o)

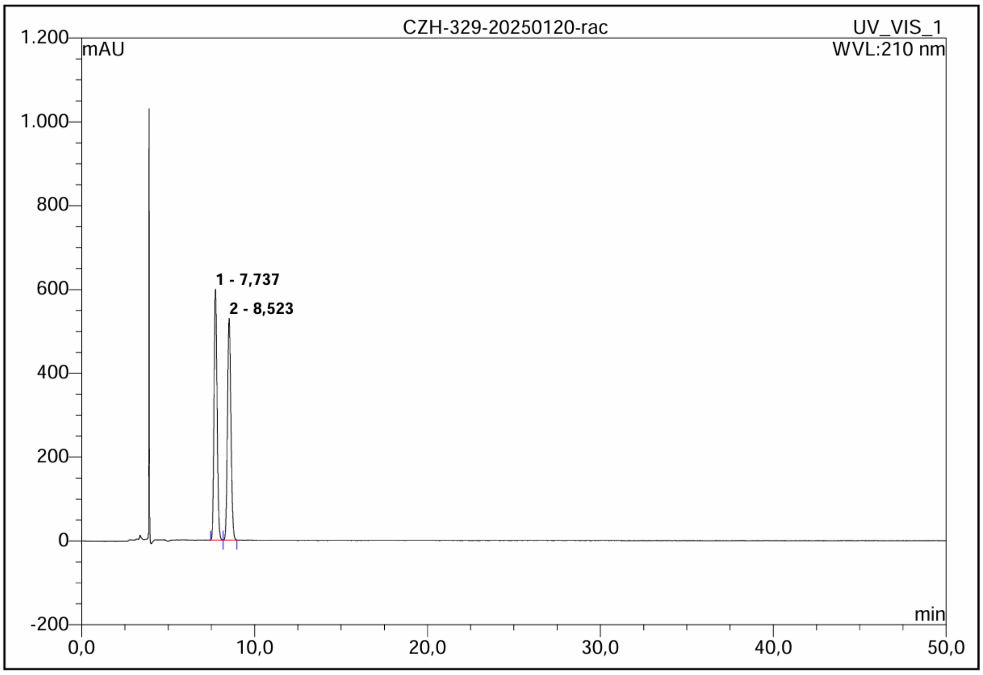

No.	Ret.Time min	Peak Name	Height mAU	Area mAU*min	Rel.Area %	Amount	Type
1	7,74	n.a.	599,118	122,159	49,98	n.a.	BM
2	8,52	n.a.	529,724	122,274	50,02	n.a.	MB
Total:			1128,842	244,433	100,00	0,000	

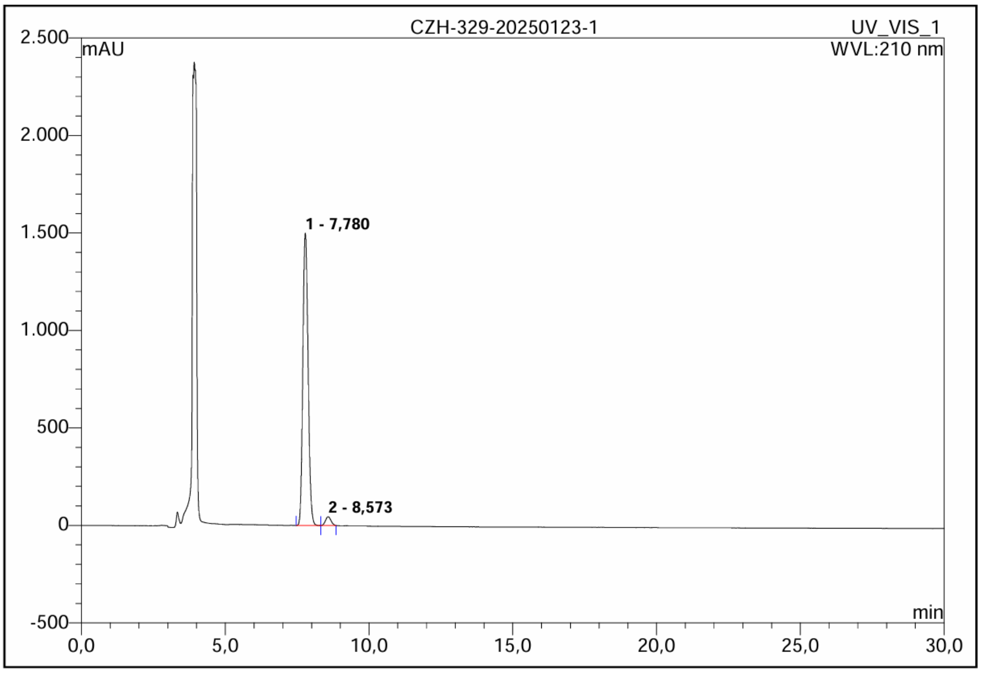

No.	Ret.Time min	Peak Name	Height mAU	Area mAU*min	Rel.Area %	Amount	Type
1	7,78	n.a.	1498,930	317,136	96,92	n.a.	BM
2	8,57	n.a.	45,298	10,074	3,08	n.a.	MB
Total:			1544,228	327,210	100,00	0,000	

(*S*)-4-Isopropyl-3,4-dihydroquinolin-2(1*H*)-one (1p)

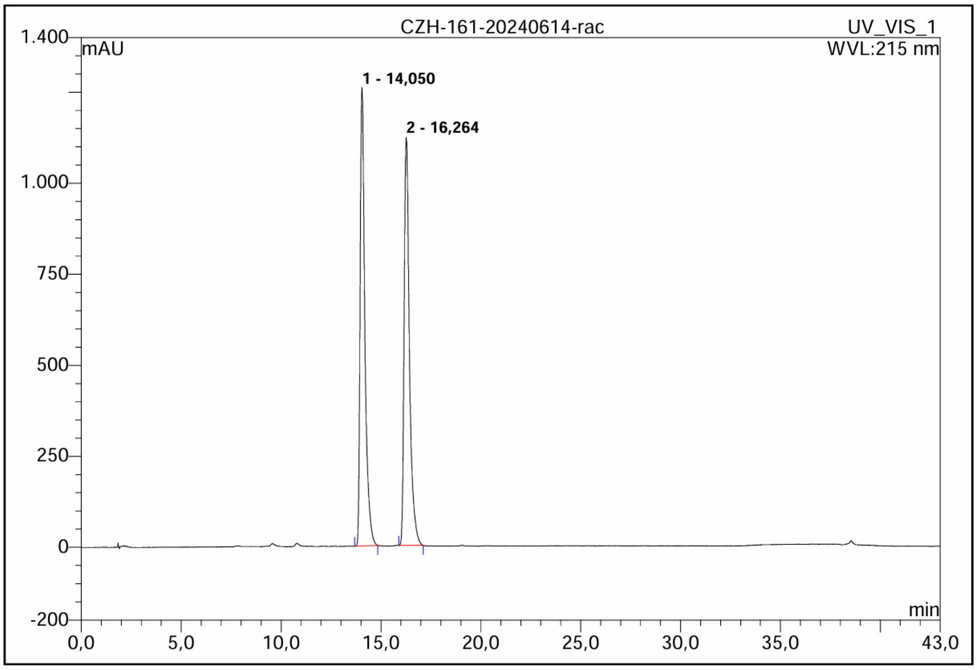

No.	Ret.Time min	Peak Name	Height mAU	Area mAU*min	Rel.Area %	Amount	Type
1	14,05	n.a.	1258,953	347,495	50,08	n.a.	BMB
2	16,26	n.a.	1120,900	346,452	49,92	n.a.	BMB
Total:			2379,853	693,947	100,00	0,000	

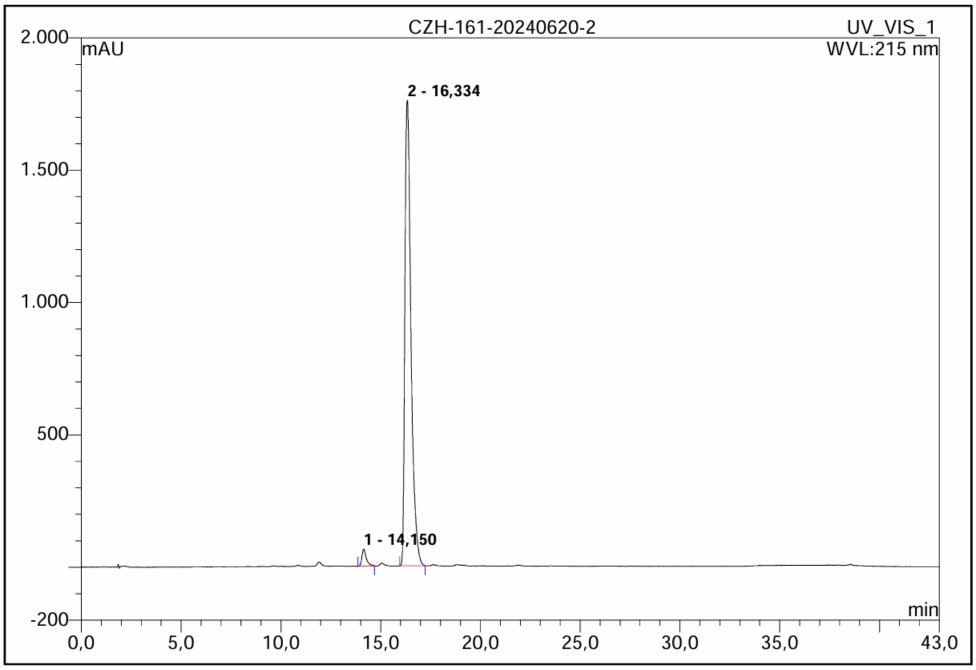

No.	Ret.Time min	Peak Name	Height mAU	Area mAU*min	Rel.Area %	Amount	Type
1	14,15	n.a.	64,117	16,233	2,53	n.a.	BMB
2	16,33	n.a.	1758,971	624,682	97,47	n.a.	BMB
Total:			1823,088	640,915	100,00	0,000	

(*S*)-4-(4,4,4-Trifluorobutyl)-3,4-dihydroquinolin-2(1*H*)-one (1q)

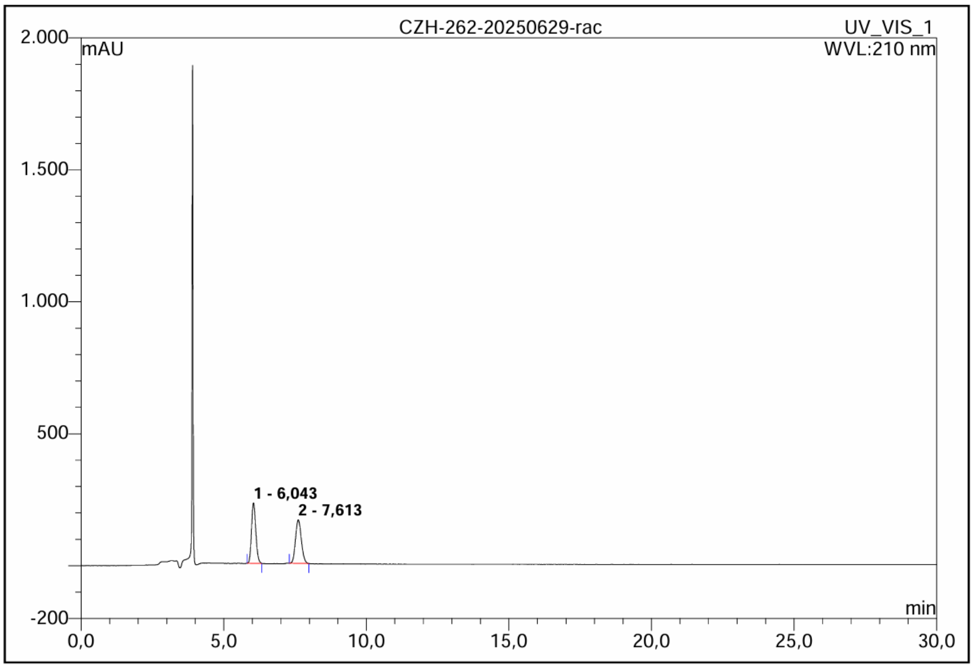

No.	Ret.Time min	Peak Name	Height mAU	Area mAU*min	Rel.Area %	Amount	Type
1	6,04	n.a.	229,625	39,864	50,49	n.a.	BMB
2	7,61	n.a.	165,053	39,090	49,51	n.a.	BMB
Total:			394,678	78,955	100,00	0,000	

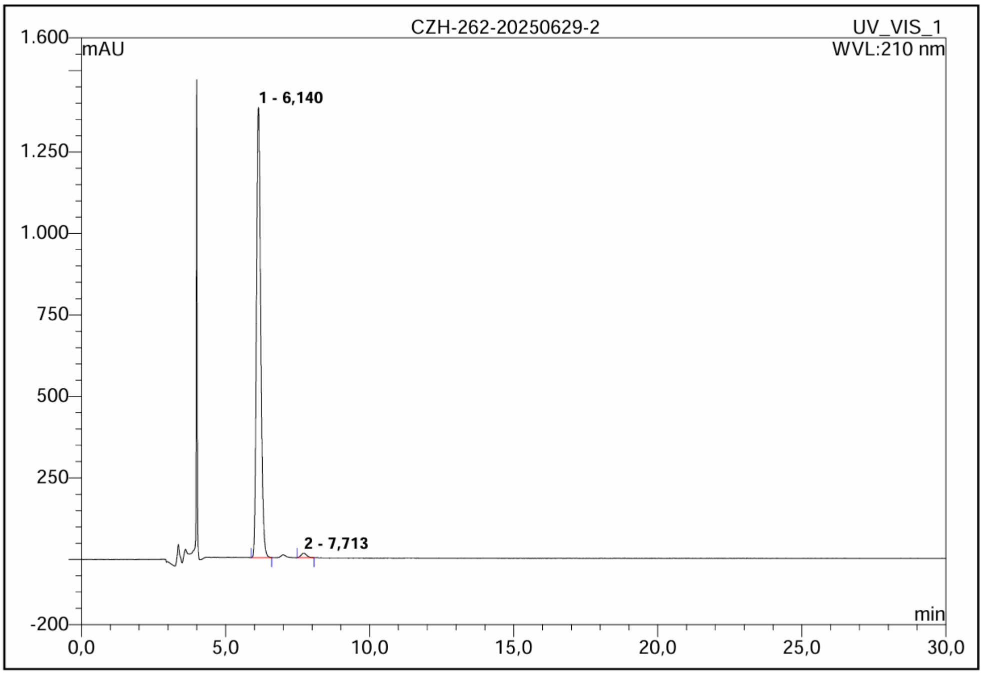

No.	Ret.Time min	Peak Name	Height mAU	Area mAU*min	Rel.Area %	Amount	Type
1	6,14	n.a.	1381,265	239,575	98,77	n.a.	BMB
2	7,71	n.a.	13,420	2,988	1,23	n.a.	BMB*
Total:			1394,685	242,564	100,00	0,000	

(*S*)-4-(2-(Trimethylsilyl)ethyl)-3,4-dihydroquinolin-2(1*H*)-one (1r)

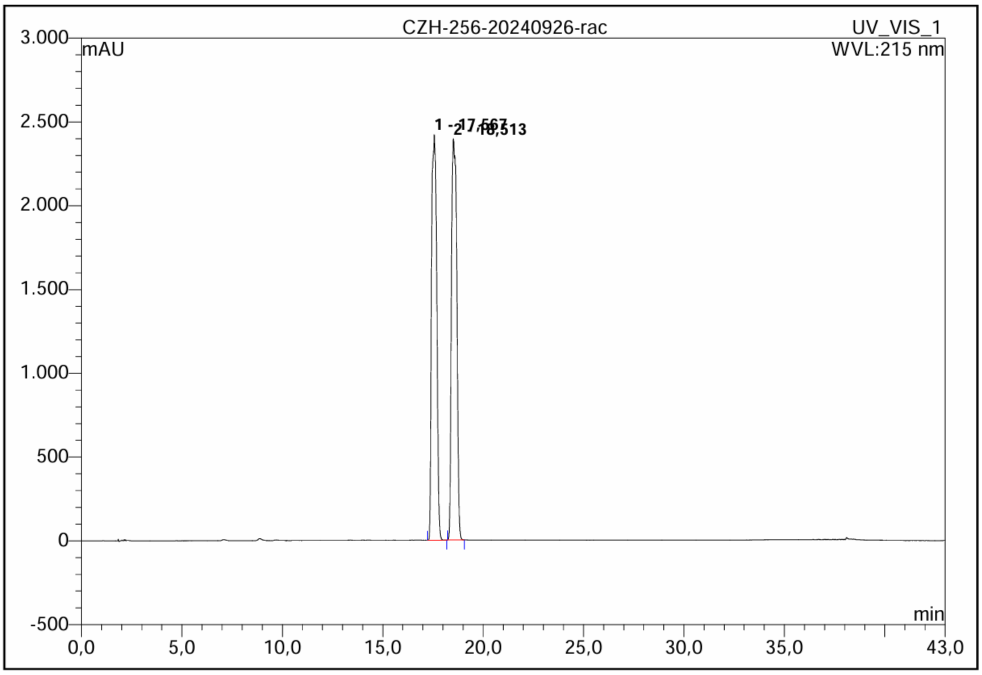

No.	Ret.Time min	Peak Name	Height mAU	Area mAU*min	Rel.Area %	Amount	Type
1	17,57	n.a.	2420,620	724,415	49,71	n.a.	BMB*
2	18,51	n.a.	2394,492	732,935	50,29	n.a.	BMB*
Total:			4815,112	1457,350	100,00	0,000	

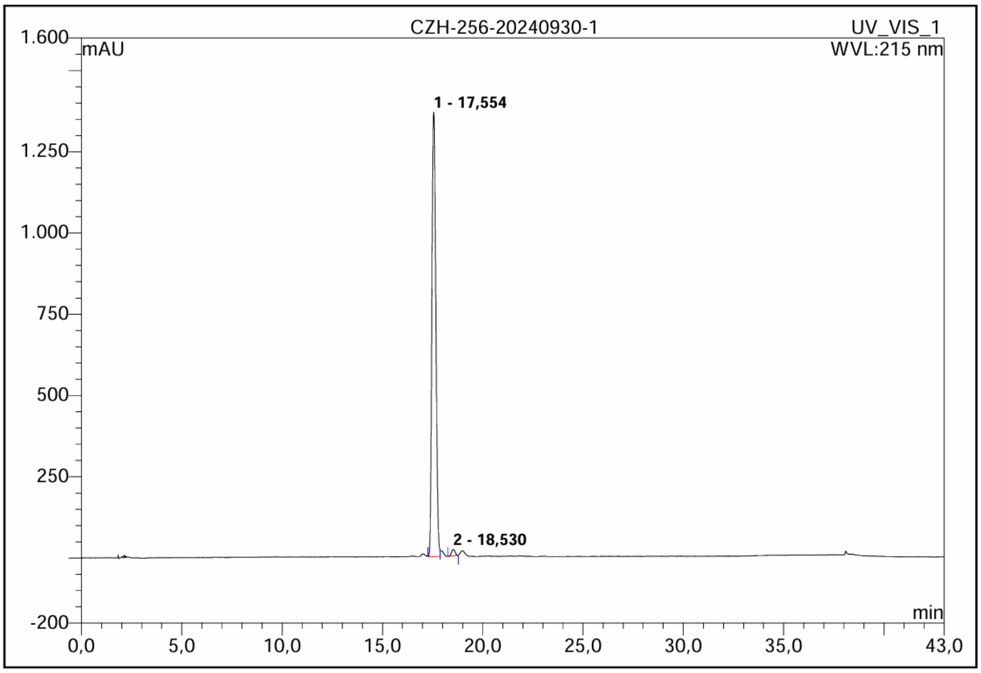

No.	Ret.Time min	Peak Name	Height mAU	Area mAU*min	Rel.Area %	Amount	Type
1	17,55	n.a.	1366,560	311,447	98,81	n.a.	BM *
2	18,53	n.a.	19,423	3,749	1,19	n.a.	BMB*
Total:			1385,984	315,196	100,00	0,000	

(*S*)-Butyl 2-oxo-1,2,3,4-tetrahydroquinoline-4-carboxylate (1s)

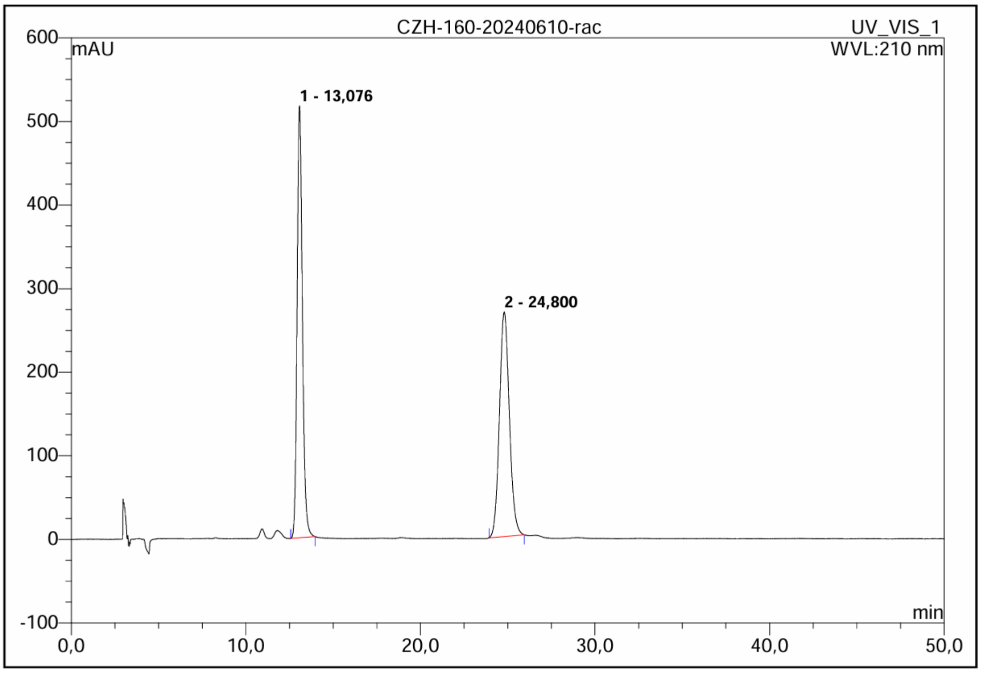

No.	Ret.Time min	Peak Name	Height mAU	Area mAU*min	Rel.Area %	Amount	Type
1	13,08	n.a.	516,859	181,022	50,58	n.a.	BMB
2	24,80	n.a.	268,671	176,893	49,42	n.a.	BMB
Total:			785,530	357,914	100,00	0,000	

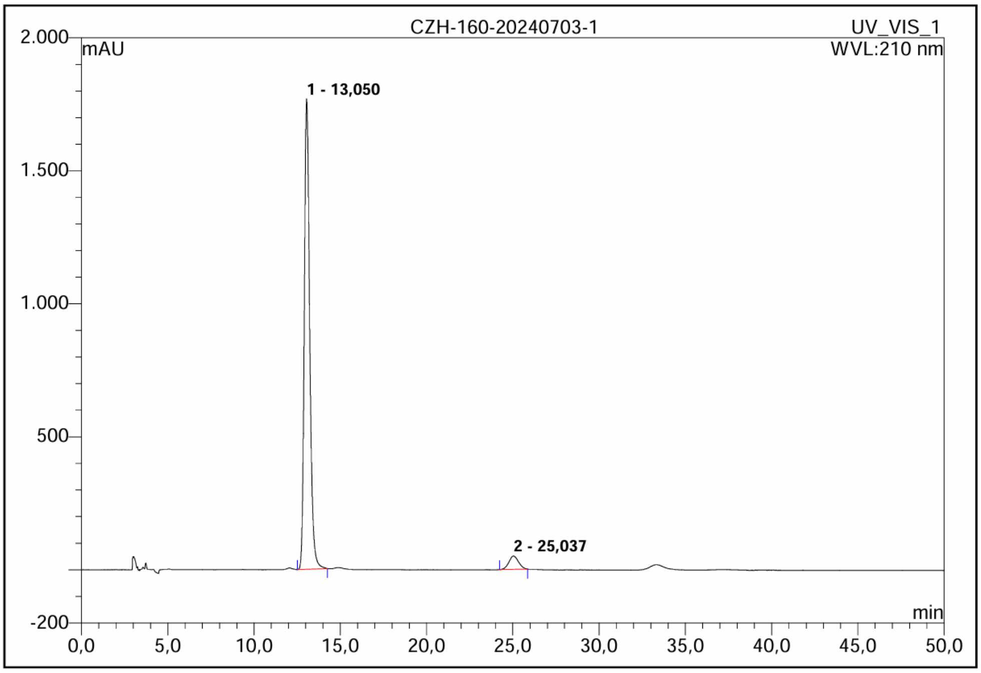

No.	Ret.Time min	Peak Name	Height mAU	Area mAU*min	Rel.Area %	Amount	Type
1	13,05	n.a.	1768,719	644,185	95,17	n.a.	BMB
2	25,04	n.a.	50,187	32,727	4,83	n.a.	BMB
Total:			1818,905	676,912	100,00	0,000	

(*R*)-4-Methyl-1,4-dihydro-2*H*-benzo[*d*][1,3]oxazin-2-one (8)

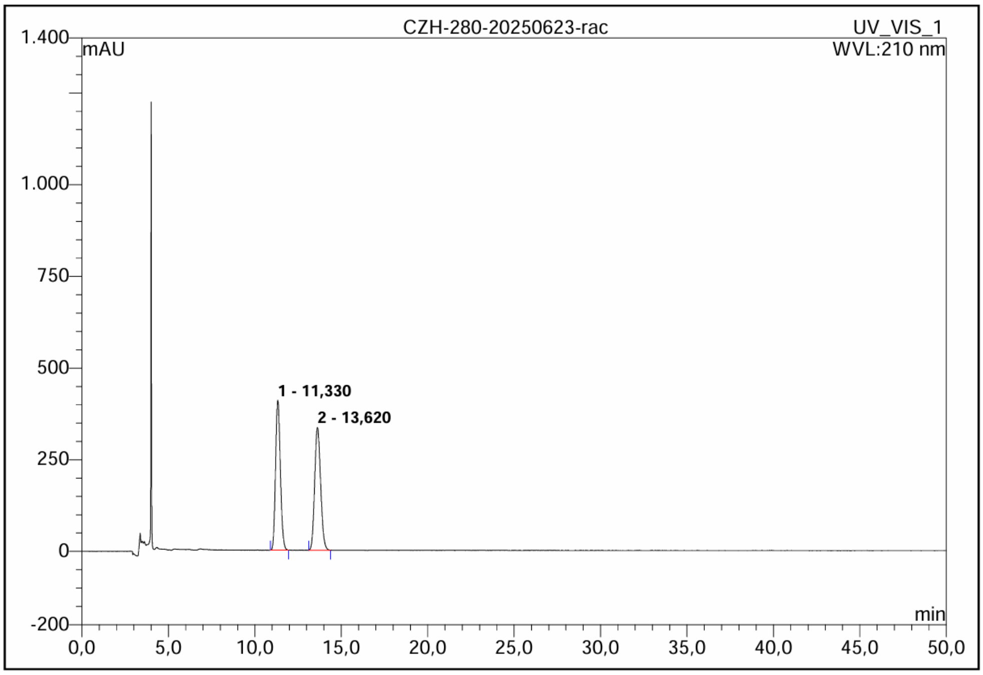

No.	Ret.Time min	Peak Name	Height mAU	Area mAU*min	Rel.Area %	Amount	Type
1	11,33	n.a.	408,597	131,472	49,62	n.a.	BMB
2	13,62	n.a.	334,535	133,480	50,38	n.a.	BMB
Total:			743,132	264,952	100,00	0,000	

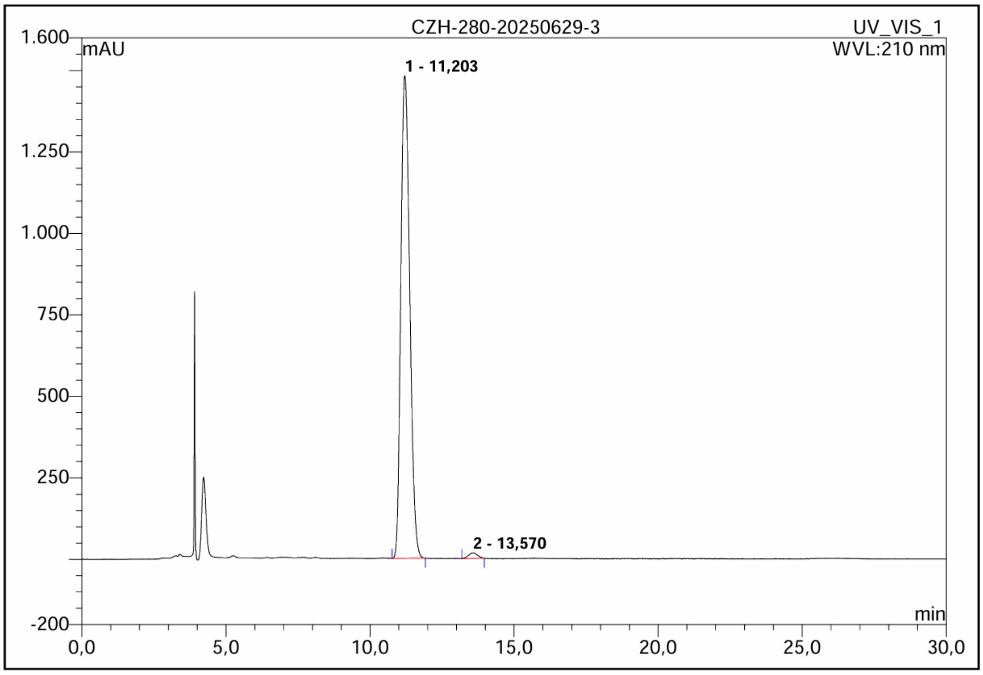

No.	Ret.Time min	Peak Name	Height mAU	Area mAU*min	Rel.Area %	Amount	Type
1	11,20	n.a.	1480,585	530,620	98,87	n.a.	BMB
2	13,57	n.a.	16,572	6,082	1,13	n.a.	BMB*
Total:			1497,157	536,702	100,00	0,000	

(*S*)-2-Methyl-2*H*-benzo[*b*][1,4]oxazin-3(4*H*)-one (9)

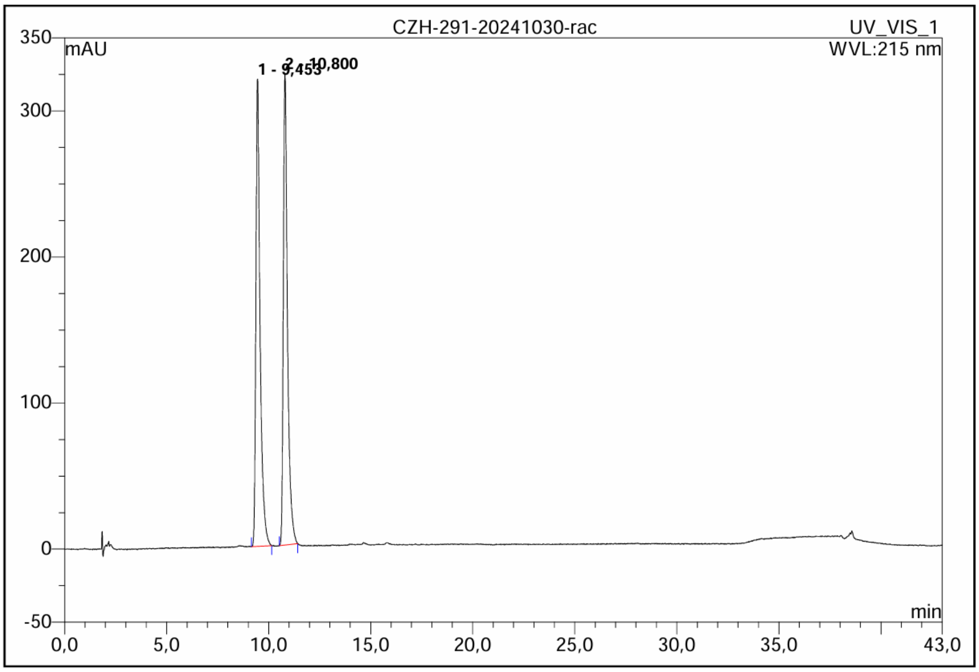

No.	Ret.Time min	Peak Name	Height mAU	Area mAU*min	Rel.Area %	Amount	Type
1	9,45	n.a.	320,046	76,840	50,11	n.a.	BMB
2	10,80	n.a.	323,096	76,499	49,89	n.a.	BMB
Total:			643,141	153,339	100,00	0,000	

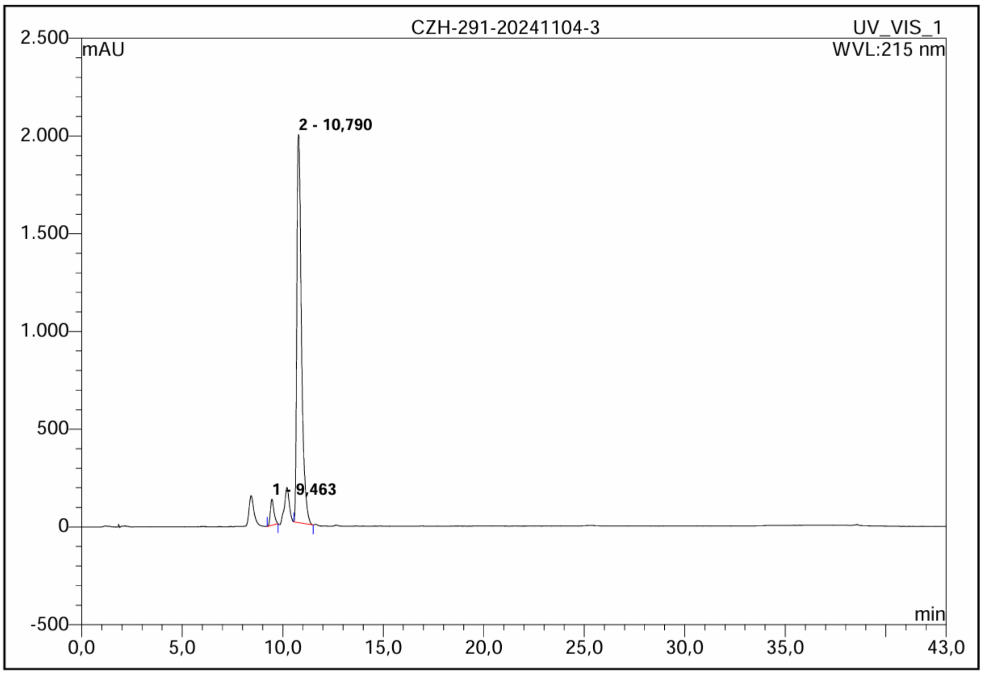

No.	Ret.Time min	Peak Name	Height mAU	Area mAU*min	Rel.Area %	Amount	Type
1	9,46	n.a.	133,829	27,765	4,95	n.a.	BMB*
2	10,79	n.a.	1985,781	533,276	95,05	n.a.	BMB*
Total:			2119,610	561,040	100,00	0,000	

(*R*)-1-Methyl-4-phenyl-3,4-dihydroquinolin-2(1*H*)-one (10)

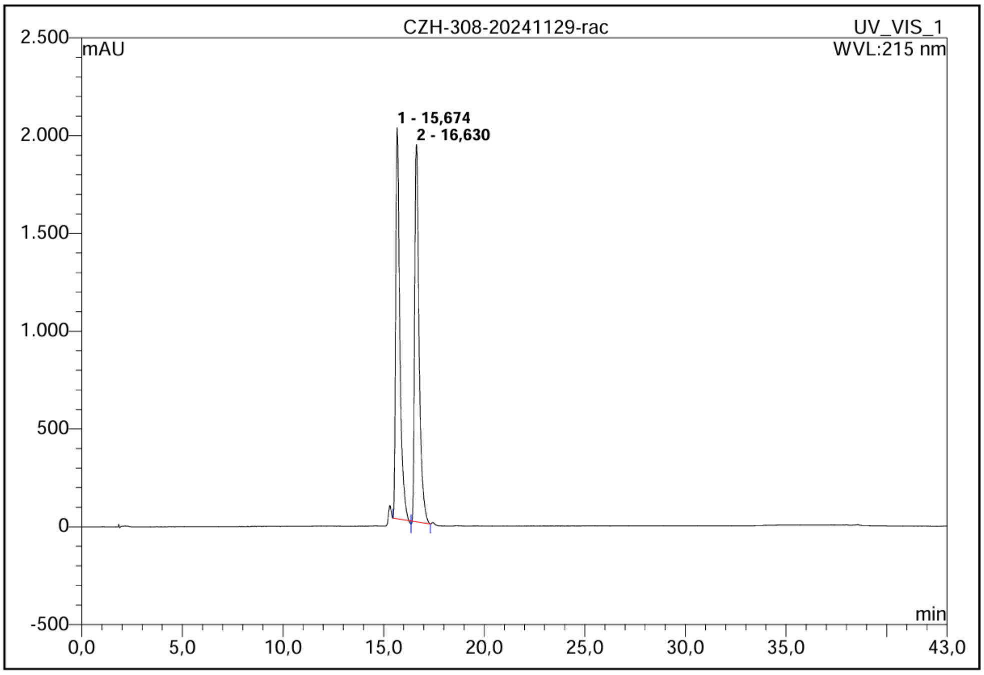

No.	Ret.Time min	Peak Name	Height mAU	Area mAU*min	Rel.Area %	Amount	Type
1	15,67	n.a.	1999,818	482,837	49,48	n.a.	BM *
2	16,63	n.a.	1929,772	493,064	50,52	n.a.	MB*
Total:			3929,590	975,901	100,00	0,000	

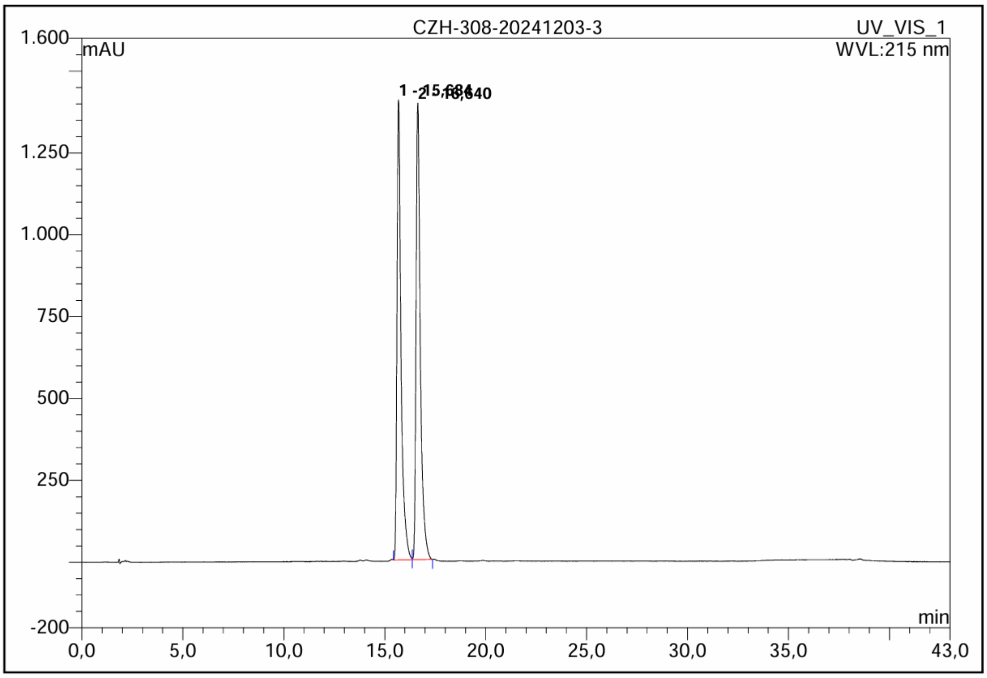

No.	Ret.Time min	Peak Name	Height mAU	Area mAU*min	Rel.Area %	Amount	Type
1	15,68	n.a.	1404,672	329,813	49,21	n.a.	BM
2	16,64	n.a.	1393,555	340,394	50,79	n.a.	MB
Total:			2798,226	670,208	100,00	0,000	

(*3S*,*4R*)-3-Phenyl-4-phenyl-3,4-dihydroquinolin-2(1*H*)-one (12)

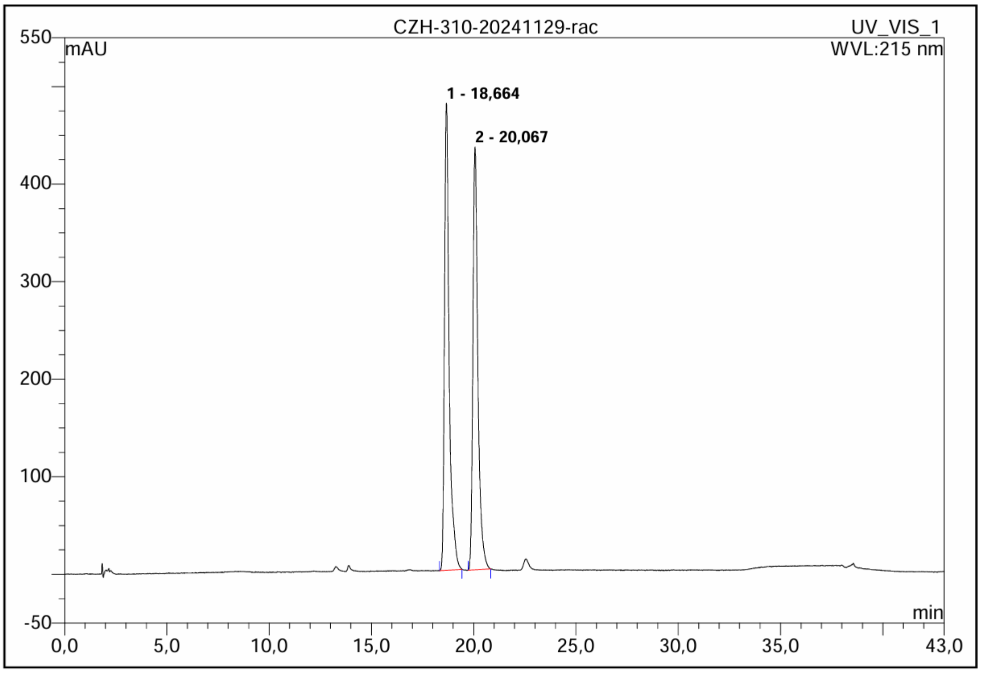

No.	Ret.Time min	Peak Name	Height mAU	Area mAU*min	Rel.Area %	Amount	Type
1	18,66	n.a.	478,666	127,209	50,80	n.a.	BMB
2	20,07	n.a.	433,447	123,217	49,20	n.a.	BMB
Total:			912,113	250,426	100,00	0,000	

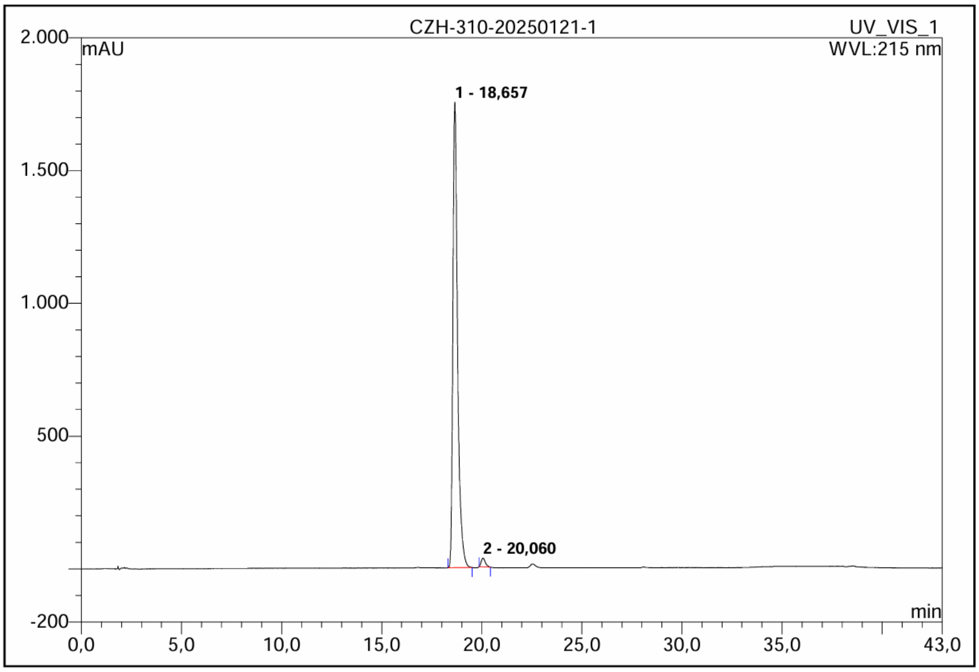

No.	Ret.Time min	Peak Name	Height mAU	Area mAU*min	Rel.Area %	Amount	Type
1	18,66	n.a.	1754,013	476,449	98,37	n.a.	BMB
2	20,06	n.a.	32,738	7,905	1,63	n.a.	BMB*
Total:			1786,750	484,354	100,00	0,000	

(*R*)-4-Phenyl-1,2,3,4-tetrahydroquinoline (13)

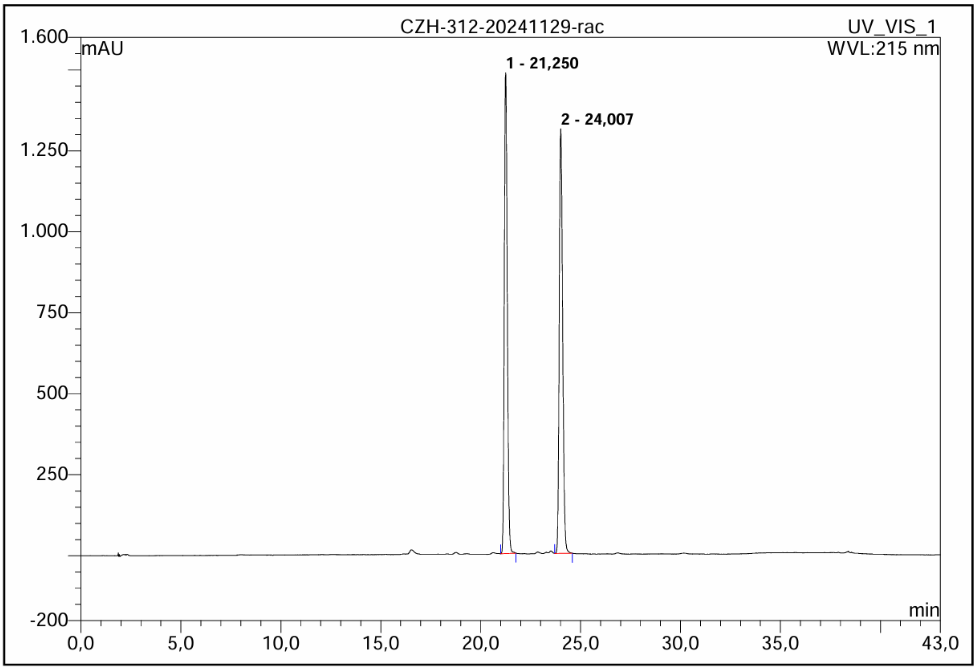

No.	Ret.Time min	Peak Name	Height mAU	Area mAU*min	Rel.Area %	Amount	Type
1	21,25	n.a.	1483,859	256,124	49,54	n.a.	BMB
2	24,01	n.a.	1310,970	260,909	50,46	n.a.	BMB
Total:			2794,829	517,033	100,00	0,000	

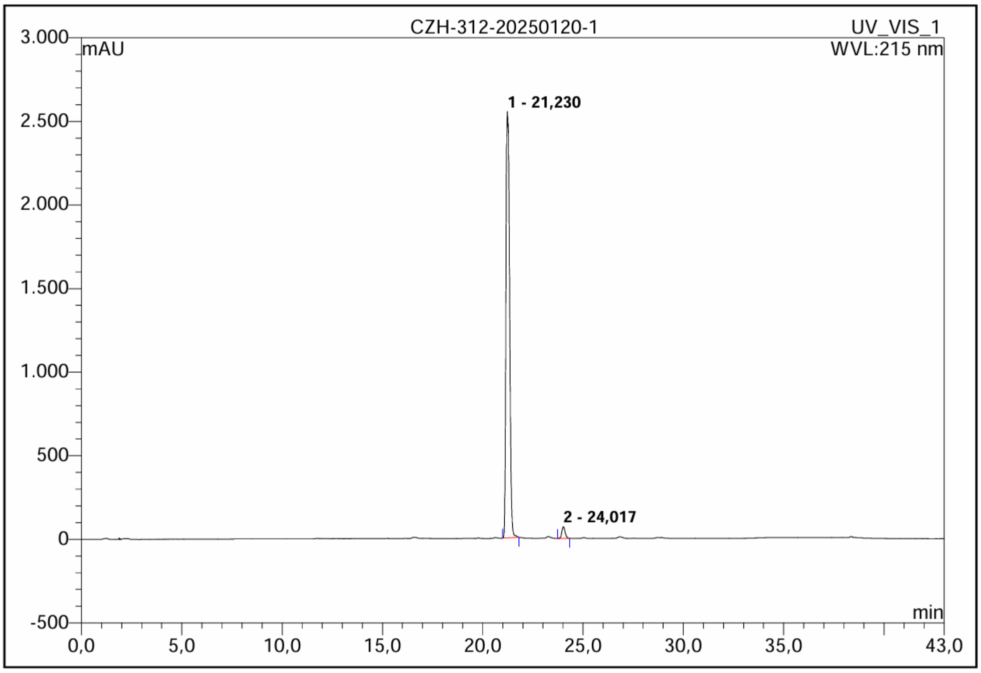

No.	Ret.Time min	Peak Name	Height mAU	Area mAU*min	Rel.Area %	Amount	Type
1	21,23	n.a.	2550,821	537,554	97,62	n.a.	BMB*
2	24,02	n.a.	69,576	13,117	2,38	n.a.	BMB*
Total:			2620,396	550,671	100,00	0,000	

(*R*)-1,4-Diphenyl-3,4-dihydroquinolin-2(1*H*)-one (14)

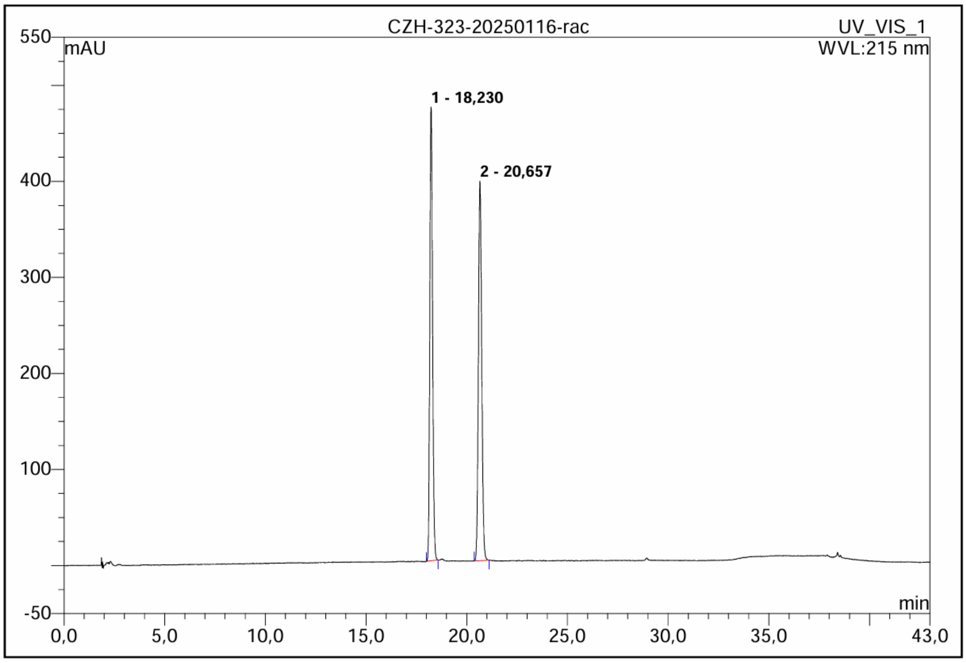

No.	Ret.Time min	Peak Name	Height mAU	Area mAU*min	Rel.Area %	Amount	Type
1	18,23	n.a.	472,442	75,247	49,78	n.a.	BMB
2	20,66	n.a.	395,200	75,926	50,22	n.a.	BMB
Total:			867,642	151,173	100,00	0,000	

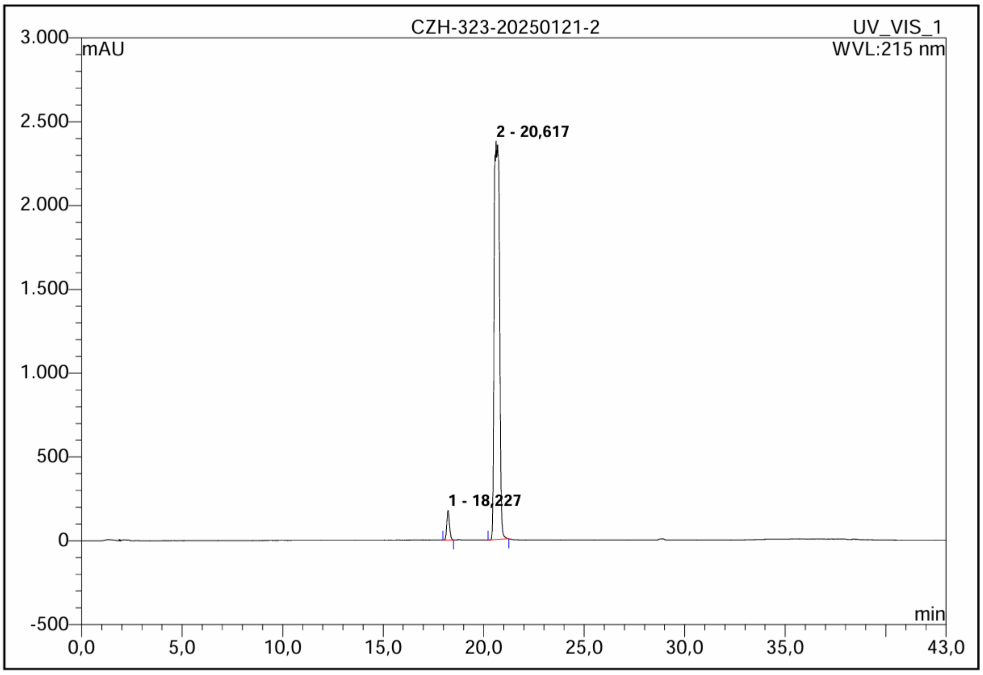

No.	Ret.Time min	Peak Name	Height mAU	Area mAU*min	Rel.Area %	Amount	Type
1	18,23	n.a.	177,384	28,030	3,67	n.a.	BMB*
2	20,62	n.a.	2377,706	735,579	96,33	n.a.	BMB*
Total:			2555,090	763,609	100,00	0,000	

(*R*)-4-Phenyl-3,4-dihydroquinoline-2(1*H*)-thione (15)

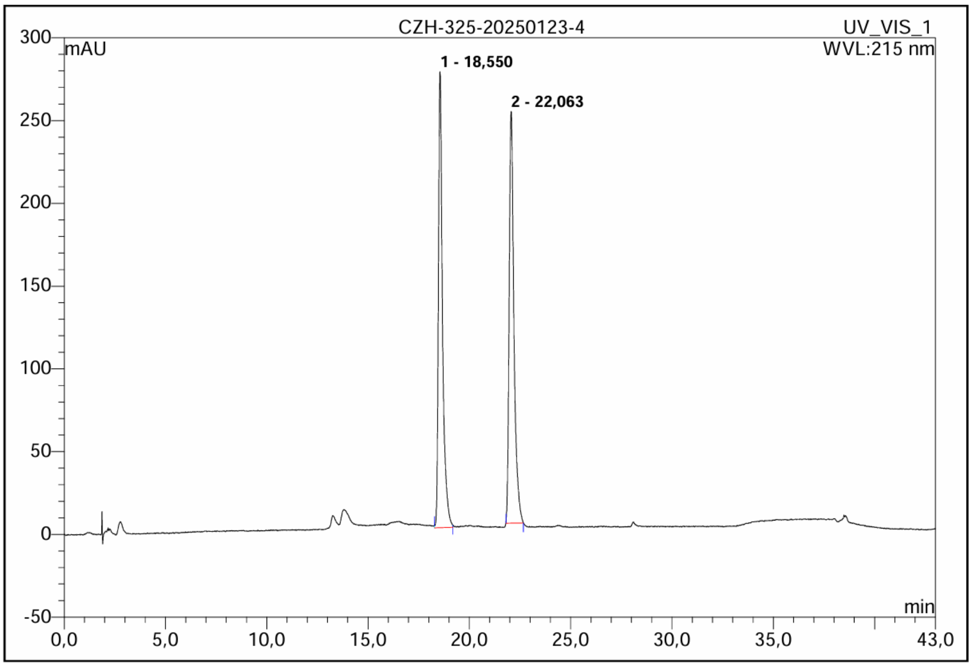

No.	Ret.Time min	Peak Name	Height mAU	Area mAU*min	Rel.Area %	Amount	Type
1	18,55	n.a.	275,571	64,961	49,05	n.a.	M *
2	22,06	n.a.	248,733	67,471	50,95	n.a.	BMB*
Total:			524,304	132,431	100,00	0,000	

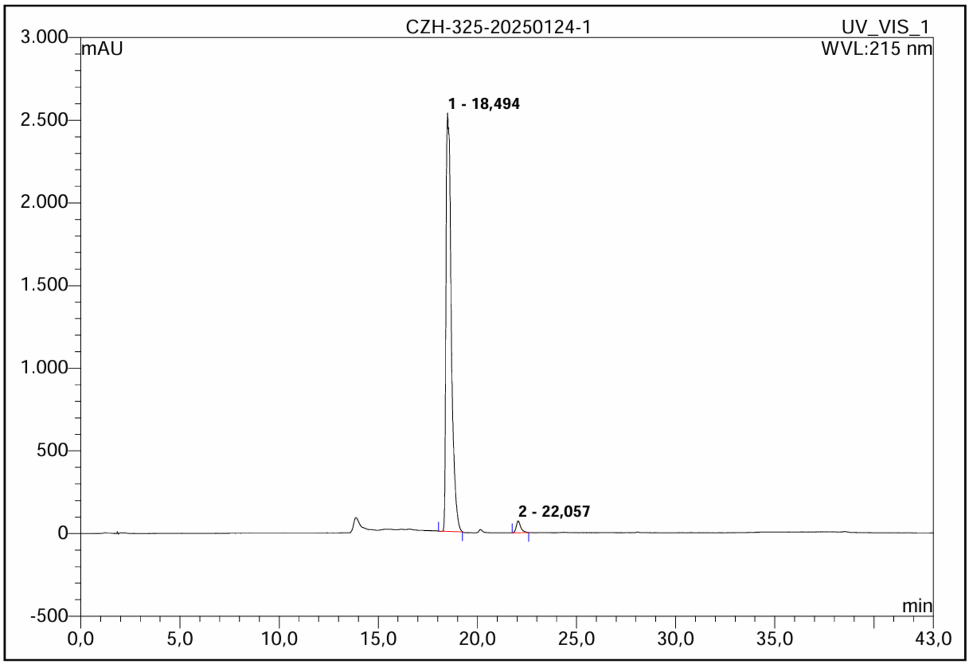

No.	Ret.Time min	Peak Name	Height mAU	Area mAU*min	Rel.Area %	Amount	Type
1	18,49	n.a.	2530,743	782,058	97,65	n.a.	BMB*
2	22,06	n.a.	71,001	18,826	2,35	n.a.	BMB
Total:			2601,744	800,885	100,00	0,000	

10. References

1. Chisholm, D. R.; Zhou, G.-L.; Pohl, E.; Valentine, R.; Whiting, A., Practical synthetic strategies towards lipophilic 6-iodotetrahydroquinolines and -dihydroquinolines. *Beilstein J. Org. Chem.* **2016,** *12*, 1851-1862.
2. Souza, B. G. d.; Choudhary, S.; Vilela, G. G.; Passos, G. F. S.; Costa, C. A. C. B.; Freitas, J. D. d.; Coelho, G. L.; Brandão, J. d. A.; Anderson, L.; Bassi, Ê. J.; Araújo-Júnior, J. X. d.; Tomar, S.; Silva-Júnior, E. F. d., Design, synthesis, antiviral evaluation, and In silico studies of acrylamides targeting nsP2 from Chikungunya virus. *Eur. J. Med. Chem.* **2023,** *258*, 115572.
3. Guo, Y.; Harutyunyan, S. R., Highly Enantioselective Catalytic Addition of Grignard Reagents to N-Heterocyclic Acceptors. *Angew. Chem., Int. Ed.* **2019,** *131*, 13084-13088.
4. Martin, O.; de la Cuesta, E.; Avendaño, C., Electrophilic substitution in 3- and 4-methyl-2(1h)quinolinone through metallated species. *Tetrahedron* **1995,** *51*, 7547-7554.
5. Zhu, B.-H.; Zhang, Y.-Q.; Xu, H.-J.; Li, L.; Deng, G.-C.; Qian, P.-C.; Deng, C.; Ye, L.-W., Regio- and Stereoselective Synthesis of Diverse 3,4-Dihydro-2-quinolones through Catalytic Hydrative Cyclization of Imine- and Carbonyl-Ynamides with Water. *ACS Catal.* **2021,** *11*, 1706-1713.
6. Matsumoto, M.; Espenson, J. H., Kinetics of the Interconversion of Parahydrogen and Orthohydrogen Catalyzed by Paramagnetic Complex Ions. *J. Am. Chem. Soc.* **2005,** *127*, 11447-11453.
7. Zhang, L.; Qureshi, Z.; Sonaglia, L.; Lautens, M., Sequential Rhodium/Palladium Catalysis: Enantioselective Formation of Dihydroquinolinones in the Presence of Achiral and Chiral Ligands. *Angew. Chem., Int. Ed.* **2014,** *53*, 13850-13853.
8. Horn, J.; Li, H. Y.; Marsden, S. P.; Nelson, A.; Shearer, R. J.; Campbell, A. J.; House, D.; Weingarten, G. G., Convergent synthesis of dihydroquinolones from o-aminoarylboronates. *Tetrahedron* **2009,** *65*, 9002-9007.
9. Park, J. O.; Youn, S. W., Rhodium-Catalyzed Domino Conjugate Addition−Cyclization Reactions for the Synthesis of a Variety of *N*- and *O*-Heterocycles: Arylboroxines as Effective Carbon Nucleophiles. *Org. Lett.* **2010**, 12, 2258–2261.
10. Sun, W.; Au, C.-M.; Wong, K.-W.; Chan, K. L.; Ngai, C. K.; Lee, H. K.; Lin, Z.; Yu, W.-Y., Intramolecular Arene C(sp2)–H Amidation Enabled by Ferrocenium-Mediated Decomposition of 1,4,2-Dioxazol-5-ones as Amidyl Radical Precursors. *ACS Catal.* **2023,** *13*, 11389-11398.
11. Corpas, J.; Mauleón, P.; Gómez Arrayás, R.; Carretero, J. C., anti-Hydroarylation of Activated Internal Alkynes: Merging Pd and Energy Transfer Catalysis. *Org. Lett.* **2020,** *22*, 6473-6478.
12. Westland, R. D.; Cooley, R. A., Jr.; Holmes, J. L.; Hong, J. S.; Lin, M. H.; Zwiesler, M. L., Antiradiation agents. Substituted 2-pyridyloxy and 2-quinolyloxy derivatives of S-2-(alkylamino)ethyl hydrogen thiosulfates and 3-alkylthiazolidines and substituted 2-pyridyloxy derivatives of 2-(alkylamino)ethanethiols and corresponding disulfides. *J. Med. Chem.* **1973,** *16*, 319-327.
13. Porey, A.; Santra, S.; Guin, J., Highly Enantioselective Synthesis of Functionalized Glutarimide Using Oxidative N-Heterocyclic Carbene Catalysis: A Formal Synthesis of (−)-Paroxetine. *J. Org. Chem.* **2019,** *84*, 5313-5327.
14. de Robichon, M.; Kratz, T.; Beyer, F.; Zuber, J.; Merten, C.; Bach, T., Enantioselective, Intermolecular [π2+σ2] Photocycloaddition Reactions of 2(1H)-Quinolones and Bicyclo[1.1.0]butanes. *J. Am. Chem. Soc.* **2023,** *145*, 24466-24470.
15. Kamei, Y.; Seino, Y.; Yamaguchi, Y.; Yoshino, T.; Maeda, S.; Kojima, M.; Matsunaga, S., Silane- and peroxide-free hydrogen atom transfer hydrogenation using ascorbic acid and cobalt-photoredox dual catalysis. *Nat. Commun.* **2021,** *12*, 966.

16. Zhang, P.; Terefenko, E. A.; Fensome, A.; Wrobel, J.; Winneker, R.; Lundeen, S.; Marschke, K. B.; Zhang, Z., 6-Aryl-1,4-dihydro-benzo[d][1,3]oxazin- 2-ones: A Novel Class of Potent, Selective, and Orally Active Nonsteroidal Progesterone Receptor Antagonists. *J. Med. Chem.* **2002,** *45*, 4379-4382.
17. Zhang, L.; Zhang, S., Method for the Synthesis of 2H-1,4-Benzoxazin-3-(4H)-ones via Ligand-Free Copper-Catalyzed Cascade Reaction. *Synthesis* **2023,** *55*, 3179-3185.
18. Kobayashi, K.; Fuchimoto, Y.; Hayashi, K.; Mano, M.; Tanmatsu, M.; Morikawa, O.; Konishi, H., A Convenient Synthesis of 3-(1-Aminoalkyl)quinolin-2(1H)-one Derivatives. *Synthesis* **2005,** *2005*, 2673-2676.
19. Hu, T.; Lückemeier, L.; Daniliuc, C.; Glorius, F., Ru-NHC-Catalyzed Asymmetric Hydrogenation of 2-Quinolones to Chiral 3,4-Dihydro-2-Quinolones. *Angew. Chem., Int. Ed.* **2021,** *60*, 23193-23196.
20. Malig, T. C.; Yu, D.; Hein, J. E., A Revised Mechanism for the Kinugasa Reaction. *J. Am. Chem. Soc.* **2018,** *140*, 9167-9173.
21. Matsuura, H.; Aoi, A.; Satou, C.; Nakaya, M.; Masuta, C.; Nabeta, K., Simultaneous UPLC MS/MS analysis of endogenous jasmonic acid, salicylic acid, and their related compounds. *Plant Growth Regul.* **2009,** *57*, 293-301.
22. Darshana, D.; Sureram, S.; Mahidol, C.; Ruchirawat, S.; Kittakoop, P., Spontaneous conversion of prenyl halides to acids: application in metal-free preparation of deuterated compounds under mild conditions. *Org. Biomol. Chem.* **2021,** *19*, 7390-7402.
23. Chen, X.; Liu, C.-Y.; Zhou, J.-H.; Zhou, X.-Y., Pd-Catalyzed Cyclization of o-Iodoanilines with Acrylates or Acrylic Acids: A Convenient One-Step Route to 2-Quinolinones. *Eur. J. Org. Chem.* **2024,** *27*, e202400744.
24. Kim, Y.; Shin, E.-k.; Beak, P.; Park, Y. S., Asymmetric Syntheses of 3,4-Substituted Tetrahydroquinoline Derivatives by (-)-Sparteine-Mediated Dynamic Thermodynamic Resolution of 2-(α-Lithiobenzyl)-N-pivaloylaniline. *Synthesis* **2006,** *2006*, 3805-3808.
25. Beadle, C. D.; Boot, J.; Camp, N. P.; Dezutter, N.; Findlay, J.; Hayhurst, L.; Masters, J. J.; Penariol, R.; Walter, M. W., 1-Aryl-3,4-dihydro-1H-quinolin-2-one derivatives, novel and selective norepinephrine reuptake inhibitors. *Bioorg. Med. Chem. Lett.* **2005,** *15*, 4432-4437.
26. Guarna, A.; Lombardi, E.; Machetti, F.; Occhiato, E. G.; Scarpi, D., Modification of the Aza-Robinson Annulation for the Synthesis of 4-Methyl-Benzo[c]quinolizin-3-ones, Potent Inhibitors of Steroid 5α-Reductase 1. *J. Org. Chem.* **2000,** *65*, 8093-8095.
